# Base-stabilized acyclic amino(ylidyl)silylenes: electron-rich donors for the stabilization of silicon-element multiple bonds†

**DOI:** 10.1039/d5sc01812a

**Published:** 2025-04-03

**Authors:** Felix Krischer, Stephan Mayer, Lennart Hensle, Daniel Knyszek, Heidar Darmandeh, Viktoria H. Gessner

**Affiliations:** a Faculty of Chemistry and Biochemistry, Ruhr-University Bochum Universitätsstrasse 150 44801 Bochum Germany viktoria.gessner@rub.de

## Abstract

Increasing the donor strength of Lewis bases is a viable strategy to stabilize reactive electron-deficient species. Herein, we utilize the strong electron-releasing power of ylide substituents to gain access to electron-rich silylenes. Based on the Roesky's amidinato chlorosilylene scaffold, we succeeded in isolating two amino(ylidyl)silylenes with a tosyl and cyano group in the ylide backbone, respectively. The tosyl system revealed to be amongst the most electron-rich silylenes known to date as measured by its Tolman electronic parameter. DFT studies showed that the ylide acts as a σ and π-donor, transferring electron-density into the empty p-orbital of the silicon center, thus resulting in its electron-richness and stability. The strong donor capacity of the silylene was used to stabilize further reactive silicon species: while treatment with carbon disulfide led to the formation of silylene-CS_2_ complexes, the reaction with N_2_O or CO_2_ was found to depend on the electronic and steric properties of the ylide substituent. Whereas the tosyl system yielded a room-temperature stable silanone, the cyano-substituted silylene formed a carbonate complex with CO_2_ and a dimeric silanone with N_2_O. Additionally, both silylenes facilitated the isolation of silicon compounds with extended π-conjugated units, highlighting the potential of ylide substituents to stabilize unusual bonding situations.

## Abstract 2

The incorporation of ylide substituents into acyclic aminosilylenes leads to enhanced donor abilities, enabling the isolation of a remarkably stable silanone and compounds with extended π-conjugated units.

## Graphical abstract

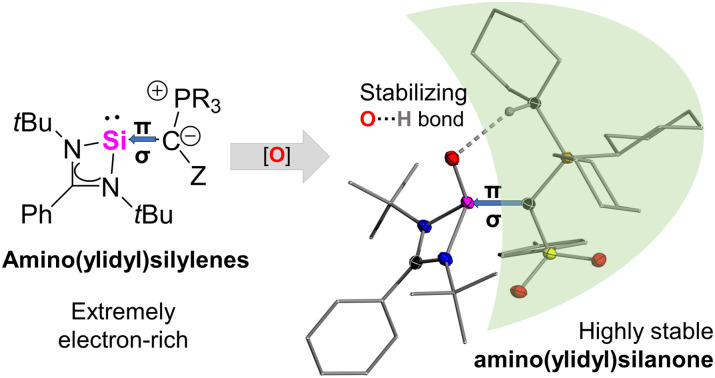

## Introduction

Silylenes, the divalent silicon analogues of carbenes, have garnered significant attention over the past years owing to their role as synthetic intermediates in material chemistry, and their potential use as potent ligands^1^ and catalysts. In general, silylenes are highly reactive, short-lived species which tend to dimerize or polymerize, but can be stabilized through thermodynamic and kinetic control by careful choice of the substitution pattern. Until to date, a diverse array of stable and isolable silylenes with distinct structural motifs and reactivity patterns have been reported.^2^ While cyclic silylenes were the first examples of isolable silylenes,^3^ various acyclic systems first with an increased coordination number at silicon^4^ and more recently also two-coordinated systems have been generated.^5^ Moreover, masked silylenes were established, which liberate a more reactive silylene (*e.g.* from disilenes or siliranes) upon reaction with other substrates.^6^

**Fig. 1:**
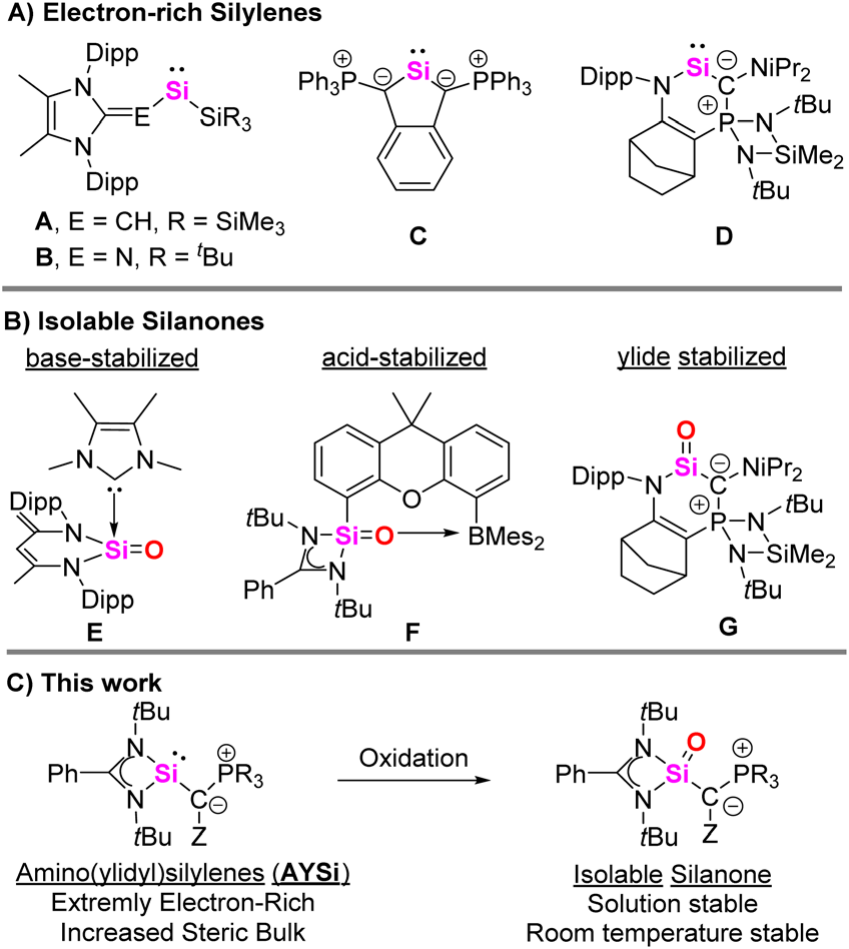
(A) Isolated ylidylsilylenes and their resonance structures, (B) examples for silanones (Dipp = 2,6-di-isopropylphenyl) and (C) amino(ylidyl)silylenes (AYSi) and the corresponding silanone presented in this work.

The control of the properties of the substituent bound to silicon is key to stabilize silylenes and control their reactivity. Therefore, various substituents have been tested, including amino, aryl, silyl, boryl, boryloxo, thiolato or phosphino groups.^5^ To increase the silylene donor strength, *e.g.* for transition metal coordination or for stabilizing other low-valent silicon species, strongly electron-donating substituents are required. Besides alkyl and silyl groups, moieties with (partial) zwitterionic bonding situations have appeared as particularly potent alternatives, such as *N*-heterocyclic olefins (*e.g.*A,^7^Fig. 1) and imines (B)^8^ as pioneered by Rivard and Inoue. Also phosphorus ylides have successfully been employed, as first demonstrated by Driess (C),^9^ and later by Kato and Baceiredo (D). The ylidylsilylenes such as D were shown to be particularly strong donors, similar to *N*-heterocyclic carbenes.^10^

The synthetic potential of highly electron-rich silylenes was demonstrated by means of their application in bond activations or the stabilization of reactive species such as silanones. Silanones are the silicon analogs of ketones, which tend to dimerize due to the weak π-interaction between silicon and oxygen.^11,12^ Many groups have targeted the isolation of stable heavier ketones, with many decisive advances being made in the past two decades. At first, base and acid-base stabilized silanones were reported, such as the systems E,^13,14^ and F,^15^ by Driess and co-workers with bidentate diamino ligands (Fig. 1). A three-coordinate silanone was first isolated by Filippou, using a metallosilylene precursor,^16,17^ followed by organic silanones reported by Iwamoto,^18^ Kato (G)^19^ as well as Inoue and Rieger,^20^ who made use of strong and bulky alkyl or ylide donors. Recently, Aldridge and co-workers were able to isolate a remarkably stable boryl-substituted silanone capable of activating ammonia across the Si–O bond,^21^ and Cui presented a strained cyclic silanone.^22^

Intrigued by their donor strengths, we became interested in accessing ylide-substituted silylenes with an acyclic ylide group. We hypothesized that due to the strong donor capacity of phosphorus ylides, these silylenes should become particularly strong donors and therefore allow for the isolation of stable silanones and other reactive silicon species.

## Results and discussion

### Synthesis of acyclic ylidylsilylenes

**Scheme 1:**
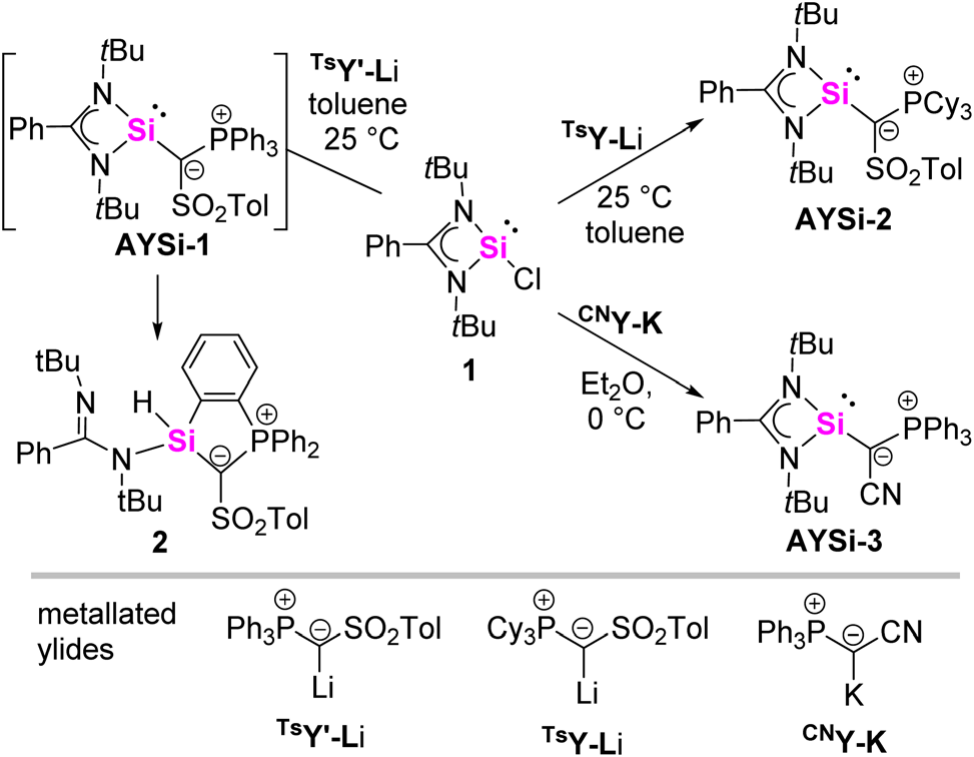
Synthesis of the cyclic compound 2 and the amino(ylidyl)silylenes (AYSI) AYSi-2 and AYSi-3.

We began our studies with targeting an amino(ylidyl)silylene (AYSi). We selected Roesky's benzamidinato chlorosilylene as precursor as this scaffold has been extensively studied in silylene chemistry and thus seemed ideal for comparing the impact of an ylide substituent on the properties of the corresponding silylenes. To access the corresponding AYSis we reacted chlorosilylene 1 with our previously reported metalated ylides ^Ts^Y-Li, ^Ts^Y′-Li and ^CN^Y-K, respectively (Scheme 1).^23,24^ Unfortunately, reaction with the PPh_3_-substituted yldiide ^Ts^Y′-Li led to the clean formation of the cyclic product 2 instead of the targeted AYSi. 2 could be isolated as a yellow solid in a 69% yield and unambiguously characterized by NMR spectroscopy as well as elemental and X-ray diffraction analysis (XRD) (see the ESI† for details). 2 is presumably formed by a C–H activation reaction of one of the phenyl groups in the phosphonium moiety at the silicon center in the transient silylene AYSi-1.

To prevent *ortho*-metalation we changed to the PCy_3_-substituted analog ^Ts^Y-Li, which led to the clean formation of a new species characterized by a signal at 33.2 ppm (*cf.* 10.1 ppm for ^Ts^Y-Li) in the ^31^P{^1^H} NMR spectrum. After work-up, the targeted silylene AYSi-2 could be obtained as room temperature stable, yellow solid in 87% yield. AYSi-2 shows a doublet at 7.73 ppm in the ^29^Si{^1^H} NMR spectrum with a coupling constant of ^2^*J*_SiP_ = 44.7 Hz, confirming the successful formation of the ylide-substituted silylene. The signal falls within the range of other base-stabilized three-coordinated silylenes,^25^ but is significantly upfield-shifted in comparison to the cyclic ylide-substituted silylene reported by Kato and Baceiredo (*cf.* 202.9 ppm; ^2^*J*_SiP_ = 9.2 Hz).^26^ The larger ^2^*J*_SiP_ coupling constant suggests a more efficient orbital overlap and therefore a stronger electron donation from the ylide substituent. This is further corroborated by the more pronounced downfield shift of the signal in the ^31^P{^1^H} NMR spectrum in comparison to the heavier diylidyltetrylenes (19.7 ppm for Y_2_Ge, 18.6 ppm for Y_2_Sn).^27,28^ In the latter, no electron donation of the lone pair of the ylidic carbon atom toward the group 14 element was observed.

In recent years, we could demonstrate that the donor properties of ylide substituents greatly depends on the substituent in the ylide backbone.^29^ To probe the effect of backbone-modification on the silylene properties, we furthermore prepared AYSi-3 featuring a cyano group in the backbone. AYSi-3 was synthesized *via* a similar procedure than AYSi-2 and isolated as a yellow solid in a good yield of 78%. In comparison to AYSi-2, the ^29^Si{^1^H} NMR spectrum is slightly downfield shifted to 23.7 ppm with a larger ^2^*J*_SiP_ coupling constant of 62.3 Hz. It is interesting to note, that in contrast to AYSi-1 the cyano system does not undergo an intramolecular C–H activation of the PPh_3_ group at room temperature, suggesting distinct differences in the silylene properties due to backbone variation. However, when a C_6_D_6_ solution of AYSi-3 was heated to 70 °C for 4 days, the silylene selectively converted into the C–H activation product analogous to 2 (see ESI† for details).

**Fig. 2:**
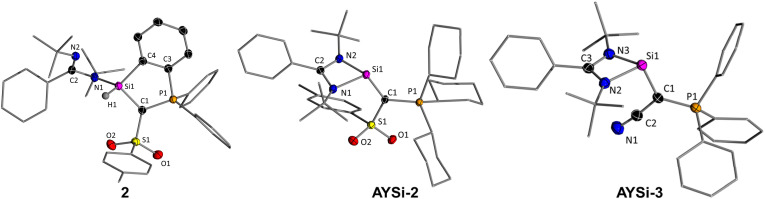
Crystal structure of 2, AYSi-2, and AYSi-3 with thermal ellipsoids drawn at 50% probability level. Hydrogen atoms omitted for clarity. Important bond lengths [Å] and angles [°] are given in Table 1, crystallographic details in the ESI.†

To unambiguously confirm the formation of the ylide-substituted silylenes, single crystals of AYSi-2 and AYSi-3 were grown by vapor diffusion of pentane into a saturated benzene solution or by storage of a saturated diethyl ether solution at −30 °C, respectively (Fig. 2 and Table 1).^30^ In the molecular structure, AYSi-2 exhibits a trigonal pyramidal geometry around the silicon atom with nearly identical Si–C (1.888(1) Å) and Si–N bond lengths (1.872(1) Å and 1.897(1) Å). The Si–C bond is longer compared to the ylidylsilylene D by Kato and Baceiredo (*cf.* 1.773(2) Å) due to the additional coordination of the benzamidinato ligand.^10^ However, it is shorter than in aryl substituted amidinato silylenes (1.926–1.967 Å), indicating additional π-donation from the ylide to the silicon center (see below).^31^ Compared to the metalated ylide (127.4(2)°), AYSi-2 exhibits a more acute P–C1–S angle of 113.32(8)°, presumably due to steric repulsion between the tosyl group and the amidinato ligand. Furthermore, the C1–P and C1–S bonds of 1.7468(14) Å and 1.7047(14) Å are considerably lengthened relative to the metalated ylide ^Ts^Y-Li. This can be attributed to the transfer of electron density from the yldiide ligand to the silicon center, resulting in a lower partial charge at the ylidic carbon atom C1 and reduced electrostatic attraction within the P–C1–S linkage. This corroborates well with the short Si–C bond and a partial double bond character.

**Table 1:** Important bond lengths [Å] and angles [°] of the isolated AYSi in comparison to the metalated ylides ^Ts^Y-Li and ^CN^Y-K

	AYSi-1^a^	AYSi-2	AYSi-3	TsY-Li^b^	^CN^Y-K^c^
P–C1	1.783	1.747(2)	1.702(3)	1.676(2)	1.650(2)
S/C2–C1	1.725	1.704(2)	1.411(3)	1.662(3)	1.377(3)
Si–N	1.895	1.897(1)	1.890(2)	—	—
	1.193	1.872(1)	1.877(3)		
Si–C1	1.878	1.888(2)	1.890(3)	—	—
P–C1–S/C2	110.3	113.32(8)	121.9(2)	120.9(2)	127.6(2)
P–C1–Si	118.5	119.79(8)	121.09(14)	—	—
Si–C1–S/C2	130.2	125.67(8)	116.58(19)	—	—

a Values refer to the energy-optimized structure of AYSi-1.

b Values taken from ref. 24.

c Values correspond to the 18-crown-6 complex reported in ref. 24b.

The structural parameters of the cyano-substituted AYSi-3 are similar to those of AYSi-2 indicating a similar electronic structure at the silylene center. However, due to the smaller steric hindrance of the cyano group in AYSi-3, the Si–C1–P angle widens to 121.9(2)° (*cf.* 113.32(8)° in AYSi-2). This might explain, why AYSi-3 can be isolated whereas it’s tosyl analog AYSi-1 undergoes C–H activation of the PPh_3_ phenyl group. The calculated P–C1–Si angle in the latter amounts to only 110.3°, thus bringing the C–H group into proximity to the reactive silicon center and facilitating the activation process.

### Bonding analysis and ligand properties

**Fig. 3:**
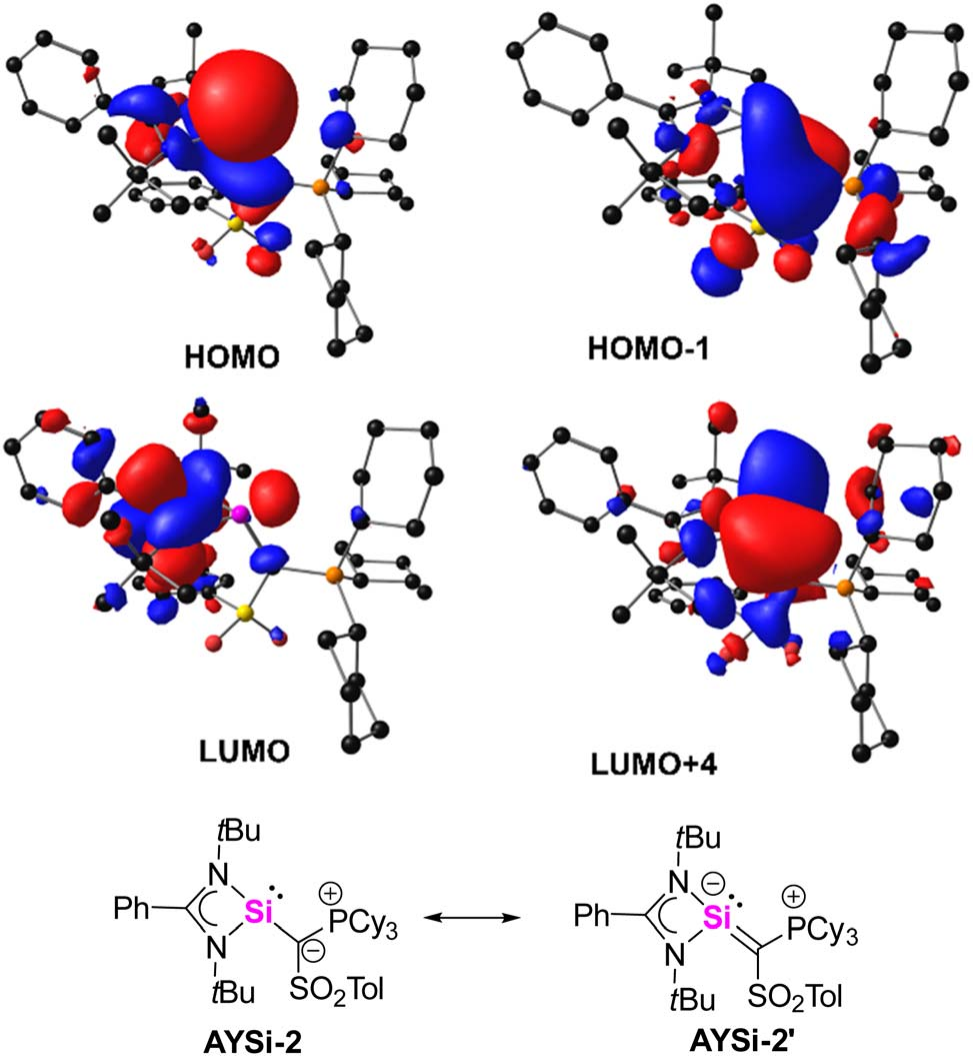
Selected molecular orbitals of the AYSi-2 computed at the PBE0/def2tzvpp level of theory (top). Possible resonance structures of AYSi-2 (bottom).

To gain further insights into the bonding situation of the two AYSis, density functional theory (DFT)^32^ calculations were conducted on the PBE0/def2tzvpp level of theory.^33^ The energy optimized structures well reproduced the solid state structures allowing for an analysis of the Kohn–Sham and natural bond orbitals (NBO).^34^ The highest occupied molecular orbital (HOMO) of AYSi-2 represents the lone pair at the silicon atom, while the HOMO-1 reflects the π-interaction between the ylidic carbon atom and the empty p-orbital at the silicon center (Fig. 3). This contrasts the observations made for germylenes and stannylenes derived from the same metalated ylide, where no π-donation from the ylide substituent to the low-valent tetrel center was detected but corroborates with the short Si–C bonds observed in the crystal structures. In AYSi-2, this interaction is even stronger than the π-donation from the nitrogen atoms of the benzamidinato ligand. Second order perturbation theory indicates that the donation of the lone pair on the ylidic carbon into the empty orbital at silicon is stronger by 4.7 kcal mol^−1^. Due to the base-stabilization by the benzamidinato ligand as well as the π-donation of the ylidic ligand, the formally empty p-orbital at the silicon center is high in energy and is represented by LUMO+4. Overall, these observations suggest that AYSi-2 can be described by two resonance structures, silylene AYSi-2 and the silyl anion form AYSi-2′ (Fig. 3 bottom). However, the Wiberg bond indices (WBI) of the Si–C bonds in AYSi-2 as well as in AYSi-3 are both small (0.76 and 0.69, respectively), indicating that the silylene form is still dominant. Importantly, the WBI for the cyano system is slightly smaller due the delocalization of the π-density into the cyano group, reducing the donation toward silicon.

**Scheme 2:**
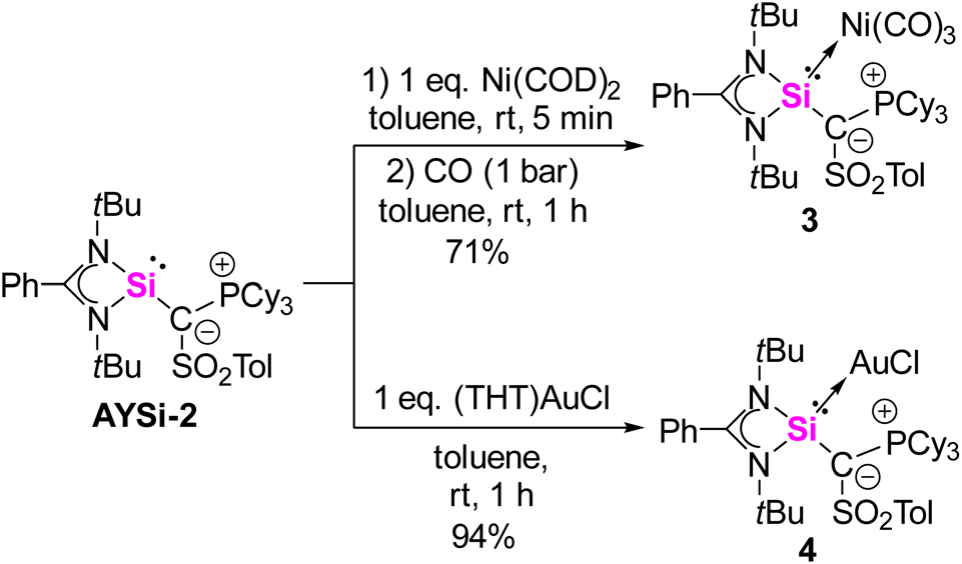
Synthesis of nickel complex 3 and gold complex 4 from the isolated silylene AYSi-2 (COD = cyclooctadiene, THT = tetrahydrothiophene).

**Fig. 4:**
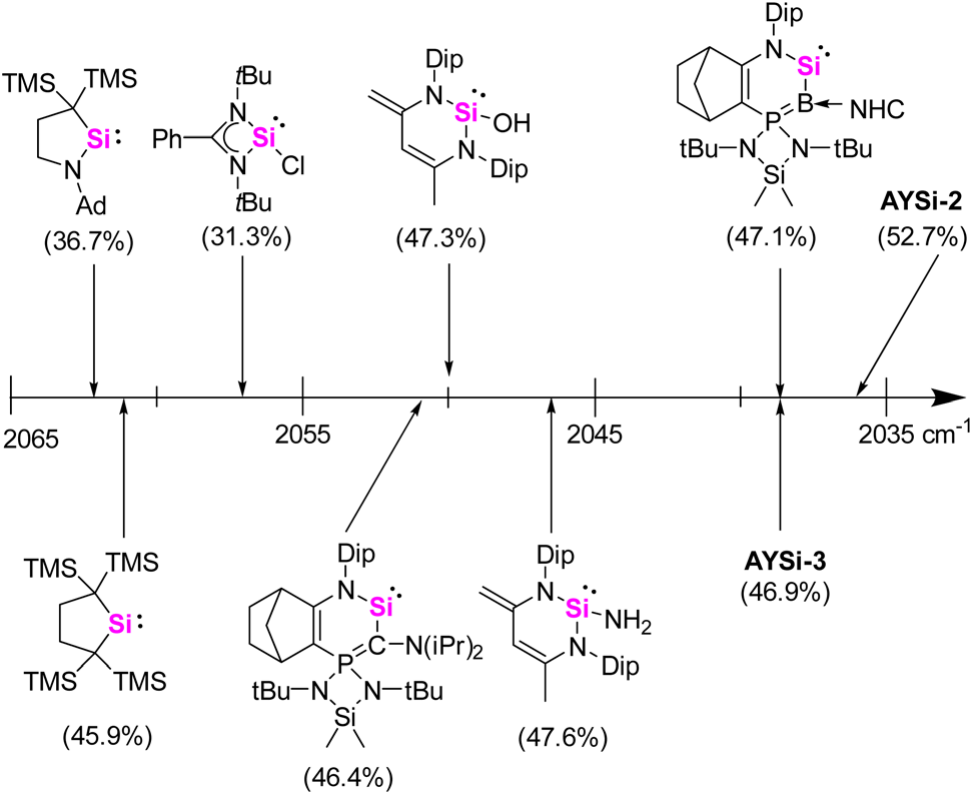
Comparison of the TEP values for various silylenes determined by asymmetric CO stretching frequency of LNi(CO)_3_ in toluene at room temperature.^26,31^ % *V*_bur_ is given in parentheses.

A widely applied experimental method for the quantification of the electron-donating properties of ligands is the Tolman Electronic Parameter (TEP) which measures the CO stretching frequency in l-Ni(CO)_3_ complexes.^35^ The corresponding silylene nickel complexes were synthesized by the reaction of one equivalent of the AYSi ligands with Ni(COD)_2_ (COD = 1,5-cylcooctadiene) at 0 °C and subsequent ligand exchange with CO. Complex 3 could be isolated as a colorless solid in a good yield of 71% (Scheme 2, see the ESI† for details). The IR spectra for the Ni complexes of AYSi-2 and AYSi-3 in toluene showed characteristic bands for the CO vibration at 2036 and 2038 cm^−1^, respectively. Both values are extremely red-shifted, confirming the high donor strength of the two AYSis, with AYSi-2 being the stronger donor (Fig. 4). The slightly lower donor ability of AYSi-3 can be attributed to the partial delocalization of π-density into the cyano group. A comparison of the IR vibration of the nickel carbonyl complexes of AYSi-2 and AYSi-3 with those of other silylenes reported in literature shows that silylenes with a di- and tri-coordinated silicon center typically give TEP values ranging between 2045 cm^−1^ and 2076 cm^−1^.^36^ The only other silylene that comes close to the value of AYSi-2 is the boraylide-substituted silylene by Kato and Baceiredo, whereas the analogous AYSi C only showed a TEP value of 2051 cm^−1^.^26^

**Fig. 5:**
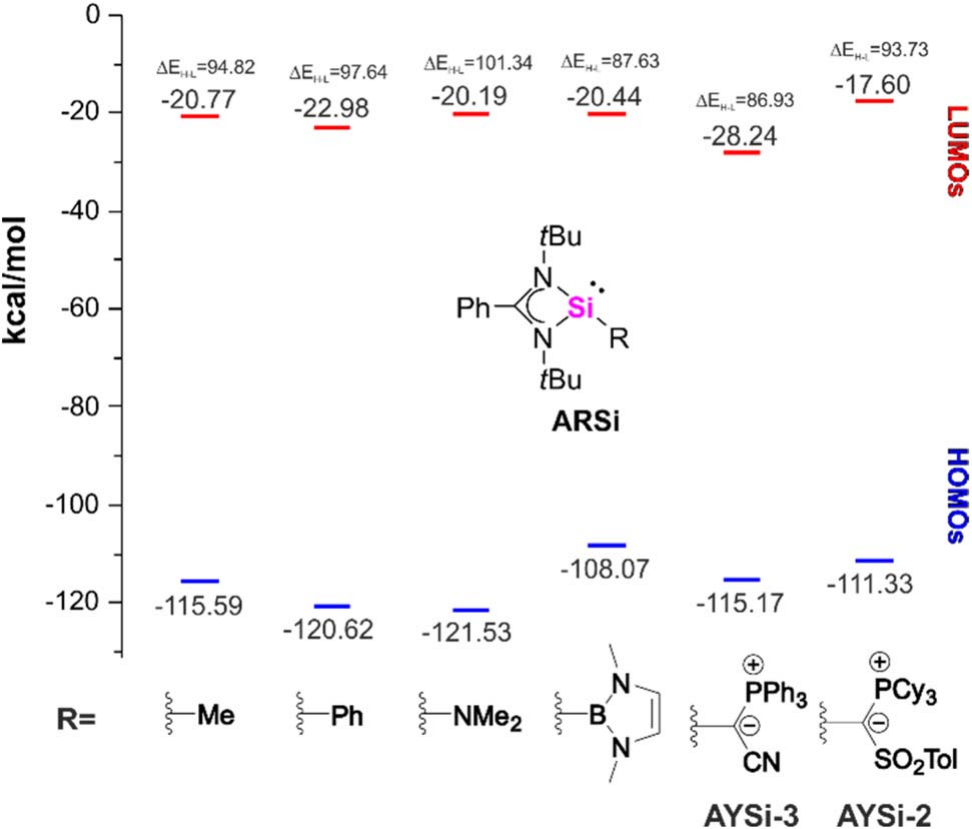
Calculated HOMO–LUMO energies of amidine-supported silylenes ARSi with different R substituents.

To fully disentangle the electronic influence of the ylide-substituent we analyzed the HOMO–LUMO energies of a series of amidine-supported silylenes ARSi with varying substituents at the silicon center (Fig. 5, see ESI† for details). The calculated orbital energies demonstrate significant differences between the two ylide substituents. Owing to the strongly electron-withdrawing cyano group,^27*b*^AYSi-3 features a low-lying HOMO in the range of a simple methyl substituent and a low-lying LUMO, resulting in a relatively small gap of Δ*E*_H–L_ of 86.9 kcal·mol^−1^. In contrast, Δ*E*_H–L_ of AYSi-2 is larger with 93.7 kcal·mol^−1^, with significantly higher lying LUMO (−17.6 kcal·mol^−1^) and HOMO energies (−111.3 kcal·mol^−1^), respectively. The latter is energetically comparable to NHB substituted ARSi (−108.1 kcal·mol^−1^). This observation supports the high nucleophilicity of silylene AYSi-2 as indicated by the TEP values, which is only surpassed by tetracoordinated silylenes, such as Tacke's bis(amidinato) system (*E*_HOMO_ = −100.4 kcal·mol^−1^).^38^

To also quantify the steric properties of the AYSi, we initially reacted AYSi-2 with one equivalent of (THT)AuCl (THT = tetrahydrothiophene) to obtain the corresponding gold complex 4 as a colourless solid in 94% yield (Scheme 2). Unfortunately, no crystal structure could be obtained so we opted to calculate the buried volume (% *V*_bur_) from the energy optimized structure obtained from DFT calculations, yielding a value of 52.7%. To better rank this value, we determined the % *V*_bur_ for some literature known silylenes by the same method (Fig. 4). Within that series, AYSi-2 shows the highest steric demand, caused by the bulky tricyclohexylphosphonium group pointing towards the gold center. In comparison, Roesky's chlorosilylene only shows a % *V*_bur_ of 31.3%. The cyano system is likewise sterically demanding but exhibits a lower % *V*_bur_ value of 46.9%, which is close to the value of Kato's boraylide-substituted silylene.

### Exploiting the donor strength of amino(ylidyl)silylenes

**Fig. 6:**
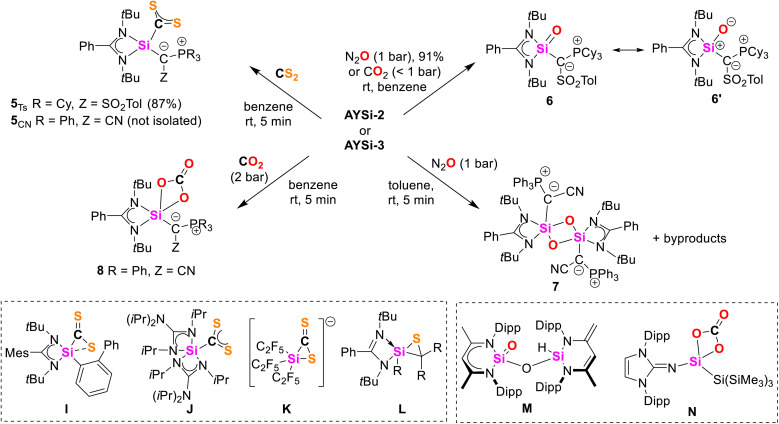
(top) Reactivity studies of silylenes AYSi-2 and AYSi-3: synthesis of the AYSi-CS_2_ adducts 5_Ts_ and 5_CN_; oxidation of AYSi-2 under an atmosphere of N_2_O (or CO_2_) to silanone 6, reaction of AYSi-3 with N_2_O to siloxane 7 and with CO_2_ to carbonate complex 8. Bottom left: reported silylenes-CS_2_ adducts by Driess (I) and Tacke (J), and examples of anionic (K) and neutral (L) thiasiliranes by Hoge, and Roesky and Stalke. Bottom right: reported silanone M by Driess and carbonate N by Inoue.

The extremely electron-donating and sterically demanding nature of silylenes AYSi-2 and AYSi-3 suggests a strong potential for forming complexes with small molecules and stabilizing Si=E multiple bonds. At first, we explored their potential in adduct formation with more challenging electrophiles such as CS_2_ or CO_2_. Upon addition of CS_2_ to a benzene solution of AYSi-2, the color immediately turned red, resulting in the precipitation of the CS_2_ adduct 5_Ts_, which could be isolated as a red solid with a good yield of 87% (Fig. 6). The ^29^Si{^1^H} NMR spectrum showed an upfield shifted signal at −39.3 ppm, indicating the successful formation of the AYSi-CS_2_ adduct. This was further confirmed by the extremely deshielded signal for the carbon atom of the CS_2_ moiety resonating at 273.3 ppm in the ^13^C{^1^H} NMR spectrum, compared to 192.7 ppm for free CS_2_. These shifts are in accordance with observations made for silylene-CS_2_ adducts I,^37^ and J,^38^ albeit the upfield shift in the ^29^Si{^1^H} spectrum is less pronounced for 5_Ts_. This can presumably be attributed to the coordination number of the silicon center. While all previously reported CS_2_ adducts featured a penta-coordinated silicon center, it is only tetra-coordinated in the AYSi-CS_2_ adducts as confirmed by XRD analysis. The Si–S distances amount to 3.0280(7) Å and 3.0310(7) Å and are thus significantly longer than the Si–S bonds found in neutral (L, 2.09–2.21 Å)^39^ or anionic thiasiliranes (K, 2.660 Å).^40^ Furthermore, the Si1–C42 bond length of 1.9074(18) Å is longer than the one found in I (1.855 Å) and J (1.865 Å) corroborating with the description of 5_Ts_ as a CS_2_ adduct. As a consequence of the CS_2_ coordination, the Si1–C1, Si1–N1 and Si–N2 bonds shorten (1.839(2) Å, 1.819(2) Å and 1.833(2) Å), reflecting the electron transfer from the Lewis basic silicon center to CS_2_ and from the ylide (and amidinato) ligand to silicon.

AYSi-3 demonstrated the same reactivity towards CS_2_ than AYSi-2. Upon addition of CS_2_, a similar color change from yellow to red was observed and the ^31^P{^1^H} NMR spectrum showed the selective formation of a new compound, which we could identify as the CS_2_ complex 5_CN_. However, despite its selective formation, compound 5_CN_ could not be isolated as it decomposed upon removal of the solvent to a complex product mixture. This underscores the beneficial properties of the more electron-rich AYSi-2 for isolating reactive species. Repeating the experiment with a slight excess of CS_2_, allowed a full NMR spectroscopic characterization and crystal structure analysis, confirming the successful formation of the AYSi-CS_2_ adduct 5_CN_ (see the ESI† for details).

**Fig. 7:**
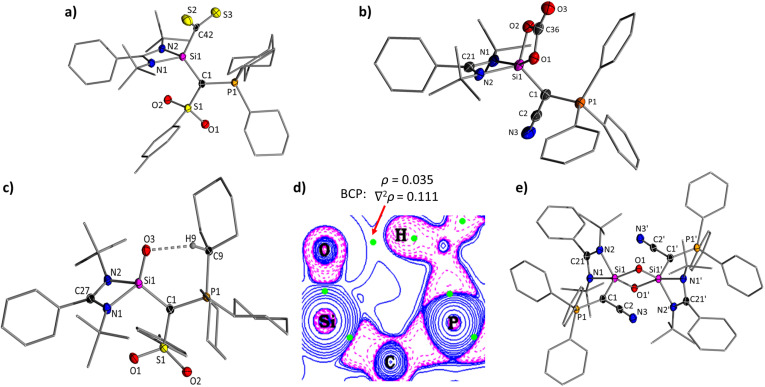
(top) Crystal structures of (a) CS_2_ complex 5_Ts_ and (b) carbonate 8 and (bottom) structure of (c) silanone 6 and (e) siloxane 7. (d) Contour plot of the Laplacian of the electron density in 6 highlighting bond critical points (BCP) in green. Thermal ellipsoids are drawn at the 50% probability level and all hydrogen atoms except H9 were omitted for clarity. Important bond lengths [Å] and [°]: 5_Ts_: Si1–C1 1.8391(18), Si1–C42 1.9074(18), S2–C42 1.6468(19), S3–C42 1.6733(18), Si1–S2 3.0280(7), Si1–S3 3.0310(7), S1–C36–S2 127.83(11). 8: Si1–C1 1.8161(13), Si1–O1 1.8011(10), Si1–O2 1.7271(10), C36–O1 1.3361(18), C36–O2 1.3673(17), C36–O3 1.1965(18), P1–C1–Si1 124.16(8). 6. C1–Si1 1.8550(13), Si1–O3 1.5455(9), C1–P1 1.7631(12), O3–H9 2.157(16), P1–C1–S1 119.85(7), Si1–C1–P1 120.34(7), Si1–C1–S1 119.07(7). 7: C1–Si 1 1.8568(15), C1–P1 1.7113(14), Si1–O1 1.6708, Si1–O1′ 1.7355(10), Si1–N1 2.0021(12), Si1–N2 1.8443(13), Si1–O1–Si1′ 95.43(5), O1–Si1–O1′ 84.57(5), Si1–C1–C2 114.73(10), Si1–C1–P1 137.60(8), P1–C1–C2 107.65(10).

Motivated by these results, we attempted the isolation of the corresponding CO_2_ complexes. However, addition of carbon dioxide to a toluene solution of AYSi-2 led to liberation of carbon monoxide and the formation of the stable silanone 6. Silanone 6 was also readily accessible from the reaction of solid AYSi-2 with an atmosphere of N_2_O, leading to a discoloration of the yellow solid overnight. The ^29^Si{^1^H} NMR spectrum of 6 features a signal at −40.6 ppm with a coupling constant of ^2^*J*_SiP_ = 19.3 Hz, in line with the successful oxidation of the silylene center. In the molecular structure (Fig. 7), silanone 6 exhibits a Si–O bond of 1.5455(9) Å, which is shorter than typical Si–O single bonds (1.63 Å),^41^ but on the longer end of reported values for Si=O double bonds.^16,17,20,42^ For example, Kato's silanone G featured a Si=O bond length of 1.533(1) Å, Aldridge's boryl-substituted amidinato-silanone of 1.5406(9) and 1.5384(9) Å,^19*a*,21^ and Iwamoto's tricoordinate silanone a Si–O distance of only 1.518 (2) Å.^18^ This clearly argues for a strong polarization of the Si–O bond in 6, which presumably contributes to its high stability even toward heating (see below) or the reaction with another equiv. CO_2_ to form the corresponding carbonate, as has been reported for other silanones (*e.g.*N).^42*a*,43–45^ Silanone formation by reaction with CO_2_ has less often been observed (*e.g.* for the formation of M).^46,47^ The oxidation of AYSi-2 results in a further shortening of the Si–C and Si–N bond lengths, while the respective bond angles widen to accommodate the oxygen substituent.

Interestingly, a short intramolecular contact of 2.157(16) Å is found between the oxygen and the hydrogen of the α-carbon of the cyclohexyl group. This hydrogen bond is even present in solution as evident by the downfield shift of the CH signal to 3.54–3.38 ppm in the ^1^H NMR spectrum (relative to 2.27–2.09 ppm in ^Ts^Y-H and to 1.95–1.84 in ^Ts^Y-Li). The hydrogen bonding interaction is also supported by Quantum Theory of Atoms in Molecules (QTAIM) calculations and by Non-Covalent Interactions (NCI) analysis, yielding a bond critical point between the oxygen and the hydrogen atom as well as an attractive interaction (Fig. 7, see ESI† for details). Additionally, second-order perturbation theory calculations show three LP(O)∙∙∙ σ*(H–C) interactions ranging from 2.5–6.8 kcal·mol^−1^ (See ESI†).

In line with the long Si–O bond, the calculations revealed a strongly polarized Si–O double bond. The WBI of the Si–O bond of 1.01 is smaller than the corresponding values calculated for parent dimethylsilanone (1.37) and the cyclic silanone G (1.14) by Kato,^19*a*^ as well as other amidine-supported silanones ARSi=O (see the ESI† for details). Natural Population Analysis (NPA) yielded strongly opposing charges at Si (*q*_Si_ = 2.21 e) and O (*q*_O_ = −1.29 e). Nonetheless, silanone 6 revealed to be highly stable towards dimerization or oligomerization. No decomposition was observed in THF solution over the course of two months at room temperature (see ESI†). Furthermore, the silanone is indefinitely stable at 80 °C in THF solution. Thus, it ranks amongst the most stable silanones reported to date.^17*b*,18,22^

Given the high stability of 6, we next focused on the cyano system AYSi-3. Within minutes, the ^31^P{^1^H} NMR spectrum of a toluene solution of AYSi-3 exposed to an atmosphere of N_2_O revealed the appearance of a new species at 28.9 ppm. This species could not be isolated due to decomposition during the work-up process. However, single crystals were obtained by crystallization from the reaction mixture, revealing the formation of the dimeric siloxane 7 (Fig. 7). The crystal structure of 7 exhibits an asymmetrical bonding within the planar Si–O–Si–O ring, characterized by one shorter Si–O bond (1.671(1) Å) and one longer Si–O bond (1.736(1) Å). Both bond distances are clearly longer than the Si=O bond in silanone 6, consistent with typical single bonds.^48^

To understand the instability of the corresponding monomeric silanone of 7 we performed additional computational studies (see ESI† for details). Bonding analysis revealed negligible differences in the electronic structure between the hypothetic monomeric 7 and 6. For instance, the Wiberg bond index of the Si–O bond of 1.09 closely resembles the value of 6 (1.01). This suggests that the reduced stability of monomeric 7 relative to 6 is not due to electronic effects but rather the reduced steric bulk of the cyano-substituted ylide compared to its tosyl substituted counterpart. The preferential formation of the dimer is also further supported by the energetic preference of the dimer 7, which was found to be favored by 32.5 kcal mol^−1^ over the monomeric silanone. In contrast, dimerization of 6 is disfavored by 11.5 kcal mol^−1^.

To verify the presence of the monomeric silanone of 7, we attempted to trap it by addition of CO_2_. The formation of carbonate complexes has been widely reported in the reaction of silylenes with CO_2_, involving the intermediate generation of a transient silanone species.^42–45^ Indeed, exposure of AYSi-3 to an atmosphere of CO_2_ led to decolorization of the toluene solution after stirring for 1 day at RT and the precipitation of a colorless solid, which could be isolated in 39% yield. XRD analysis confirmed the formation of carbonate complex 8, supporting the intermediate presence of the silanone. In 8, the carbonate ligand is chelating the silicon centre *via* two oxygen atoms with Si–O bond lengths of 1.727(1) and 1.801(1) Å. In the ^13^C-NMR spectrum in C_6_D_6,_ the carbonyl carbon appears at 153.1 ppm and the IR spectrum displays a band at 1785 cm^−1^, which falls within the expected range for C=O stretching frequencies. The formation of carbonate complex 8 contrasts the reaction of AYSi-2 which forms the stable silanone 6 under the same conditions.

**Fig. 8:**
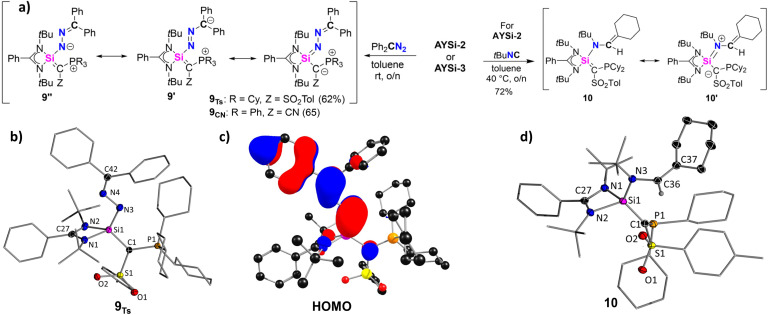
(a) Reactivity of the AYSi2 and AYSi3 with diphenyldiazomethane to obtain 9_Ts_ and 9_CN_ and reaction of AYSi-2 with *t*BuNC yielding 9. (b) Solid-state structure of 9_Ts_. Thermal ellipsoids are shown at the 50% probability level and hydrogen atoms have been omitted for clarity. Important bond lengths [Å] and angles [°]: Si1–C1 1.8437(15), Si1–N3 1.6765(13), N3–N4 1.3848(17), N4–C42 1.2965(19), Si1–N1 1.8370(13), Si1–N2 1.8469(13), C1–Si1–N3 112.66(6), Si1–N3–N4 108.07(9), N3–N4–C42 117.94(12). (c) HOMO of 9 and (d) crystal structure of 10. Important bond lengths [Å] and angles [°]: Si1–C1 1.8044(13), Si1–N3 1.7230(11), Si1–N1 1.8193(13), Si1–N2 1.8803(11), C1–P1 1.8052(13), N3–C36 1.4502(16), C36–C37 1.3391(19).

As AYSi-2 forms a stable silanone, we next explored whether further compounds with Si=E multiple bonds can be accessed. Initially, we targeted the formation of a silene through reaction with a diazomethane and subsequent extrusion of N_2_. However, the reaction of AYSi-2 with 1,1-diphenyl diazomethane led to no gas evolution. NMR spectroscopy revealed the formation of a new species featuring a signal at 31.5 ppm in the ^31^P{^1^H} NMR spectrum and at −16.5 ppm in the ^29^Si{^1^H} NMR spectrum. This species was identified as silazine 9_Ts_ (Fig. 8), which could be isolated as a yellow solid in a good yield of 62%. AYSi-3 showed a similar reactivity furnishing 9_CN_ in 65% yield. XRD analyses of both compounds unambiguously confirmed the structure of the silazine with a conjugated Si–N–N–C linkage. A similar motif was reported by Filippou starting from an NHC-stabilized chlorosilylenes.^49^ The newly formed Si1–N3 bond in 9_Ts_ of 1.677(1) Å is slightly elongated compared to Filippou's silazine (*cf.* 1.655 Å), but considerably shorter than in a dimeric complex reported by Roesky (*cf.* 1.735 Å),^50^ in which a diazo compound was coordinated by two silicon centers to form a Si–N–Si–N four-membered ring. The N3–N4 and the N4–C42 bond lengths of 1.3848(17) Å and 1.2965(19) Å are in agreement with partial double bond character and suggest π-delocalization within the entire C–Si–N–N–C linkage as expressed by the different resonance structures 9, 9′ and 9′′. This is further supported by the planarity of this linkage and the delocalized nature of the HOMO of 9_Ts_. NBO analysis revealed opposing NPA charges at the Si (*q* = 2.19 e) and the N3 (*q* = −1.02 e) atom, as well as high negative charge at the C1 carbon atom, suggesting a dominance of the structures 9 and 9′′ (see ESI† for further details).

As a further attempt to construct a Si–C double bond starting from AYSi-2, we next reacted the silylene with *tert*-butyl isocyanide, *t*BuNC. Other silylenes have reported to form silaketenimines, R_2_Si=C=NR, upon reaction with isocyanides.^51,5*e*^ However, AYSi-2 reacted with *t*BuNC to the rearranged compound 10, which was isolated as a colorless solid in a good yield of 72%, and its structure confirmed by XRD analysis (Fig. 8). In compound 10, a bond between the silicon center and the nitrogen atom of the former isocyanide is formed, accompanied by the transfer of one cyclohexyl group of the phosphonium moiety and its C–H bond activation at the former isocyanide carbon atom. The reduction of the phosphonium to the phosphine group is evidenced by a significant upfield shift from 33.2 to −13.9 ppm in the ^31^P{^1^H} NMR spectrum. Furthermore, additional signals in the aliphatic region in the ^13^C{^1^H} NMR spectrum reflect the newly formed cyclohexene moiety. Notably, AYSi-3 showed no reactivity towards *t*BuNC even at elevated temperatures. In the molecular structure of 10, the Si1–N3 bond amounts to 1.723(1) Å, which is significantly shorter than the Si–N bonds to the amidinato ligand, but longer than the Si–N bond in 9. In contrast, the former N3–C36 multiple bond in the isocyanide is increased to 1.4502(16) Å, while the C36–C37 distance (1.3391(13) Å) is clearly in the rage of a double bond. The Si–C1 bond to the ylide ligand (1.804(1) Å) is the shortest among all reported compounds based on ^Ts^Y. These bond lengths clearly suggest the preference of the silaalkene form 10 with a Si=C double bond over the zwitterionic silaiminium form 10′.

Overall, the reported reactivities highlight the electronic flexibility of the ylide ligands in the AYSis. These ligands act as strong yet flexible electron reservoirs, facilitating the transfer of electron density as required by the silicon center and thereby stabilizing compounds with unique bonding situations.

## Conclusions

In conclusion, we reported on the isolation of amino(ylidyl)silylenes through salt elimination using metalated ylides together with Roesky's benzamidinato chlorosilylene. The stability of the AYSis were found to strongly depend on the steric and electronic properties of the ylidic ligand. Two AYSis with a tosyl (AYSi-2) and a cyano-substituted (AYSi-3) ylide could be isolated in excellent yields. These AYSi's were found to be extremely electron-rich, featuring TEP values of 2036 and 2038 cm^−1^, respectively, which are the lowest values reported for silylenes to date. This can largely be attributed to the electron-donation of the lone pair on the ylidic carbon into the p-orbital of the silicon center. NBO analysis, however, revealed that the AYSis are still best represented by a silylene rather than a silyl anion with a C–Si double bond. The electron-rich nature and the high steric demand of AYSis, allowed for the isolation of the silylene-CS_2_ adducts 5_TS_ and 5_CN_, which featured a tetracoordinate silicon center with no further interaction with the sulfur atoms. In contrast, treatment of AYSi-2 with carbon dioxide or N_2_O led to the formation of the remarkably stable silanone 6, which showed no sign of decomposition even in refluxing THF solution over several weeks. This extraordinary stability of silanone 6 is attributed to steric shielding and the presence of an intramolecular hydrogen bond. In AYSi-3, these stabilizing factors are missing, leading to the formation of a reactive silanone that dimerizes or directly reacts with CO_2_ to the corresponding carbonate complex. The unique properties of the AYSis were furthermore exploited to form silazines with a conjugated C–Si–N–N–C linkage through the reaction with diphenyl diazomethane and a unique silene through reaction with *tert*-butylisocycanide and the transfer of a cyclohexyl group from the PCy_3_ moiety in the ylide.

Overall, this study showcases the potential of ylide substituents to effectively stabilize highly reactive silicon compounds without compromising their reactivity. By adjusting the electronic and steric properties of the ylide substituents, we can tune the properties of the target compounds, ultimately leading to the isolation of an exceptionally stable silanone and a series of silicon compounds with extended π-conjugated units. We are currently further exploiting the potential of ylide substituents to stabilize other reactive main group species.

## Data availability

The data that support the findings of this study are available in the ESI.† This includes experimental procedures, NMR and IR spectra as well as crystallographic and computational details.

## Author contributions

V. H. G. designed and oversaw the project. F. K. planned the study, carried out most of the synthetic work, analyzed the spectroscopic data and performed computational studies on the bonding situation of the AYSis, 6 and 10. D. K. performed the majority of the computational studies. S. M. assisted in the synthesis and data analysis of the AYSis, L. H. assisted in the synthesis of compounds 6–10. H. D. performed initial experiments towards the synthesis of ylidylsilylenes. The manuscript was written by F. K. and V. H. G with the help of L. H. and D. K.

## Conflicts of interest

There are no conflicts to declare.

## Supplementary Material

SC-016-D5SC01812A-s001

SC-016-D5SC01812A-s002
