# Supplementary material for: Base-stabilized acyclic amino(ylidyl)silylenes: electron-rich donors for the stabilization of silicon-element multiple bonds
Source: Chem Sci. 2025 Apr 3;16(19):8346–56. doi: 10.1039/d5sc01812a (PMC11980799; doi:10.1039/d5sc01812a)
Supplement: SC-016-D5SC01812A-s001 [file SC-016-D5SC01812A-s001.pdf]

Supplementary Information (SI) for Chemical Science.
This journal is © The Royal Society of Chemistry 2025

Base-Stabilized Acyclic Amino(ylidyl)silylenes: Electron-Rich Donors for the Stabilization of Silicon-Element Multiple Bonds

Felix Krischer, Stephan Mayer,‡ Lennart Hensle, ‡ Daniel Knyszek, Heidar Darmandeh and Viktoria H. Gessner*

Faculty of Chemistry and Biochemistry, Inorganic Chemistry II, Ruhr-University Bochum, Universitätsstr. 150, 44801 Bochum (Germany)
E-mail: viktoria.gessner@rub.de

Table of Contents

1 General Experimental Details **2**
2 Experimental Procedures **3**
2.1 Synthesis of 2 3
2.2 Synthesis of AYSi-2 3
2.3 Synthesis of AYSi-3 4
2.4 Synthesis of AYSi-2 nickel complex 3 4
2.5 Synthesis of AYSi-2 gold complex 4 5
2.6 Synthesis of AYSi-2-CS_2 adduct 5_{Ts} 5
2.7 Synthesis of AYSi-3-CS_2 adduct 5_{CN} 6
2.8 Synthesis of Silanone 6 6
2.9 Synthesis of Siloxane 7 7
2.10 Synthesis of AYSi-2 carbonate complex 8 7
2.10 Synthesis of Silazine 9_{Ts} 8
2.11 Synthesis of Silazine 9_{CN} 9
2.12 Synthesis of 10 9
3 Stability test monitored by NMR spectroscopy **11**
3.1 Stability of silylene AYSi-2 in toluene 11
3.2 stability of silylene AYSi-3 in C_6D_6 14
3.3 Stability of silanone 6 in THF 16
4 Reactivity of silanone 6 towards CO_2 ..... **20**
5 NMR and IR Spectra of the Isolated Compounds **22**
6 Crystal Structure Analyses **50**
7 Computational Details **87**
7.1 General remarks 87
7.2 Hydrogen Bonding in Silanone 6 87
7.3 Results of the NBO analysis 88
7.4 Coordinates of the optimized structures 91
8. References **147**

1 General Experimental Details

All experiments were carried out under a dry, oxygen-free argon atmosphere using standard Schlenk techniques. Involved solvents were dried using an MBraun SPS-800 (THF, DCM, toluene, acetonitrile, diethylether, hexane and pentane). Deuterated solvents were degassed and stored over molecular sieves in an argon-filled glovebox. **^{Ts}Y'-Na**,[27a] **^{Ts}Y-Li**,[22] **^{CN}Y-K**,[27b] and Roesky's chlorosilylene **1**[26b] were prepared according to literature procedures. All other reagents were purchased from Sigma-Aldrich, ABCR, Rockwood Lithium or Acros Organics and used without further purification.

NMR spectra were recorded on Avance-400 spectrometers at 25 °C if not stated otherwise. All values of the chemical shift are in ppm regarding the δ-scale. All spin-spin coupling constants (*J*) are printed in Hertz (Hz). To display multiplicities and signal forms correctly the following abbreviations were used: s = singlet, d = doublet, t = triplet m = multiplet, dd = doublet of doublet, br = broad signal. Signal assignment was supported by APT, HSQC (^{1}H / ^{13}C) and HMBC (^{1}H / ^{13}C) correlation experiments.

IR-Spectra were recorded in an argon filled glovebox on a Shimadzu IRSpirit with QATR-S module and in transmission mode with a Specac "Omni-cell" with KBr plates and a 0.1 mm spacer. Measurement and processing details for individual spectra can be extracted from the corresponding tables in the supporting information.

Elemental analyses were performed on an Elementar vario MICRO-cube elemental analyzer.

HRMS-ESI: An LTQ Orbitrap Velos (Thermo Fisher Scientific, Bremen, Germany) was used for direct infusion via a syringe pump. The heated desolvation capillary was set to 200°C and a spray voltage of 1.8 kV was supplied. In the tune file the LTQ Orbitrap was set to the following parameters (R = 30,000; IT = 500 ms; AGC Target = 1,000,000).
HRMS-LIFDI: A JEOL AccuTof GCv (JMS-T100GCV) (JEOL, Tokyo, Japan) was equipped with a LIFDI source from Linden (CMS, Weyhe, Germany). The emitter heating current was set to 20 mA min^{-1} at a constant rate.

X-Ray data collection of the compounds was conducted with an Oxford Synergy. The structures were solved using dual space FT and direct methods, refined with the Shelx software package and expanded using Fourier techniques.[36-39] The crystals of all compounds were mounted in an inert oil (perfluoropolyalkylether). Crystal structure determination was affected at 100 K. Crystallographic data (including structure factors) have been deposited with the Cambridge Crystallographic Data Centre as supplementary publication no. CCDC 2350308-2350312, 2366468 and 2366469. For explicit assignment, see chapter 5.1. Copies of the data can be obtained free of charge on application to Cambridge Crystallographic Data Centre, 12 Union Road, Cambridge CB2 1EZ, UK; [fax: (+44) 1223-336-033; email: deposit@ccdc.cam.ac.uk].

Safety comments
Caution! Metal bases such as organolithium bases, metal alkoxides or amides, especially as neat compounds, are severely air-/moisture-sensitive and pyrophoric organometallic compounds. These compounds need to be handled under an inert gas atmosphere to exclude reactions with oxygen and water. Guidelines for their handling can be found in literature: T. L. Rathman, J. A. Schwindeman, *Org. Process Res. Dev.* **2014**, *18*, 1192.

Caution! Carbon monoxide (CO) is a highly toxic gas. Reactions should be performed in well-ventilated fumehoods, ideally with a CO sensor.

2 Experimental Procedures

2.1 Synthesis of 2

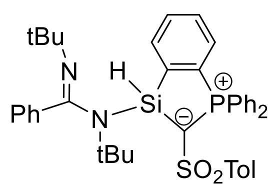

307 mg (0.68 mmol, 1 eq.) **^{Ts}Y'-Li** and 200 mg (0.68 mmol, 1 eq.) PhC(N*t*Bu$_2$)$_2$SiCl (**1**) were dissolved in 10 mL diethyl ether at 0 °C. The reaction was allowed to warm to room temperature overnight and subsequently filtered. The reaction solution was concentrated and stored at -30 °C upon which yellow crystals formed. The crystals were filtered off and washed with cold pentane and dried *in vacuo* to obtain the product as a yellow solid (322 mg, 0.47 mmol, 69%). Single crystals suitable for X-ray diffraction analysis were formed while keeping the reaction solution at -30 °C in diethyl ether.

^{1}H-NMR (400.3 MHz, CD$_2$Cl$_2$): δ = 8.55 (dd, $^{3}J_{HH}$ = 7.5 Hz,$^{4}J_{HH}$ = 2.2 Hz, 1H, C$H_{SiPh,o}$), 7.87 – 7.77 (m, 3H, C$H_{SiPh,m+p}$), 7.63 – 7.54 (m, 6H, C$H_{PPh,o,\ CPh,o}$), 7.53 – 7.49 (m, 1H, C$H_{CPh,p}$), 7.49 – 7.42 (m, 6H, C$H_{PPh2,m+p}$), 7.41 – 7.36 (m, 2H, C$H_{CPh,m}$), 7.22 (d, $^{3}J_{HH}$ = 8.0 Hz, 2H, C$H_{Tol,o}$), 6.91 (d, $^{3}J_{HH}$ = 8.0 Hz, 2H, C$H_{Tol,m}$), 5.65 (d, $^{3}J_{HP}$ = 8.2 Hz, Si*H*), 2.25 (s, 1H, CH_3), 1.07 (s, 18H, C(CH_3)$_3$) ppm.
^{13}C{^{1}H}-NMR (100.7 MHz, CD$_2$Cl$_2$): δ = 163.1 (s, N*C*N), 149.9 (d, $^{2}J_{PC}$ = 19.8 Hz, SiC_{Ph}), 146.4 (s, $C_{Tol,ipso}$), 141.1 (s, $C_{Ph,ipso}$), 140.4 (s, $C_{Tol,p}$), 136.3 (d, $^{3}J_{PC}$ = 13.9 Hz, Si$C_{Ph,o}$), 134.4 (d, $^{1}J_{PC}$ = 98.1 Hz, SiC$_{Ph,o}$), 134.0 (d, $^{2}J_{PC}$ = 10.9 Hz, P$C_{Ph,o}$), 132.3 (s, $C_{Ph,o}$), 131.8 (d, $^{4}J_{PC}$ = 2.9 Hz, P$C_{Ph,p}$), 130.4 (s, Si$C_{Ph,m}$), 130.4 (d, $^{2}J_{PC}$ = 25.2 Hz, Si$C_{Ph,m}$), 130.3 (s, Si$C_{Ph,p}$), 129.0 (s, $C_{Ph,p}$) 128.9 (d, $^{1}J_{PC}$ = 86.0 Hz, P$C_{Ph,ipso}$), 128.8 (d, $^{3}J_{PC}$ = 12.6 Hz, P$C_{Ph,m}$), 128.7 (s, $C_{Tol,m}$), 127.6 (s, $C_{Ph,m}$), 126.1 (s, $C_{Tol,o}$), 55.4 (s, *C*(CH$_3$)$_3$), 44.4 (d, $^{1}J_{PC}$ = 84.1 Hz, P*C*Si), 32.4 (s, C(*C*H$_3$)$_3$), 21.3 (s, *C*H$_3$) ppm.
^{29}Si{^{1}H}-NMR (79.5 MHz, CD$_2$Cl$_2$): δ = -29.1 (d, $^{2}J_{PSi}$ = 34.7 Hz) ppm.
^{31}P{^{1}H}-NMR (162.0 MHz, C$_6$D$_6$): δ = 16.9 (s) ppm.
Elem. Anal. Calcd. for $C_{41}H_{45}N_2O_2PSSi$: C, 71.48; H, 6.58; N, 4.07; S, 4.65. Found: C, 71.29; H, 6.56; N, 3.99; S, 4.28.
HRMS-ESI (m/z): [M-H]$^+$ calcd for $C_{41}H_{46}N_2O_2PSSi$, 689.2776; found, 689.2787.

2.2 Synthesis of AYSi-2

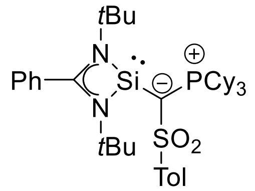

2.27 g (5.0 mmol, 1 eq.) Li[Cy$_3$PCSO$_2$Tol] and 1.47 g (5.0 mmol, 1 eq.) PhC(N*t*Bu$_2$)$_2$SiCl were dissolved in 150 mL toluene and stirred for 2 h at room temperature. The reaction mixture was filtered, and the volatiles were removed *in vacuo*. The residue was washed with 50 mL pentane and subsequently dried to yield the product as a yellow solid (3.07 g, 4.3 mmol, 87%). Single crystals suitable for X-ray diffraction analysis was obtained by pentane vapor diffusion into a saturated benzene solution of **AYSi-2**.

^{1}H-NMR (400.3 MHz, C$_6$D$_6$): δ = 8.35 (d, $^{3}J_{HH}$ = 7.8 Hz, 2H, C$H_{Tol,o}$), 7,68 (d, $^{3}J_{HH}$ = 7.6 Hz, 1H, C$H_{Ph,o}$), 7.12 (d, $^{3}J_{HH}$ = 7.8 Hz, 2H C$H_{Tol,m}$), 7.09 – 6.97 (m, 3H, C$H_{Ph,o+m}$), 6.95 – 6.87 (m, 1H, C$H_{Ph,p}$), 3.42 – 3.24 (m, 3H, C$H_{Cy,ipso}$), 2.30 – 2.15 (m, 6H, C$H_{Cy,m}$), 2.07 (s, 3H,

$CH_{3,Tol}$), 1.89 – 1.71 (m, 12H, $CH_{Cy,o}$), 1.69 – 1.57 (m, 4, $CH_{Cy,p}$), 1.42 – 1.15 (m, 26H, $CH_{Cy,m+p}$, $CH_{3,tBu}$) ppm.

$^{13}C\{^1H\}$-NMR (100.7 MHz, C_6D_6): δ = 157.1 (s, NCN), 150.5 (s, $C_{Tol,ipso}$), 139.3 (s, $C_{Tol,p}$), 136.0 (s, $C_{Ph,ipso}$), 131.3 (s, $C_{Ph,o}$), 129.3 (s, $C_{Tol,m}$), 129.0 (s, $C_{Tol,p}$), 128.5 (s, $C_{Tol,o}$), 127.2 (s, $C_{Ph,m}$), 53.8 (s, $C(CH_3)_3$), 48.1 (d, $^1J_{PC}$ = 58.6 Hz, PCSi), 35.2 (d, $^1J_{PC}$ = 48.7 Hz, $PC_{Cy,ipso}$), 32.0 (s, $C(CH_3)_3$), 28.8 (d, $^3J_{PC}$ = 6.2 Hz, $PC_{Cy,m}$), 27.7 (d, $^2J_{PC}$ = 6.2 Hz, $PC_{Cy,o}$), 26.7 (s, $PC_{Cy,p}$), 21.2 (s, $CH_{3,Tol}$) ppm.

$^{29}Si\{^1H\}$-NMR (79.5 MHz, C_6D_6): δ = 7.7 (d, $^2J_{PSi}$ = 44.7 Hz) ppm.

$^{31}P\{^1H\}$-NMR (162.0 MHz, C_6D_6): δ = 33.2 (s) ppm.

Elem. Anal. Calcd. for $C_{41}H_{63}N_2O_2PSSi$: C, 69.64; H, 8.98; N, 3.96; S, 4.53. Found: C, 64.49; H, 8.74; N, 3.63; S, 3.06. Repeated measurements always resulted in lower values for carbon and sulfur due to incomplete combustion.

2.3 Synthesis of AYSi-3

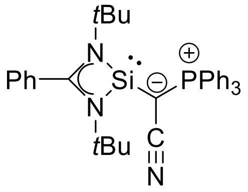

0.83 g (2.81 mmol, 1 eq.) $K[Ph_3PCCN]$ and 0.96 g (2.81 mmol, 1 eq.) $PhC(NtBu_2)_2SiCl$ were dissolved in 10 mL diethyl ether and stirred overnight at room temperature. The reaction mixture was filtered and the volatiles removed *in vacuo*. The residue was washed with twice with 5 mL pentane and dried. The product was obtained as an orange solid (1.23 g, 2.2 mmol, 78%). Single crystals suitable for X-ray diffraction analysis were obtained by storage of a saturated diethyl ether solution of **AYSi-3** at -30 °C.

^1H-NMR (400.3 MHz, C_6D_6): δ = 8.23 – 8.15 (m, 1H, $CH_{CPh,p}$), 7.93 – 7.82 (m, 6H, $CH_{PPh3,m}$), 7.10 – 7.01 (m, 9H, $CH_{PPh,o+pz}$), 7.00 – 6.89 (m, 4H, $CH_{CPh,o+m}$), 1.35 (s, 18H, $C(CH_3)_3$) ppm.

$^{13}C\{^1H\}$-NMR (100.7 MHz, C_6D_6): δ = 162.80 (s, NCN), 135.25 (s, $C_{CPh,ipso}$), 133.97 (d, $^2J_{PC}$ = 9.3 Hz, $C_{PPh3,o}$), 133.16 (s, $C_{CPh,ipso}$), 131.83 (d, $^4J_{PC}$ = 2.9 Hz, $C_{PPh3,p}$), 130.24 (s, $C_{CPh,o}$), 129.27 (d, $^1J_{PC}$ = 18.7 Hz, $C_{PPh3,ipso}$), 128.65 (d, $^3J_{PC}$ = 12.4 Hz, $C_{PPh3,m}$), 127.22 (s, $C_{Ph,m}$), 125.00 (d, $^2J_{PC}$ = 6.3 Hz, CCN), 53.01 (s, $C(CH_3)_3$), 31.71 (s, $C(CH_3)_3$), 15.87 (d, $^1J_{PC}$ = 106.8 Hz, CCN) ppm.

$^{29}Si\{^1H\}$-NMR (79.5 MHz, C_6D_6): δ = 23.68 (d, $^2J_{PSi}$ = 62.3 Hz) ppm.

$^{31}P\{^1H\}$-NMR (162.0 MHz, C_6D_6): δ = 24.9 (s) ppm.

HRMS-ESI (m/z): $[M-H]^+$ calcd for $C_{35}H_{39}N_3PSi$, 560.2632; found, 560.2650.

2.4 Synthesis of AYSi-2 nickel complex 3

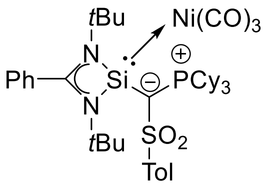

70.6 mg (0.10 mmol, 1 eq.) **AYSI-2** was dissolved in 5 mL toluene and cooled to -30 °C and added to 27.5 mg (0.10 mmol, 1 eq.) $Ni(COD)_2$ at -30 °C in the dark. The solution was stirred for 5 minutes, and the reaction mixture was allowed to warm to room temperature. The volatiles were removed *in vacuo*. 5 mL pentane were added to the residue and the solution was filtered. The filtrate was dried *in vacuo* to yield the product as a colorless solid (60.0 mg, 0.07 mmol, 71%).

^1H-NMR (400.3 MHz, C_6D_6): δ = 8.36 (d, $^3J_{HH}$ = 8.2 Hz, 2H, $CH_{Tol,o}$), 7.88 (d, $^3J_{HH}$ = 8.4 Hz, $CH_{Ph,o}$), 7.57 (d, $^3J_{HH}$ = 7.6 Hz, $CH_{Ph,o}$), 6.97 – 6.87 (m, 5H, $CH_{Ph,m+p}$ + $CH_{Tol,m}$), 3.25 (q, $^3J_{HH}$ = 12.9 Hz, 3H,

$CH_{Cy,ipso}$), 2.31 – 2.18 (m, 6H, $CH_{Cy,p}$), 2.02 (s, 3H, $CH_{3,Tol}$), 1.70 – 1.41 (m, 30H, $CH_{Cy,o}$ + $C(CH_3)_3$), 1.38 – 1.20 (m, 8H, $CH_{Cy,m}$), 1.18 – 0.95 (m, 4H, $CH_{Cy,m}$) ppm.
$^{13}C\{^1H\}$-NMR (100.7 MHz, C_6D_6): δ = 202.7 (s, *C*O), 168.0 (s, N*C*N), 150.6 (s, $C_{Tol,ipso}$), 140.0 (s, $C_{Tol,p}$), 133.8 (s, $C_{Ph,ipso}$), 131.7k (s, $C_{Ph,o}$), 129.7 (s, $C_{Ph,p}$), 128.7 (s, $C_{Tol,m}$), 127.6 (s, $C_{Ph,m}$), 126.6 (s, $C_{Tol,o}$), 55.0 (s, $C(CH_3)_3$), 45.8 (d, $^1J_{PC}$ = 55.6 Hz, P*C*Si), 34.7 (d, $^1J_{PC}$ = 46.6 Hz, P$C_{Cy,ipso}$), 32.0 (s, $C(CH_3)_3$), 29.1 (d, $^3J_{PC}$ = 3.3 Hz, P$C_{Cy,m}$), 27.3 (d, $^2J_{PC}$ = 11.8 Hz, P$C_{Cy,o}$), 26.4 (s, P$C_{Cy,p}$), 21.1 (s, $CH_{3,Tol}$) ppm.
$^{29}Si\{^1H\}$-NMR (79.5 MHz, C_6D_6): δ = 63.0 (d, $^2J_{PSi}$ = 33.7 Hz) ppm.
$^{31}P\{^1H\}$-NMR (162.0 MHz, C_6D_6): δ = 31.9 (s) ppm.
Elem. Anal. Calcd. for $C_{44}H_{63}N_2NiO_5PSSi$: C, 62.19; H, 7.47; N, 3.30; S, 3.77. Found: C, 62.13; H, 7.30; N, 3.32; S, 2.48.

2.5 Synthesis of AYSi-2 gold complex 4

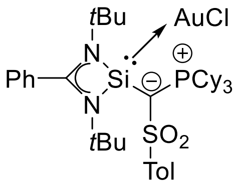

70.6 mg (0.10 mmol, 1 eq.) **AYSi-2** and 32.1 mg (0.10 mmol, 1 eq.) (THT)AuCl were dissolved in 5 mL toluene and stirred for one hour at room temperature. The reaction mixture was filtered over Celite, and the volatiles removed *in vacuo*. The product was obtained as a colourless solid (88.2 mg, 0.094 mmol, 94%).

^1H-NMR (400.3 MHz, C_6D_6): δ = 8.25 (d, $^3J_{HH}$ = 8.2 Hz, 2H, $CH_{Tol,o}$), 7.49 (d, $^3J_{HH}$ = 8.0 Hz, 1H, $CH_{Ph,p}$), 6.98 – 6.90 (m, 4H, $CH_{Ph,o+m}$), 6.97 – 6.90 (m, 2H $CH_{Tol,m}$), 3.60 – 3.40 (m, 2H, $CH_{Cy,ipso}$), 2.58 – 2.51 (m, 1H, $CH_{Cy,ipso}$), 2.03 (s, 3H, $CH_{3,Tol}$), 1.98 – 1.83 (m, 6H, $CH_{Cy,p}$), 1.64 – 1.35 (m, 34H, $CH_{Cy,o+m}$ + $C(CH_3)_3$), 1.29 – 0.96 (m, 8H, $CH_{Cy,m}$) ppm.
$^{13}C\{^1H\}$-NMR (100.7 MHz, C_6D_6): δ = 173.7 (s, N*C*N), 149.5 (s, $C_{Tol,ipso}$), 140.6 (s, $C_{Tol,p}$), 133.8 (s, $C_{Ph,ipso}$), 131.7 (s, $C_{Ph,p}$), 129.7 (s, $C_{Ph,o}$), 129.5 (s, $C_{Ph,o}$), 128.9 (s, $C_{Ph,m}$), 128.7 (s, $C_{Tol,o}$), 127.6 (s, $C_{Ph,m}$), 126.6 (s, $C_{Tol,m}$), 54.5 (s, $C(CH_3)_3$), 46.4 (d, $^1J_{PC}$ = 44.7 Hz, P*C*Si), 37.4 (d, $^1J_{PC}$ = 46.7 Hz, P$C_{Cy,ipso}$), 32.2 (s, $C(CH_3)_3$), 31.5 (d, $^1J_{PC}$ = 51.9 Hz, P$C_{Cy,ipso}$), 28.7 (br d, $^3J_{PC}$ = 3.2 Hz, P$C_{Cy,m}$), 27.4 (br d, $^2J_{PC}$ = 12.1 Hz, P$C_{Cy,o}$), 26.5 (s, P$C_{Cy,p}$), 21.1 (s, $CH_{3,Tol}$) ppm.
$^{29}Si\{^1H\}$-NMR (79.5 MHz, C_6D_6): δ = 28.1 (d, $^2J_{PSi}$ = 26.9 Hz) ppm.
$^{31}P\{^1H\}$-NMR (162.0 MHz, C_6D_6): δ = 31.0 (s) ppm.
Elem. Anal. Calcd. for $C_{41}H_{63}AuClN_2O_2PSSi$: C, 54.22; H, 6.76; N, 2.98; S, 3.41. Found: C, 54.18; H, 6.47; N, 2.56; S, 2.95.

2.6 Synthesis of AYSi-2-CS$_2$ adduct 5$_{Ts}$

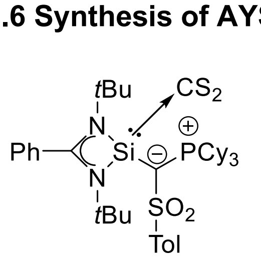

70.6 mg (0.10 mmol, 1 eq.) **AYSi-2** were dissolved in 3 ml of toluene and 6 µL CS_2 (0.10 mmol, 1. eq.) were added. The reaction mixture turned red and was stirred for 5 minutes at room temperature. The volatiles were removed *in vacuo* and the residue washed with 5 ml pentane. The remaining solid was dried *in vacuo* to yield the product as a purple powder (68.4 mg, 0.09 mmol, 87%). Single crystals suitable for X-ray diffraction analysis were obtained by slow evaporation of a saturated dichloromethane solution of **5$_{Ts}$**.

^{1}H-NMR (400.3 MHz, C_6D_6): δ = 8.30 (d, $^3J_{HH}$ = 8.2 Hz, 2H, C$H_{Tol,o}$), 7.41 (d, $^3J_{HH}$ = 8.2, 1H, C$H_{Ph,p}$), 7.06 (d, 2H, $^3J_{HH}$ = 7.2 Hz, 1H, C$H_{Ph,o}$), 6.94 (d, $^3J_{HH}$ = 8.2 Hz, 2H C$H_{Tol,m}$), 6.88 (d, $^3J_{HH}$ = 7.2 Hz, 1H, C$H_{Ph,o}$), 6.85 – 6.78 (m, 2H, C$H_{Ph,m}$), 3.81 – 3.66 (m, 3H, C$H_{Cy,ipso}$), 2.39 – 2.28 (m, 6H, C$H_{Cy,m}$), 2.00 (s, 3H, C$H_{3,Tol}$), 1.63 – 1.53 (m, 24H, C$H_{Cy,m}$, C$H_{3,tBu}$), 1.45 – 1.29 (m, 12H, C$H_{Cy,o}$), 1.08 – 0.93 (m, 6H, C$H_{Cy,p}$) ppm.
^{13}C{^{1}H}-NMR (100.7 MHz, C_6D_6): δ = 273.3 (s, CS_2), 180.0 (s, NCN), 148.6 (s, $C_{Tol,ipso}$), 141.4 (s, $C_{Tol,p}$), 131.0 (s, $C_{Ph,ipso}$), 130.5 (s, $C_{Ph,o}$), 129.5 (s, $C_{Tol,p}$), 129.1 (s, $C_{Tol,o}$), 128.5 (s, $C_{Ph,m}$), 127.4 (s, $C_{Tol,m}$), 55.4 (s, $C(CH_3)_3$), 40.7 (d, $^1J_{PC}$ = 60.7 Hz, PCSi), 38.0 (d, $^1J_{PC}$ = 43.3 Hz, P$C_{Cy,ipso}$), 31.6 (s, $C(CH_3)_3$), 29.4 (d, $^3J_{PC}$ = 3.3 Hz, P$C_{Cy,m}$), 27.2 (d, $^2J_{PC}$ = 12.4 Hz, P$C_{Cy,o}$), 26.4 (s, P$C_{Cy,p}$), 21.1 (s, $CH_{3,Tol}$) ppm.
^{29}Si{^{1}H}-NMR (79.5 MHz, C_6D_6): δ = -39.4 (d, $^2J_{Psi}$ = 21.5 Hz) ppm.
^{31}P{^{1}H}-NMR (162.0 MHz, C_6D_6): δ = 32.8 (s) ppm.

2.7 Synthesis of AYSi-3-CS$_2$ adduct 5$_{CN}$

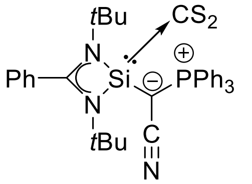

56.1 mg (0.10 mmol, 1 eq.) **AYSi-3** were dissolved in 1 ml of C_6D_6 and 7.2 µL CS_2 (0.12 mmol, 1.2 eq.) were added. The reaction mixture turned red and was stirred for 5 minutes at room temperature. The reaction mixture was characterized by multinuclear NMR spectroscopy. Single crystals suitable for X-ray diffraction analysis were obtained by adding CS_2 to a concentrated solution of **AYSi-3** in toluene at -30 °C. The product could not be isolated.

^{1}H-NMR (400.3 MHz, C_6D_6): δ = 7.90 – 7.81 (m, 6H, C$H_{PPh,m}$), 7.47 (d, $^3J_{HH}$ = 8.0, 1H, C$H_{Ph,p}$), 7.08 – 7.02 (m, 9H, C$H_{PPh,o+p}$), 6.99 (d, 2H, $^3J_{HH}$ = 7.7 Hz, 1H, C$H_{Ph,o}$), 6.86 (d, $^3J_{HH}$ = 7.7 Hz, 1H, C$H_{Ph,o}$), 6.81 – 6.73 (m, 2H, C$H_{Ph,m}$), 1.42 (s, 18H, C$H_{3,tBu}$) ppm.
^{13}C{^{1}H}-NMR (100.7 MHz, C_6D_6): δ = 269.9 (s, CS_2), 180.6 (s, NCN), 134.1 (d, $^3J_{PC}$ = 10.2 Hz, $C_{PPh3,m}$), 132.6 (d, $^4J_{PC}$ = 3.1 Hz, $C_{PPh3,p}$), 130.6 (s, $C_{CPh,o}$), 129.5 (s, $C_{CPh,p}$), 128.9 (d, $^2J_{PC}$ = 12.5 Hz, $C_{PPh3,o}$), 128.5 (d, $^2J_{PC}$ = 12.4 Hz, CCN), 127.7 (d, $^3J_{PC}$ = 12.4 Hz, $C_{PPh3,m}$), 127.7 (s, $C_{Ph,m}$), 126.9 (d, $^1J_{PC}$ = 126.9 Hz, $C_{PPh3,ipso}$), 124.3 (s, $C_{CPh,ipso}$), 56.1 (s, $C(CH_3)_3$), 31.1 (s, $C(CH_3)_3$), 3.4 (d, $^1J_{PC}$ = 117.5 Hz, CCN) ppm.
^{29}Si{^{1}H}-NMR (79.5 MHz, C_6D_6): δ = -48.9 (d, $^2J_{Psi}$ = 22.0 Hz) ppm.
^{31}P{^{1}H}-NMR (162.0 MHz, C_6D_6): δ = 25.9 (s) ppm.

2.8 Synthesis of Silanone 6

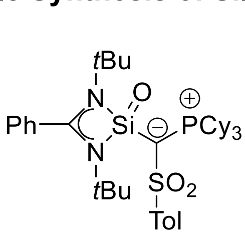

70.6 mg (0.10 mmol, 1 eq.) **AYSi-2** were added to a Schlenk tube and the atmosphere was exchanged to N_2O. The powder was stirred in that atmosphere overnight to yield the product as a colorless solid (65.4 mg, 0.09 mmol, 90%). Single crystals suitable for X-ray diffraction analysis were obtained by slow evaporation of a saturated benzene solution of **6**.

^{1}H-NMR (400.3 MHz, C_4D_8O): δ = 8.10 (d, $^3J_{HH}$ = 7.9 Hz, 2H, C$H_{Tol,o}$), 7.61 – 7.54 (m, 2H, C$H_{Ph,o}$), 7.50 – 7.42 (m, 2H, C$H_{Ph,m}$), 7.41 – 7.33 (m, 1H, C$H_{Ph,p}$), 7.18 (d, $^3J_{HH}$ = 7.9 Hz, 2H C$H_{Tol,m}$), 3.54

– 3.38 (m, 3H, C$H_{Cy,ipso}$), 2.35 (s, 3H, C$H_{3,Tol}$), 1.92 – 1.78 (m, 6H, C$H_{Cy,m}$), 1.76 – 1.54 (m, 14H, C$H_{Cy,o+p}$), 1.36 – 1.08 (m, 28H, C$H_{Cy,m+p}$, C$H_{3,tBu}$) ppm.
^{13}C{^{1}H}-NMR (100.7 MHz, C_4D_8O): δ = 178.7 (s, NCN), 151.4 (s, $C_{Tol,ipso}$), 140.6 (s, $C_{Tol,p}$), 133.6 (s, $C_{Ph,ipso}$), 131.1 (s, $C_{Ph,o}$), 131.0 (s, $C_{Ph,o}$), 129.1 (s, $C_{Tol,m}$), 128.9 (s, $C_{Ph,m}$), 128.8 (s, $C_{Ph,m}$), 128.5 (s, $C_{Tol,p}$), 127.3 (s, $C_{Ph,o}$), 55.4 (s, C(CH$_3$)$_3$), 35.2 (d, $^1J_{PC}$ = 58.4 Hz, PCSi), 34.4 (d, $^1J_{PC}$ = 47.4 Hz, P$C_{Cy,ipso}$), 32.2 (s, C(CH$_3$)$_3$), 29.3 (d, $^3J_{PC}$ = 3.0 Hz, P$C_{Cy,m}$), 28.4 (d, $^2J_{PC}$ = 12.4 Hz, P$C_{Cy,o}$), 27.3 (s, P$C_{Cy,p}$), 21.4 (s, CH$_{3,Tol}$) ppm.
^{29}Si{^{1}H}-NMR (79.5 MHz, C_6D_6): δ = -40.6 (d, $^2J_{Psi}$ = 19.3 Hz) ppm.
^{31}P{^{1}H}-NMR (162.0 MHz, C_6D_6): δ = 32.3 (s) ppm.
Elem. Anal. Calcd. for $C_{41}H_{63}N_2O_3PSSi$: C, 68.10; H, 8.78; N, 3.87; S, 4.43. Found: C, 67.77; H, 8.39; N, 4.00; S, 4.15.

Alternative Synthesis of Silanone 6:
60.0 mg (84,9 µmol, 1 eq) **AYSi-2** were weight into a Schlenk flask and dissolved in 1.5 ml of toluene. The atmosphere was changed to 1.5 bar CO_2 and the solution was stirred for 1 d at RT. The next day the yellow solution had become a colorless suspension. The colorless precipitate was isolated by filtration and was washed with pentane (1.5 mL). The remaining colorless solid was identified as silanone **6** (22.1 mg, 30.6 µmol, 36 %).

2.9 Synthesis of Siloxane 7

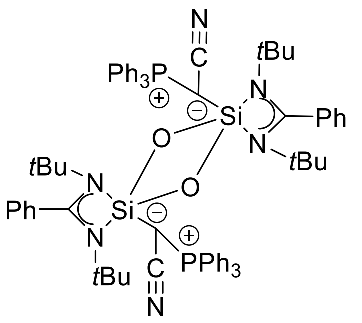

28 mg (0.05 mmol, 1 eq.) **AYSi-3** were dissolved in dry C_6D_6 in a J Young tube and the atmosphere was exchanged to 1 bar of N_2O. The reaction was stirred for one hour at room temperature and subsequently submitted for NMR experiments. Single crystals suitable for X-ray diffraction analysis formed during the reaction without stirring.

^{1}H-NMR (400.3 MHz, C_6D_6): δ = 8.15 – 8.04 (m, 8H, C$H_{PPh,o}$ + C$H_{CPh,o}$), 7.10 – 7.02 (m, 9H, C$H_{PPh3,m+p}$), 6.93 – 6.82 (m, 3H, C$H_{Ph,m+p}$), 1.31 (s, 18H, C(CH_3)$_3$) ppm.
^{13}C{^{1}H}-NMR (100.7 MHz, C_6D_6): δ = 175.84 (s, NCN), 134.57 (d, $^2J_{PC}$ = 10.2 Hz, $C_{PPh3,o}$), 132.17 (d, $^4J_{PC}$ = 2.9 Hz, $C_{PPh3,p}$), 131.41 (s, $C_{CPh,ipso}$), 129.32 (s, $C_{CPh,o}$), 129.27 (d, $^1J_{PC}$ = 18.7 Hz, $C_{PPh3,ipso}$), 128.72 (d, $^3J_{PC}$ = 12.4 Hz, $C_{PPh3,m}$), 127.68 (s, $C_{Ph,m}$), 126.92 (d, $^2J_{PC}$ = 2.7 Hz, CCN), 54.05 (s, C(CH$_3$)$_3$), 31.29 (s, C(CH$_3$)$_3$), 2.63 (d, $^1J_{PC}$ = 105.7 Hz, CCN) ppm.
^{29}Si{^{1}H}-NMR (79.5 MHz, C_6D_6): δ = -40.7 (d, $^2J_{PSi}$ = 18.2 Hz) ppm.
^{31}P{^{1}H}-NMR (162.0 MHz, C_6D_6): δ = 28.9 (s) ppm.

2.10 Synthesis of AYSi-2 carbonate complex 8

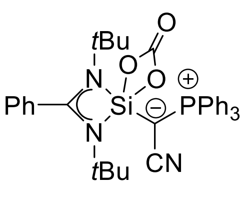

In a Schlenk flask, 60 mg (107 µmol, 1 eq) **AYSi-3** were dissolved in 2 ml toluene. The atmosphere was exchanged to 2 bar of CO_2 which led within minutes to a color change of the solution from orange to violet. After further stirring for 1 d at RT the solution had turned into a colorless suspension. The colorless precipitate was isolated from the supernatant solution. The residue

was dried *in vacuo* to yield the product as a white solid (25.8 mg, 41.6 µmol, 39%). Crystals suitable for XRD were won by slow vapor diffusion of hexane into a saturated solution of **8** in benzene.

^{1}H-NMR (400 MHz, C_6D_6) δ = 7.92 (ddd, $^{3}J_{PH}$ = 12.4, $^{3}J_{HH}$ = 7.4, $^{4}J_{HH}$ = 2.1 Hz, 6H, C$H_{PPh,o}$), 7.72 (d, $^{3}J_{HH}$ = 7.6 Hz, 1H, C$H_{Ph,p}$), 7.08 (m, 9H, C$H_{PPh,m}$ + C$H_{PPh,p}$), 6.85 (m, 4H, C$H_{Ph,o+p}$), 1.22 (s, 18H, C$H_{3,tBu}$) ppm.
^{13}C{^{1}H}-NMR (101 MHz, C_6D_6) δ = 176.3 (NCN), 153.1 (O(CO)O), 133.5 (d, $^{2}J_{PC}$ = 9.5 Hz, C$H_{PPh,o}$), 132.2 (d, $^{4}J_{PC}$ = 2.9 Hz, C$H_{PPh,p}$), 132.1 (C$H_{CPh,ipso}$), 130.3 (C$H_{CPh,p}$), 130.1 (C$H_{Ph,m}$), 129.1 (d, $^{3}J_{PC}$ = 12.1 Hz, C$H_{PPh,m}$), 127.7 (C$H_{CPh,o}$), 126.5 (C$H_{PPh,ipso}$), 54.6 (C(CH$_3$)$_{3,tBu}$), 31.1 (C(CH$_3$)$_{3,tBu}$), 2.4 (d, $^{1}J_{PH}$ = 101.1 Hz, CCN) ppm.
^{29}Si{^{1}H}-NMR (80 MHz, C_6D_6) δ = -90.2 (d, $^{2}J_{PSi}$ = 18.0 Hz) ppm.
^{31}P{^{1}H}-NMR (162 MHz, C_6D_6) δ = 28.2 ppm.

2.10 Synthesis of Silazine $\mathbf{9_{Ts}}$

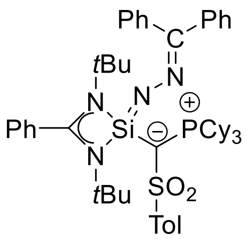

70.6 mg (0.10 mmol, 1 eq.) **AYSi-2** and 19.4 mg (0.10 mmol, 1 eq.) 1,1'-diphenyl diazomethane were dissolved in 3 ml toluene and the reaction was stirred overnight at room temperature. The reaction mixture was filtered, and the precipitate was washed with 3 ml cold toluene and subsequently dried *in vacuo*. The precipitate was recrystallized by slow vapor diffusion of hexane into a saturated solution of $\mathbf{9_{Ts}}$ in THF. The supernatant solution was taken off and the crystals were dried *in vacuo* to obtain the product as a yellow solid (50.3 mg, 0.06 mmol, 62%). After recrystallization, a few crystals were taken for X-ray diffraction analysis.

^{1}H-NMR (400.3 MHz, C_4D_8O): δ = 8.07 (d, $^{3}J_{HH}$ = 7.9 Hz, 2H, C$H_{Tol,o}$), 7.76 (d, $^{3}J_{HH}$ = 7.4 Hz, 1H, C$H_{Ph,o}$), 7.63 (d, $^{3}J_{HH}$ = 7.4 Hz, 1H, C$H_{Ph,o}$), 7.57 – 7.47 (m, 4H, C$H_{Ph2,o}$), 7.42 (t, $^{3}J_{HH}$ = 7.4 Hz, 1H C$H_{Ph,p}$), 7.34 – 7.27 (m, 4H, C$H_{Ph2,m}$), 7.23 (d, $^{3}J_{HH}$ = 7.9 Hz, C$H_{Tol,m}$), 7.18 – 7.12 (m, 1H, C$H_{Ph,o}$), 7.08 (d, $^{3}J_{HH}$ = 7.4 Hz, 2H, C$H_{Ph2,p}$), 6.95 – 6.87 (m, 1H, C$H_{Ph,m}$), 3.24 – 3.10 (m, 3H, C$H_{Cy,ipso}$), 2.36 (s, 3H, C$H_{3,Tol}$), 1.82 – 1.66 (m, 12H, C$H_{Cy,o}$), 1.64 – 1.53 (m, 6H, C$H_{Cy,m}$), 1.49 – 1.38 (m, 6H, C$H_{Cy,p}$), 1.32 (s, 18H, C$H_{3,tBu}$), 1.11 – 1.04 (m, 6H, C$H_{Cy,m}$) ppm.
^{13}C{^{1}H}-NMR (100.7 MHz, C_4D_8O): δ = 178.0 (s, NCN), 150.5 (s, $C_{Tol,ipso}$), 146.5 (s, C_{N2}), 144.4 (s, $C_{Ph2,ipso}$), 142.9 (s, $C_{Ph2,ipso}$), 141.1 (s, $C_{Tol,p}$), 133.1 (s, $C_{Ph,ipso}$), 132.1 (s, $C_{Ph2,o}$), 131.2 (s, $C_{Ph,m}$), 131.0 (s, $C_{Ph,o}$), 129.6 (s, $C_{Ph,o}$), 129.3 (s, $C_{Tol,m}$), 128.8 (s, $C_{Ph,p}$), 128.5 (s, $C_{Ph2,o}$), 128.4 (s, $C_{Ph2,m}$), 128.0 (s, $C_{Ph2,m}$), 127.4 (s, $C_{Tol,o}$), 126.4 (s, $C_{Ph2,p}$), 125.8 (s, $C_{Ph2,o}$), 124.5 (s, $C_{Ph2,p}$), 55.3 (s, C(CH$_3$)$_3$), 35.0 (d, $^{1}J_{PC}$ = 47.4 Hz, P$C_{Cy,ipso}$), 34.6 (d, $^{1}J_{PC}$ = 58.4 Hz, PCSi), 32.2 (s, C(CH$_3$)$_3$), 29.3 (s, P$C_{Cy,m}$), 28.0 (d, $^{2}J_{PC}$ = 12.3 Hz, P$C_{Cy,o}$), 27.0 (s, P$C_{Cy,p}$), 21.4 (s, C$H_{3,Tol}$) ppm.
^{29}Si{^{1}H}-NMR (79.5 MHz, C_6D_6): δ = -16.5 ppm. (A coupling constant was not determined since the shift was determined by ^{1}H-^{29}Si-HMBC experiments.)
^{31}P{^{1}H}-NMR (162.0 MHz, C_6D_6): δ = 31.5 (s) ppm.

2.11 Synthesis of Silazine $\mathbf{9_{CN}}$

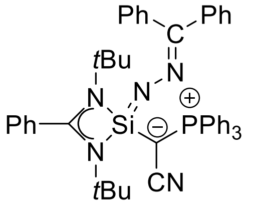

54.9 mg (98.1 µmol, 1 eq) **AYSi-3** were dissolved in 1 ml benzene and a solution of 20.0 mg (103 µmol, 1.05 eq) 1,1'-diphenyl diazomethane in 0.5 ml benzene was added. The orange solution was stirred for 1 d at RT and subsequently overlayered with 3 ml pentane. Within minutes yellow crystals formed. After 1 d the supernatant solution was removed from the yellow solid, which were washed once with 3 ml pentane. The remaining yellow solid (48.1 mg, 63.8 µmol, 65 %) was characterized as the targeted product. Crystals suitable for XRD were won by slow vapor diffusion of hexane into a saturated solution of $\mathbf{9_{CN}}$ in THF.

^{1}H-NMR (400 MHz, THF-d$_8$) δ = 7.74 (m, 6H, C$H_{PPh,o}$), 7.62 (m, 1H, C$H_{PhCPh,p}$), 7.55 (ddt, $^3J_{HH}$ = 7.0, $^4J_{HH}$ = 3.6, $^5J_{PH}$ = 1.4 Hz, 3H, C$H_{PPh,p}$), 7.50 (m, 5H, C$H_{PhCPh,o}$ + C$H_{PhCPh,p}$), 7.36 (td, J = 7.9, 3.2 Hz, 6H, C$H_{PPh,m}$), 7.07 (m, 4H, C$H_{PhCPh,m}$), 7.00 (m, 4H, C$H_{CPh,o}$ + C$H_{CPh,m}$), 6.93 (m, 1H, C$H_{CPh,p}$), 1.13 (s, 18H, C$H_{3,tBu}$) ppm.
^{13}C{^{1}H}-NMR (101 MHz, THF-d$_8$) δ = 177.9 (NCN), 147.7 ($C_{PhCPh,ipso}$), 144.2 (PhCPh), 140.4 ($C_{CPh,ipso}$), 135.2 (d, $^2J_{PC}$ = 10.3 Hz, $C_{PPh,o}$), 132.8 (d, $^4J_{PC}$ = 3.0 Hz, $C_{PPh,p}$), 131.7 ($C_{PhCPh,m}$), 130.1 (d, $^1J_{PC}$ = 382.6 Hz, $C_{PPh,ipso}$), 129.1 (d, $^3J_{PC}$ =12.8 Hz, $C_{PPh,m}$), 129.0 ($C_{PhCPh,p}$), 128.0 ($C_{PhCPh,o}$) 127.9 ($C_{CPh,m}$), 126.3 ($C_{PhCPh,p}$), 125.9 ($C_{CPh,o}$), 125.2 (d, $^2J_{PC}$ = 2.1 Hz, CCN), 124.9 ($C_{CPh,p}$), 54.9 (C(CH$_3$)$_{3,tBu}$), 31.3 (C(CH$_3$)$_{3,tBu}$).
^{31}P{^{1}H}-NMR (162 MHz, THF-d$_8$) δ = 26.6 ppm.
HRMS-LIFDI (m/z): [M]$^+$ calcd for $C_{48}H_{48}N_5PSi$, 753.3417; found, 753.3401.

2.12 Synthesis of 10

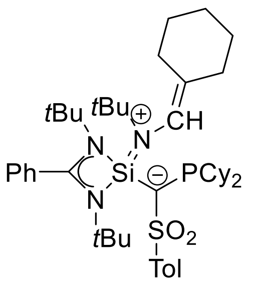

70.6 mg (0.10 mmol, 1 eq.) **AYSi-2** was dissolved in 3 ml toluene and 11.6 µL (0.10 mmol, 1 eq.) *tert*-butyl isocyanide were added. The reaction was heated to 40 °C overnight. After cooling to room temperature, the volatiles were removed *in vacuo* and 5 ml acetonitrile were added to the residue. A white precipitate formed which was filtered and washed with 2 ml of cold pentane. The residue was dried *in vacuo* to yield the product as a white solid (56.9 mg, 0.07 mmol, 72%). Single crystals suitable for X-ray diffraction analysis were obtained by slow evaporation of a saturated pentane solution of **10**.

^{1}H-NMR (400.3 MHz, C$_6$D$_6$): δ = 8.40 (d, $^3J_{HH}$ = 8.3 Hz, 2H, C$H_{Tol,o}$), 7.76 (t, $^3J_{HH}$ = 7.0 Hz, 1H, C$H_{Ph,o}$), 7.18 – 7.15 (m, 1H, C$H_{Ph,o}$), 7.03 (m, $^3J_{HH}$ = 8.3 Hz, 2H, C$H_{Tol,m}$), 6.98 – 6.88 (m, 4H, C$H_{Ph,o+m+p}$), 6.32 (s, 1H, NCH), 2.57 – 2.41 (m, 4H, NC$H_{Cy,o}$), 2.39 – 2.29 (m, 4H, PC$H_{Cy,o}$), 2.25 – 2.17 (m, 2H, PC$H_{Cy,ipso}$), 2.06 (s, 3H, C$H_{3,Tol}$), 1.92 – 1.74 (m, 8H, PC$H_{Cy,m}$), 1.71 (s, 9H, NC$H_{3,tBu}$), 1.64 – 1.52 (m, 6H, NC$H_{Cy,p}$ + PC$H_{Cy,p}$), 1.45 (s, 18H, C$H_{3,tBu}$), 1.39 – 1.29 (m, 4H, NC$H_{Cy,m}$) ppm.
^{13}C{^{1}H}-NMR (100.7 MHz, C$_6$D$_6$): δ = 176.5 (s, NCN), 148.9 (s, $C_{Tol,p}$), 140.0 (s, $C_{Tol,ipso}$), 138.7 (s, NCC_{Cy}), 131.8 (s, $C_{Ph,ipso}$), 130.4 (s, $C_{Ph,o}$), 129.5 (s, $C_{Tol,m}$), 129.4 (s, $C_{Ph,p}$), 128.6 (s, $C_{Tol,o}$), 127.7 (s, $C_{Ph,m}$), 127.1 (s, NCC$_{Cy}$), 57.9 (s, NC(CH$_3$)$_3$), 55.7 (s, C(CH$_3$)$_3$), 47.8 (d, $^1J_{PC}$ = 62.3z Hz, PCSi), 36.1 (d, $^2J_{PC}$ = 14.7 Hz, P$C_{Cy,o}$), 35.0 (s, NC$C_{Cy,o}$), 33.9 (d, $^1J_{PC}$ = 19.4 Hz, P$C_{Cy,ipso}$), 32.4 (d, $^2J_{PC}$ = 14.5 Hz, P$C_{Cy,o}$), 32.3 (s, C(CH$_3$)$_3$), 30.5 (s, NC(CH$_3$)$_3$), 30.3 (s, NC$C_{Cy,o}$), 28.5 (s, P$C_{Cy,p}$),

28.3 (d, $^3J_{PC}$ = 9.1 Hz, P$C_{Cy,m}$), 28.2 (s, NC$C_{Cy,m}$), 27.5 (s, P$C_{Cy,p}$), 27.3 (s, NC$C_{Cy,m}$), 26.7 (s, NC$C_{Cy,m}$), 21.2 (s, $CH_{3,Tol}$) ppm.
^{29}Si{^{1}H}-NMR (79.5 MHz, C_6D_6): δ = -42.1 ppm. (A coupling constant was not determined since the shift was determined by ^{1}H-^{29}Si-HMBC experiments.)
^{31}P{^{1}H}-NMR (162.0 MHz, C_6D_6): δ = -13.9 (s) ppm.
HRMS-LIFDI (m/z): $[M]^+$ calcd for $C_{46}H_{72}N_3O_2PSSi$, 789.4852; found, 789.4836.

3 Stability test monitored by NMR spectroscopy

3.1 Stability of silylene AYSi-2 in toluene

Procedure for stability testing: **AYSi-2** (20 mg) was dissolved in 0.6 mL toluene inside a J-Young NMR tube, which was heated to 50 °C over a period of 2 weeks. To represent the progress of decomposition, the ^{31}P NMR spectra measured before the heating, after 1 d, after 5 d, after 1 week and after 2 weeks are depicted.

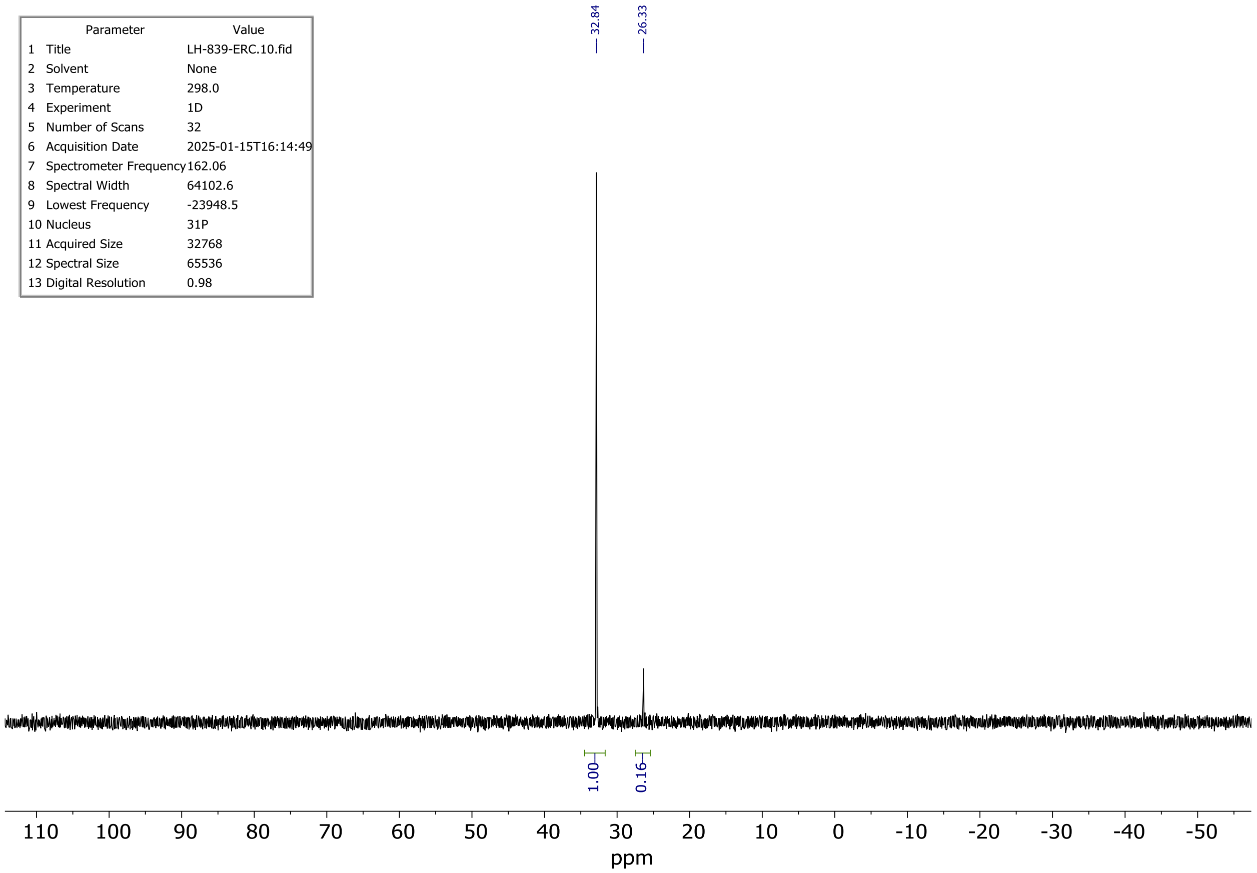

Figure S1. $^{31}P\{^1H\}$-NMR spectrum of **AYSi-2** measured in toluene before heating.

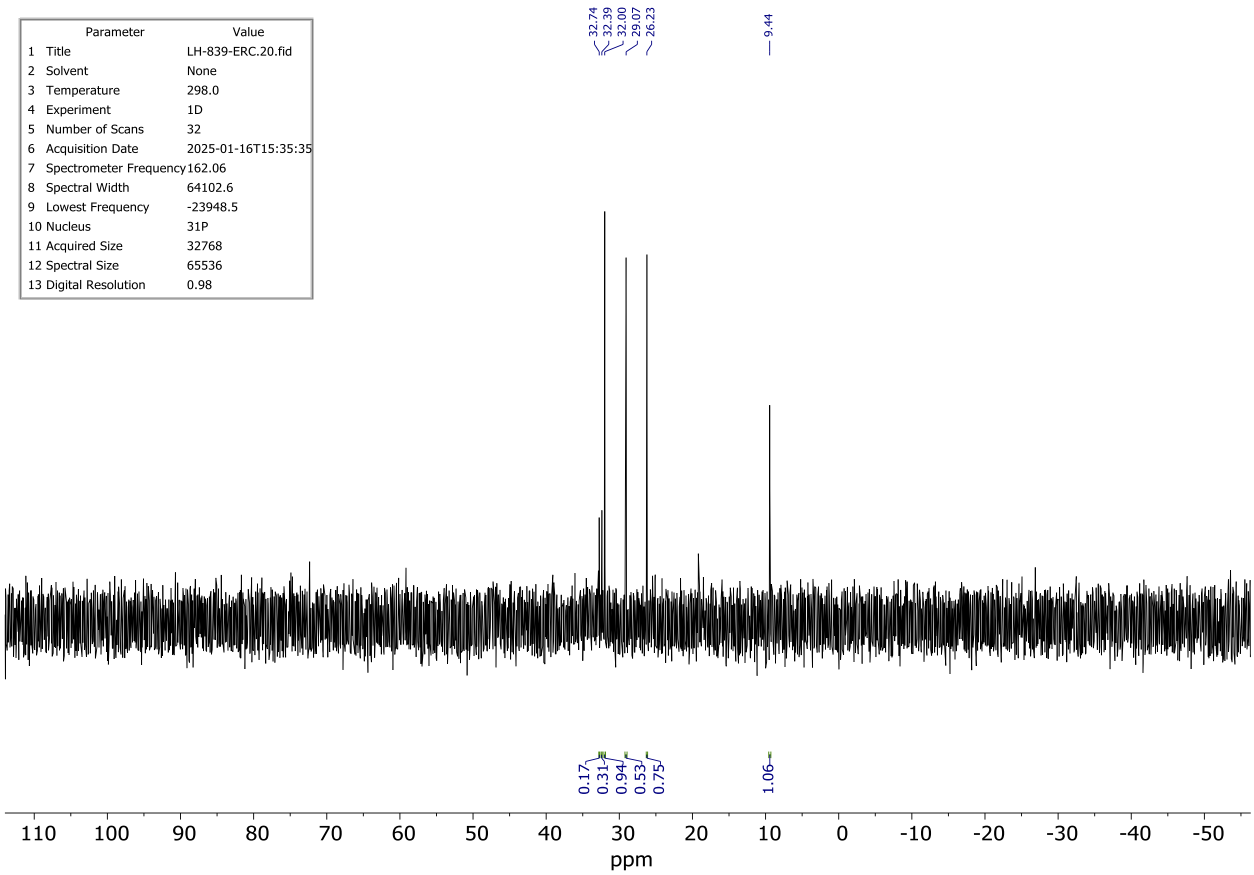

Figure S2. $^{31}P\{^1H\}$-NMR spectrum measured in toluene after heating **AYSi-2** to 50 °C for 1 d.

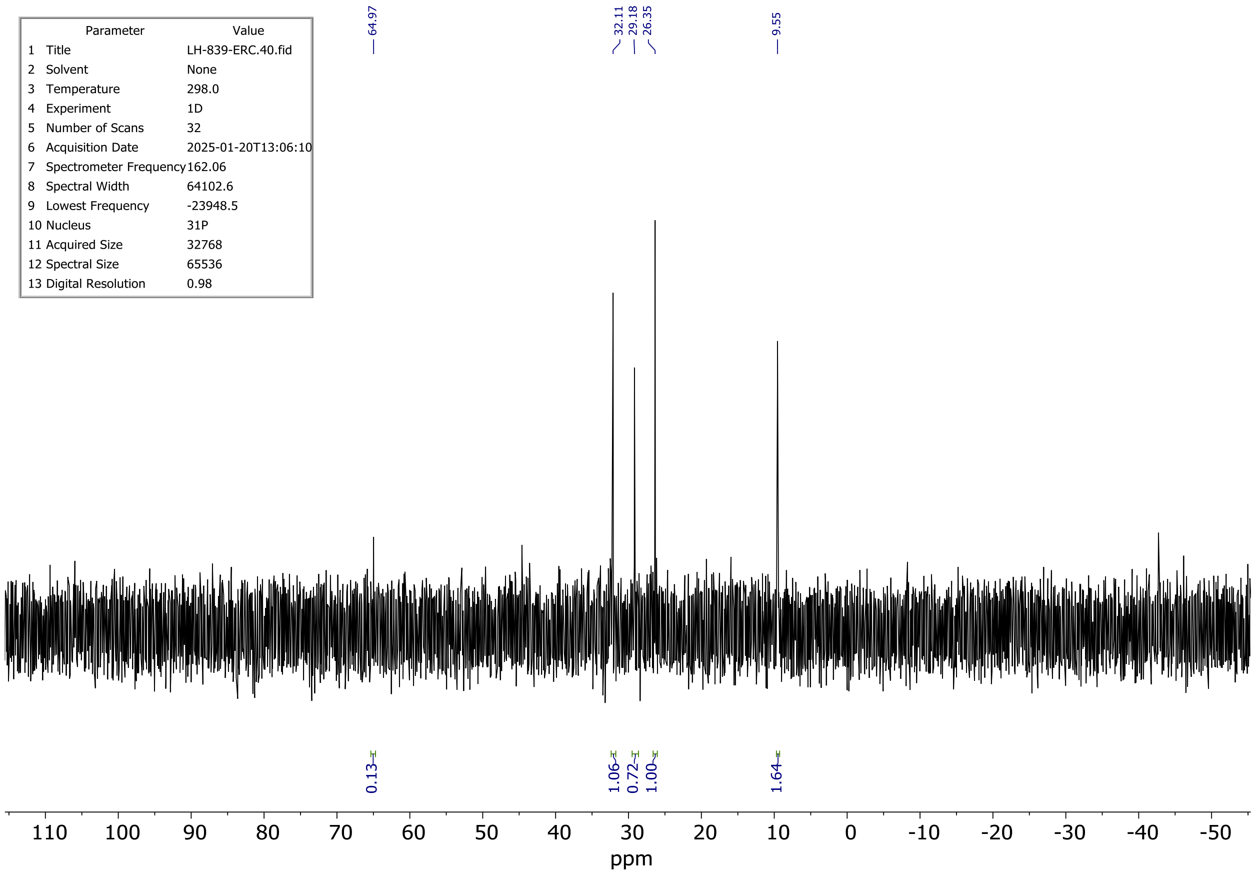

Figure S3. $^{31}P\{^1H\}$-NMR spectrum measured in toluene after heating **AYSi-2** to 50 °C for 5 d.

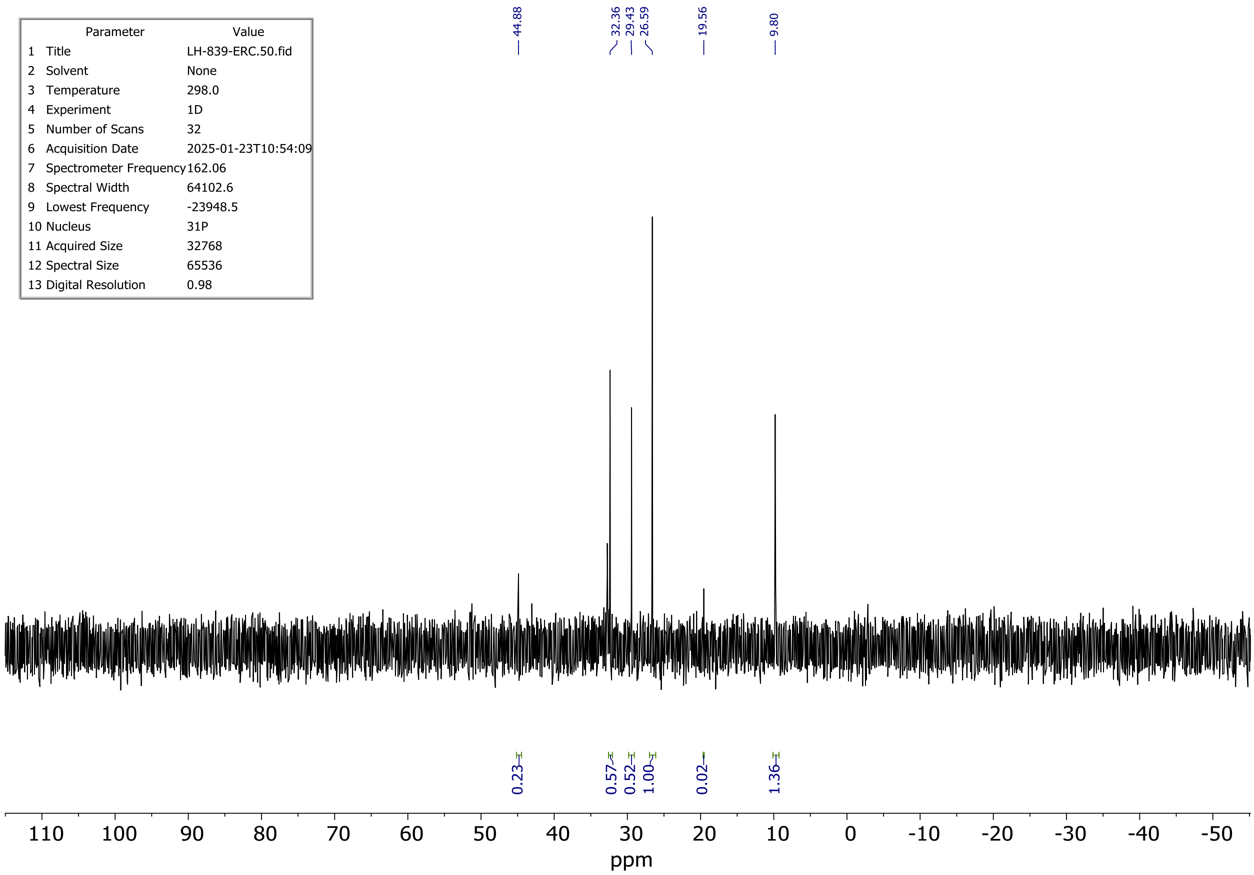

Figure S4. $^{31}P\{^{1}H\}$-NMR spectrum measured in toluene after heating **AYSi-2** to 50 °C for 1 week.

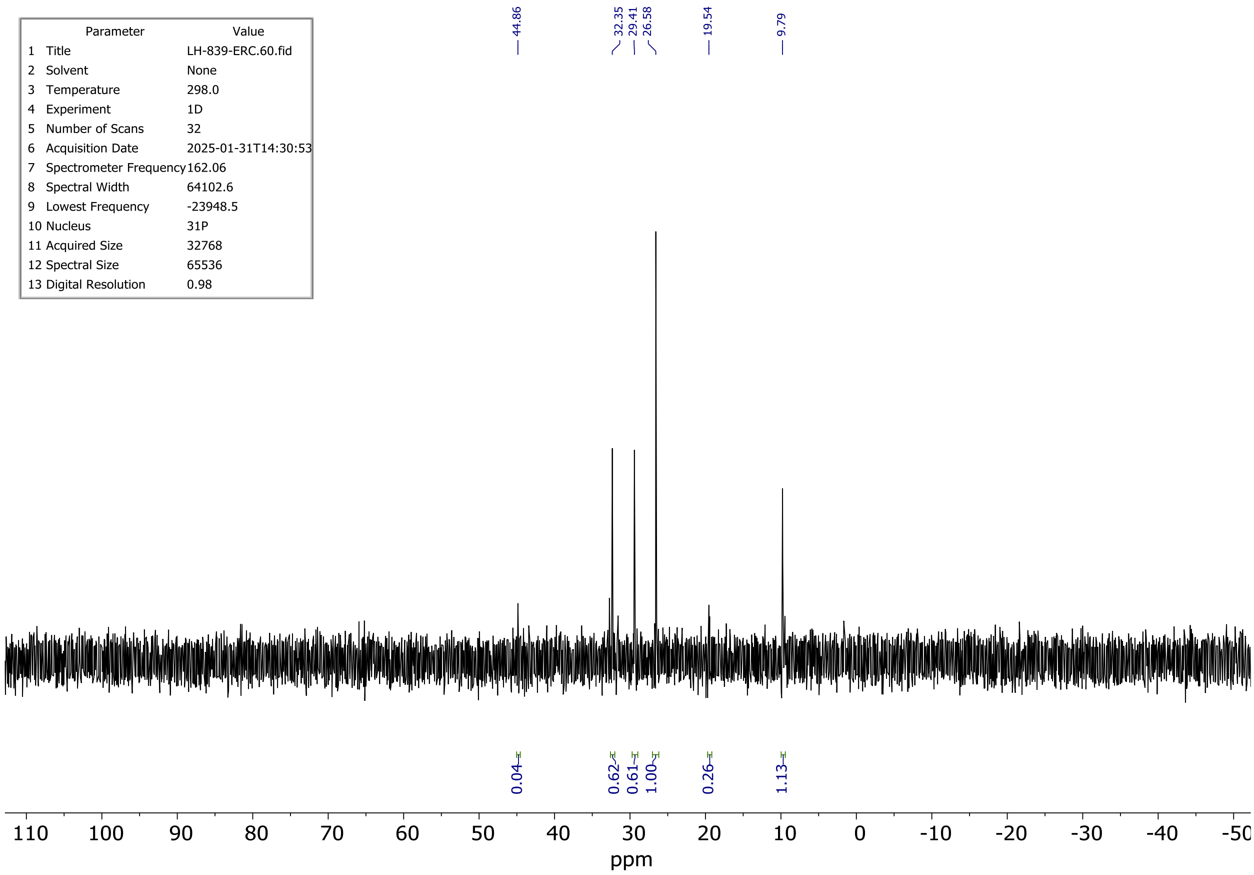

Figure S5. $^{31}P\{^{1}H\}$-NMR spectrum measured in toluene after heating **AYSi-2** to 50 °C for 2 weeks.

3.2 stability of silylene AYSi-3 in C_6D_6

Procedure for stability testing: **AYSi-2** (40 mg) was dissolved in 0.6 mL C_6D_6 in a J-Young NMR tube. The mixture was heated to 70 °C over a period of 4d. To follow the conversion to the C-H activation product, ^{31}P NMR spectra were measured after 1 d and after 4 d of heating. The spectra a presented below. To identify the formed product, a ^{1}H-NMR spectrum was recorded after 4 d of heating. As shown in Figure S8, the spectrum clearly indicates the formation of a Si-H moiety, with the proton appearing at 6.32 ppm featuring additional ^{29}Si satellites.

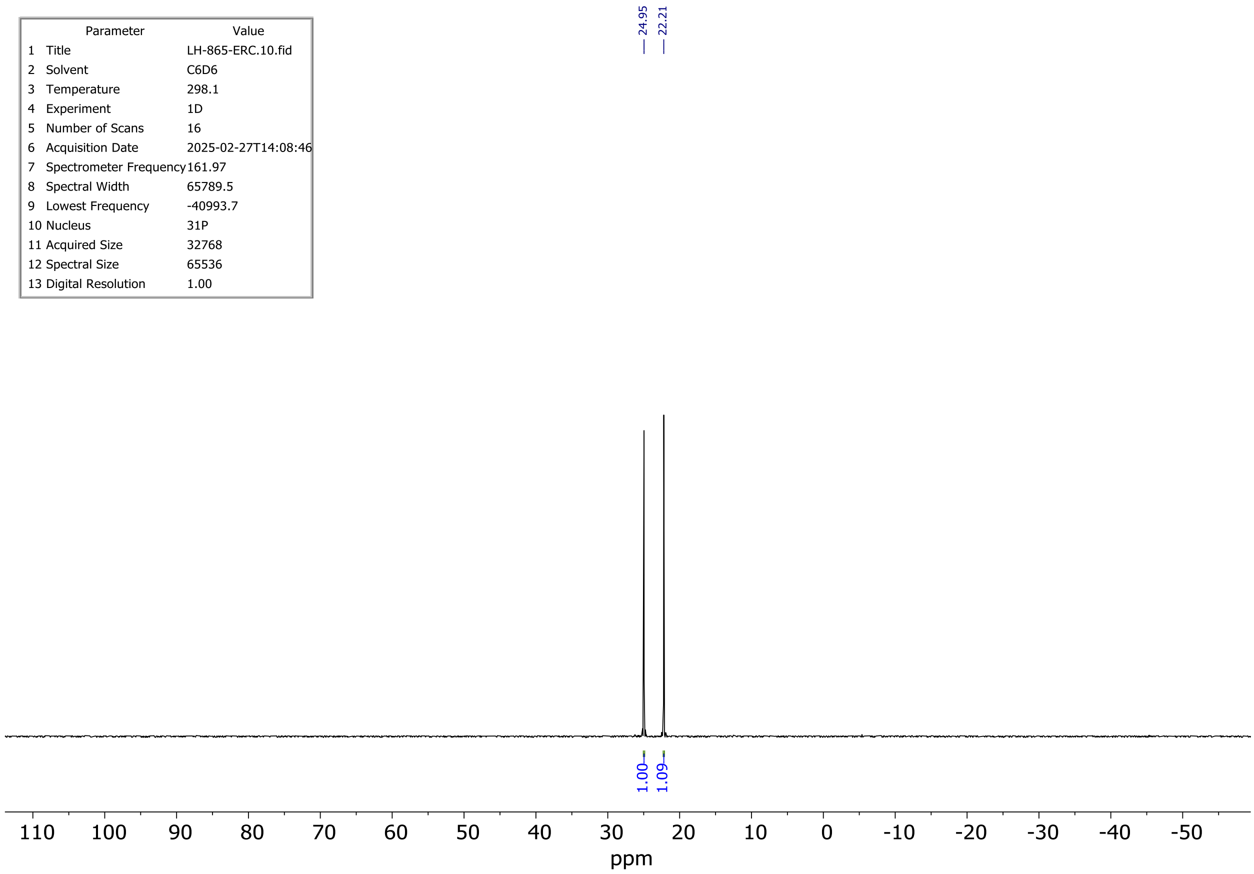

Figure S6. ^{31}P{^{1}H}-NMR spectrum measured in C_6D_6 after heating **AYSi-3** to 70 °C for 1 d.

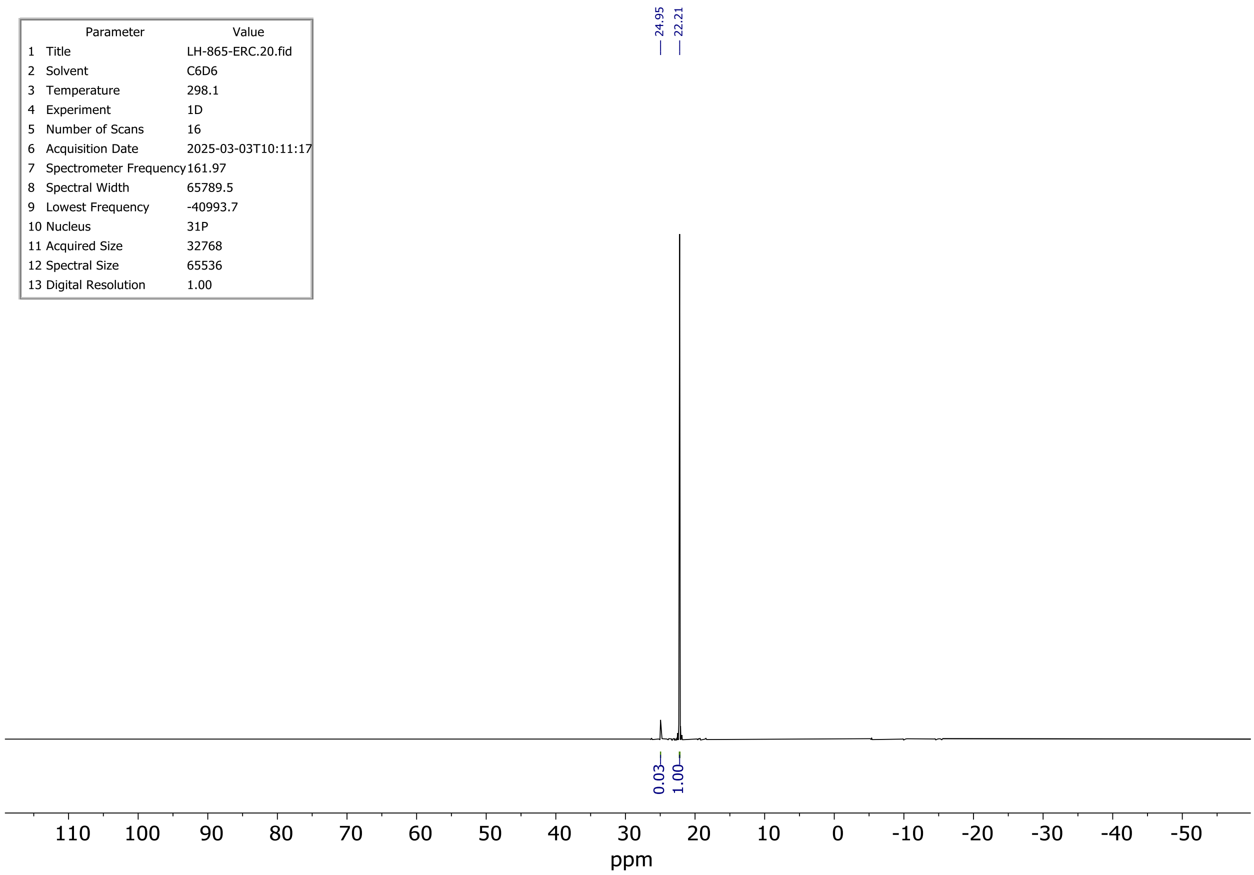

Figure S7. $^{31}P\{^1H\}$-NMR spectrum measured in C_6D_6 after heating **AYSi-3** to 70 °C for 4 d.

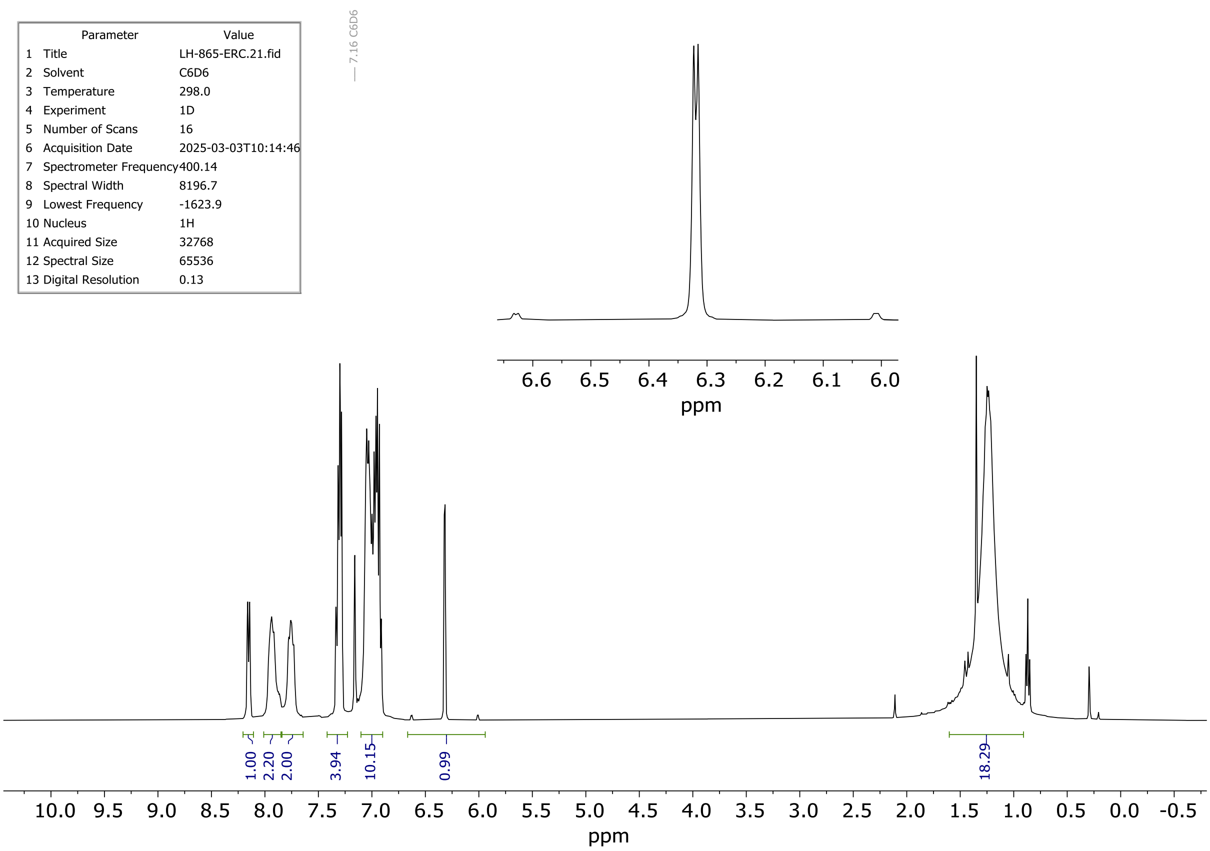

Figure S8. ^1H-NMR spectrum measured in C_6D_6 after heating **AYSi-3** to 70 °C for 4 d. The doublet at 6.32 ppm was identified as the Si-H signal due to the display of ^{29}Si-satellites, highlighted in the expansion of the spectrum.

3.3 Stability of silanone 6 in THF

Procedure for stability testing: A fresh batch of **6** was prepared by the exposure of solid **AYSi-2** to 1 bar of N_2O. The solid was taken up in dry THF-d_8 and added to an oven-dried J-Young tube. The freshly prepared sample already showed some ylide impurities (26.59 ppm) which was used as reference in the further studies. Fort stability test, the sample was kept in THF at room temperature and NMR spectra were recorded after 5 days, 1 month and 2 months.

Additionally, VT ^{1}H-NMR experiments were performed at 60 °C and -30 °C to investigate the interaction of the Cy-H *ipso*-protons with the oxygen atom at the silicon atom. While heating to 60 °C led to an improved resolution of the corresponding multiplett at 6.37 ppm, arguing for increased coalescence, cooling to –30 °C led to complete disappearance of the signal. This further corroborates with a weak interaction of the C-H moiety with the silanone.

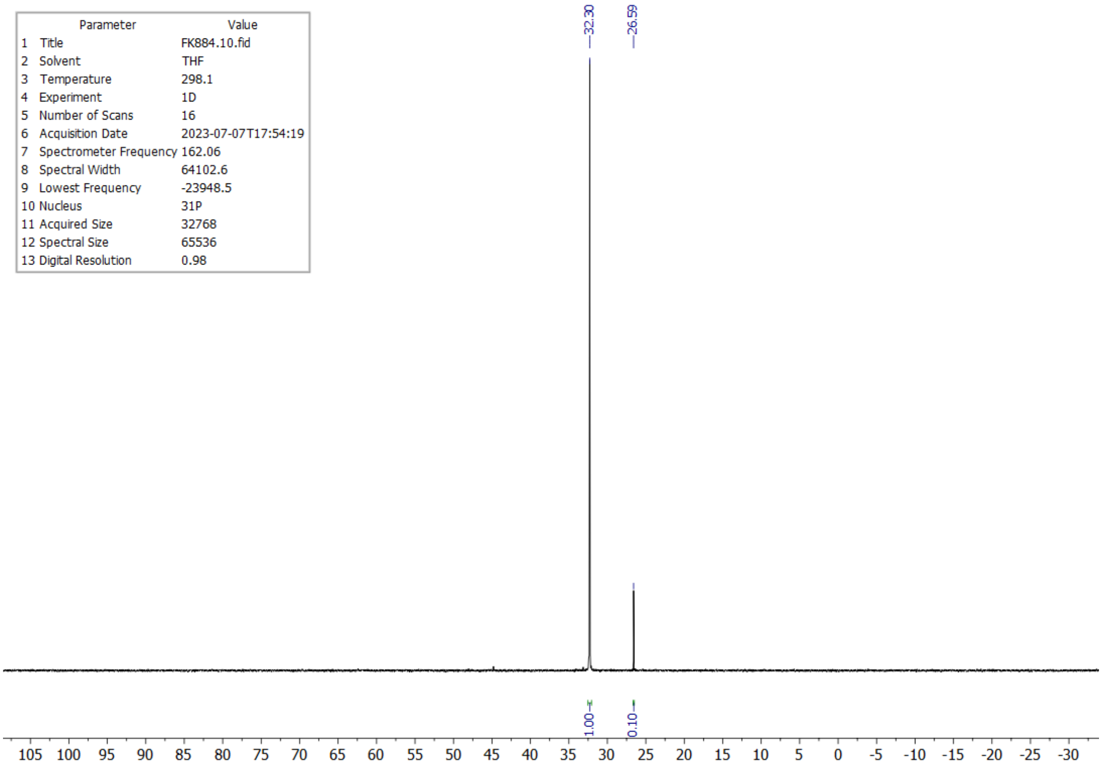

Figure S9. ^{31}P{^{1}H}-NMR spectrum of a freshly prepared sample of **6**. The peak at 26.59 ppm corresponds to the ylide which serves as an internal standard.

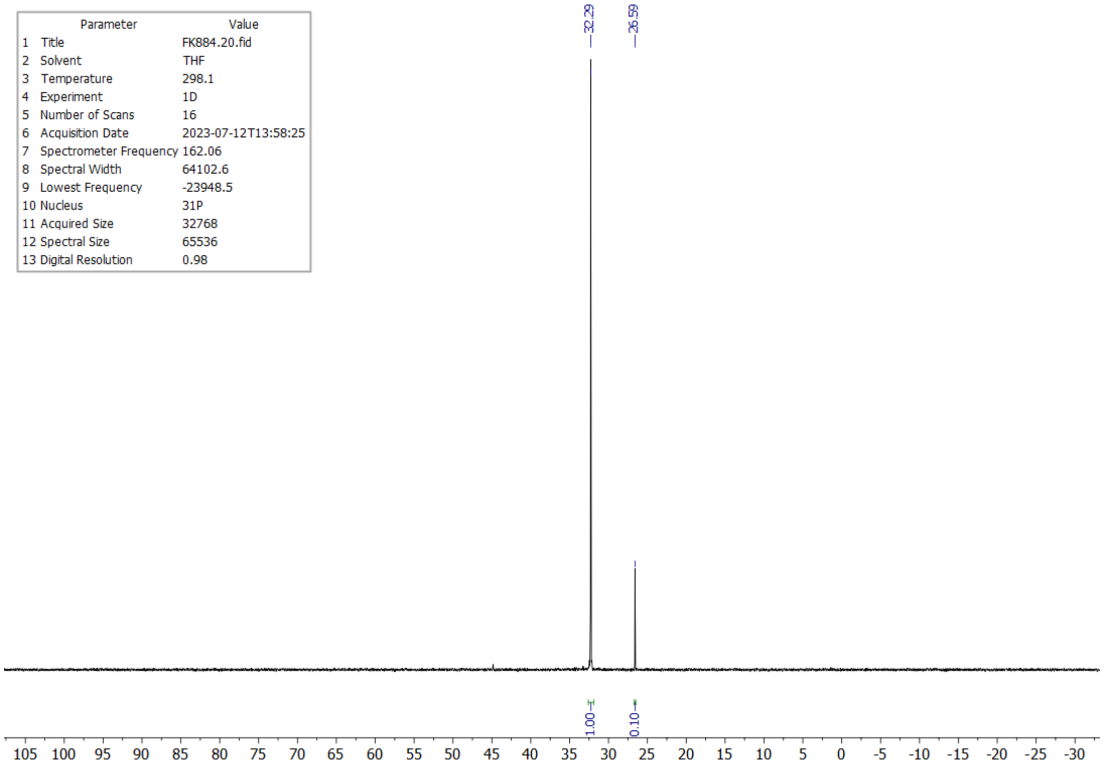

Figure S10. $^{31}P\{^{1}H\}$-NMR spectrum of **6** in THF solution measured after <u>5 days</u> in the J-Young tube.

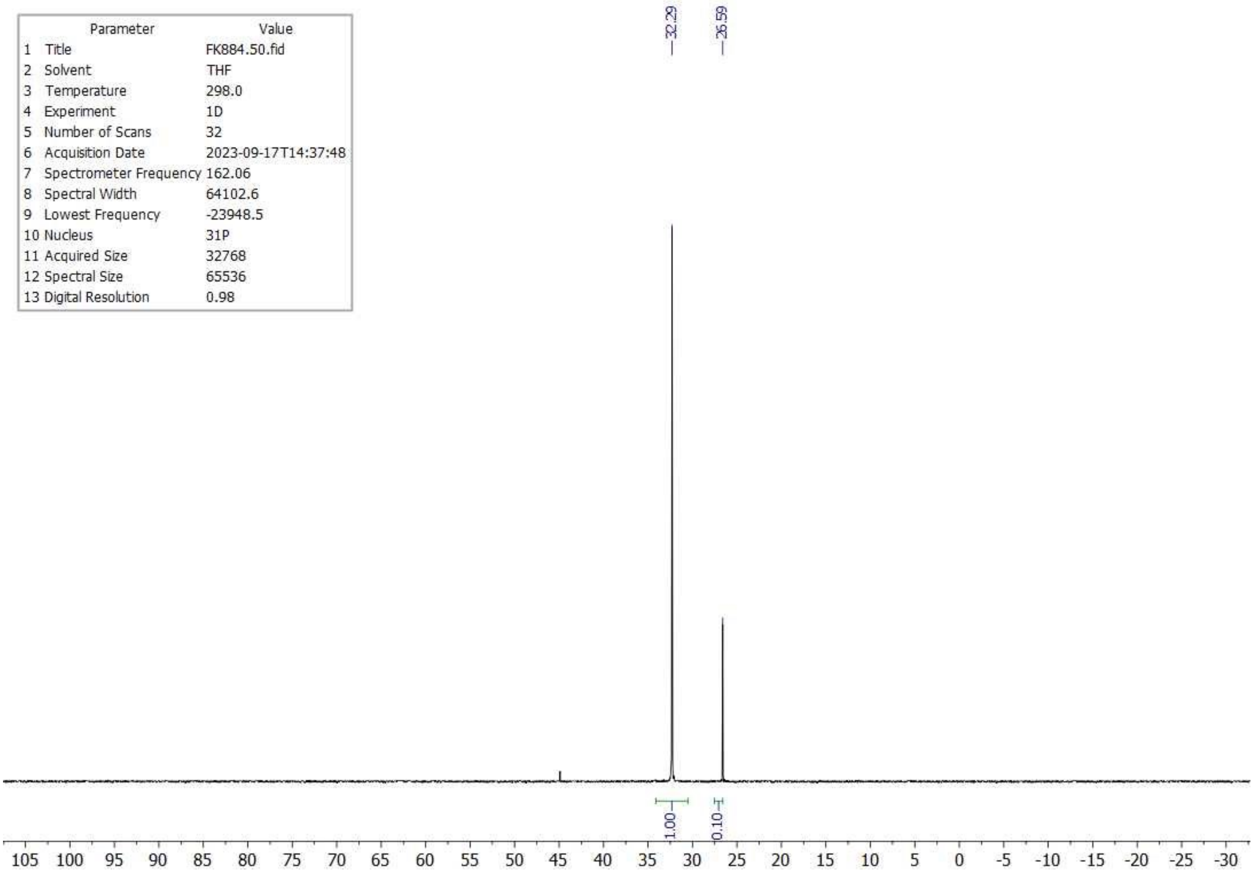

Figure S11. $^{31}P\{^{1}H\}$-NMR spectrum of **6** in THF solution measured after <u>2 months</u> in the J-Young tube.

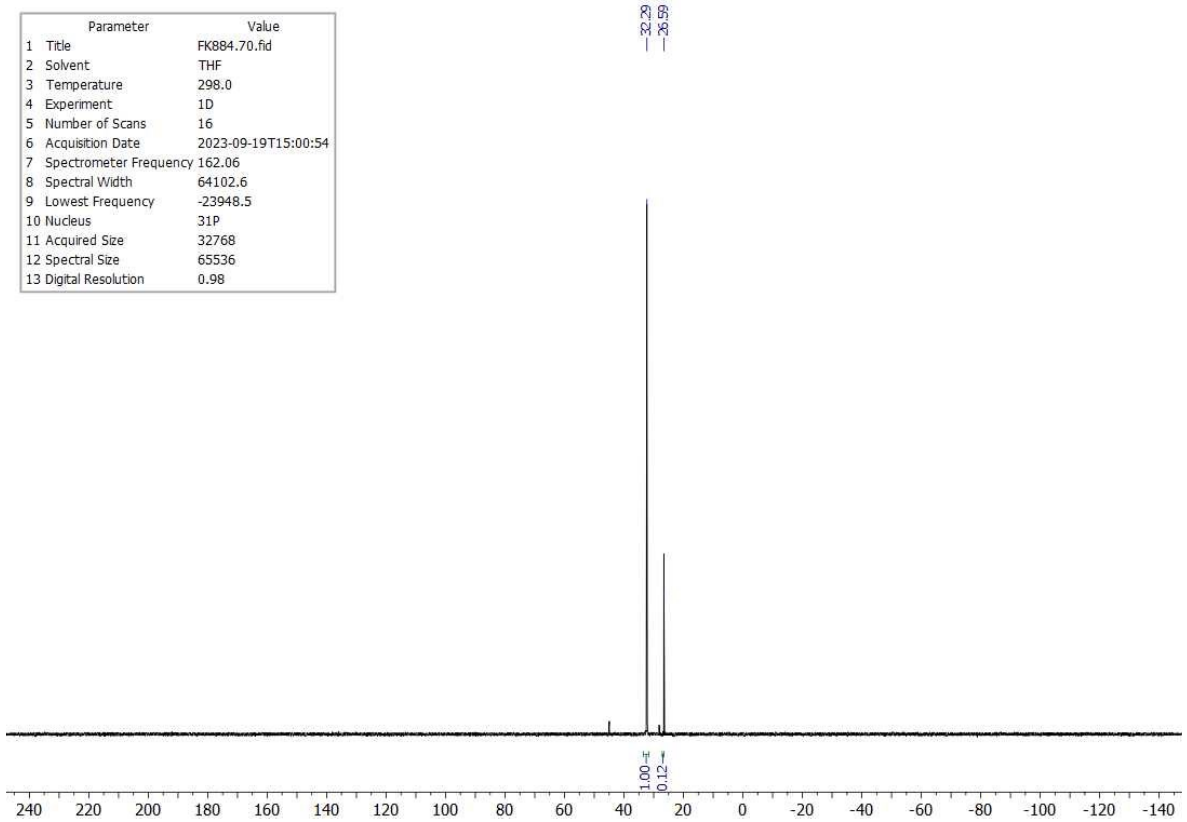

Figure S12. $^{31}P\{^1H\}$-NMR spectrum of **6** after 2 months at RT and 1d at 70 °C in the J-Young tube.

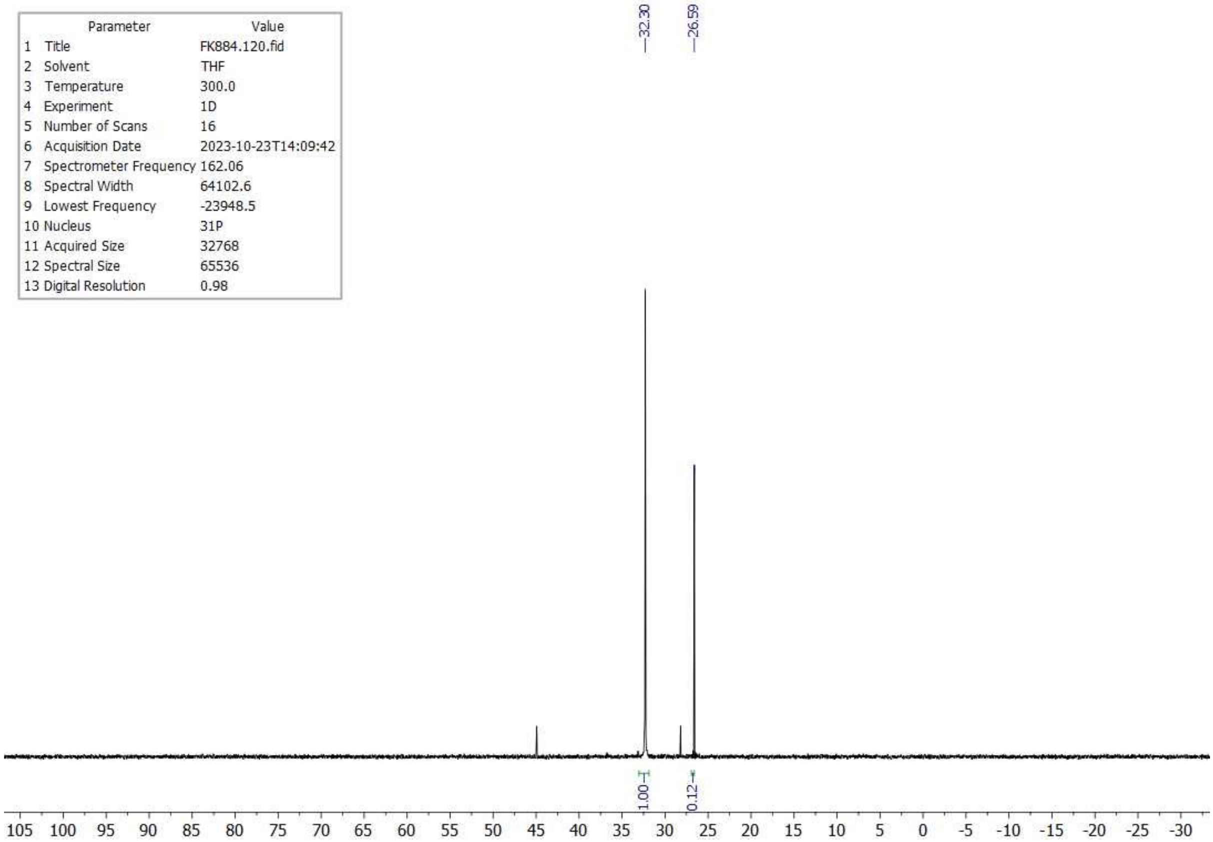

Figure S13. $^{31}P\{^1H\}$-NMR spectrum of **6** after 2 months at RT and 1 month at 70 °C in the J-Young tube.

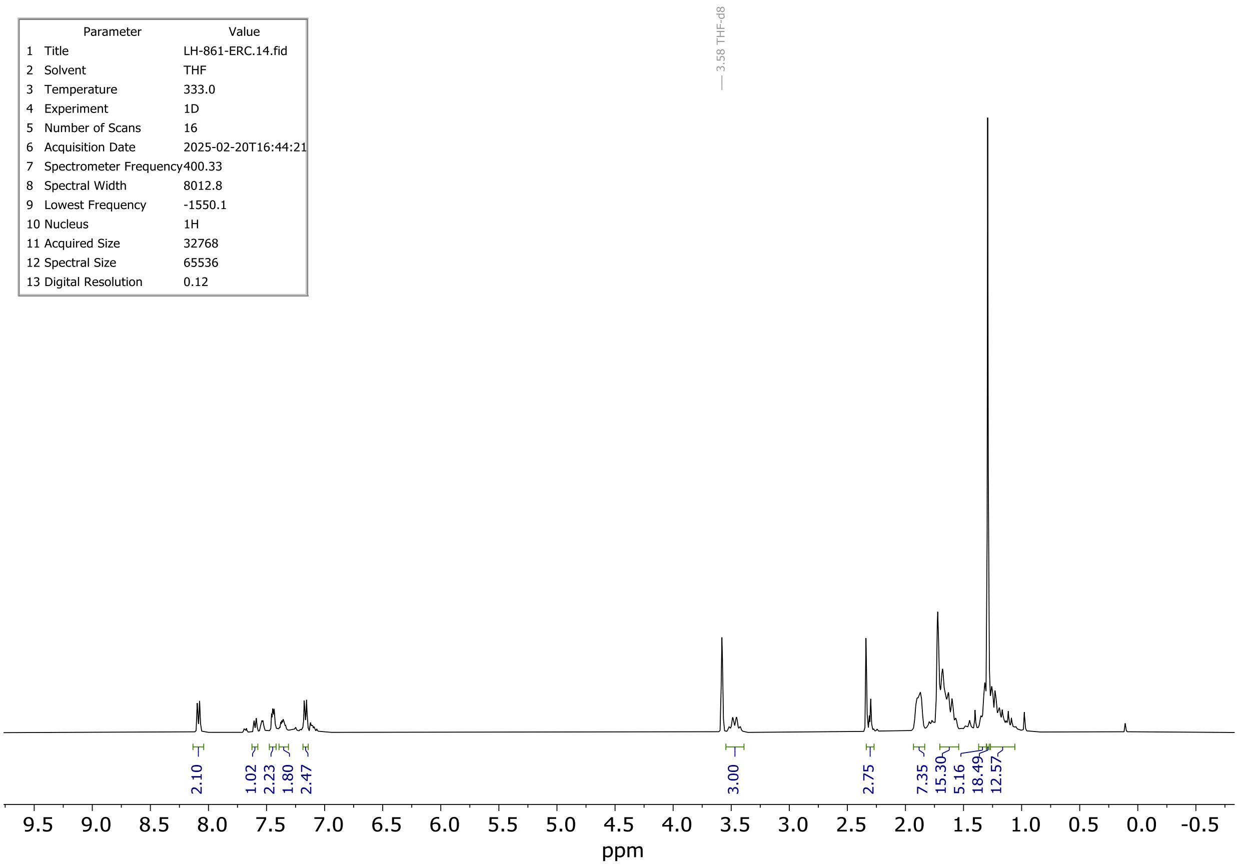

Figure S14. ^{1}H-NMR spectrum of **6** measured in d_8-THF <u>at 333K</u>.

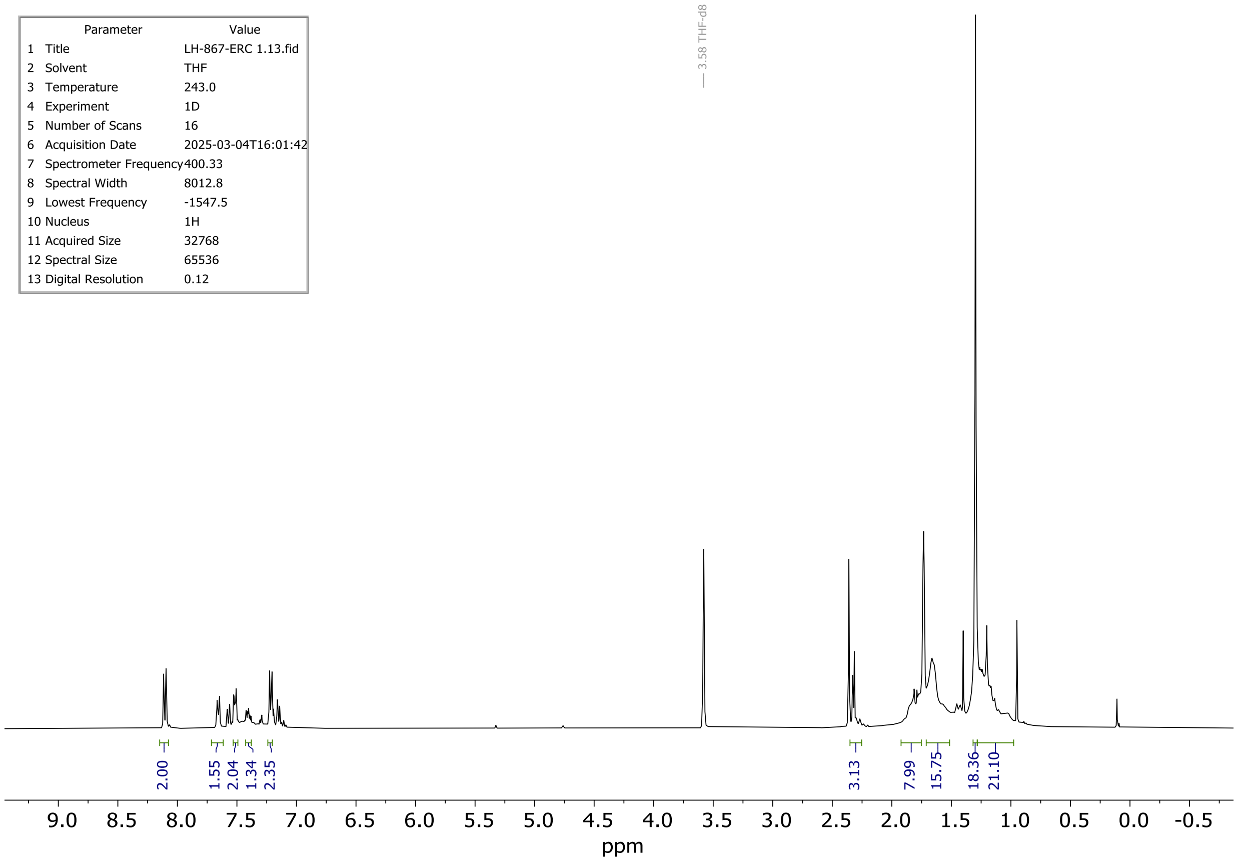

Figure S15. ^{1}H-NMR spectrum of **6** measured in d_8-THF <u>at 243K</u>.

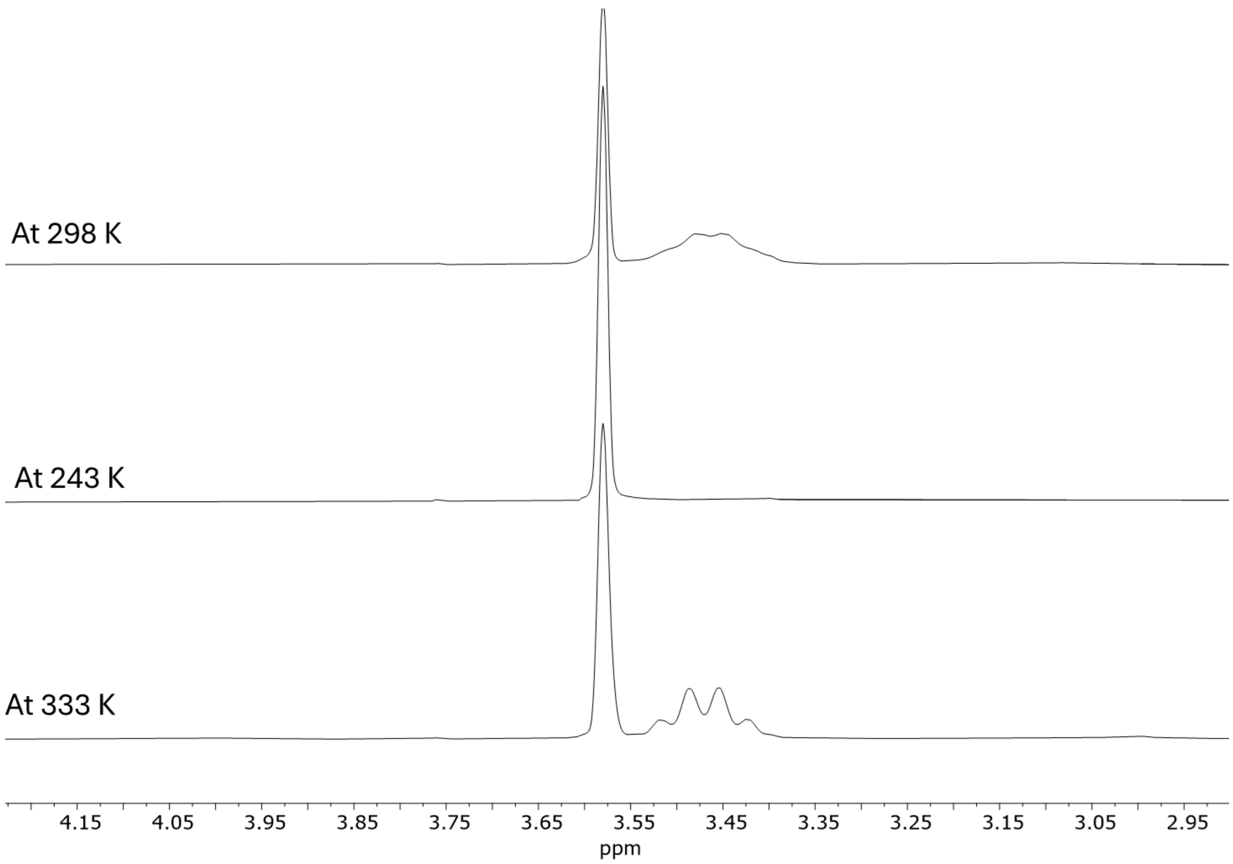

Figure S16. Excerpt from the stacked ^{1}H-NMR spectra of **6** measured in d_8-THF at 298 K (top), 243 K (middle) and 333 K (bottom), highlighting the temperature dependent behavior of the Cy-H *ipso*-signal.

4 Reactivity of silanone 6 towards CO_2

In a J-Young NMR tube, 20 mg (28.3 µmol, 1eq) **AYSi-2** were dissolved in d_8-THF (instead of toluene, see above) under an argon atmosphere, which was subsequently exchanged to 1.5 bar of N_2O, leading to immediate decolorization of the yellow solution. After shaking the tube for 4 h at RT, the atmosphere was again exchanged to 2 bar of CO_2. The next day the ^{1}H NMR spectrum displayed a slightly different shift pattern compared to silanone **6**, especially the Cy-H ipso signal was significantly altered by shifting from 3.46 ppm for **6** to 3.09 ppm in the obtained spectrum. This raised the question for the formation of a possible carbonate complex formation analogous to **8**. While the ^{13}C-NMR spectrum did not show the clear appearance of a C=O signal, the IR spectrum of the dried reaction mixture showed a band of low intensity at 1773 cm^{-1}, which could be interpreted as originating from a C=O stretching mode. In the IR spectrums of **AYSi-2** and **6** no band was observed at a comparable wavenumber. XRD analysis of crystals obtained from the reaction mixture only showed the structure of the silanone. However, the formation of the **AYSi-2** carbonate complex cannot be entirely ruled out but is likely to occur only as a minor product.

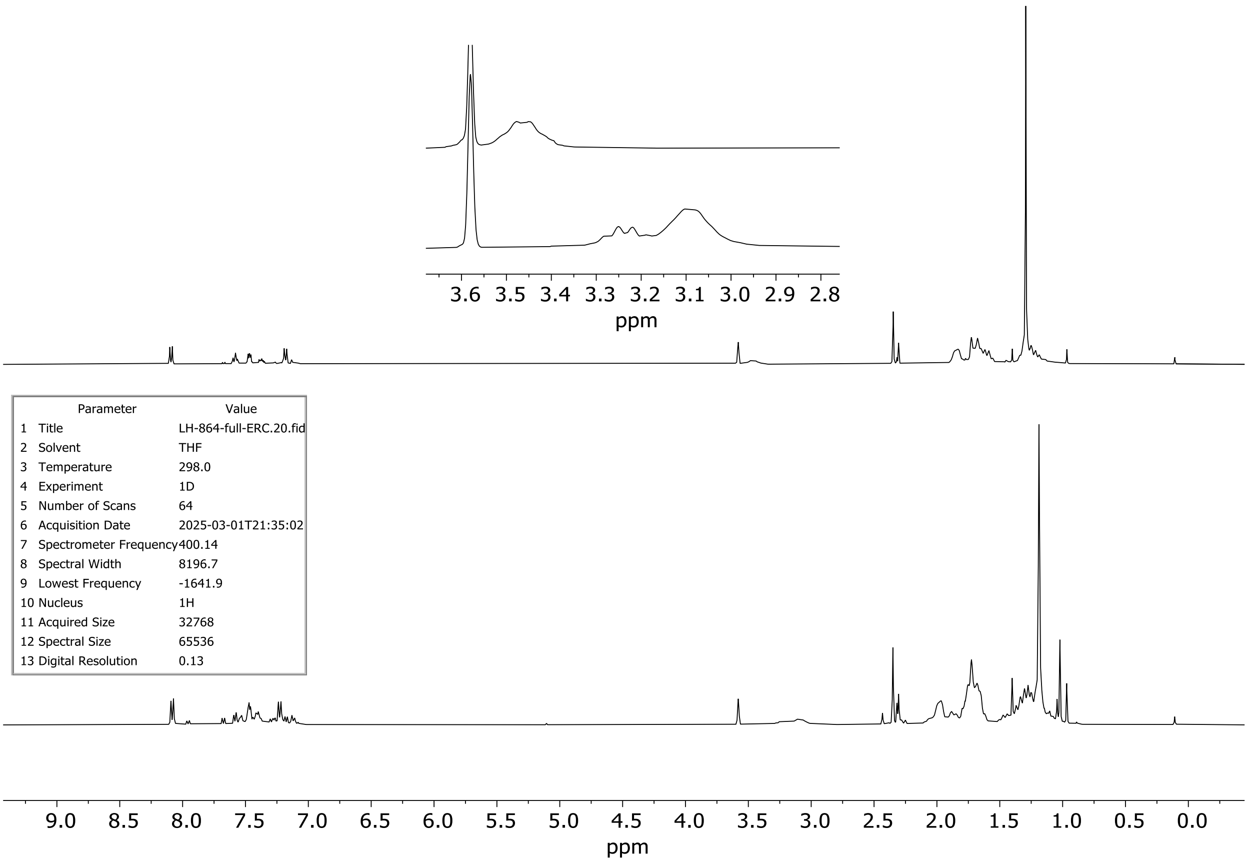

Figure S17. Stacked ^{1}H-NMR spectra of silanone **6** (top) and the CO_2 reaction mixture measured in d_8-THF. Highlighted in the expansion of the spectrum is the shifting of the Cy-H ipso signal.

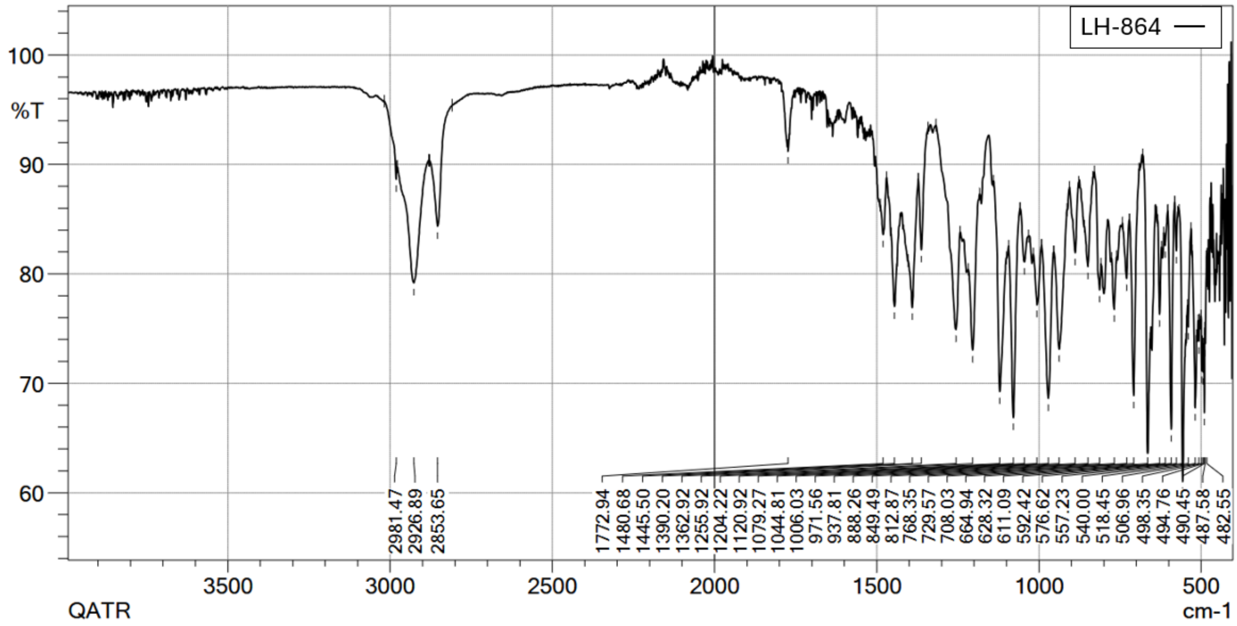

Figure S18. IR spectrum of the dried mixture from the reaction of **6** with CO_2.

5 NMR and IR Spectra of the Isolated Compounds

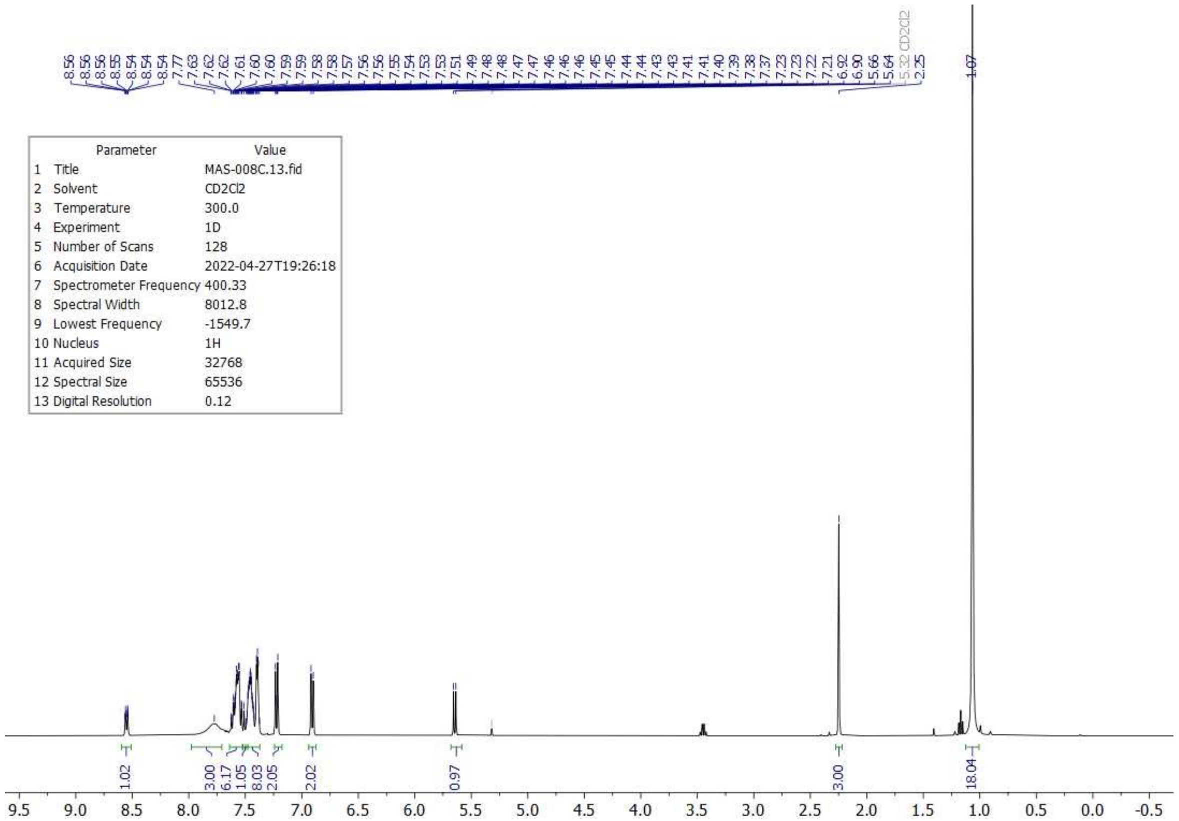

Figure S19. ^{1}H-NMR spectrum of **2** in CD_2Cl_2. The peaks at 3.43 ppm & 1.15 ppm correspond to residual diethyl ether.

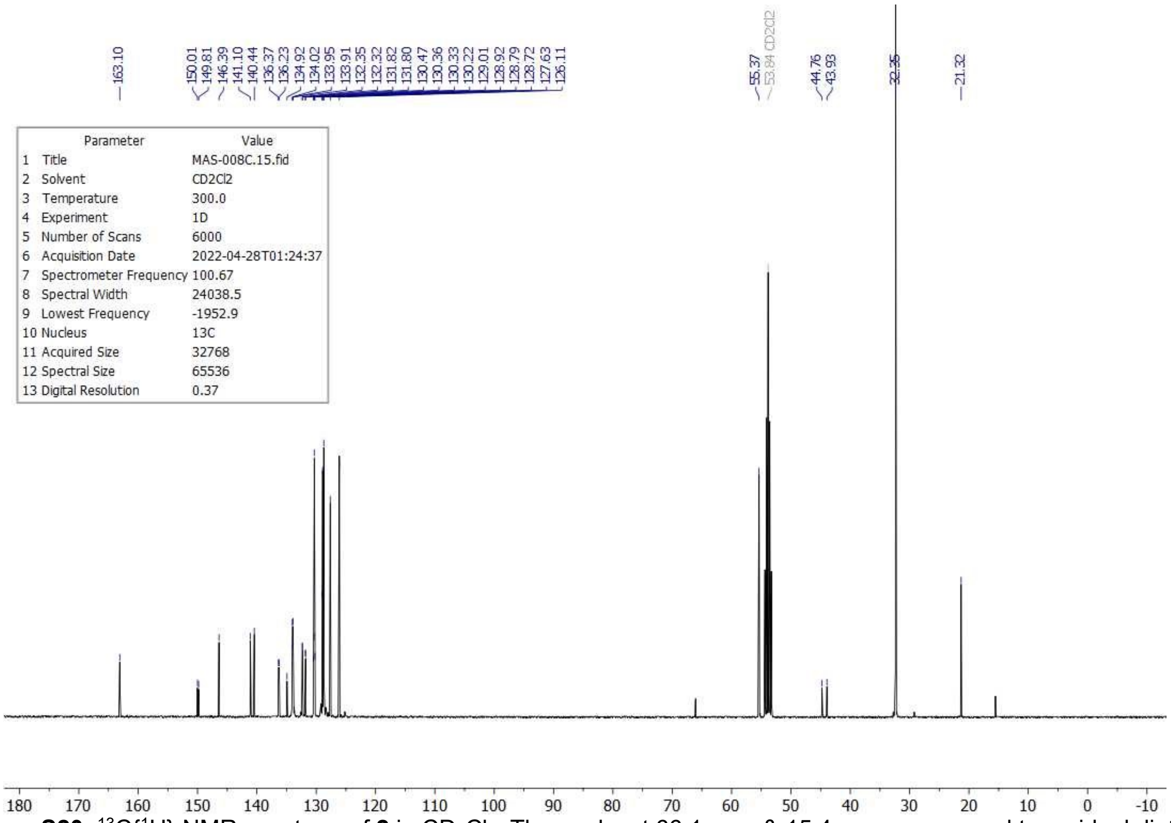

Figure S20. $^{13}C\{^{1}H\}$-NMR spectrum of **2** in CD_2Cl_2. The peaks at 66.1 ppm & 15.4 ppm correspond to residual diethyl ether.

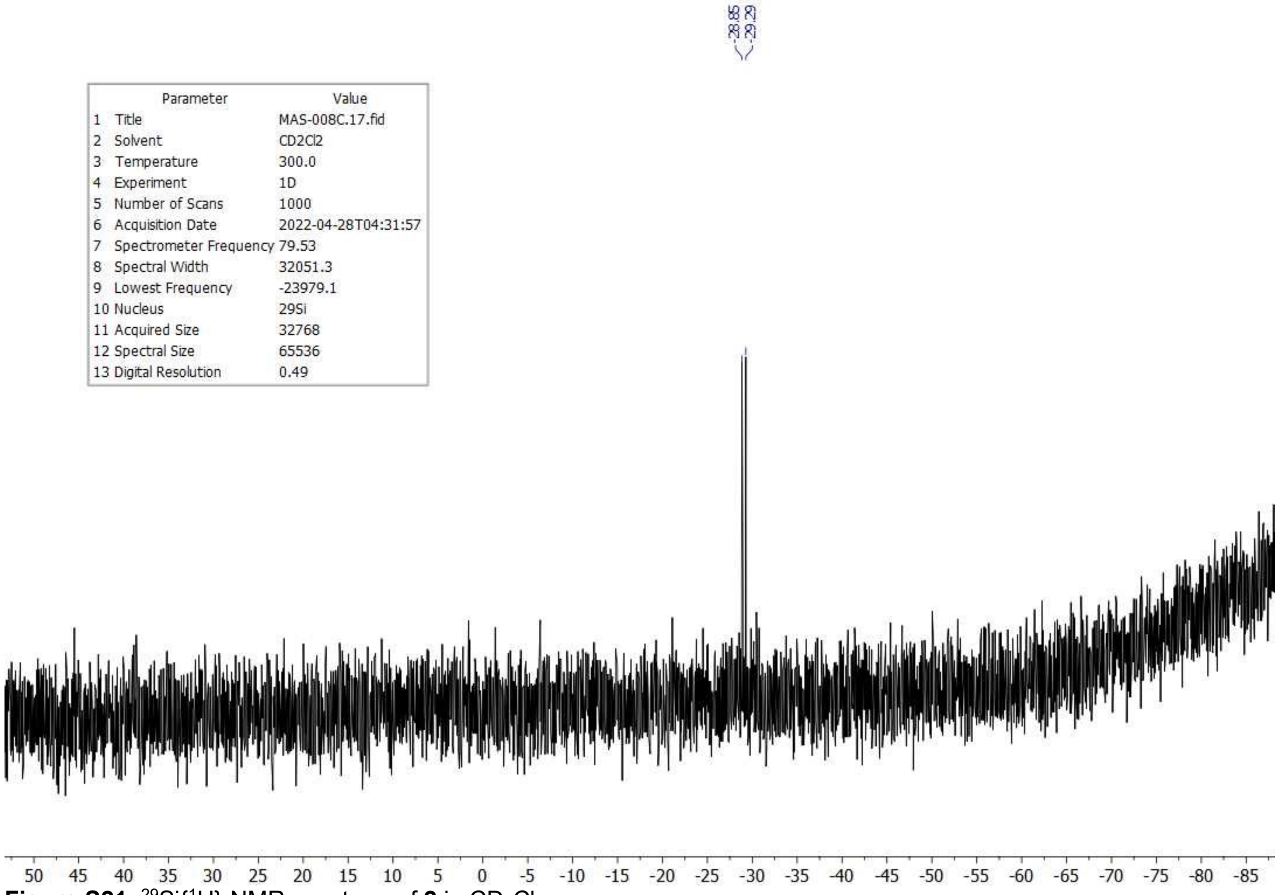

Figure S21. $^{29}Si\{^{1}H\}$-NMR spectrum of **2** in CD_2Cl_2.

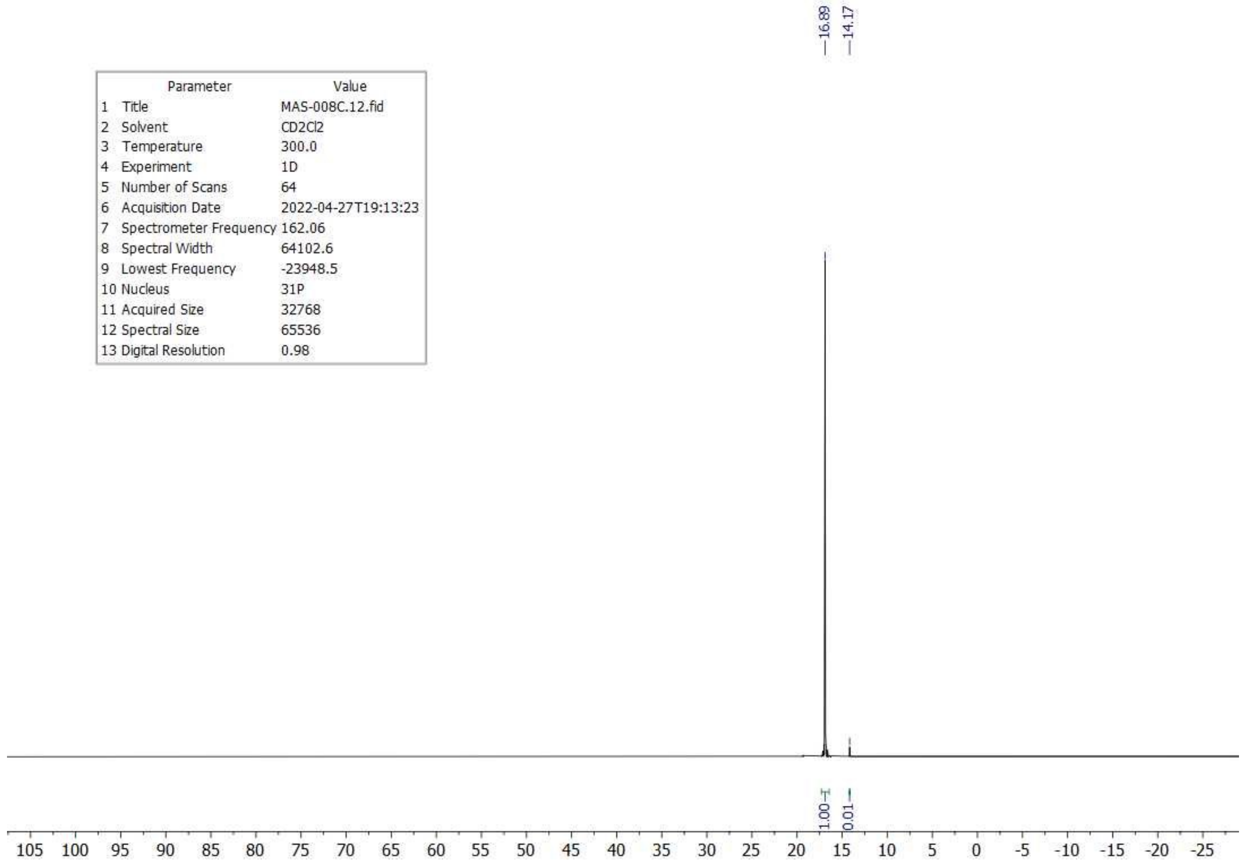

Figure S22. $^{31}P\{^{1}H\}$-NMR spectrum of **2** in C_6D_6. The peak at 14.2 ppm corresponds to the ylide.

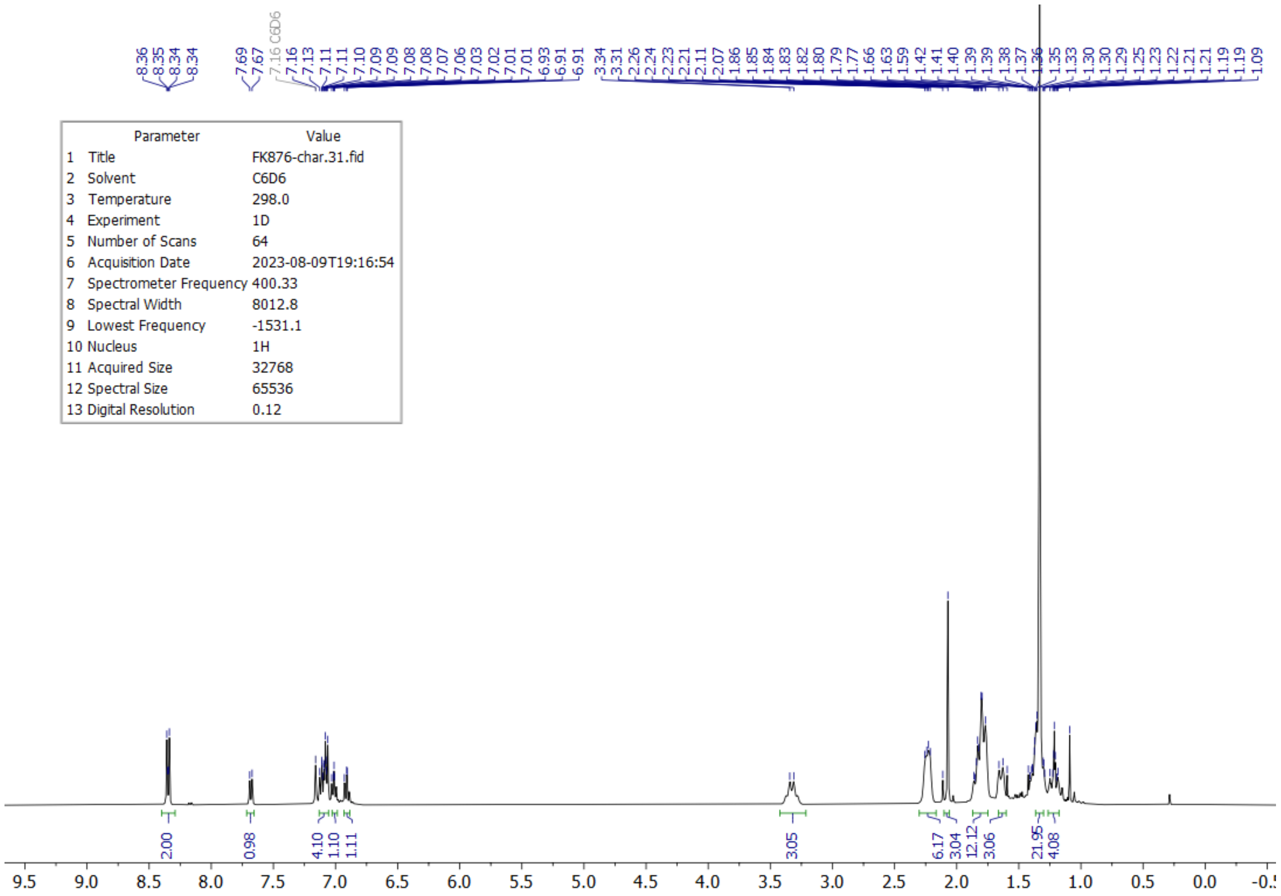

Figure S23. ^{1}H-NMR spectrum of **AYSi-2** in C_6D_6.

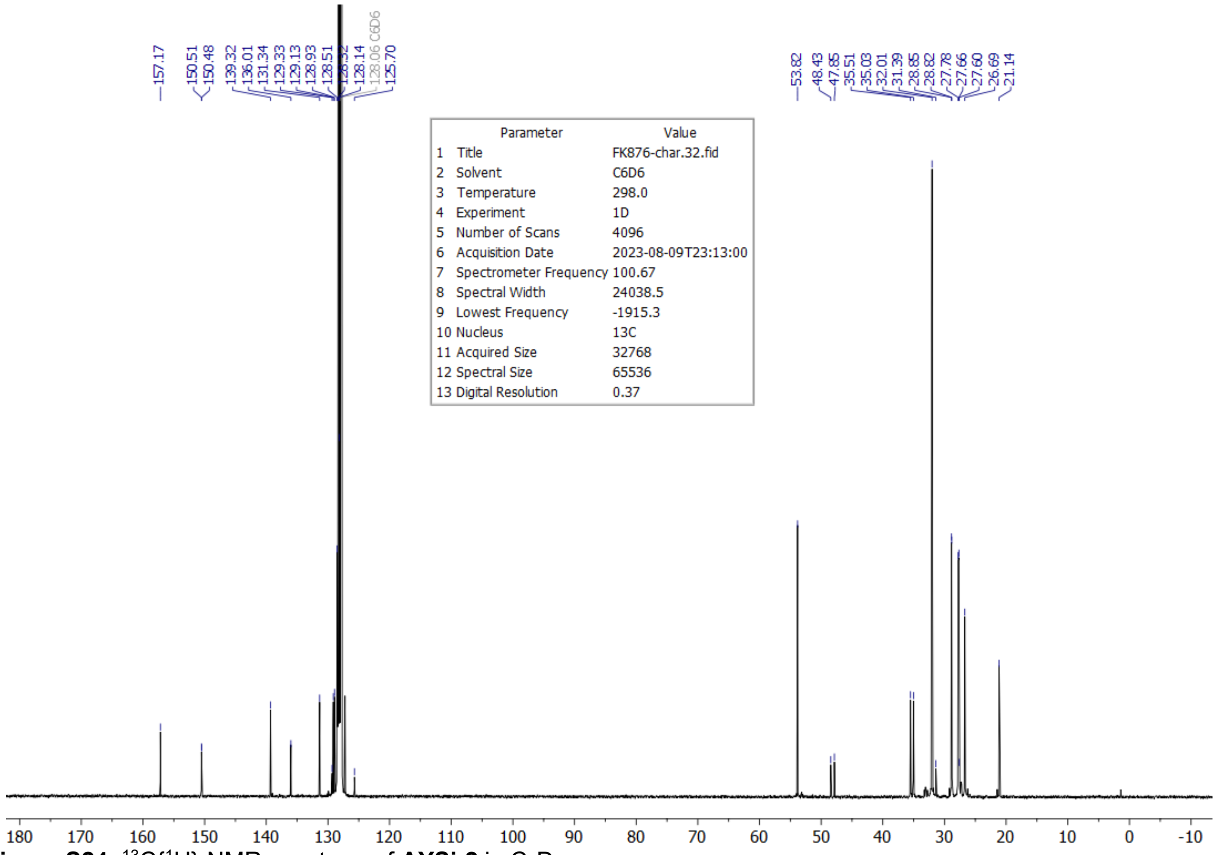

Figure S24. ^{13}C{^{1}H}-NMR spectrum of **AYSi-2** in C_6D_6.

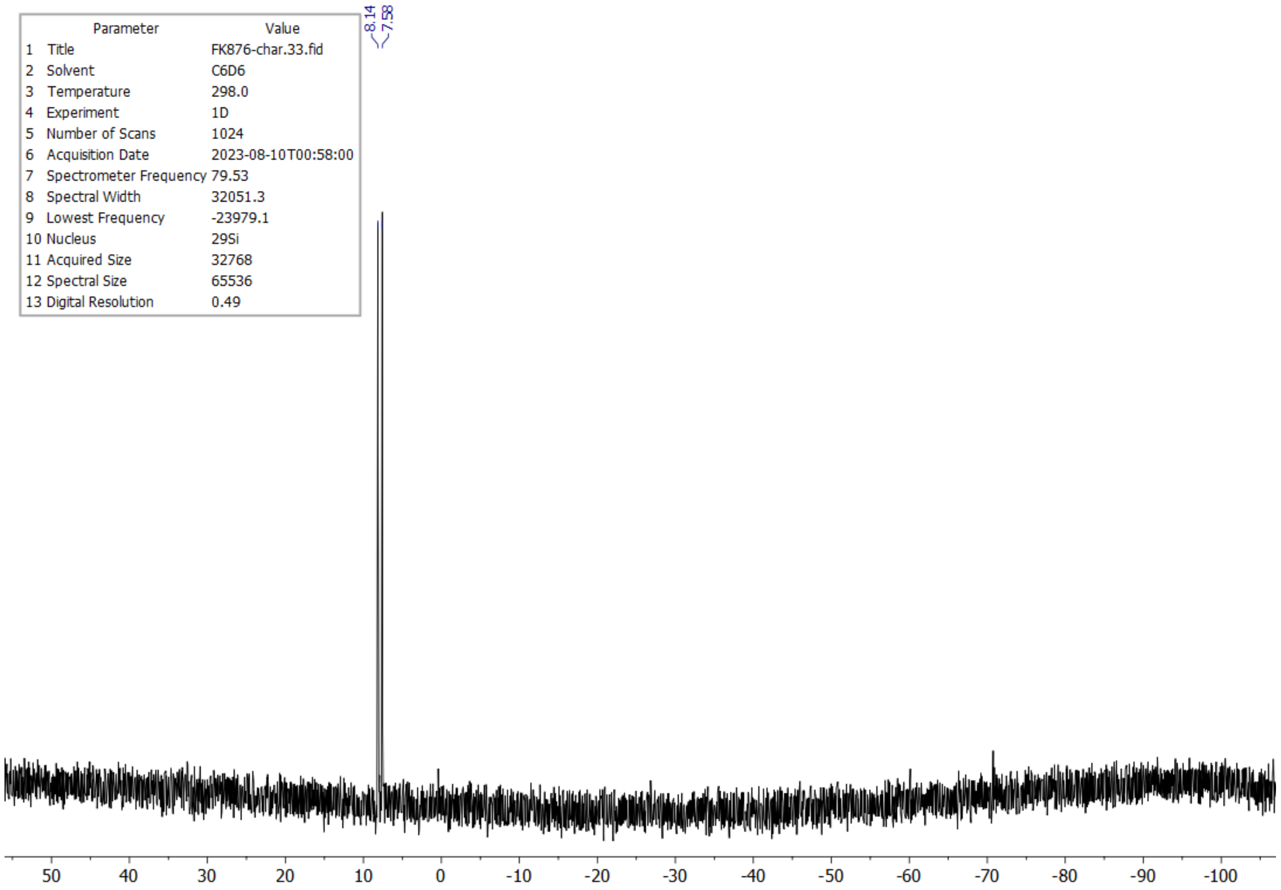

Figure S25. $^{29}Si\{^{1}H\}$-NMR spectrum of **AYSi-2** in C_6D_6.

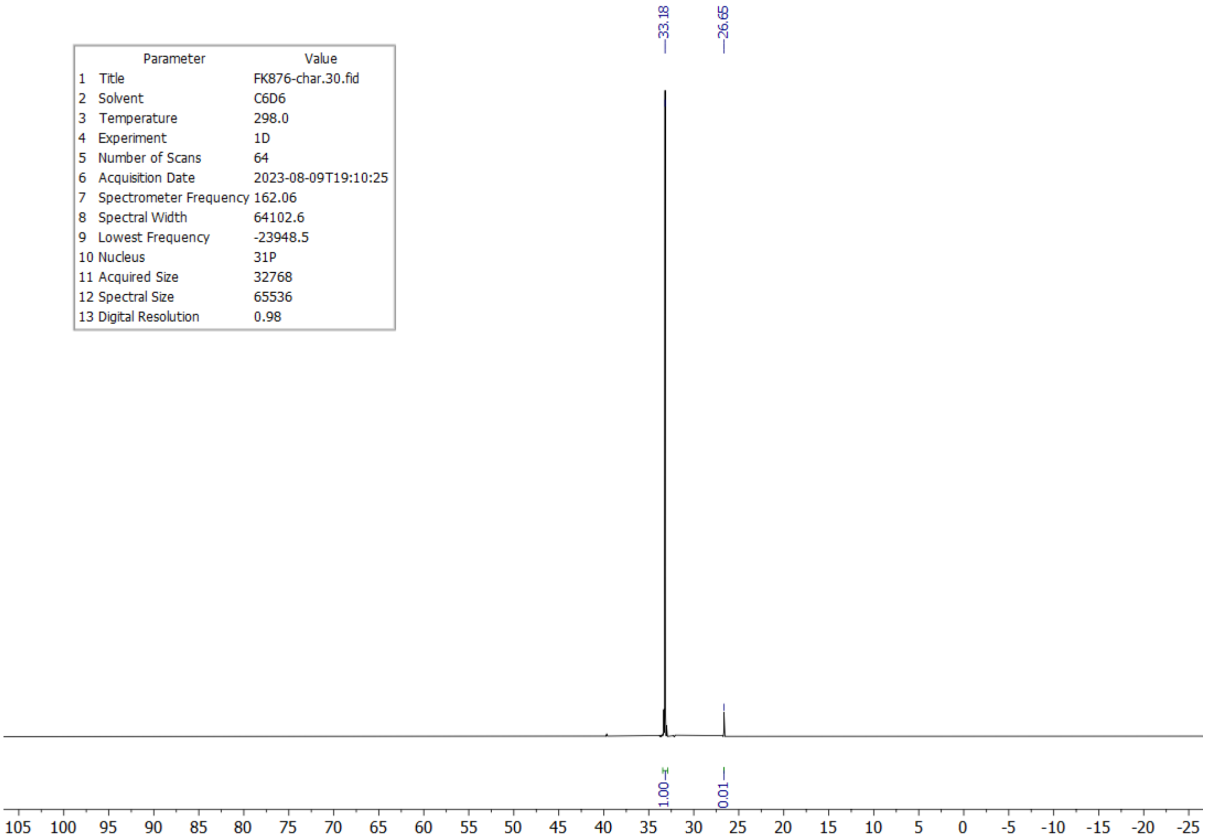

Figure S26. $^{31}P\{^{1}H\}$-NMR spectrum of **AYSi-2** in C_6D_6. The peak at 26.6 ppm corresponds to the ylide.

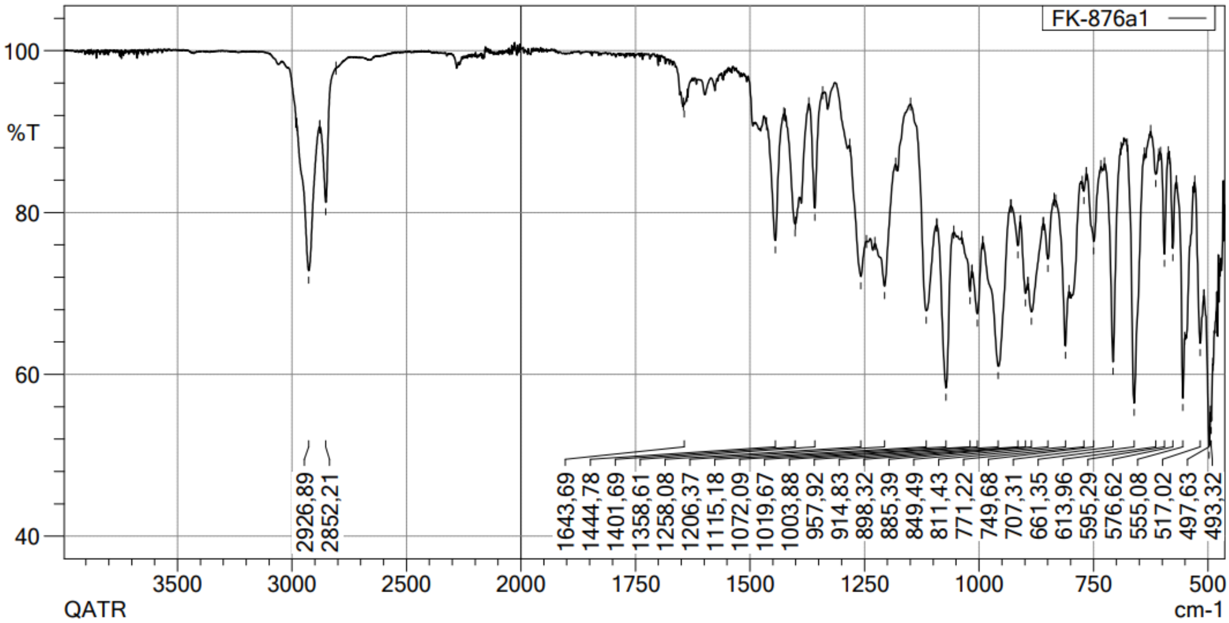

Figure S27. IR spectrum of solid **AYSi-2**.

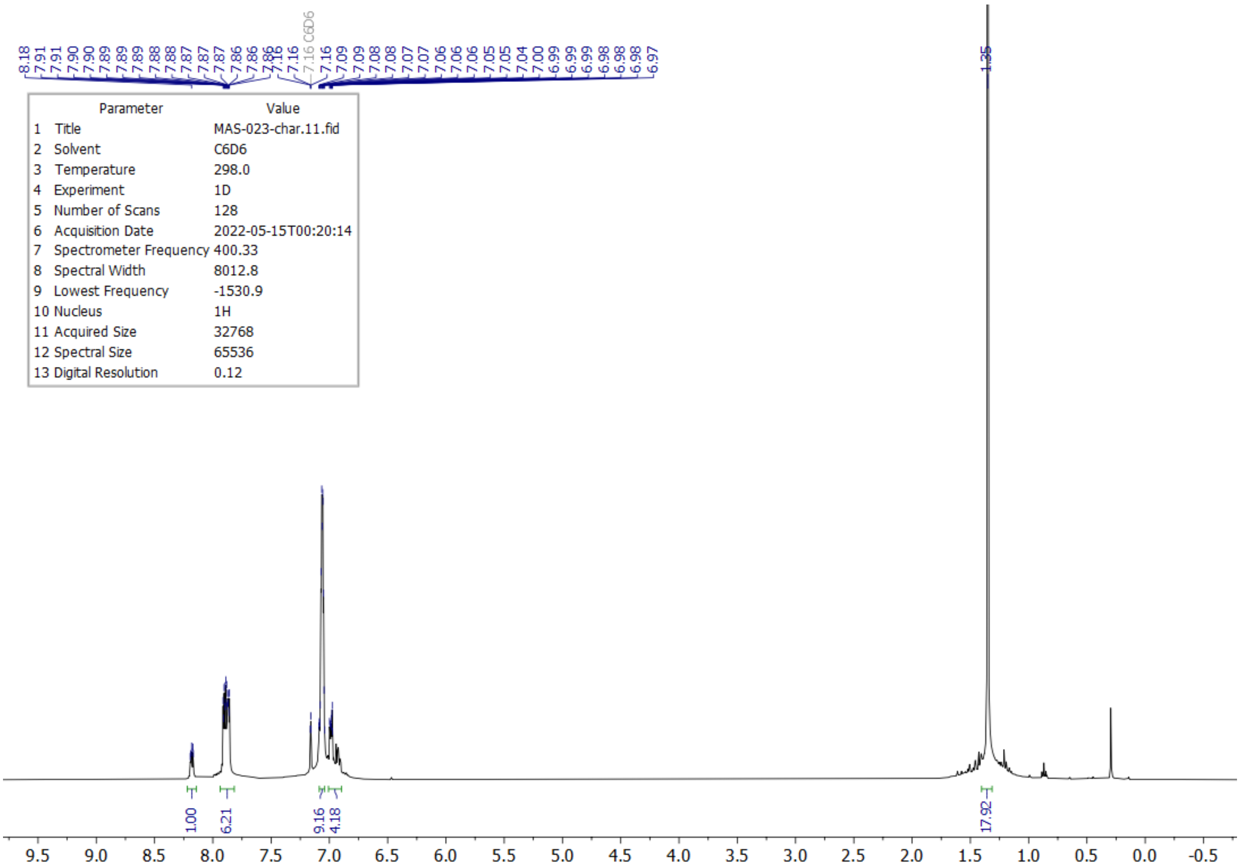

Figure S28. ^{1}H-NMR spectrum of **AYSi-3** in C_6D_6. The peaks at 1.23 ppm and 0.87 ppm correspond to residual pentane.

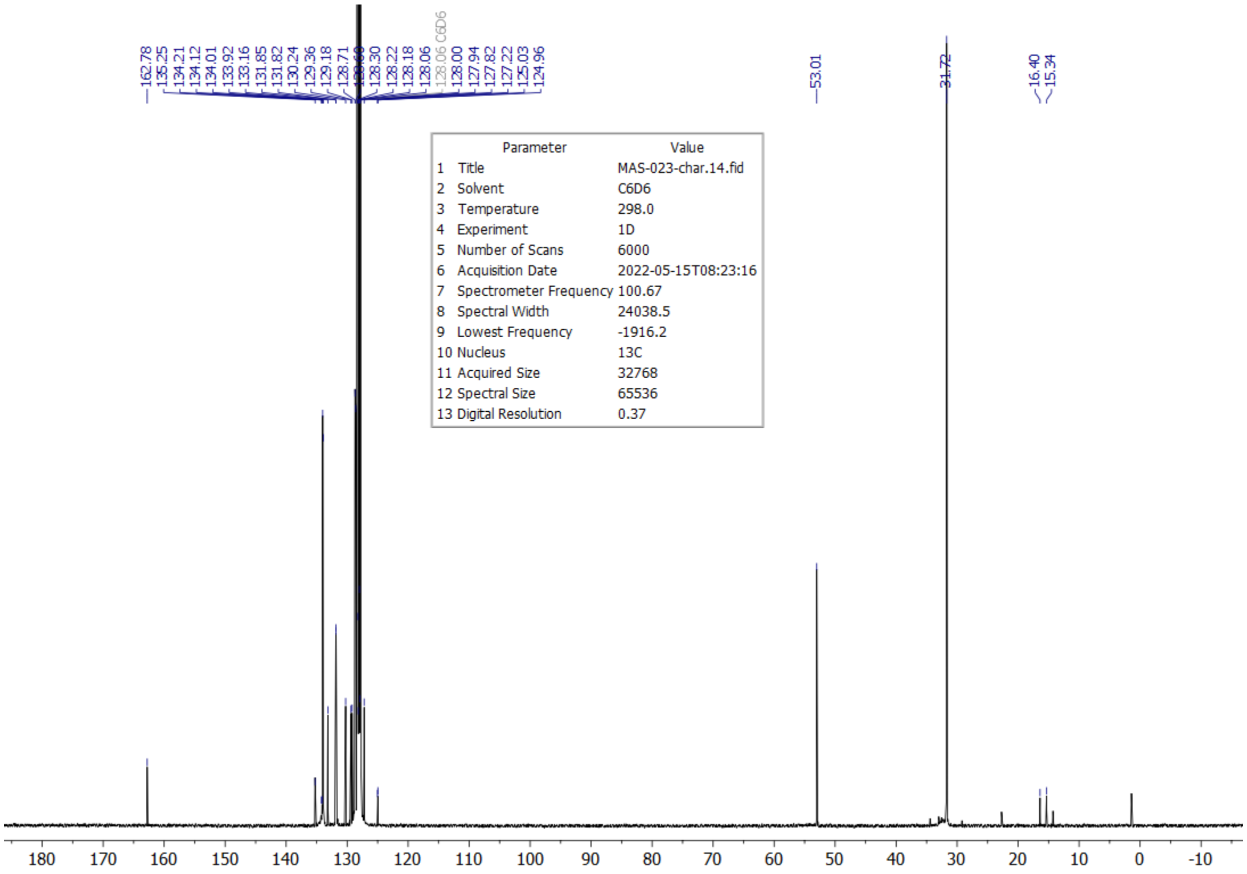

Figure S29. $^{13}C\{^1H\}$-NMR spectrum of **AYSi-3** in C_6D_6. The peaks at 34.5, 22.8 and 14.3 ppm correspond to residual pentane.

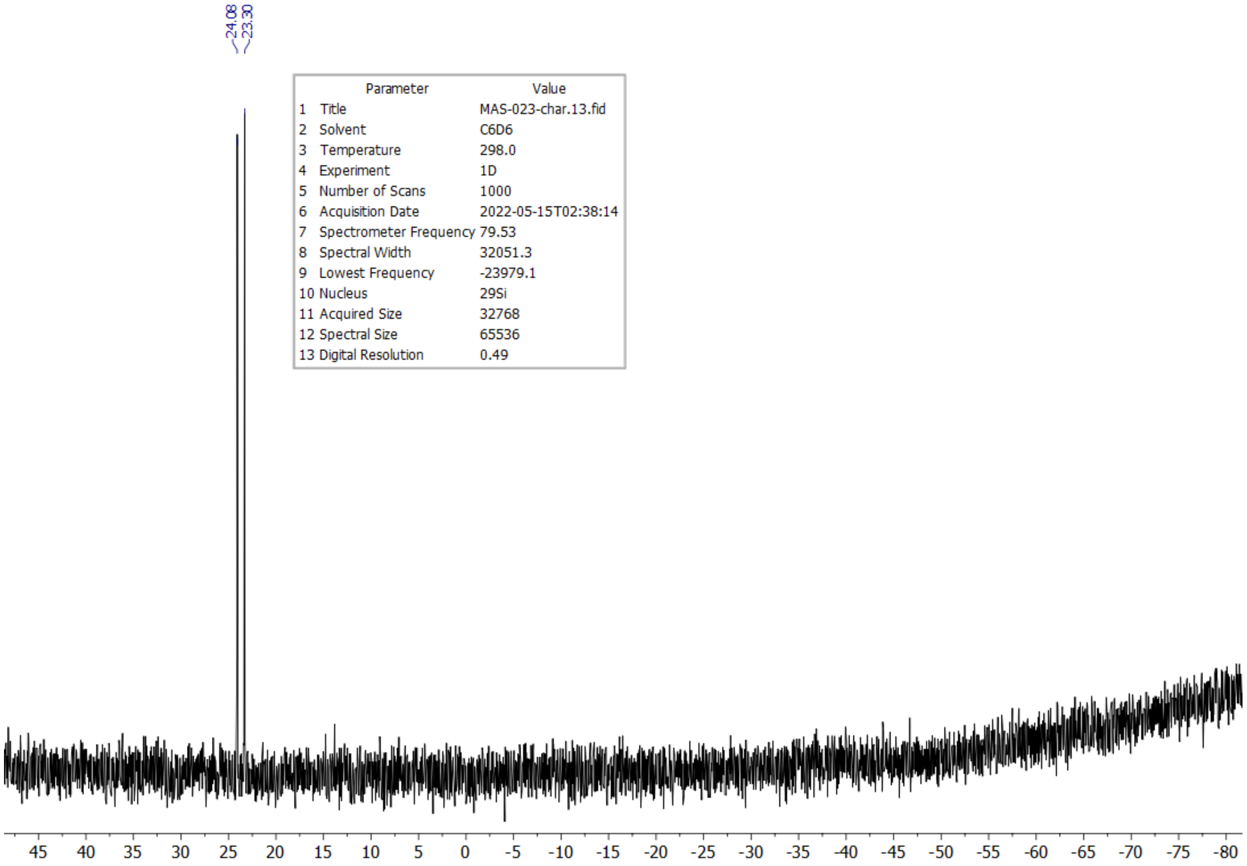

Figure S30. $^{29}Si\{^1H\}$-NMR spectrum of **AYSi-3** in C_6D_6.

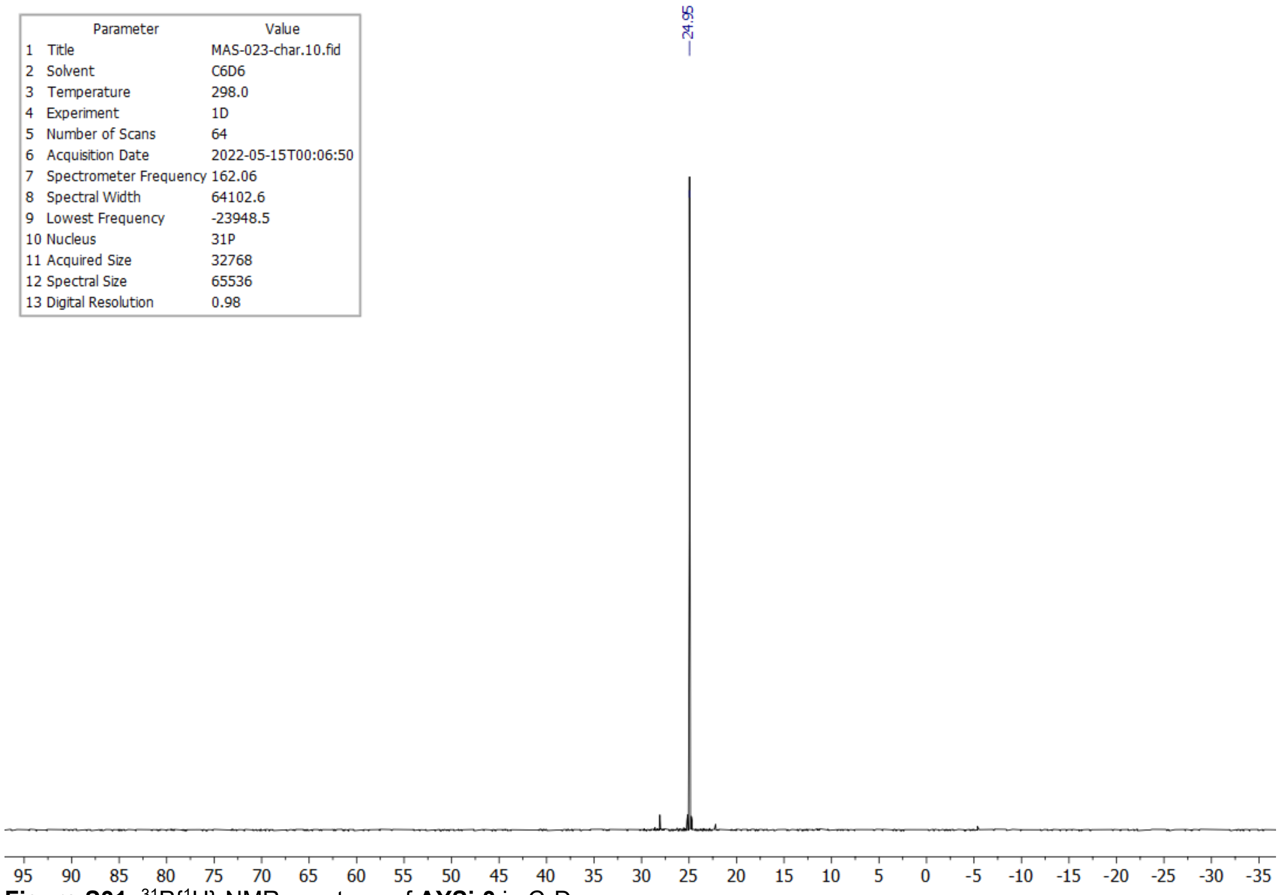

Figure S31. $^{31}P\{^{1}H\}$-NMR spectrum of **AYSi-3** in C_6D_6.

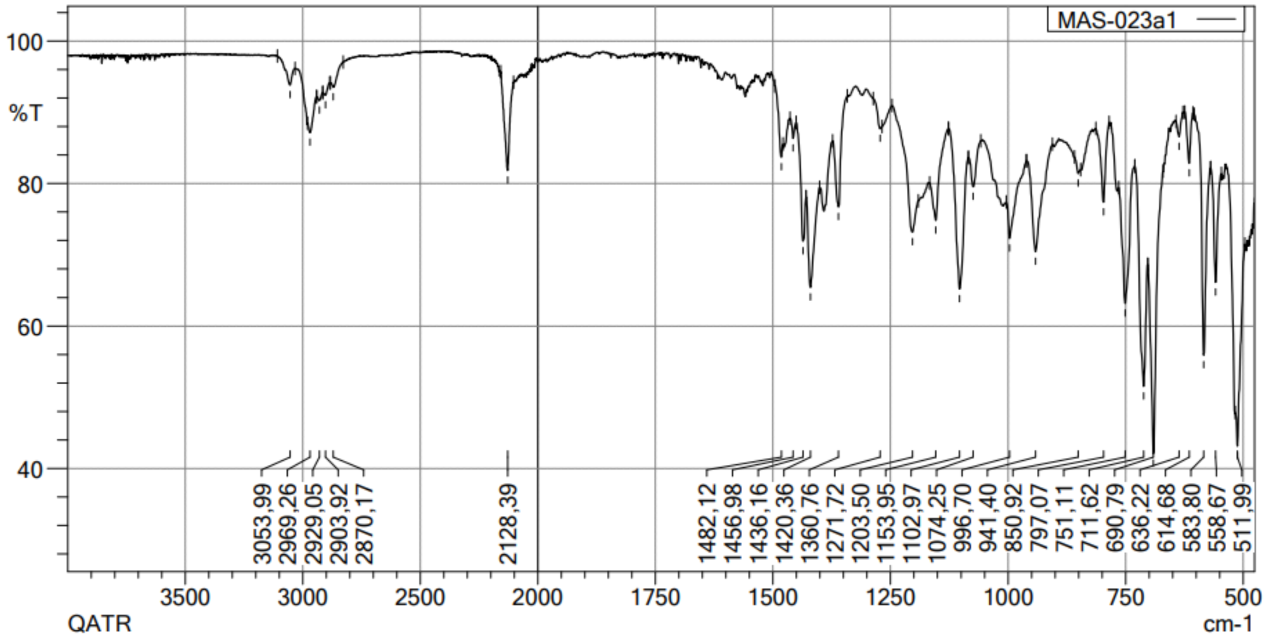

Figure S32. IR-spectrum of solid **AYSi-3**.

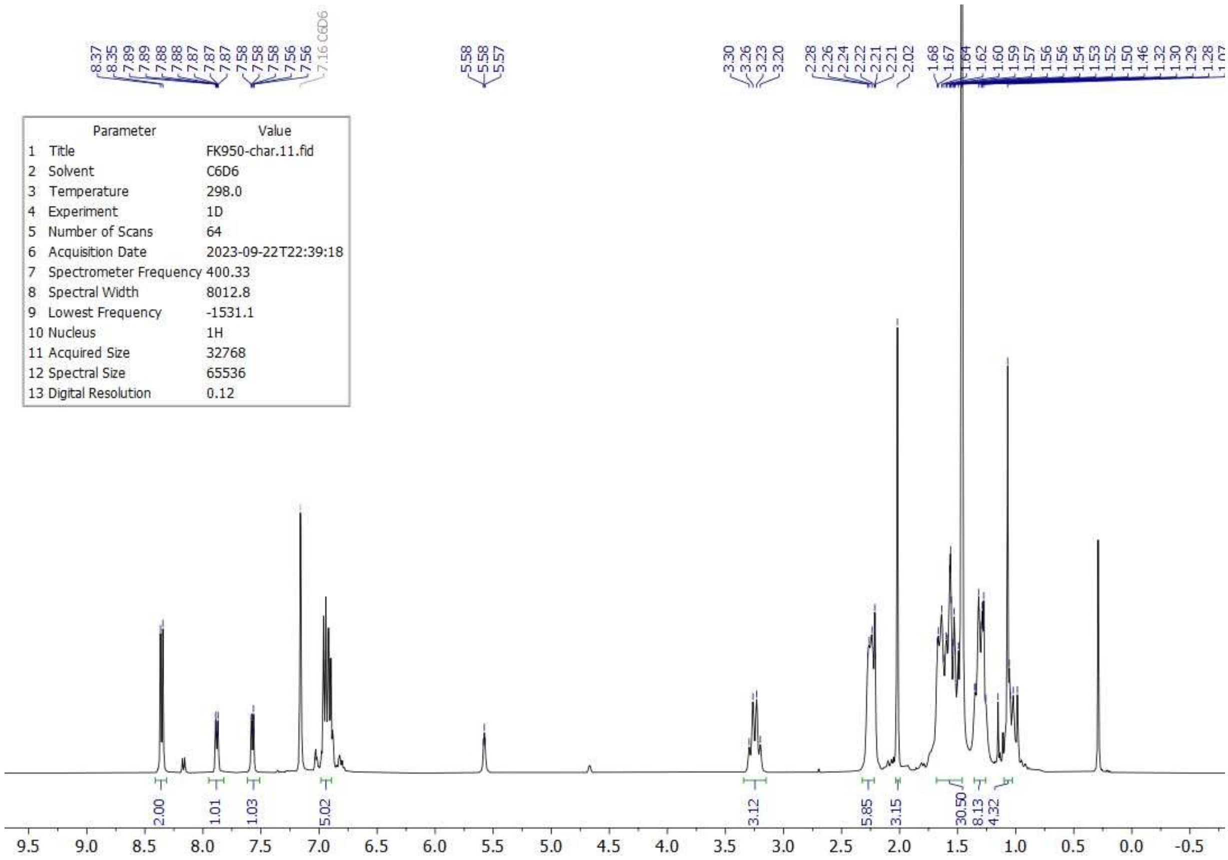

Figure S33. ^{1}H-NMR spectrum of **3** in C_6D_6. The peaks at 5.58 ppm and 2.21 ppm correspond to residual 1,5-cyclooctadiene. The peak 0.26 ppm corresponds to silicon grease.

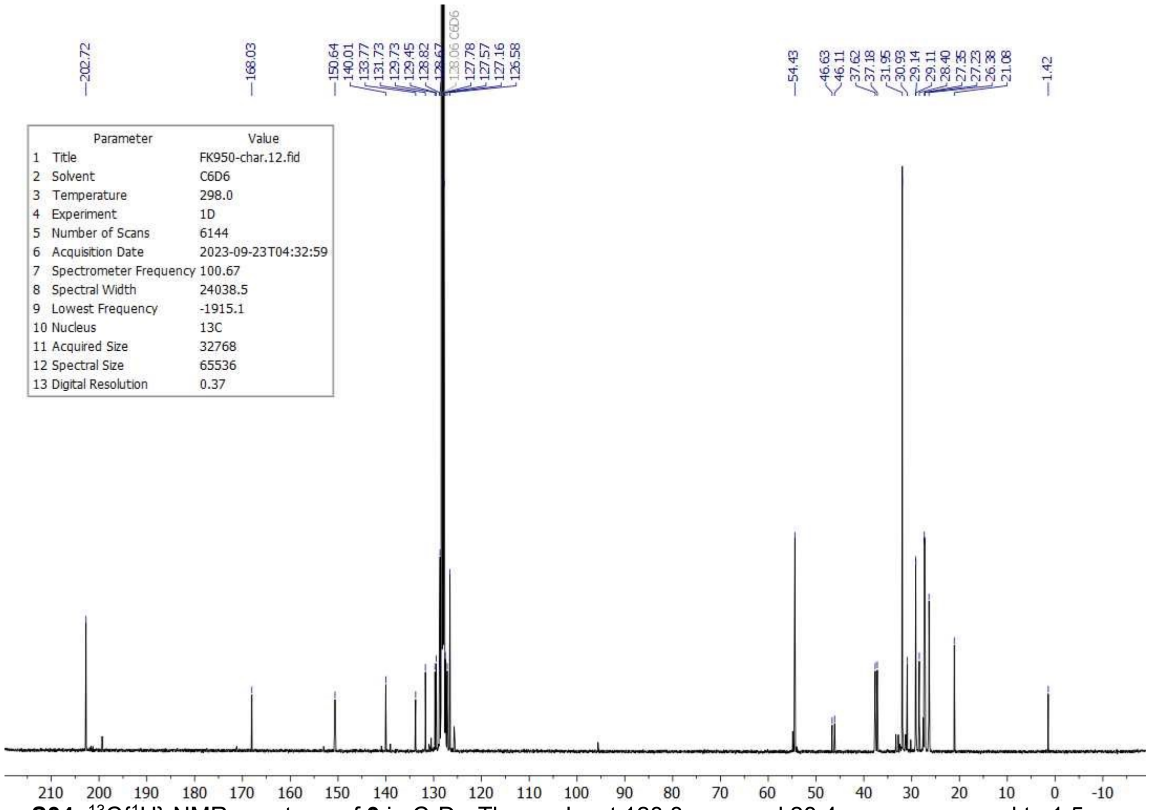

Figure S34. $^{13}C\{^{1}H\}$-NMR spectrum of **3** in C_6D_6. The peaks at 128.8 ppm and 28.4 ppm correspond to 1,5-cyclooctadiene. The peak at 1.4 ppm corresponds to silicon grease.

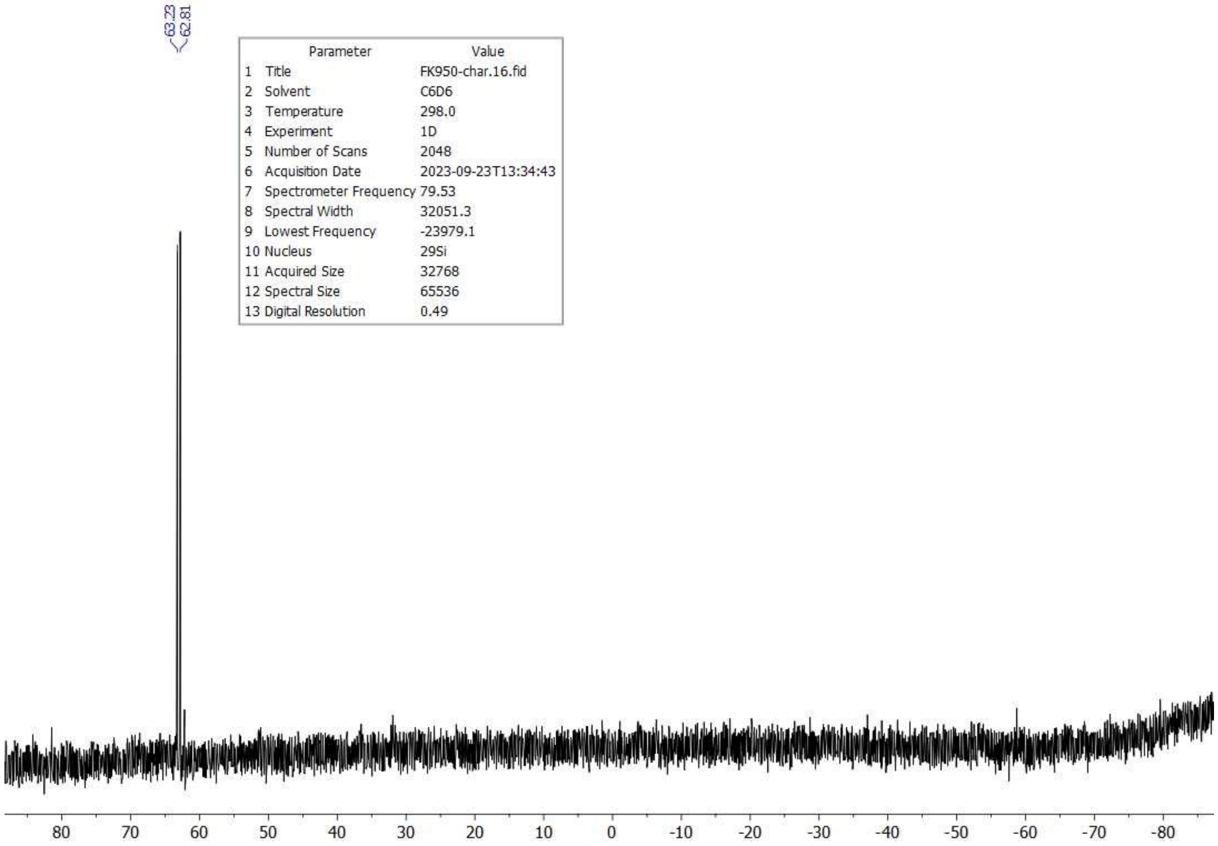

Figure S35. $^{29}Si\{^1H\}$-NMR spectrum of **3** in C_6D_6.

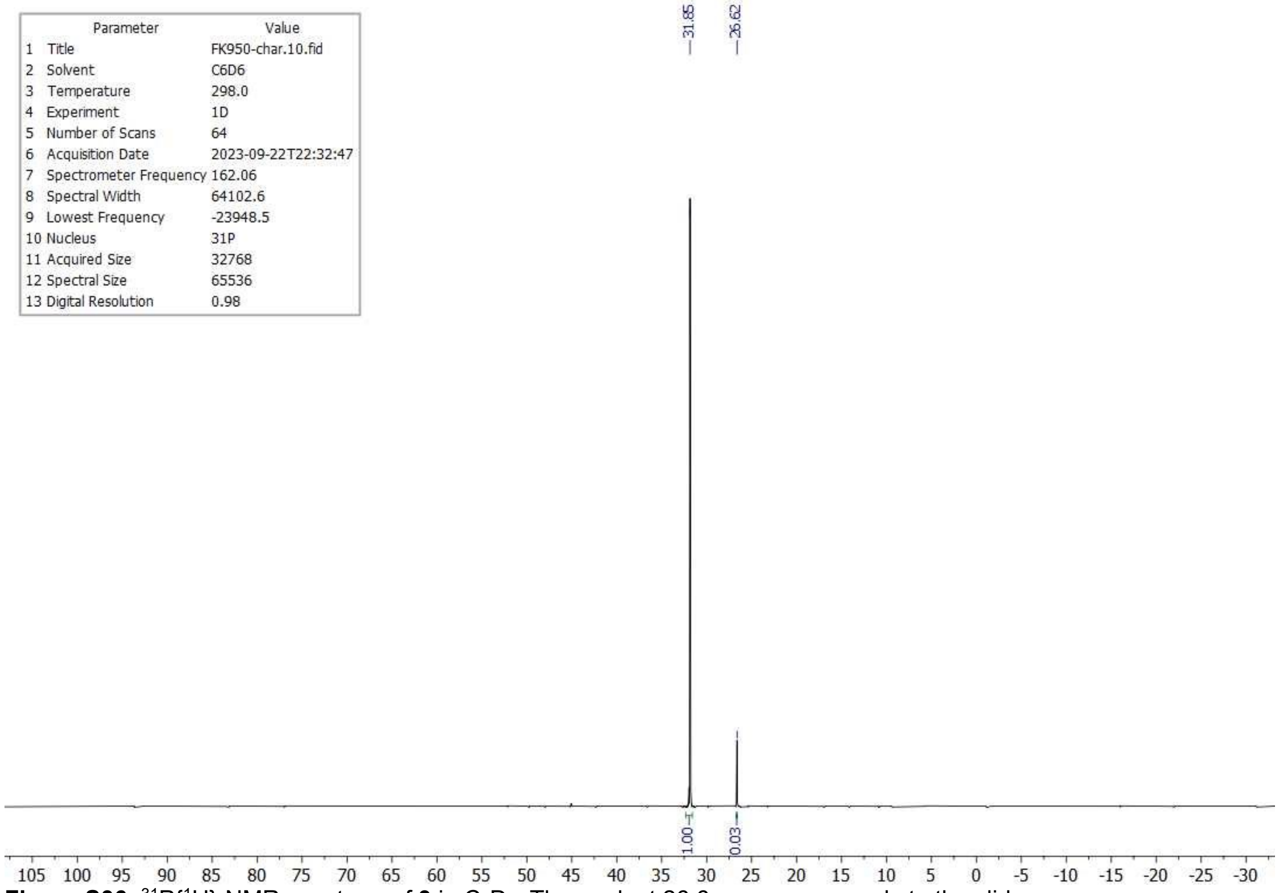

Figure S36. $^{31}P\{^1H\}$-NMR spectrum of **3** in C_6D_6. The peak at 26.6 ppm corresponds to the ylide.

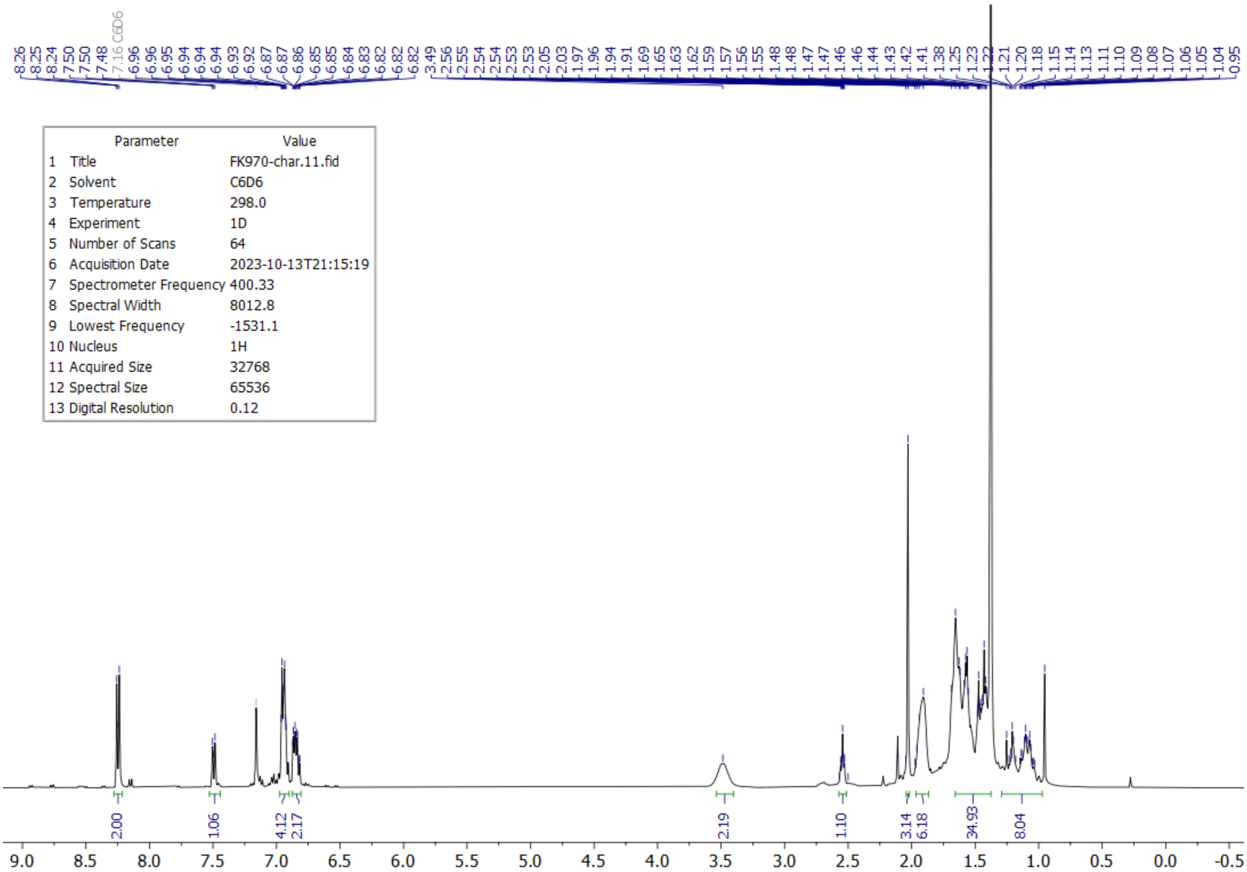

Figure S37. ^{1}H-NMR spectrum of **4** in C_6D_6. The sample contains residual toluene (7.13 ppm, 7.02 ppm, and 2.11 ppm).

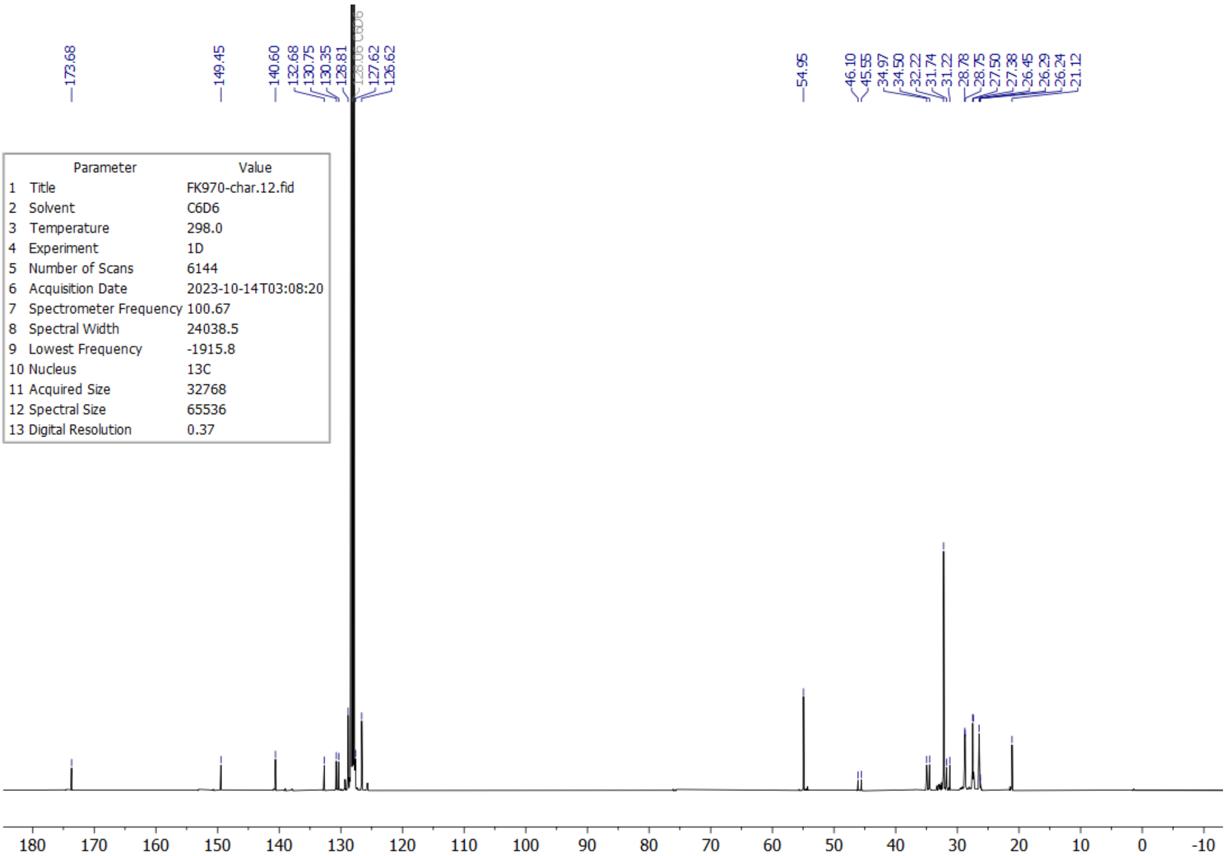

Figure S38. $^{13}C\{^{1}H\}$-NMR spectrum of **4** in C_6D_6. The sample contains residual toluene (129.3 ppm, 128.6 ppm, 125.7 ppm, and 21.1 ppm).

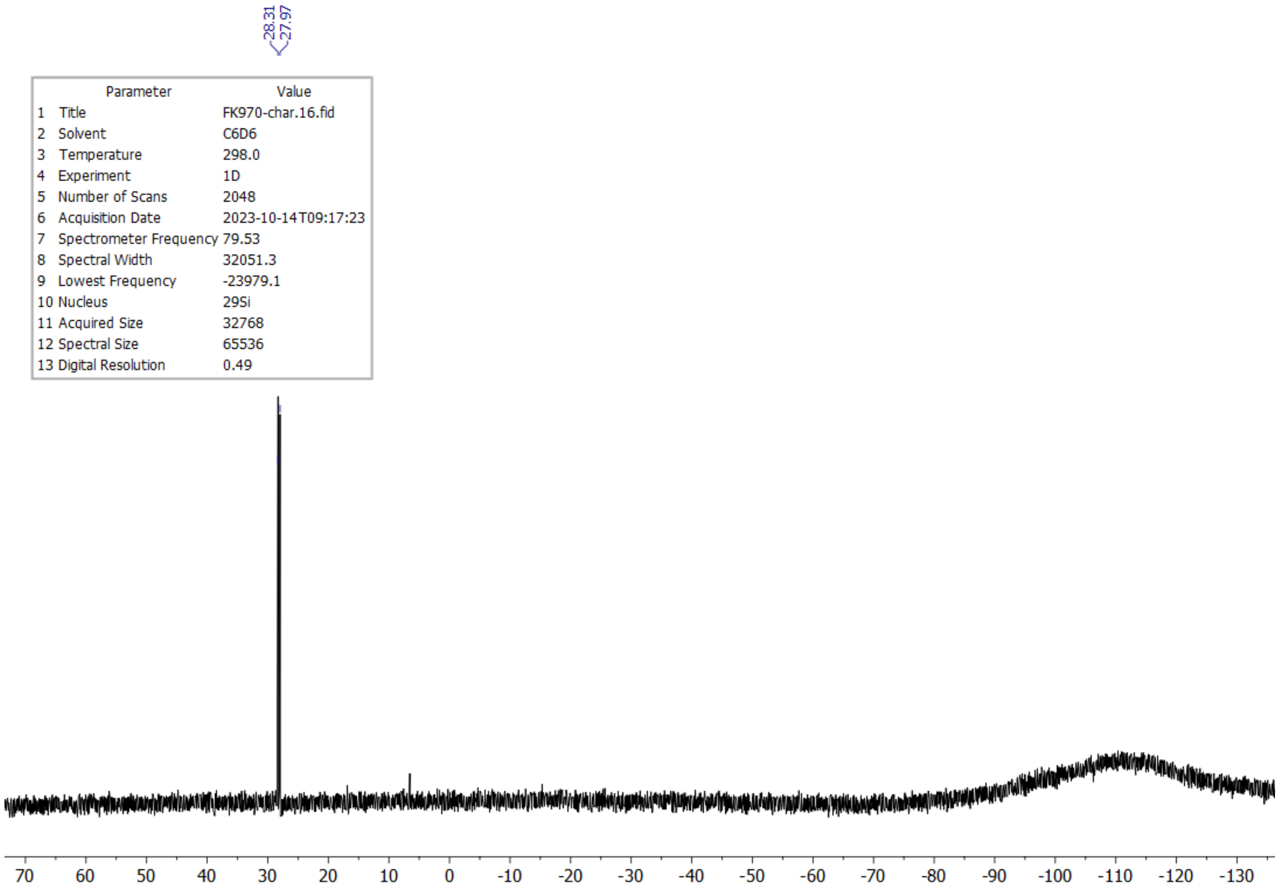

Figure S39. $^{29}Si\{^{1}H\}$-NMR spectrum of **4** in C_6D_6.

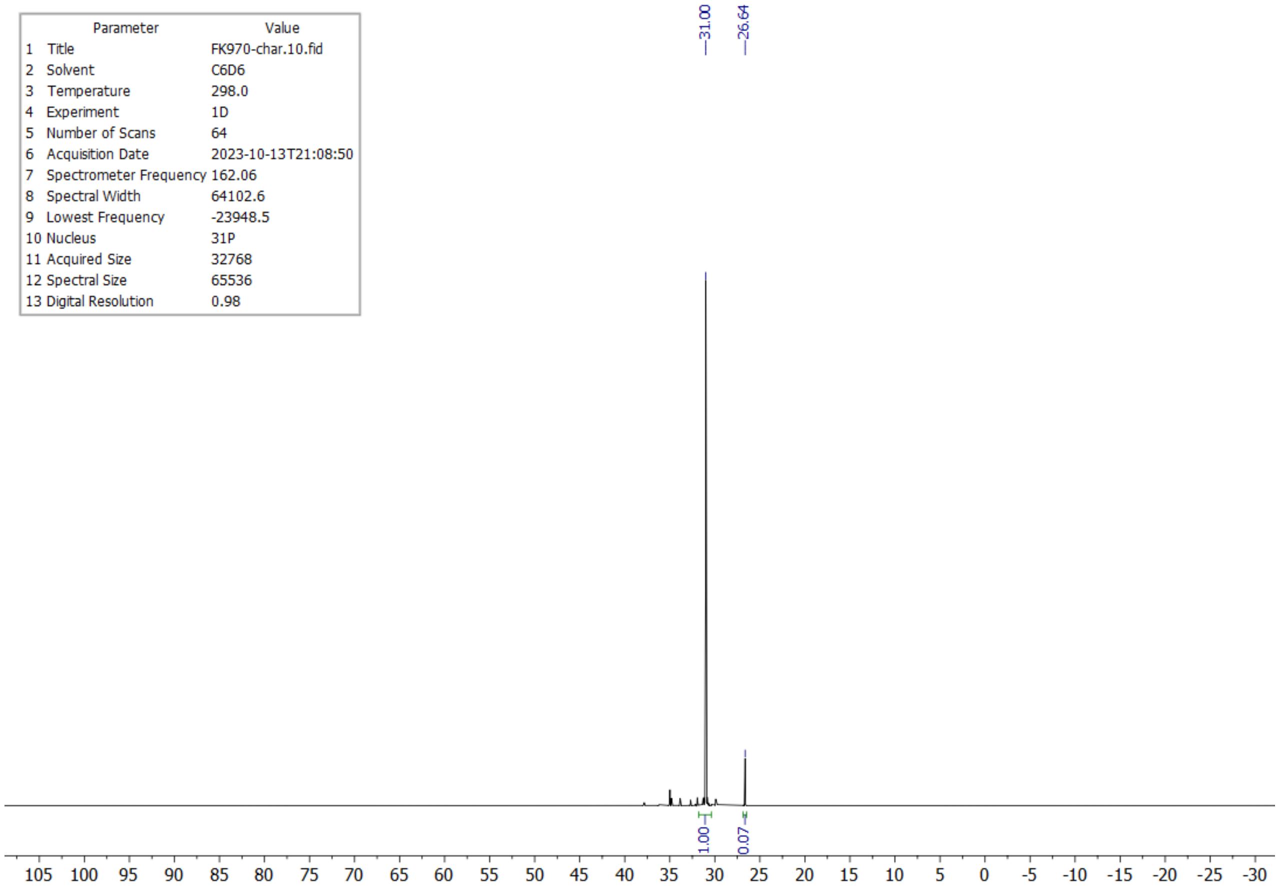

Figure S40. $^{31}P\{^{1}H\}$-NMR spectrum of **4** in C_6D_6. The peak at 26.6 ppm corresponds to the ylide.

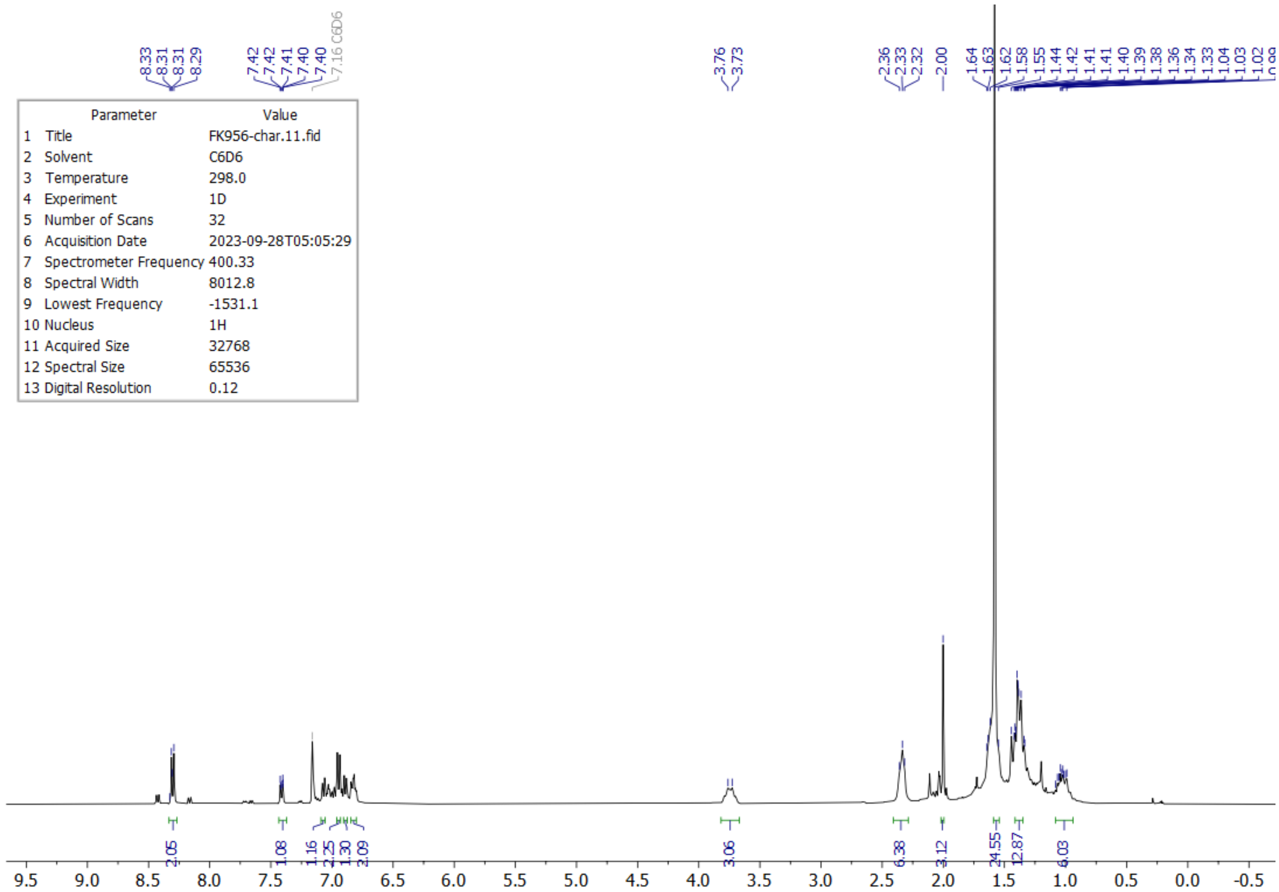

Figure S41. ^{1}H-NMR spectrum of **5$_{Ts}$** in C_6D_6.

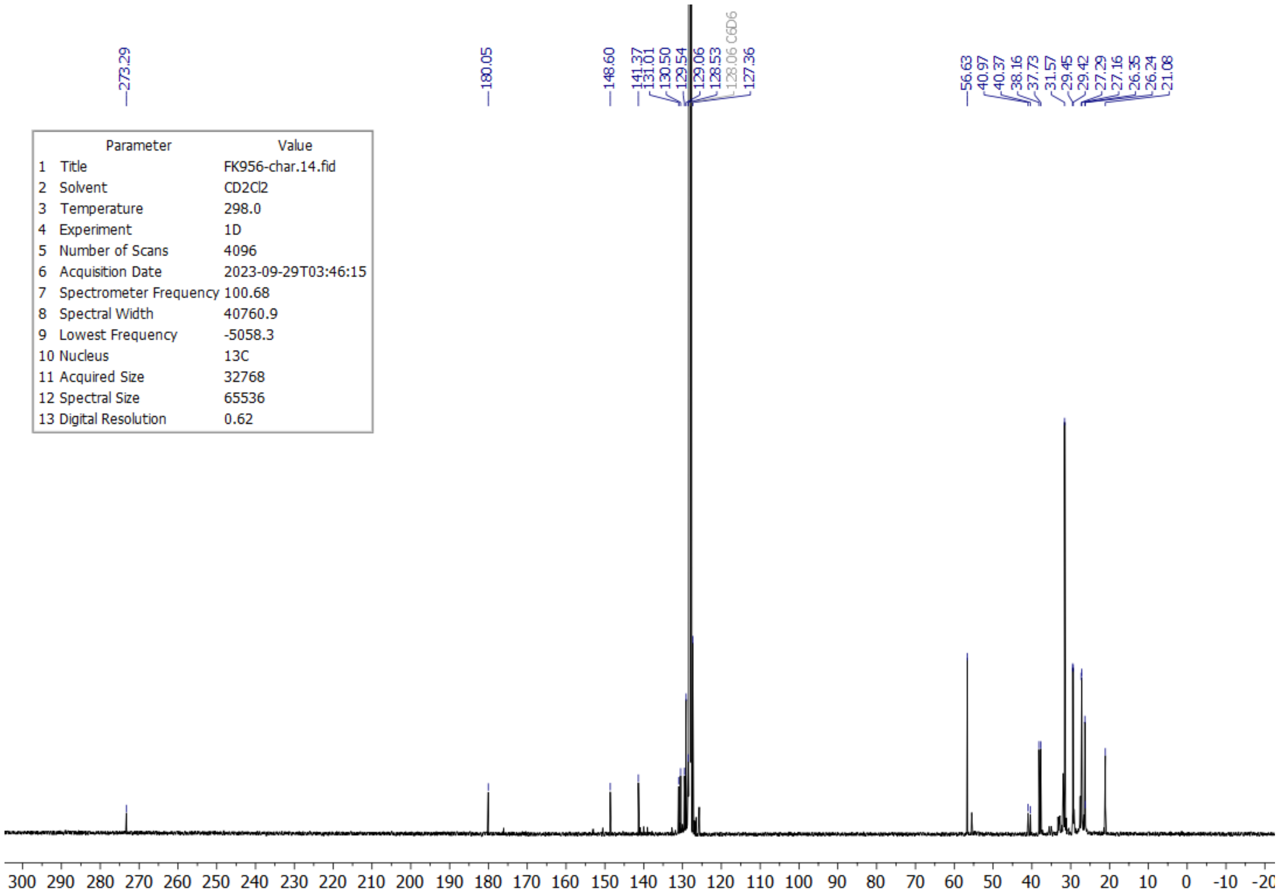

Figure S42. ^{13}C{^{1}H}-NMR spectrum of **5$_{Ts}$** in C_6D_6.

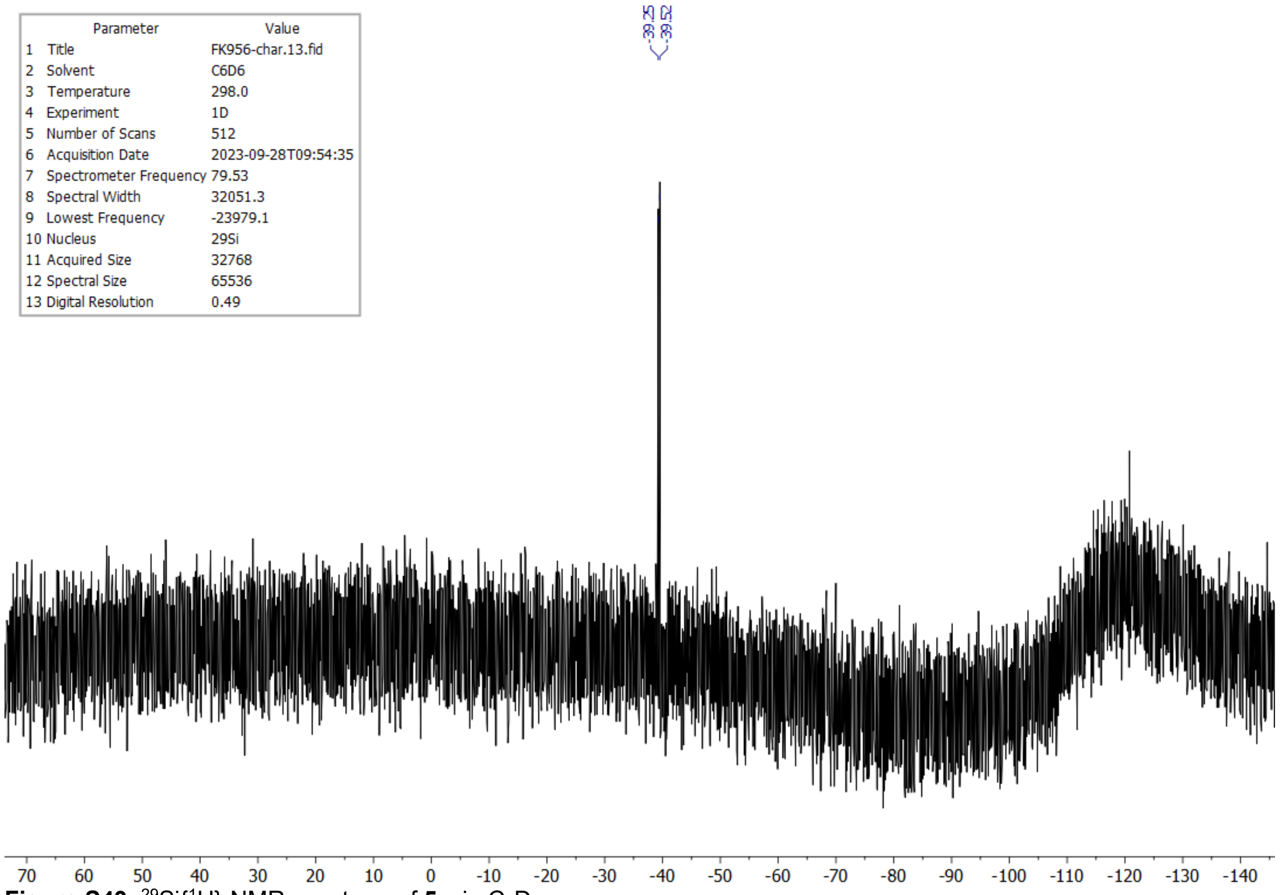

Figure S43. $^{29}Si\{^{1}H\}$-NMR spectrum of **5**$_{Ts}$ in C_6D_5.

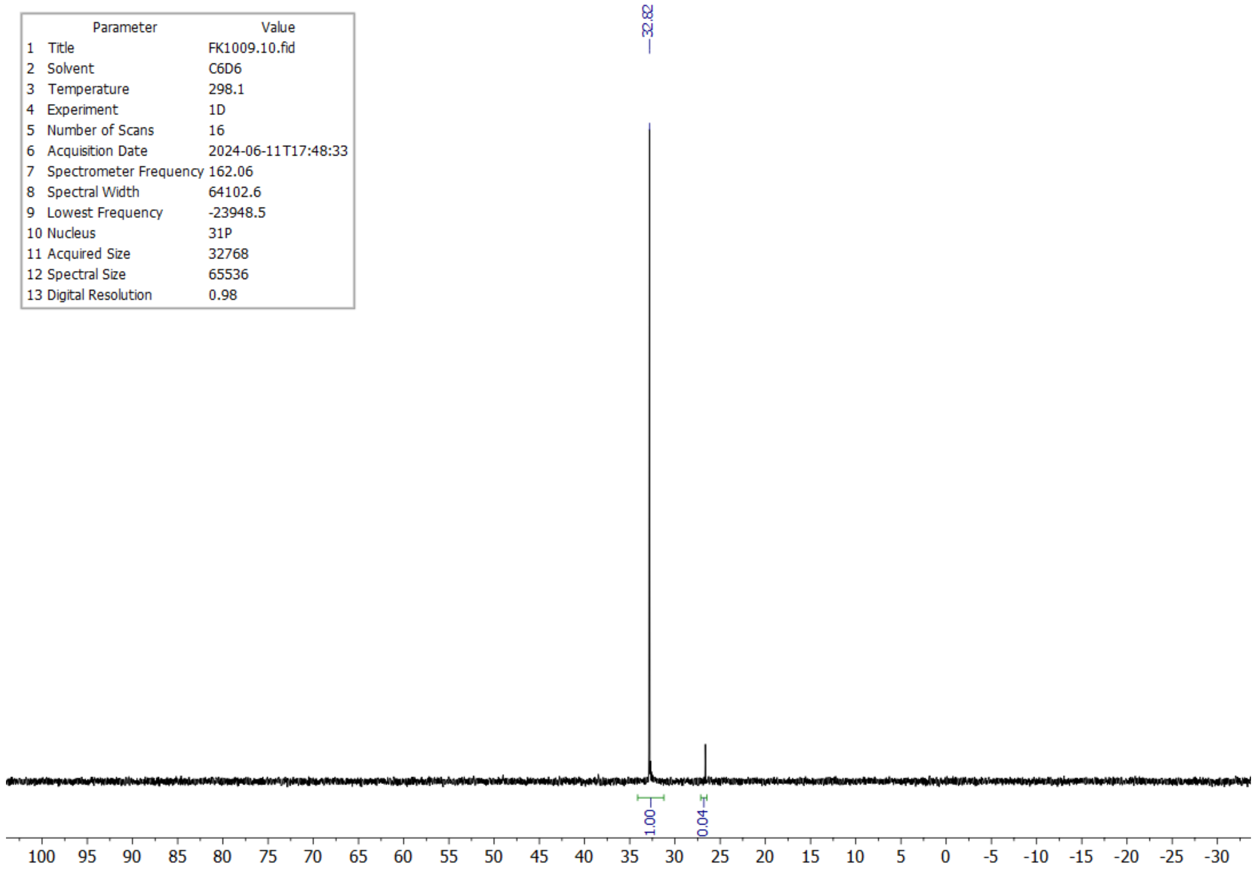

Figure S44. $^{31}P\{^{1}H\}$-NMR spectrum of **5**$_{Ts}$ in C_6D_6. The peak at 26.6 ppm corresponds to the free ylide.

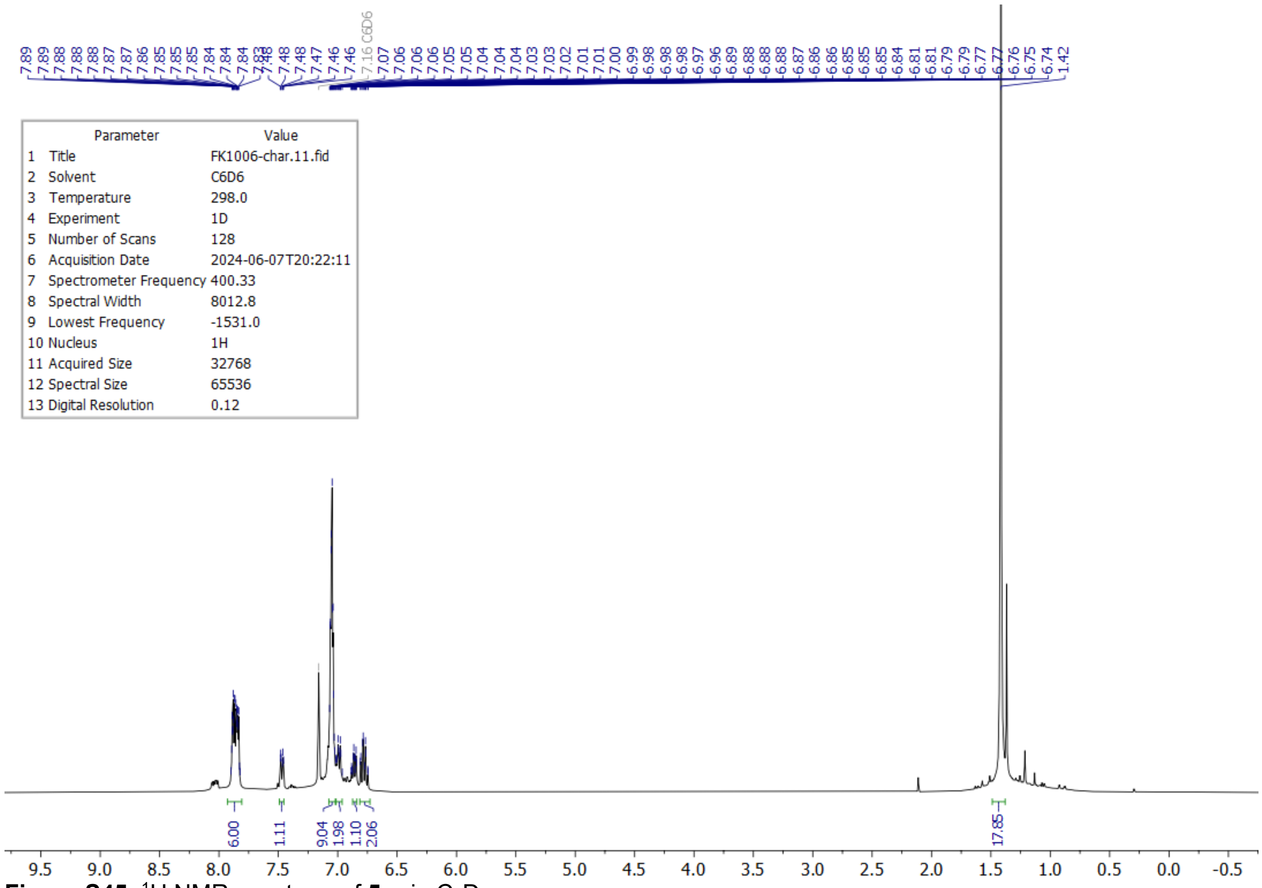

Figure S45. ^{1}H NMR spectrum of $\mathbf{5_{CN}}$ in C_6D_6.

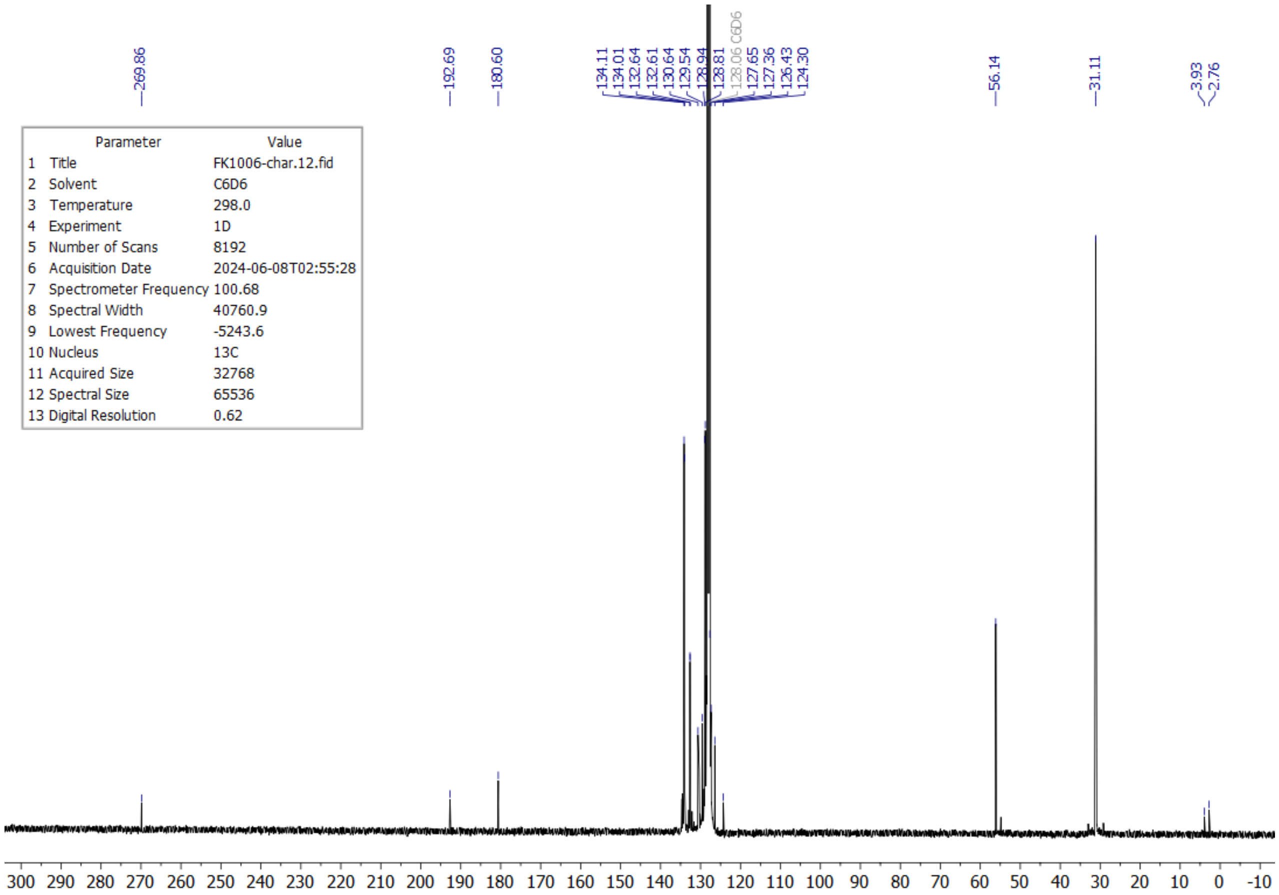

Figure S46. $^{13}C\{^{1}H\}$-NMR spectrum of $\mathbf{5_{CN}}$ in C_6D_6. The peak at 192.7 ppm corresponds to free CS_2.

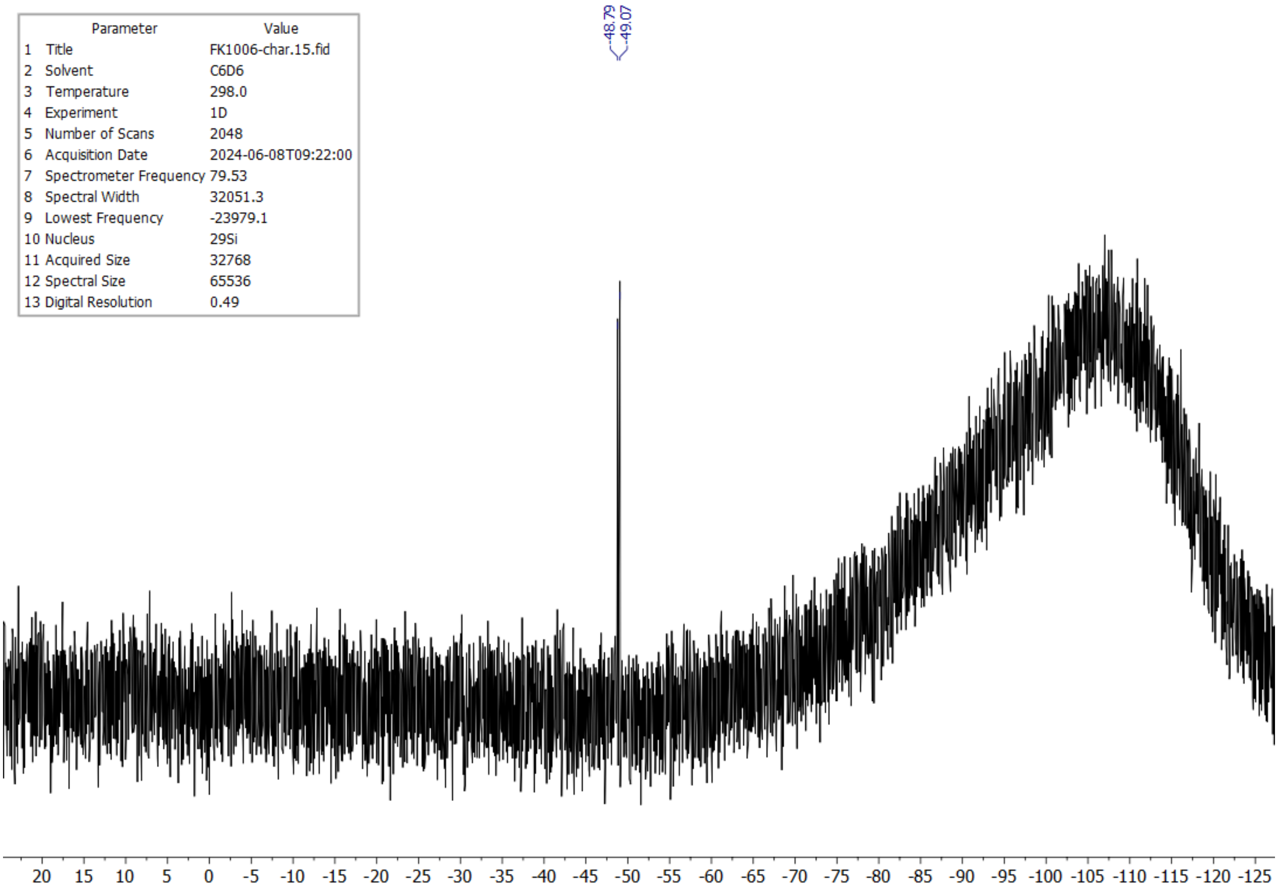

Figure S47. $^{29}Si\{^1H\}$-NMR spectrum of **5**$_{\mathbf{CN}}$ in C_6D_6.

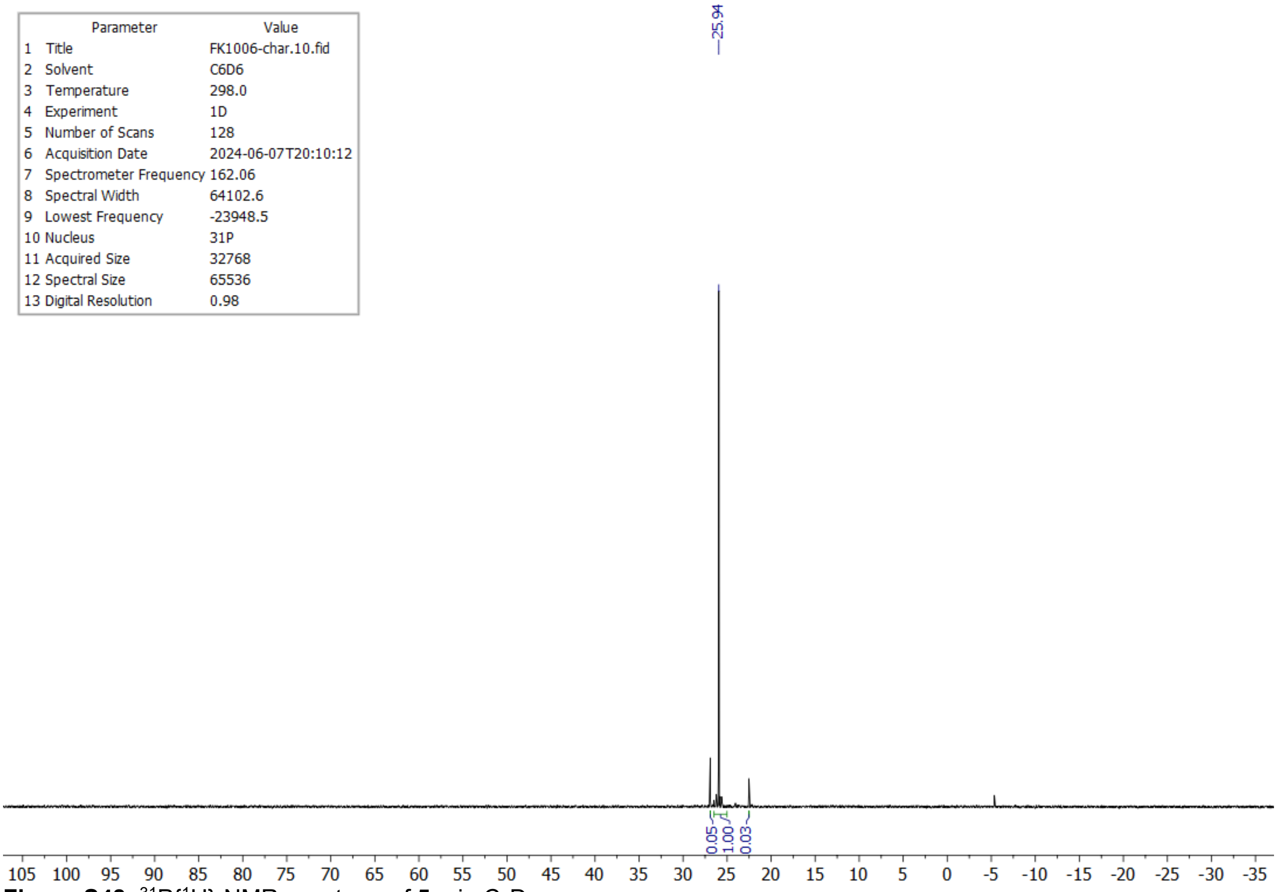

Figure S48. $^{31}P\{^1H\}$-NMR spectrum of **5**$_{\mathbf{CN}}$ in C_6D_6.

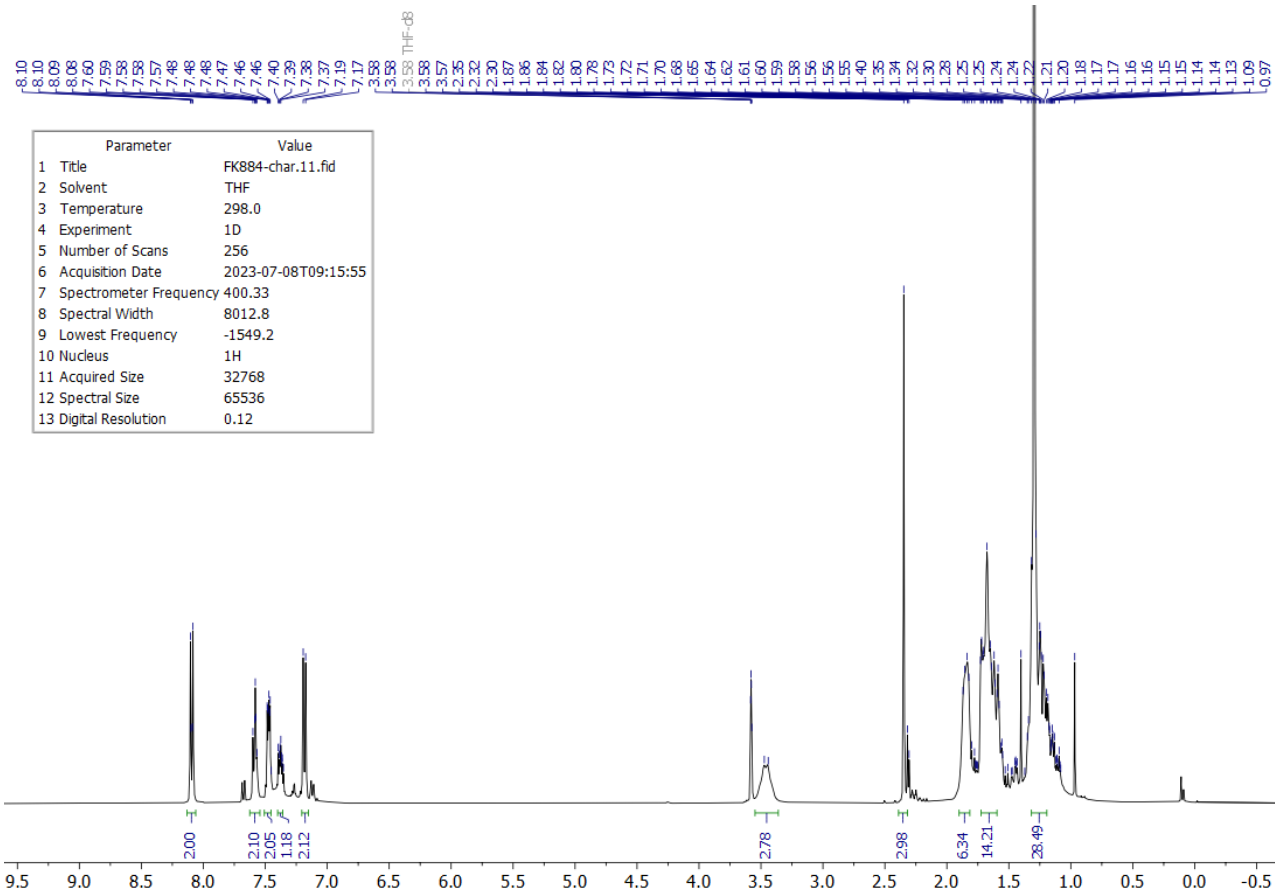

Figure S49. ^{1}H-NMR spectrum of **6** in THF-d_8. The sample contains residual toluene (7.19 ppm, 7.10 ppm, and 2.31 ppm).

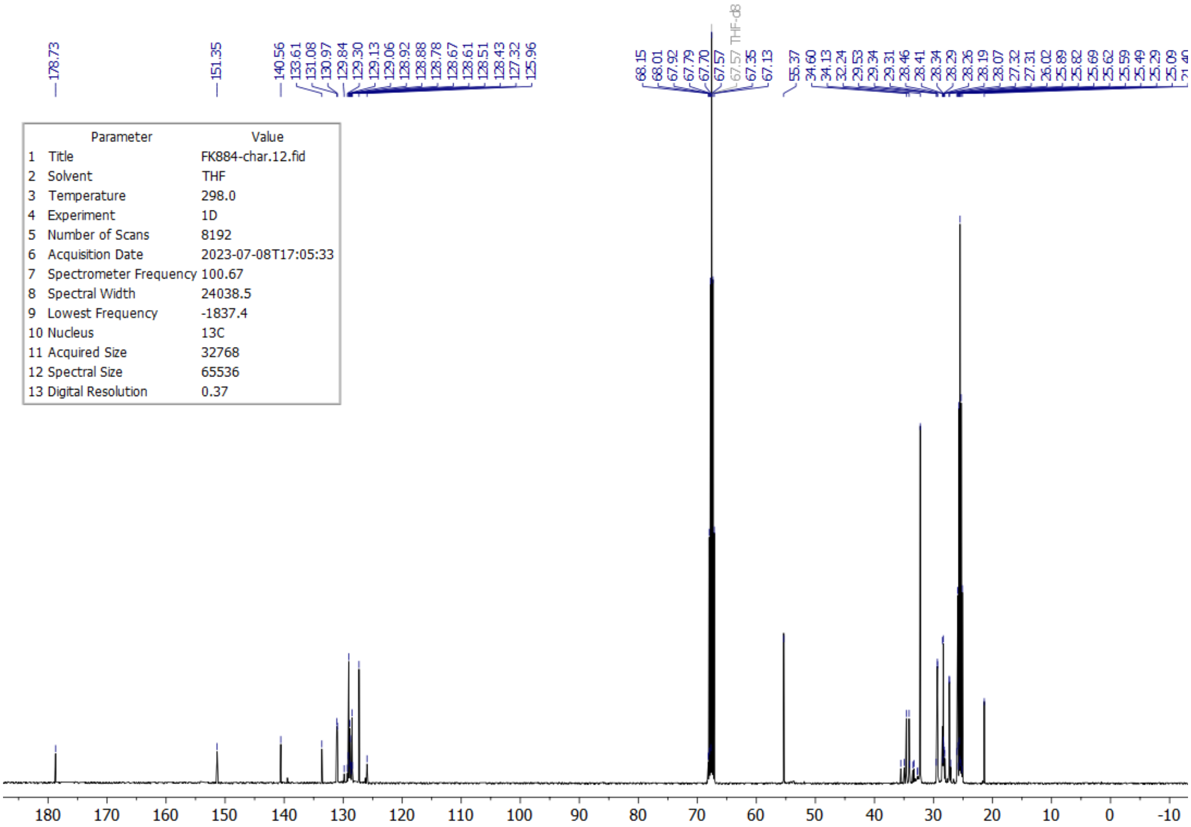

Figure S50. $^{13}C\{^{1}H\}$-NMR spectrum of **6** in THF-d_8. The sample contains residual toluene (138.2 ppm, 129.5 ppm, 128.7 ppm, 125.8 ppm, and 21.3 ppm.)

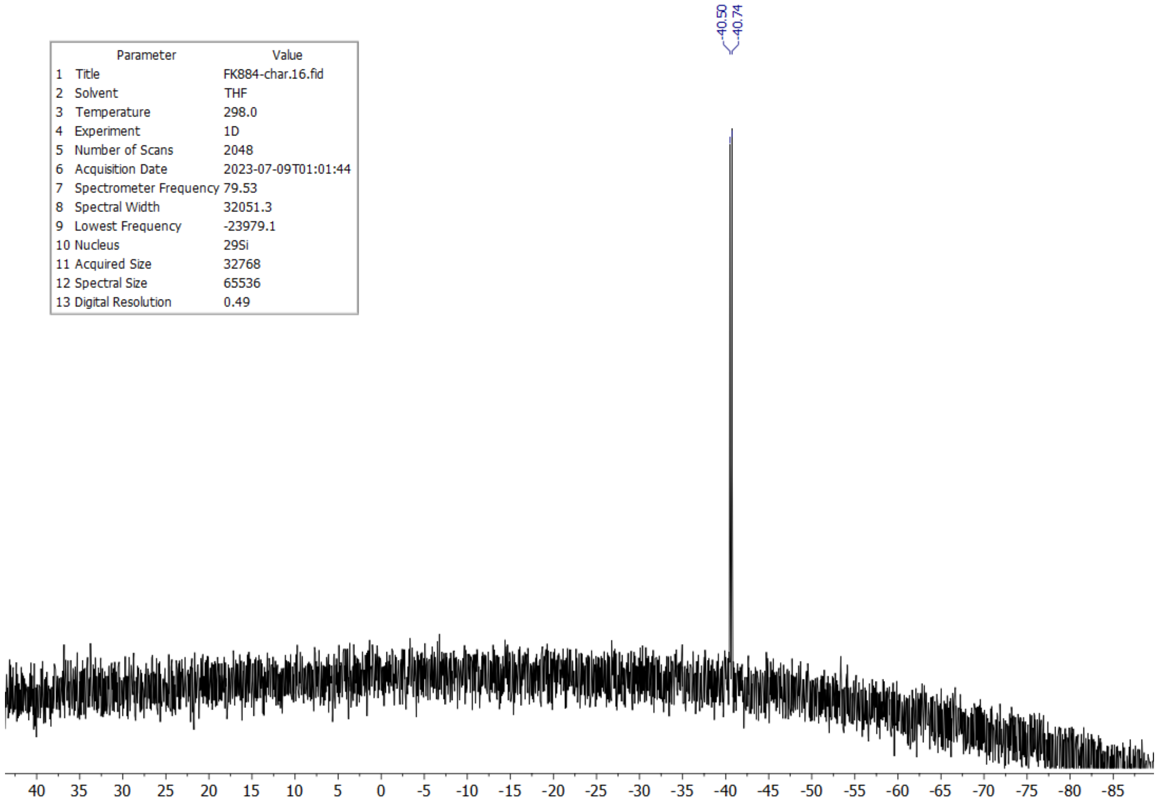

Figure S51. $^{29}Si\{^1H\}$-NMR spectrum of **6** in THF-d_8.

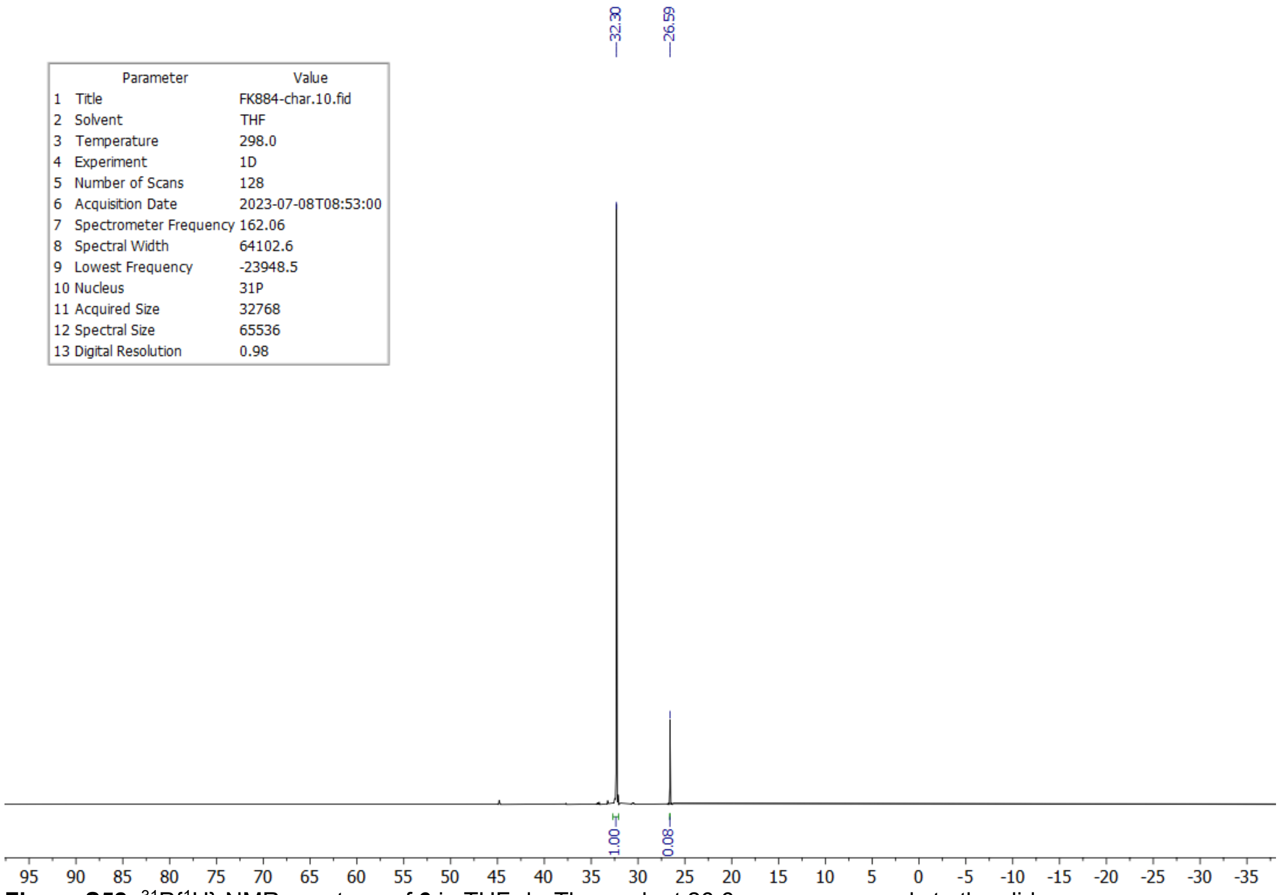

Figure S52. $^{31}P\{^1H\}$-NMR spectrum of **6** in THF-d_8. The peak at 26.6 ppm corresponds to the ylide.

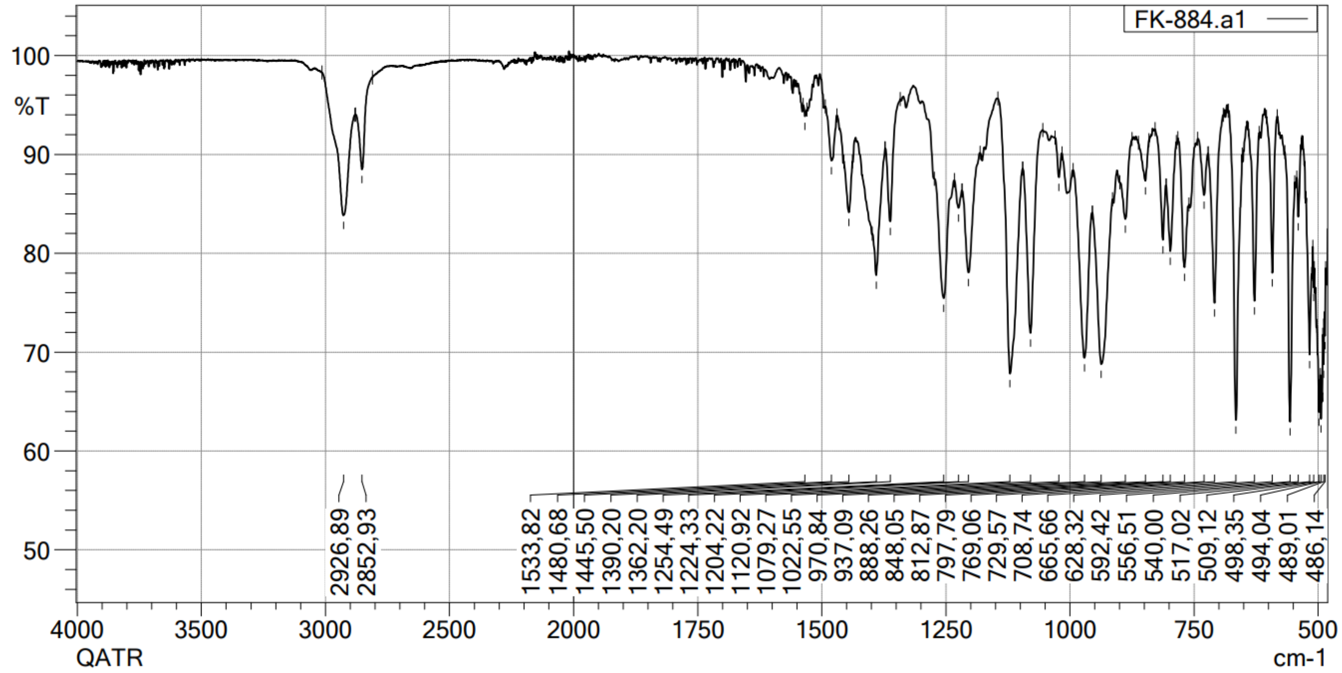

Figure S53. IR-Spectrum of solid **6**.

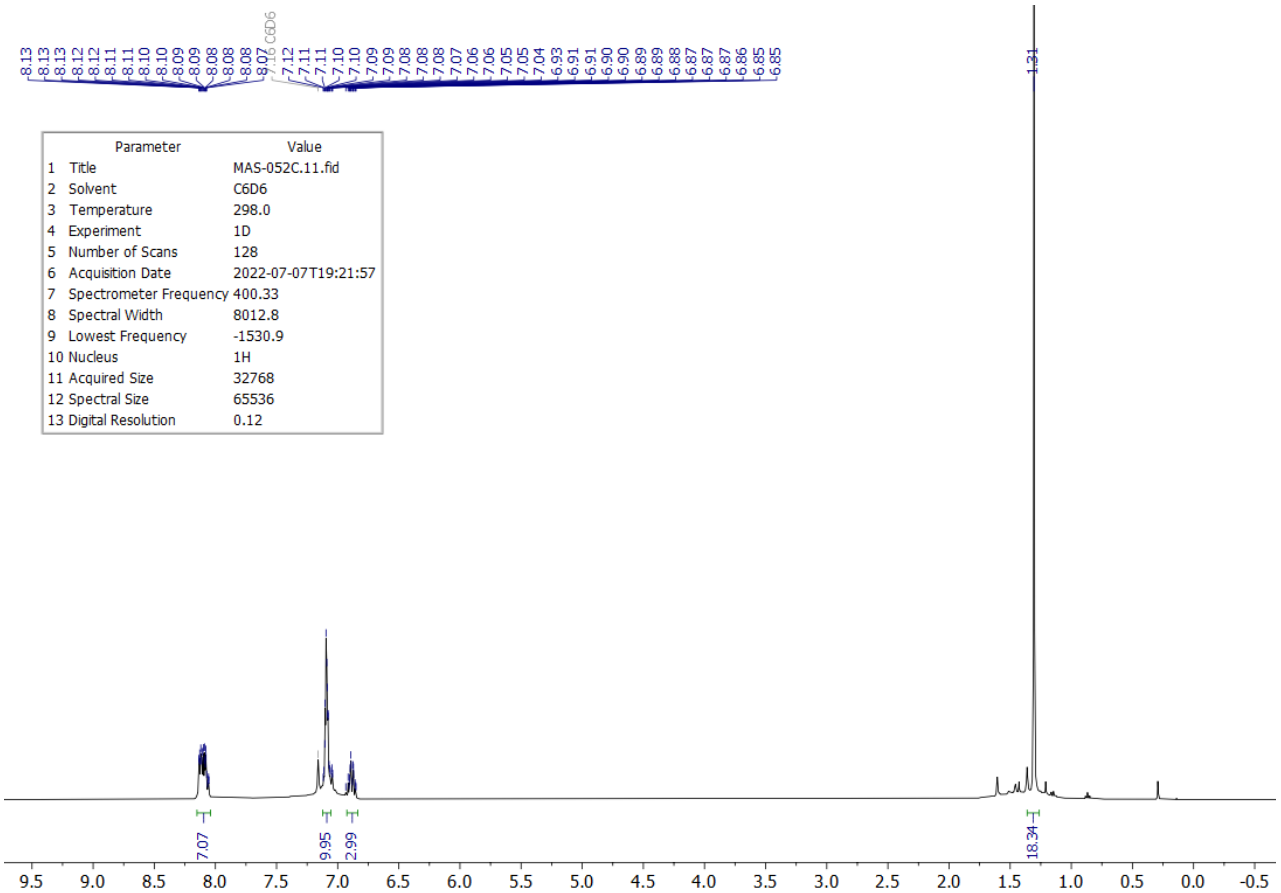

Figure S54. ^{1}H-NMR spectrum of **7** in C_6D_6.

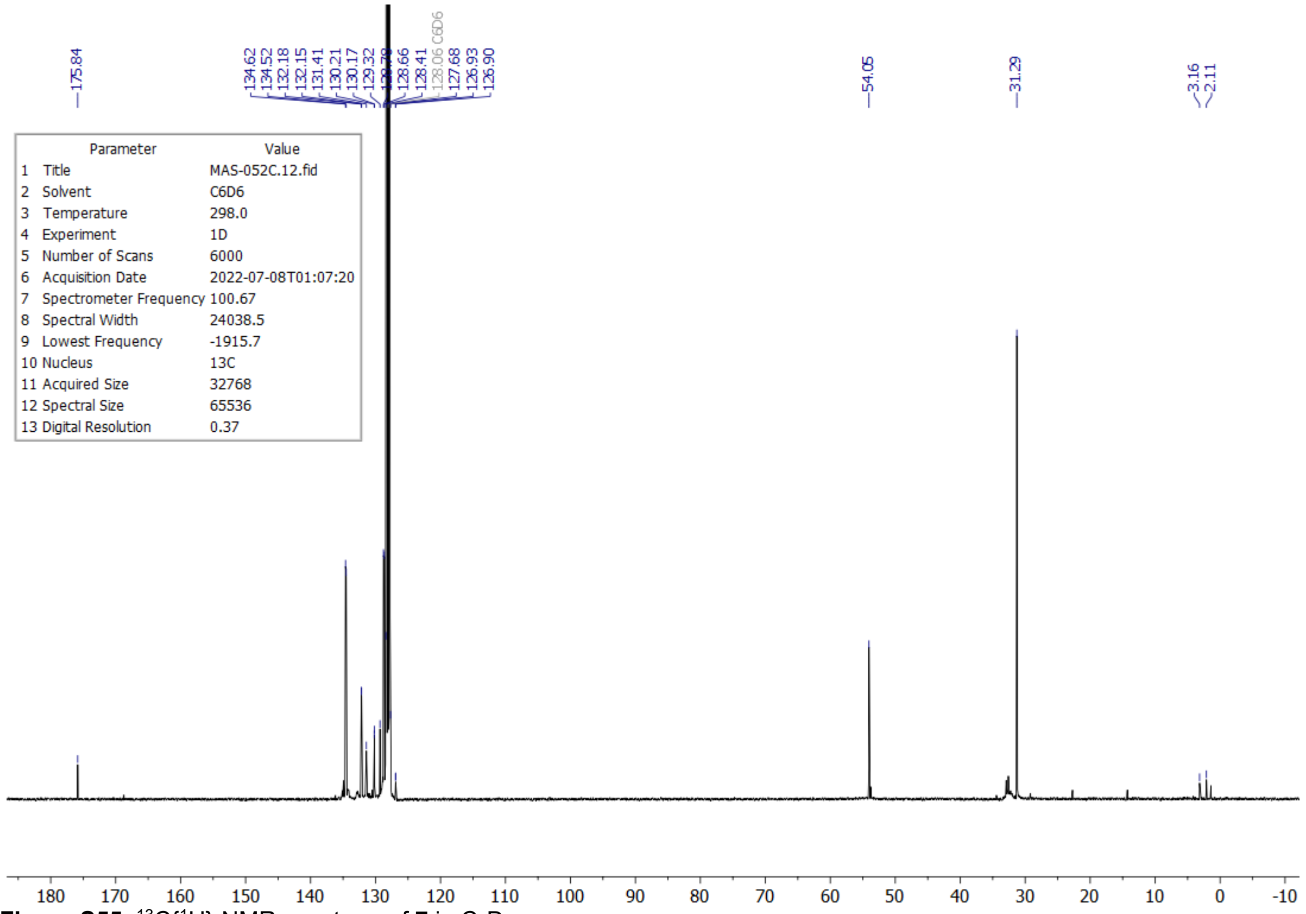

Figure S55. $^{13}C\{^{1}H\}$-NMR spectrum of **7** in C_6D_6.

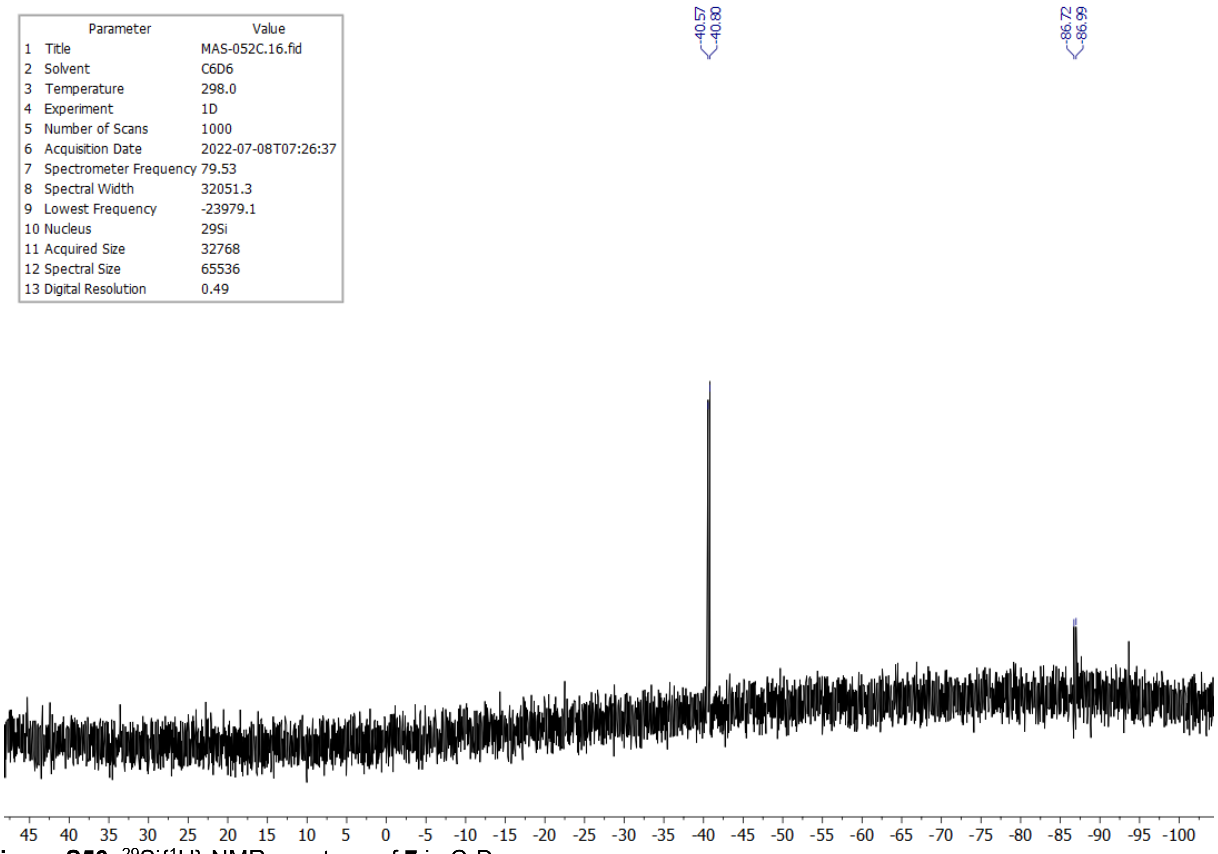

Figure S56. $^{29}Si\{^{1}H\}$-NMR spectrum of **7** in C_6D_6.

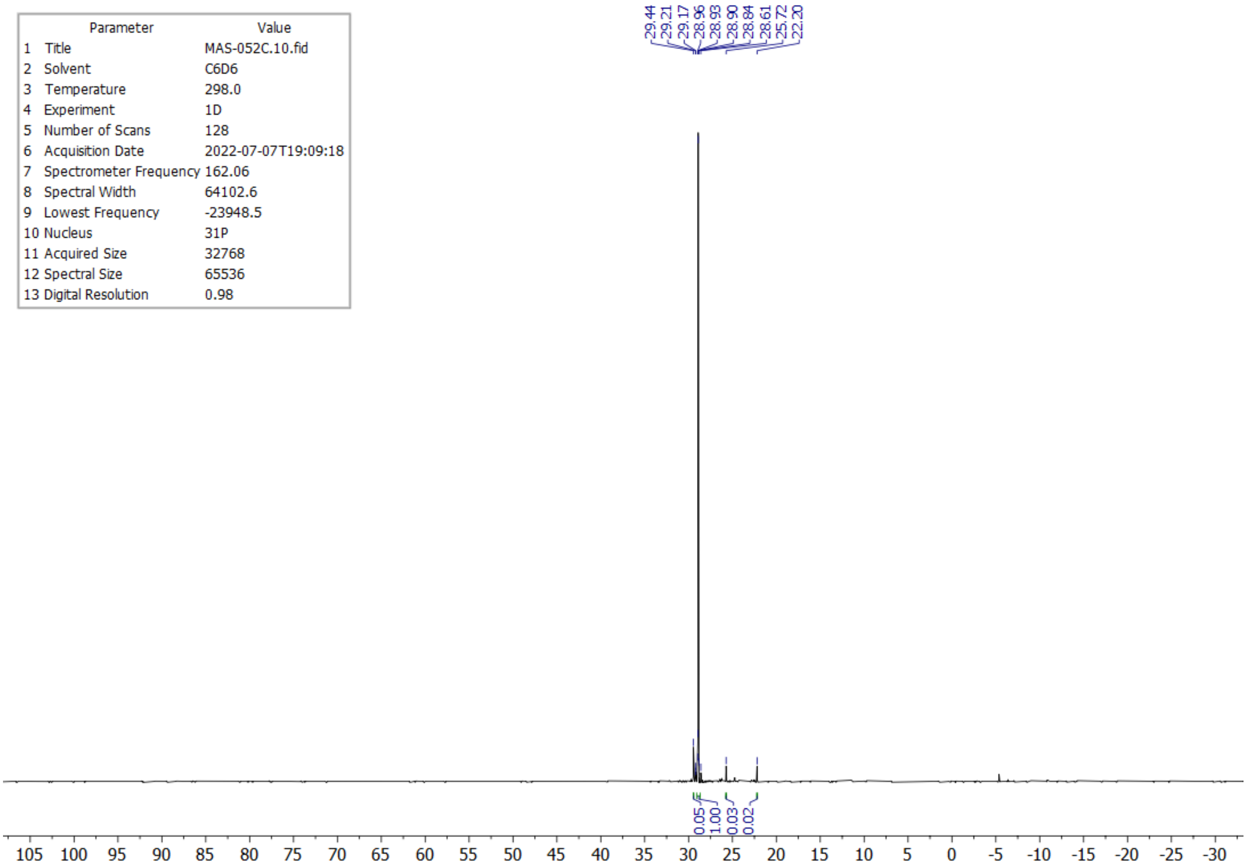

Figure S57. $^{31}P\{^1H\}$-NMR spectrum of **7** in C_6D_6.

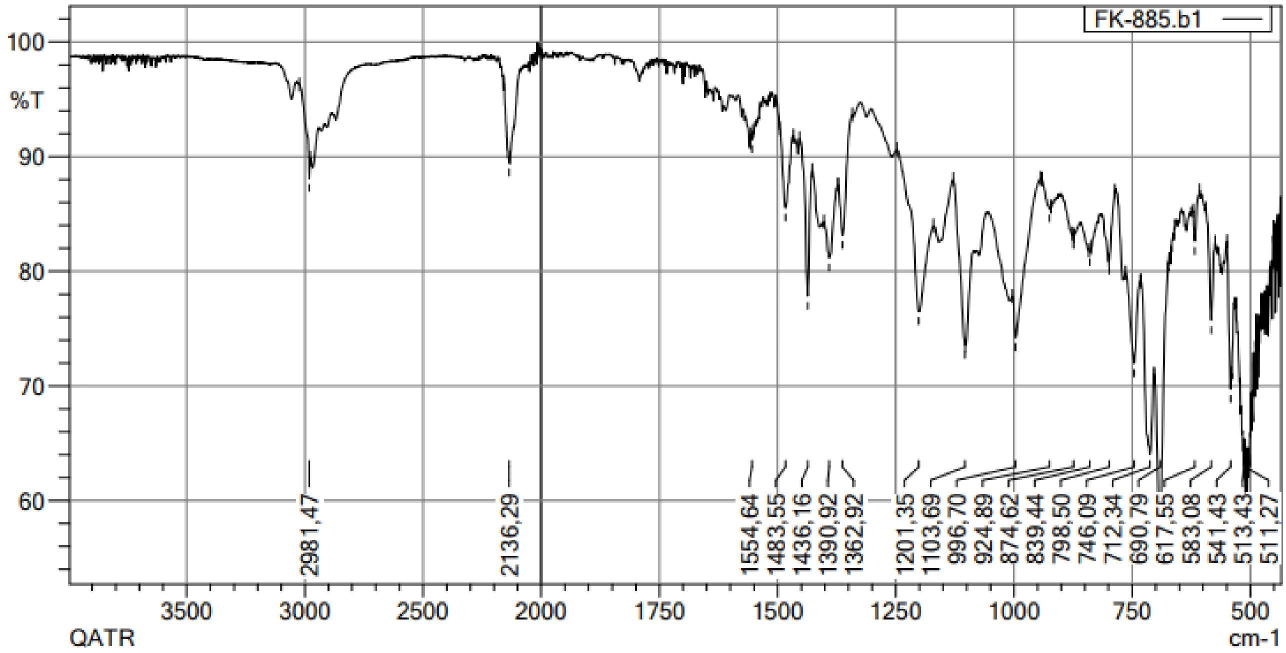

Figure S58. IR-spectrum of the dried reaction mixture of **7**.

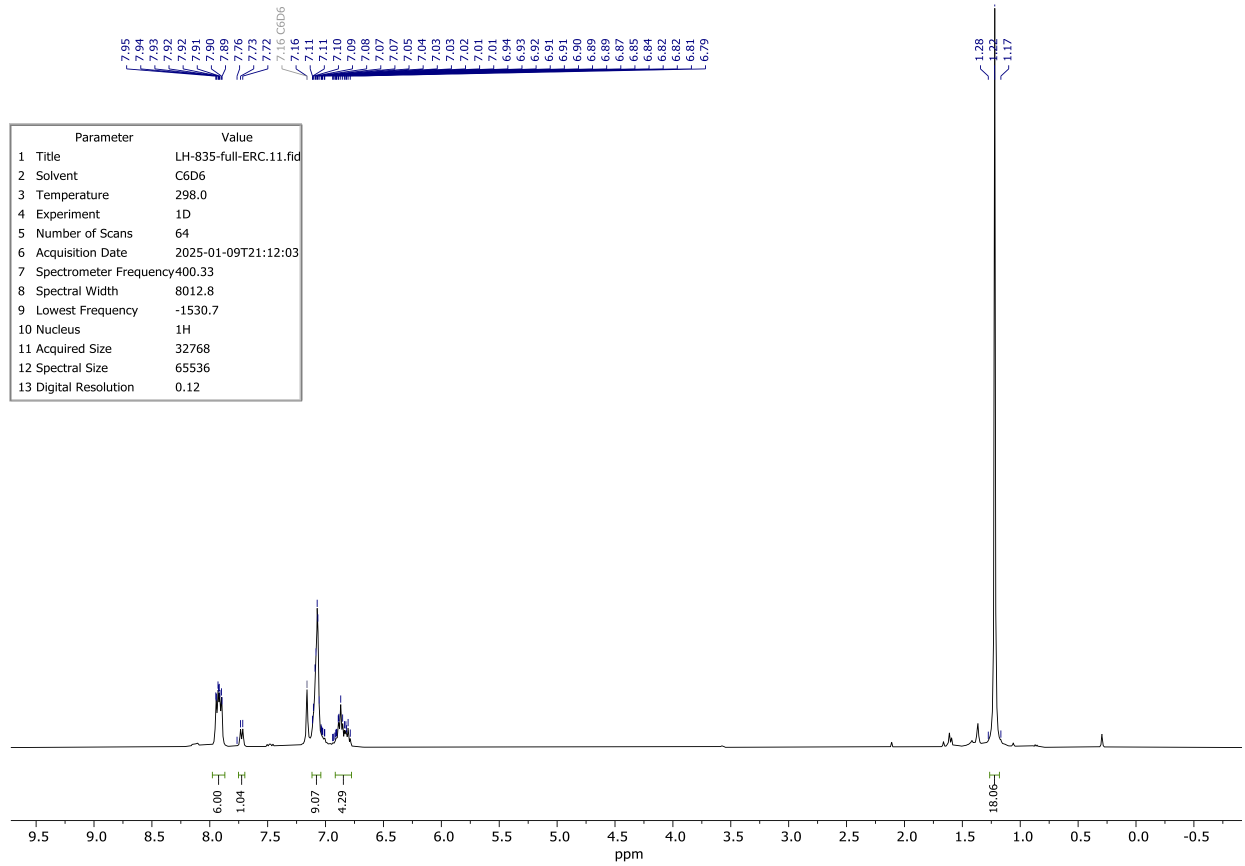

Figure S59. ^{1}H-NMR spectrum of **8** in C_6D_6.

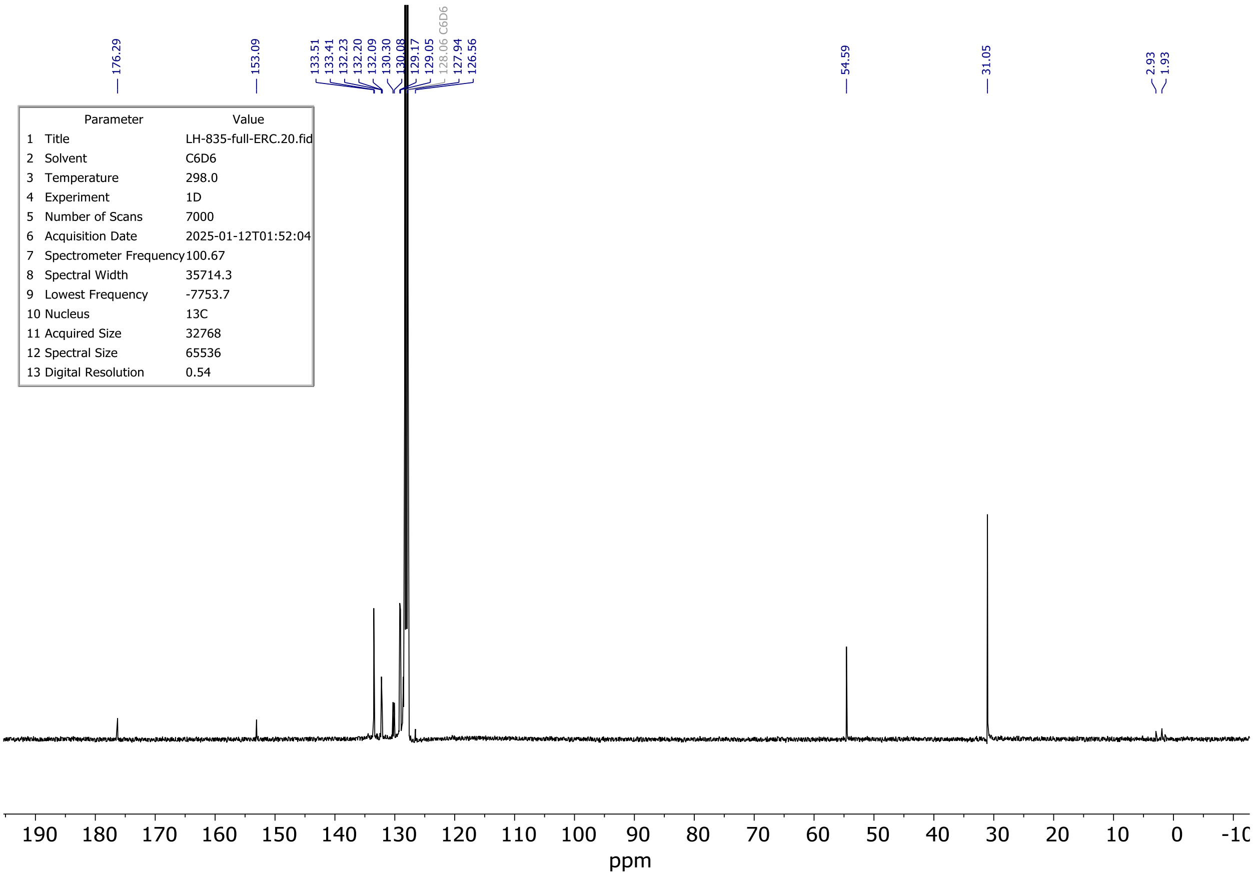

Figure S60: $^{13}C\{^{1}H\}$-NMR spectrum of **8** in C_6D_6.

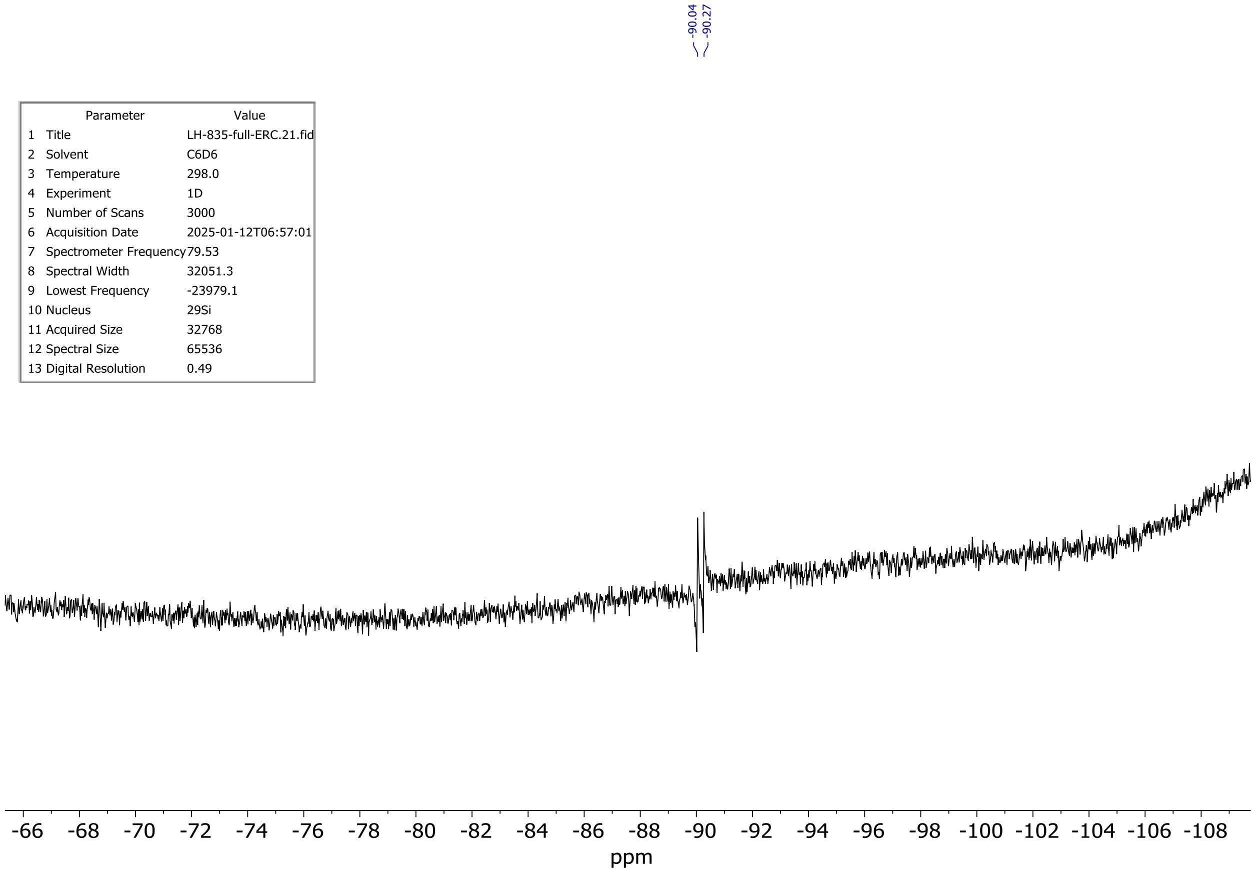

Figure S61: $^{29}Si\{^{1}H\}$-NMR spectrum of **8** in C_6D_6.

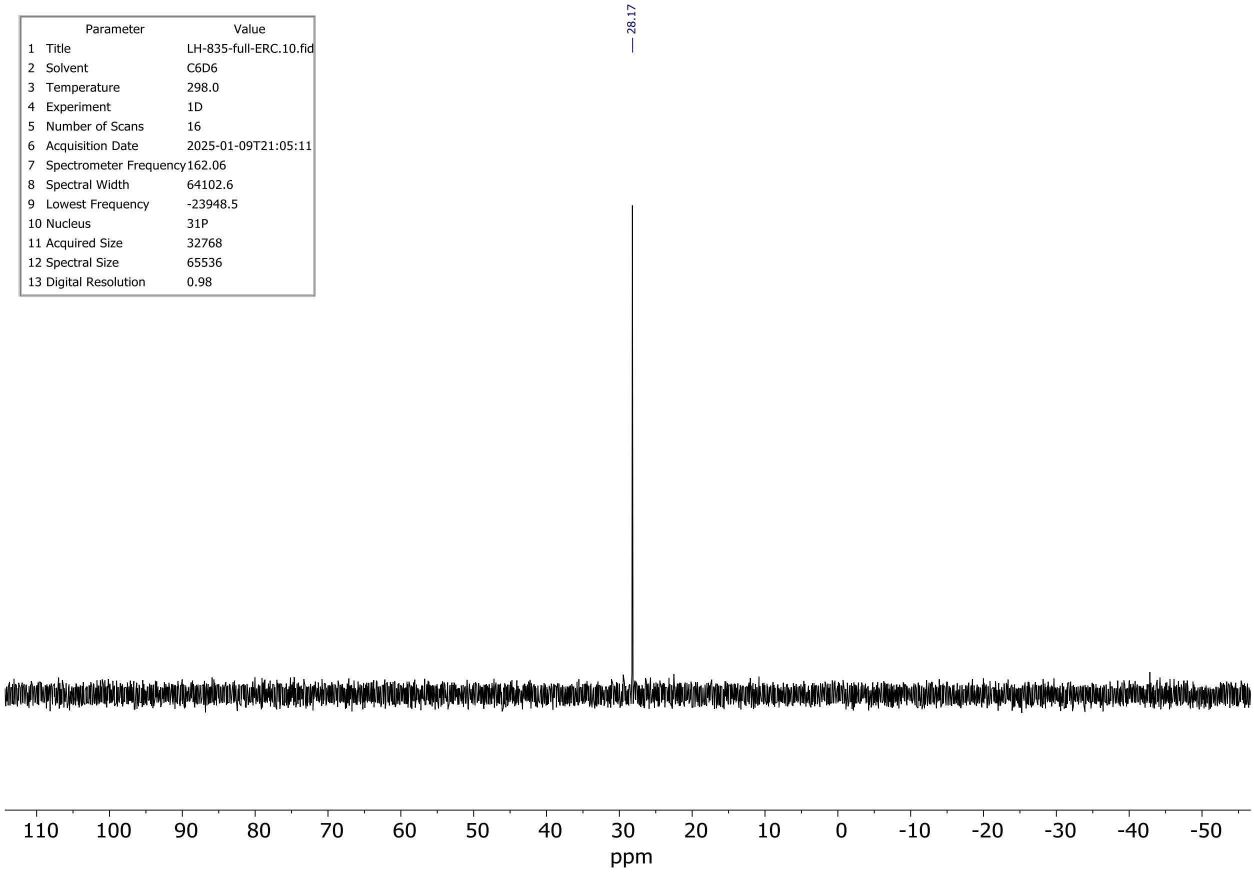

Figure S62: $^{31}P\{^{1}H\}$-NMR spectrum of **8** in C_6D_6.

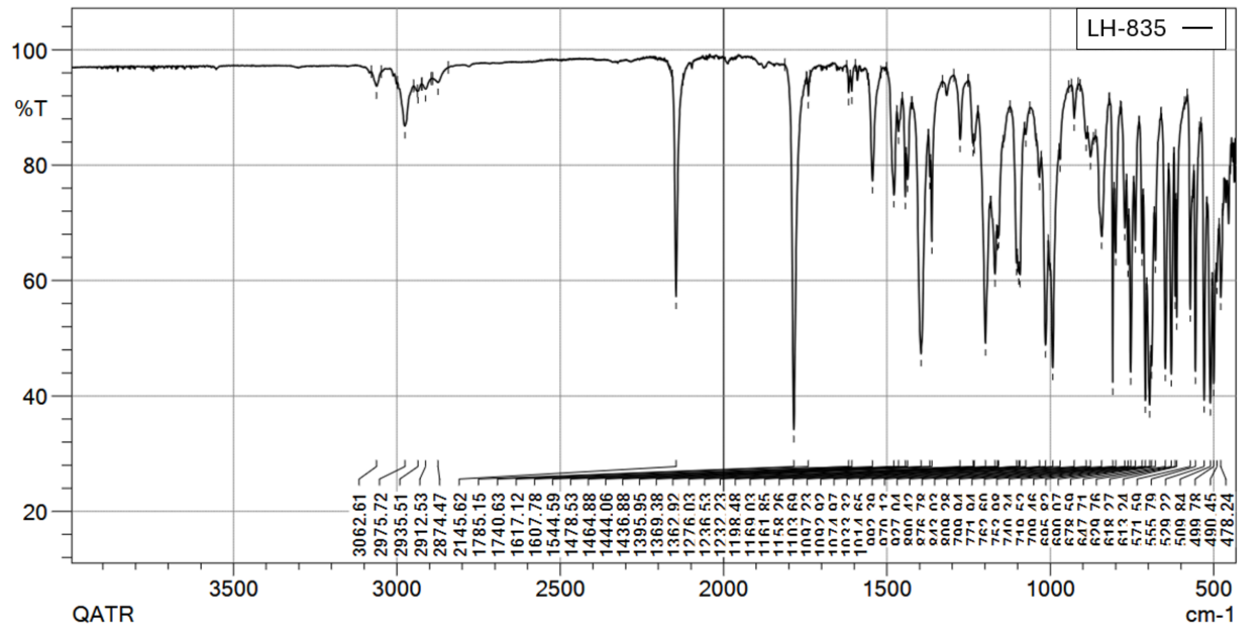

Figure S63: IR spectrum of solid **8**.

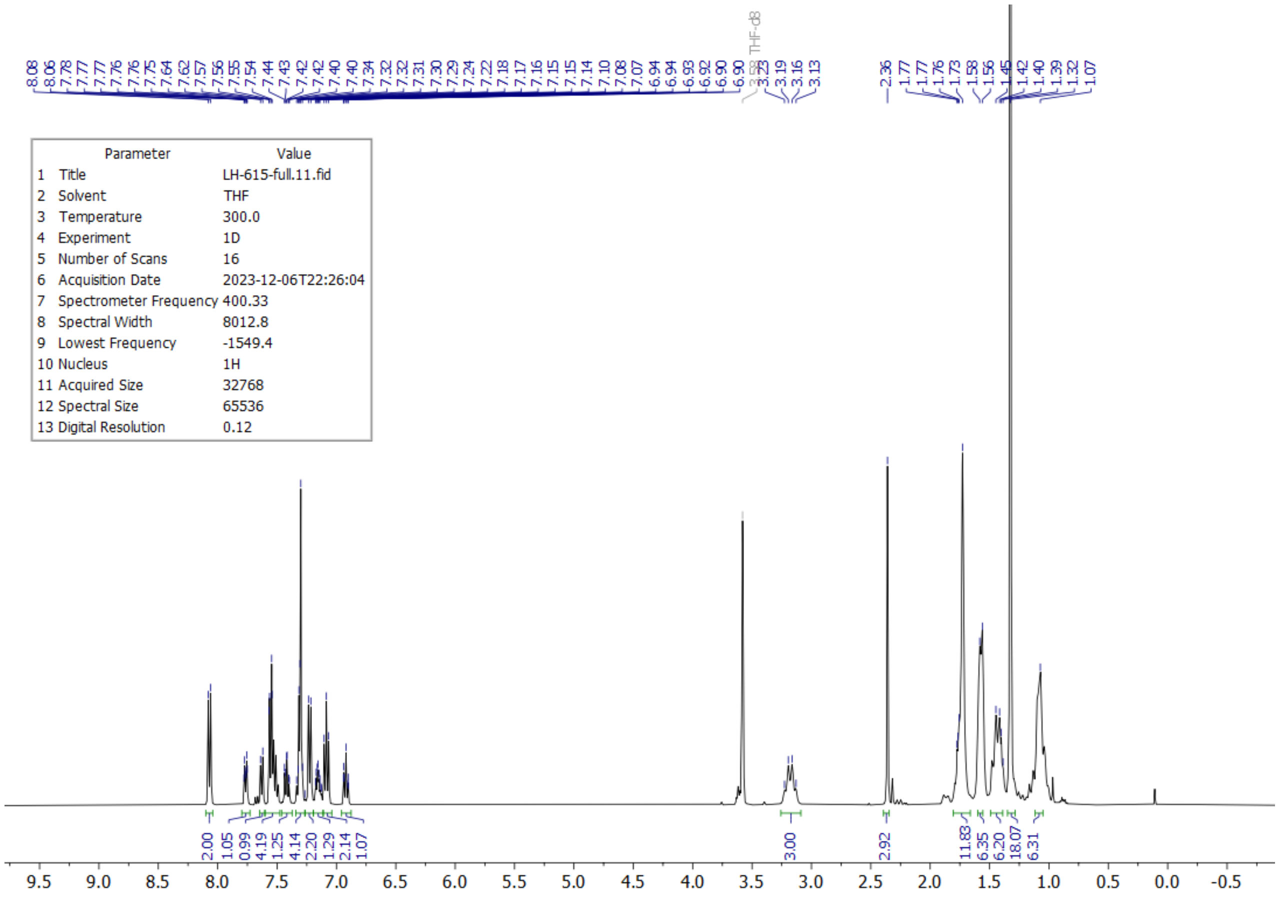

Figure S64: ^{1}H-NMR spectrum of $\mathbf{9_{Ts}}$ in THF-d_8.

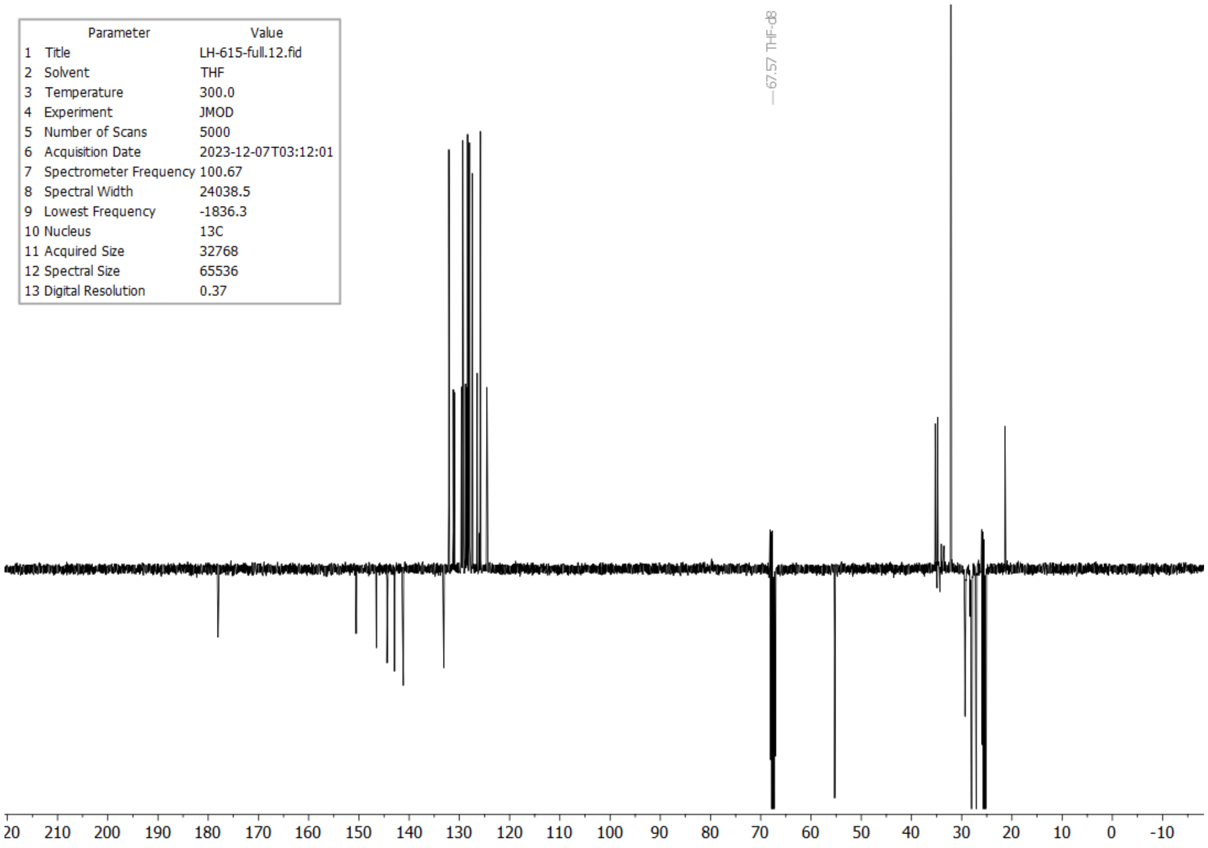

Figure S65: $^{13}C\{^{1}H\}$-APT-NMR spectrum of **9**$_{\textbf{Ts}}$ in THF-d_8.

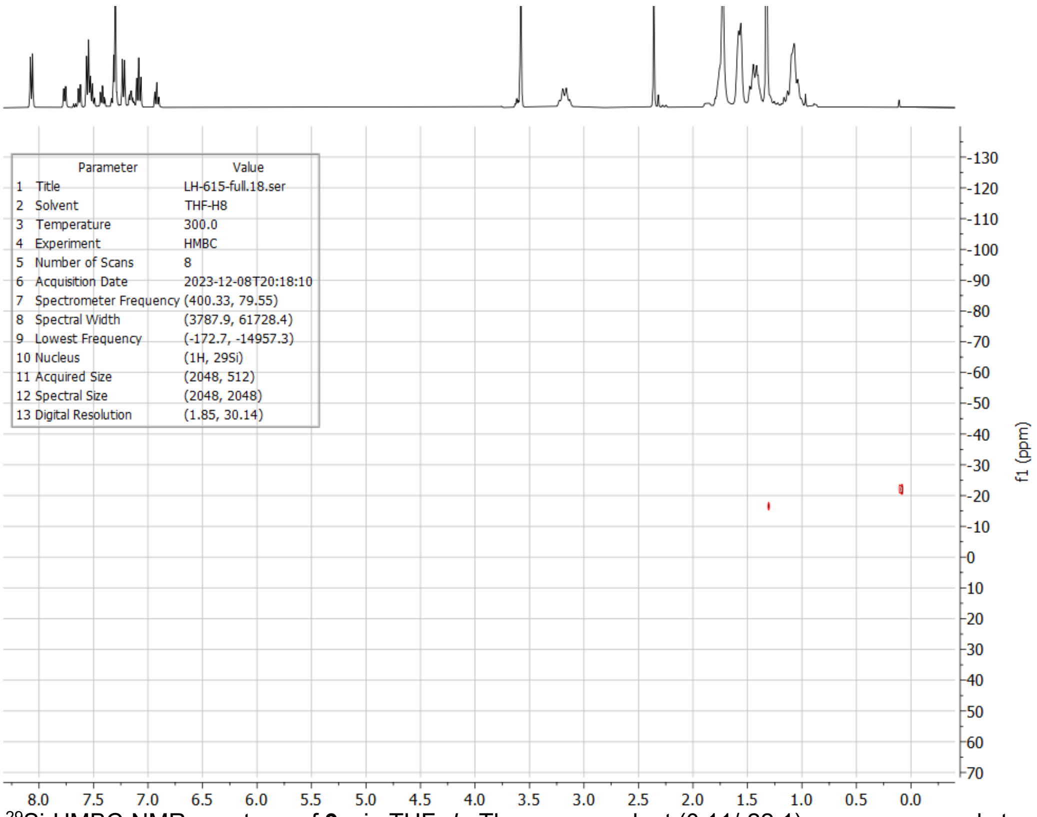

Figure S66: ^{1}H-^{29}Si-HMBC-NMR spectrum of **9**$_{\textbf{Ts}}$ in THF-d_8. The cross-peak at (0.11/-22.1) ppm corresponds to residual silicon grease.

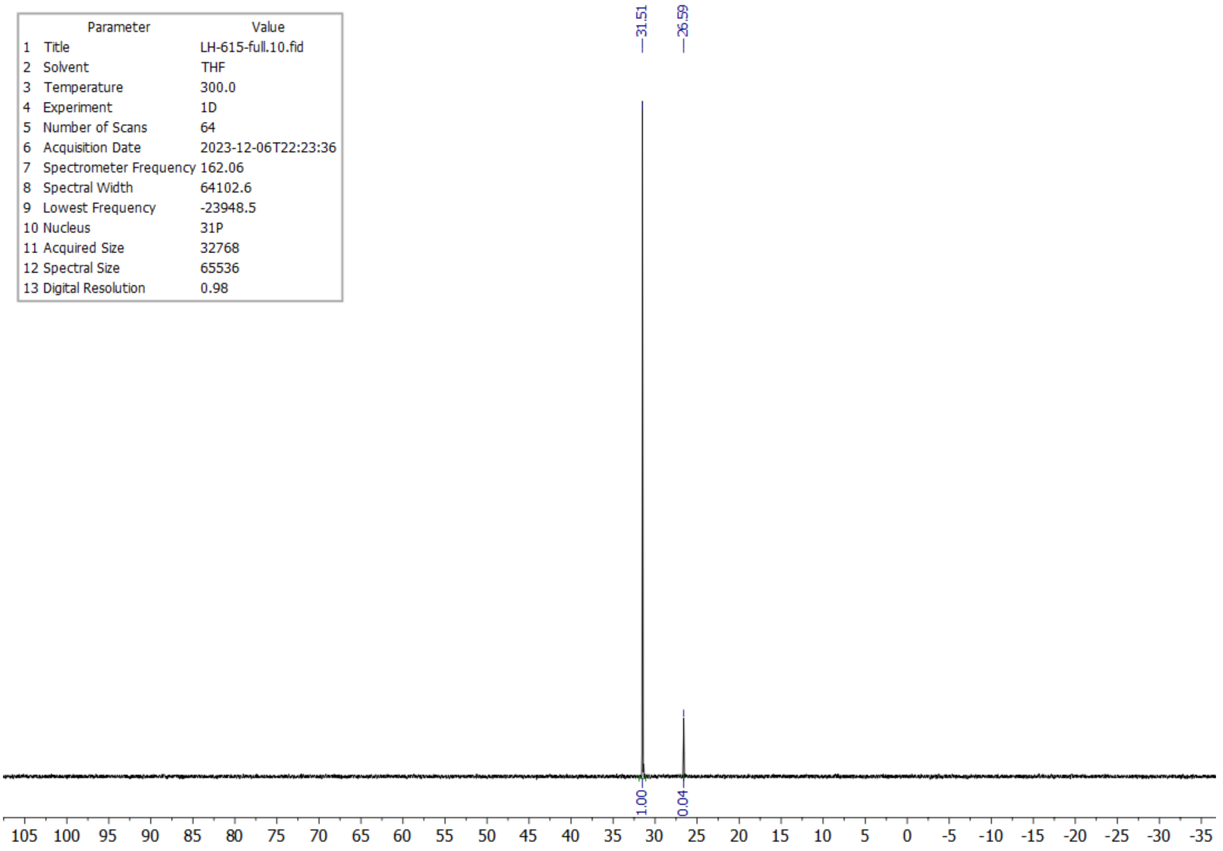

Figure S67: $^{31}P\{^{1}H\}$-NMR spectrum of **9**$_{\mathbf{Ts}}$ in THF-d_8. The peak at 26.6 ppm corresponds to free ylide.

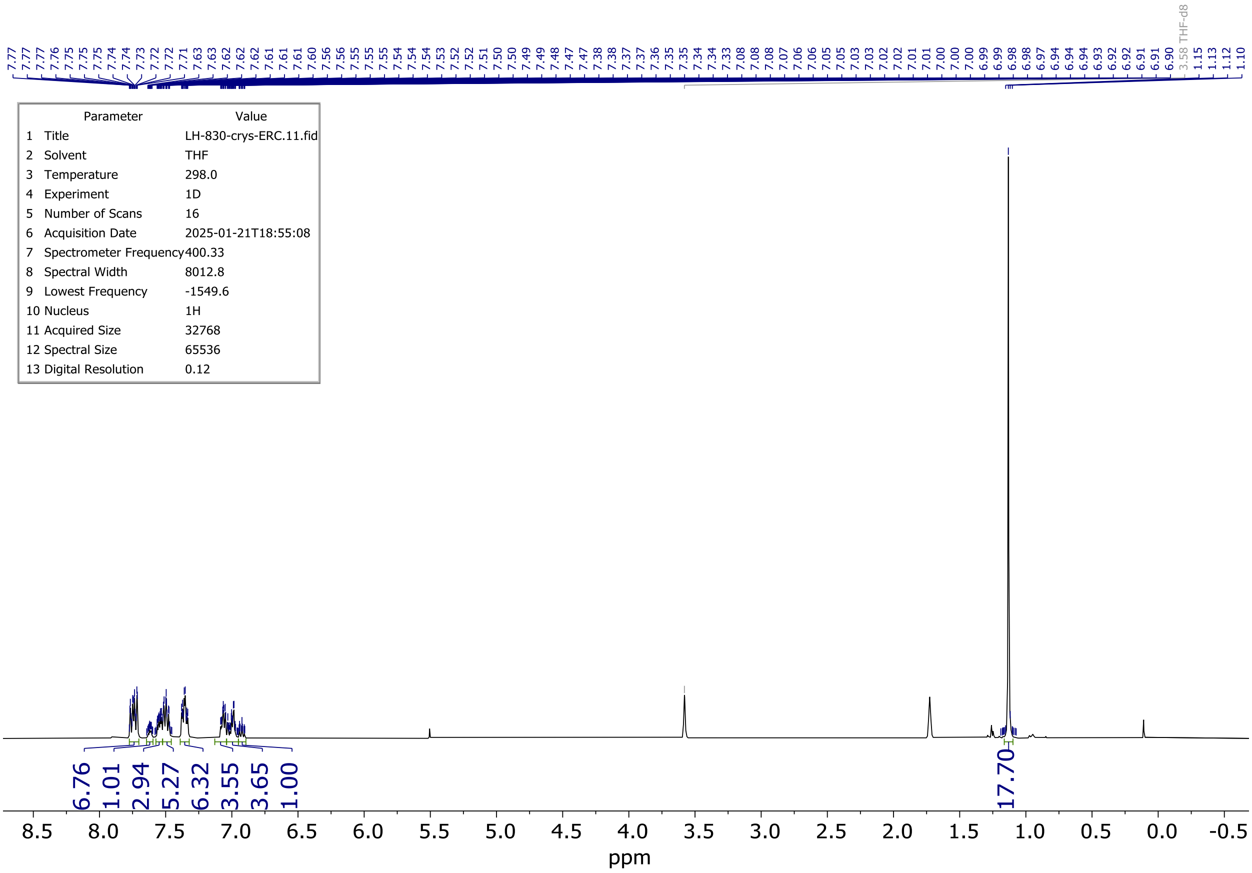

Figure S68: ^{1}H-NMR spectrum of **9**$_{\mathbf{CN}}$ in THF-d_8.

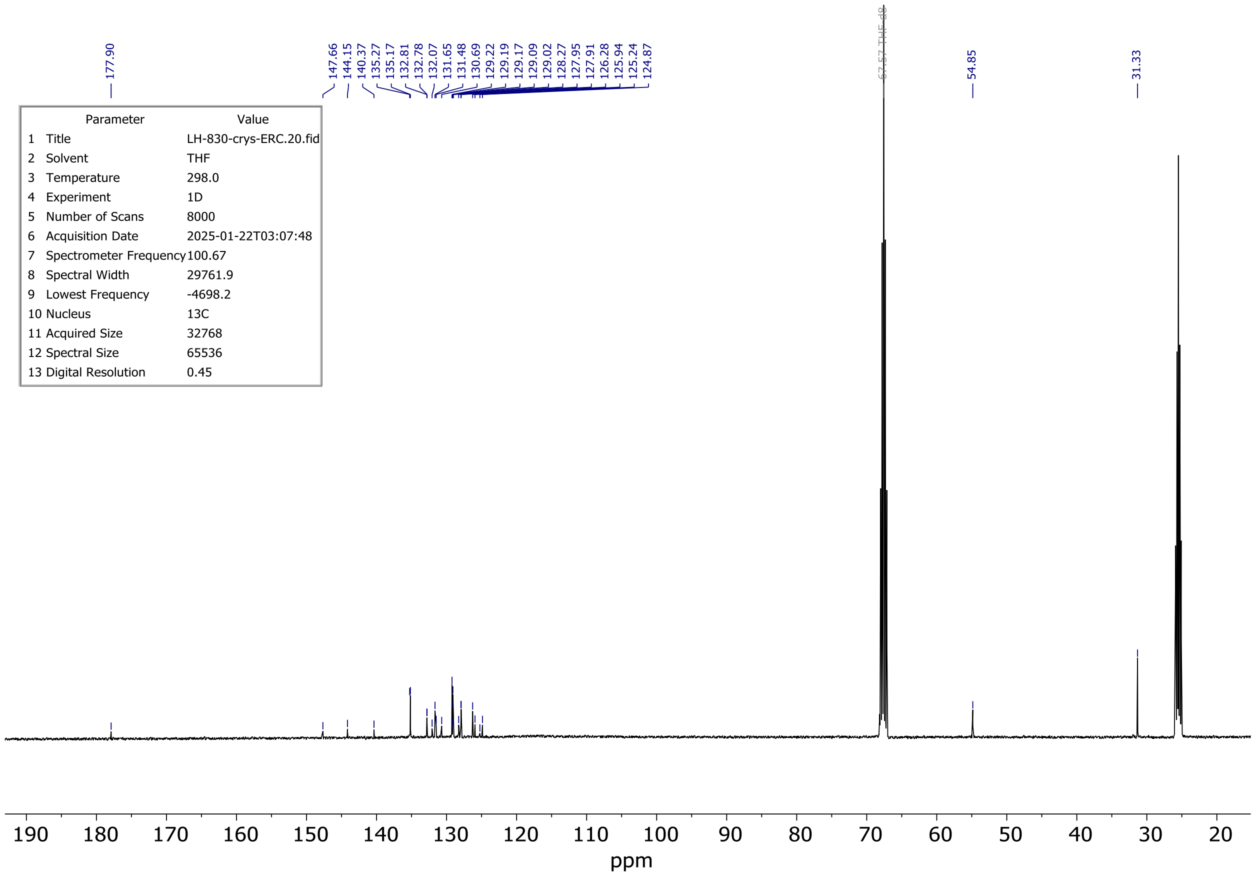

Figure S69: $^{13}C\{^1H\}$-NMR spectrum of $\mathbf{9_{CN}}$ in THF-d_8.

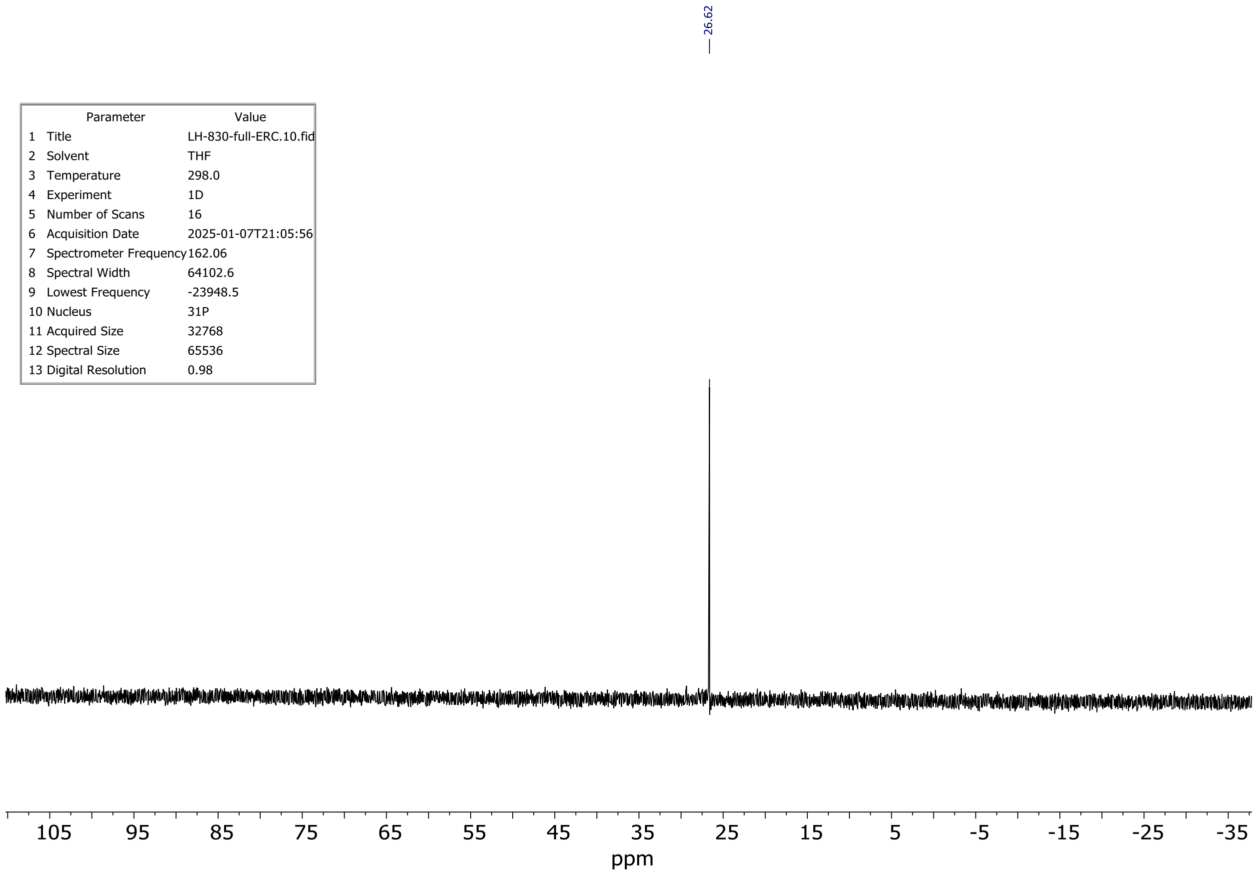

Figure S70: $^{31}P\{^1H\}$-NMR spectrum of $\mathbf{9_{CN}}$ in THF-d_8.

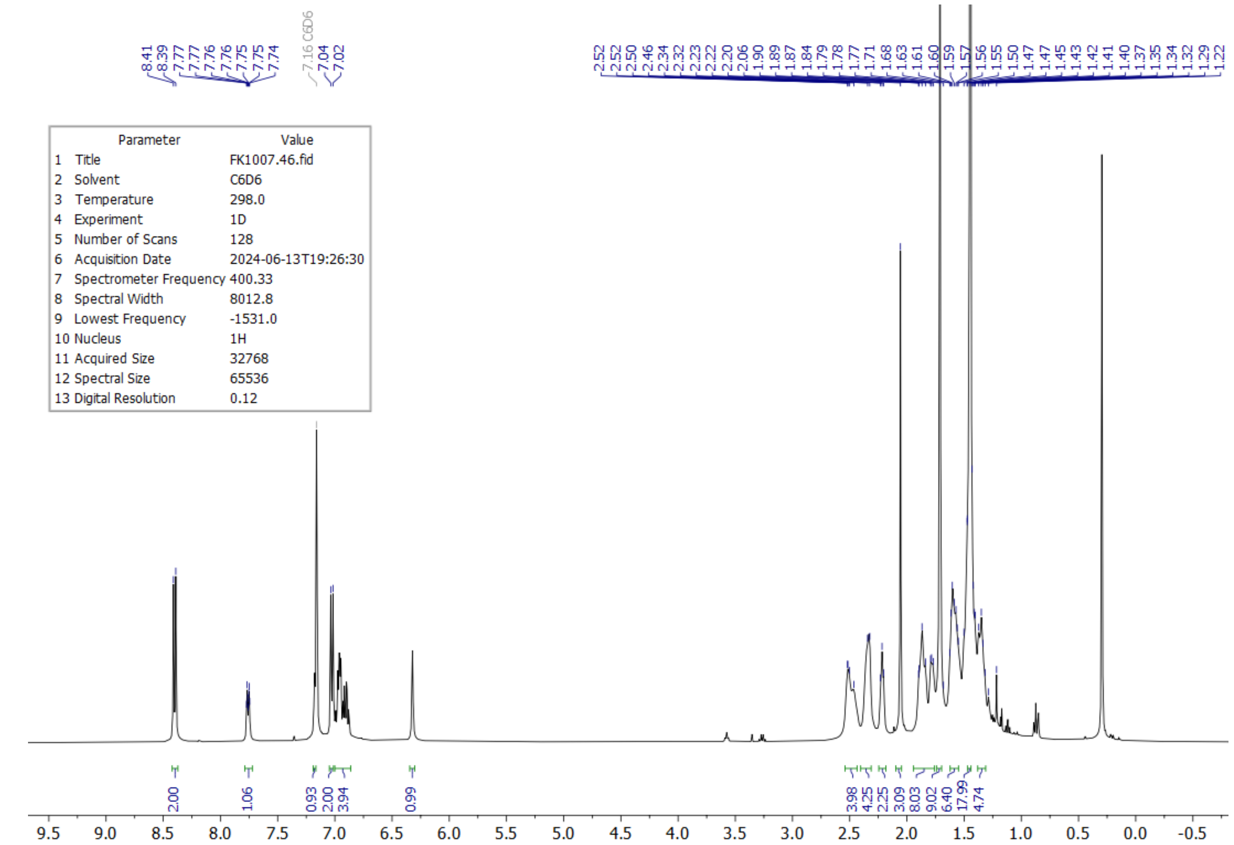

Figure S71. ^{1}H-NMR spectrum of **10** in C_6D_5- The peak at 0.26 ppm corresponds to silicon grease that could not be removed due to similar solubility.

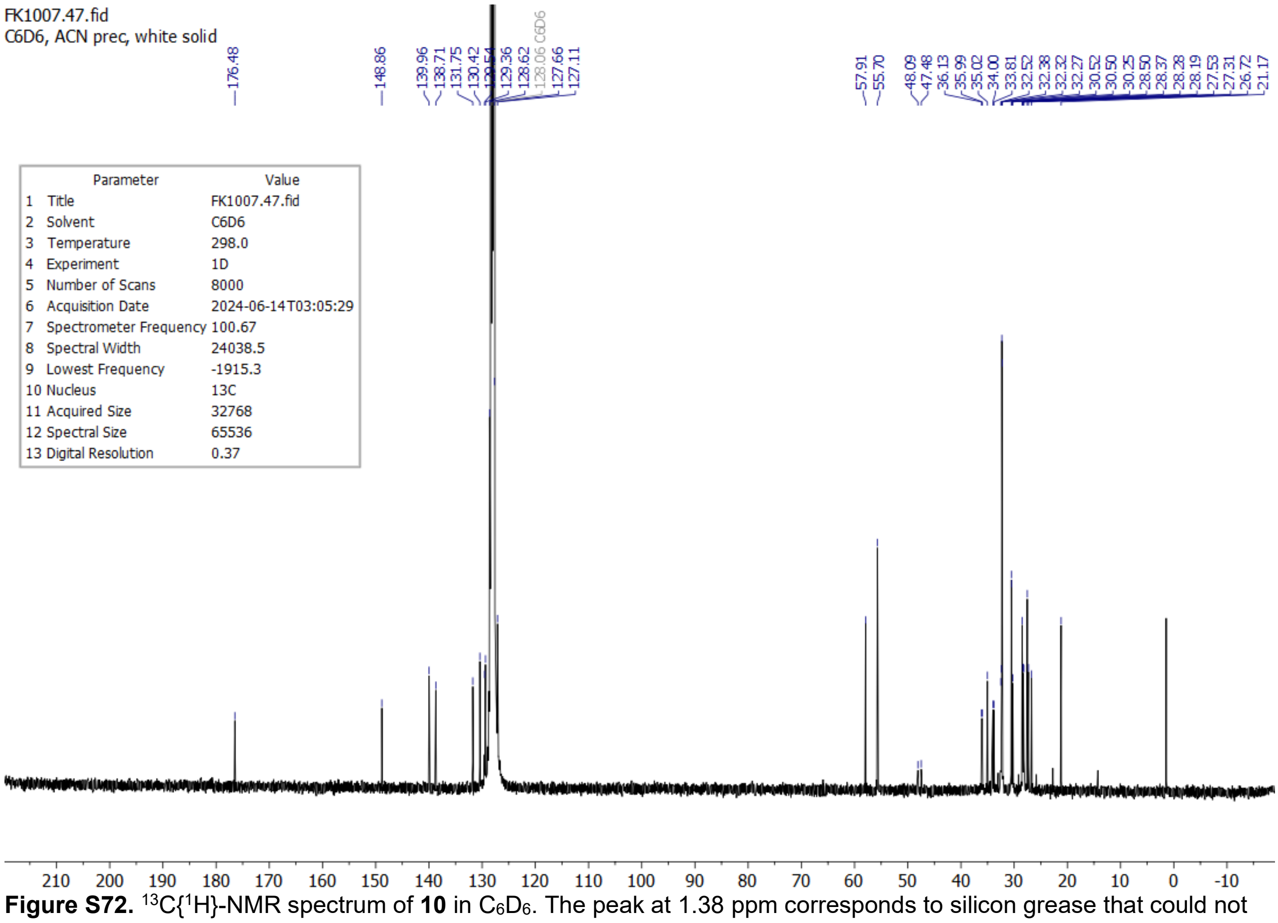

Figure S72. $^{13}C\{^{1}H\}$-NMR spectrum of **10** in C_6D_6. The peak at 1.38 ppm corresponds to silicon grease that could not be removed due to similar solubility.

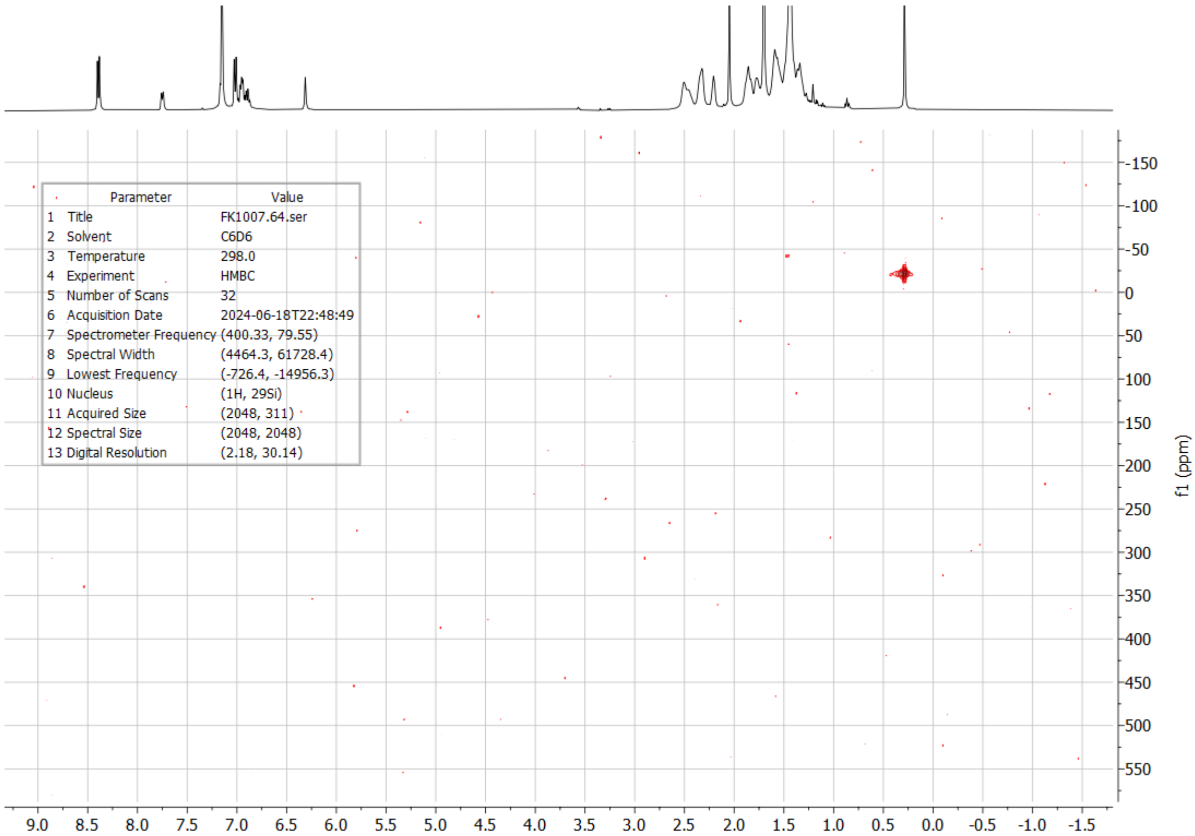

Figure S73. ^{1}H-^{29}Si-HMBC spectrum of **9** in C_6D_6. The peak at (0.29/-21.7) ppm corresponds to residual silicon grease that could not be removed due to similar solubility. The low intensity of the signal might result from the long relaxation time of **10**.

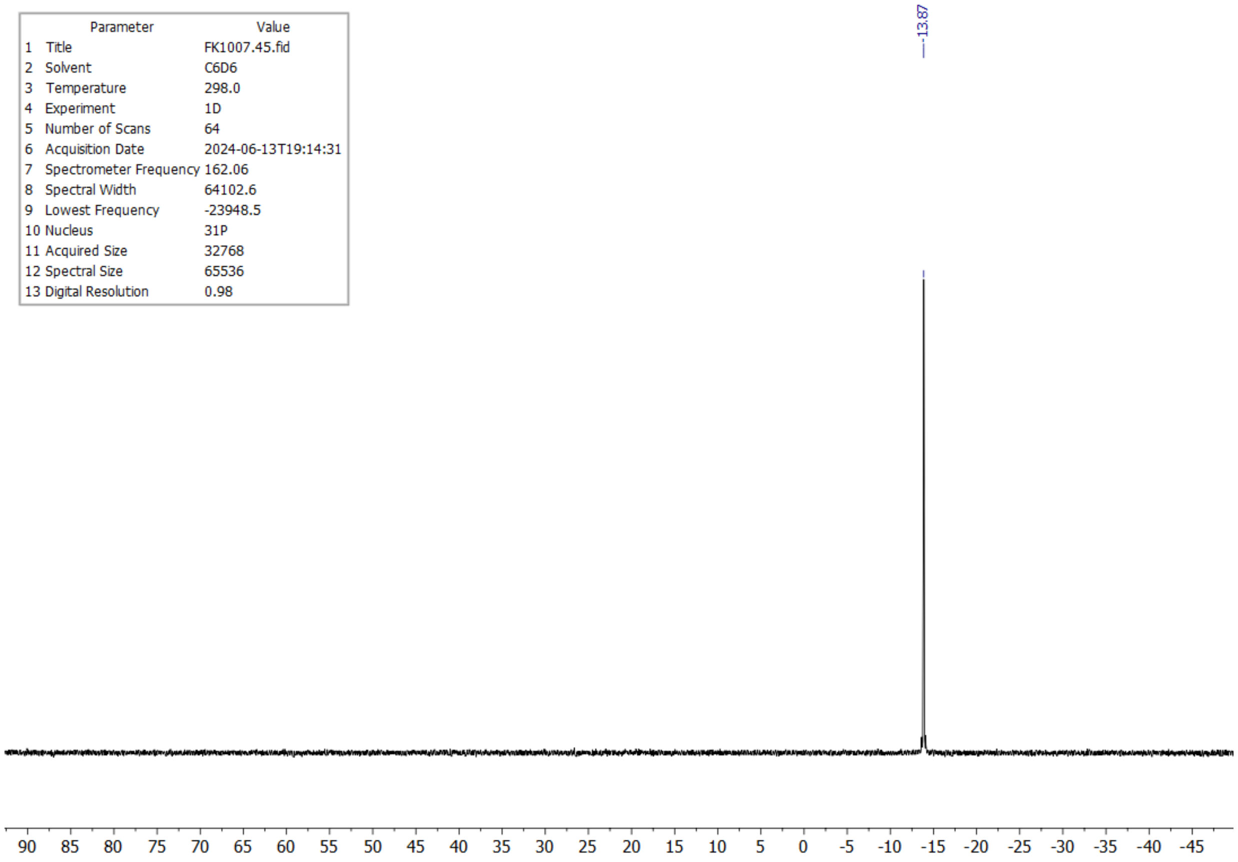

Figure S74. $^{31}P\{^{1}H\}$-NMR spectrum of **10** in C_6D_6.

6 Crystal Structure Analyses

6.1 Data collection and structure refinement details

Table S1 Data collection and structure refinement details for compounds **1**, **AYSi-2**, **AYSi-3** and **5_{Ts}**.

**Compound**	**2**	**AYSi-2**	**AYSi-3**	**5_{Ts}**
Formula	$C_{41}H_{45}N_2O_2P_1S_1Si_1$	$C_{41}H_{63}N_2O_2P_1S_1Si_1$	$C_{73}H_{79}N_6P_2Si_2$	$C_{42}H_{63}N_2O_2P_1S_3Si_1$
CCDC	2350309	2350308	2350310	2366468
Formula weight	688.91	707.05	1158.54	783.18
Temperature [K]	100(2)	100(2)	100(2)	100(2)
Wave length [Å]	1.54184	1.54184	1.54184	1.54184
Crystal system	monoclinic	triclinic	monoclinic	monoclinic
Space group	*P*21	*P*-1	*C*2/*c*	*P*21/*c*
a [Å]	9.86550(10)	9.87940(10)	64.6062(10)	13.63720(10)
b [Å]	10.66970(10)	9.96850(10)	10.33540(10)	14.83500(10)
c [Å]	17.54370(10)	21.1954(3)	19.79680(10)	20.8762(2)
α[°]	90	103.2980(10)	90	90
β [°]	94.8050(10)	98.3310(10)	94.6640(10)	100.6410(10)
γ [°]	90	90.0900(10)	90	90
Volumen [$Å^3$]	1840.19(3)	2008.66(4)	13175.16(17)	4150.79(6)
Z	2	2	8	4
Calc. density [$Mg \cdot m^{-3}$]	1.243	1.169	1.168	1.253
μ (MoKα) [mm^{-1}]	1.792	1.643	1.297	2.557
F(000)	732	768	4936	1688
Crystal dimensions [mm]	0.210 x 0.120 x 0.0 70	0.220 x 0.130 x 0.060	0.404 x 0.135 x 0.100	0.149 x 0.112 x 0.052
Theta range θ [°]	2.527 to 67.984	4.335 to 67.996	2.745 to 78.241	3.297 to 67.073
Index ranges	-11 ≤ h ≤ 11	-9 ≤ h ≤ 11	-79 ≤ h ≤ 79	-16 ≤ h ≤ 16
	-12 ≤ k ≤ 12	-11 ≤ k ≤ 11	-12 ≤ k ≤ 13	-17 ≤ k ≤ 17
	-21 ≤ l ≤ 20	-25 ≤ l ≤ 22	-24 ≤ l ≤ 24	-24 ≤ l ≤ 24
Reflections collected	53970	24161	13797	54915
Independent reflections	6680 [*R*(int) = 0.0240]	7289 [*R*(int) = 0.0349]	13797 [R(int) = 0.434]	7406 [*R*(int) = 0.0604]
Data/Restraints/Paramet er	6680/1/444	7289/0/443	13797/0/761	7406/0/467
Goodness-of-fit on F^2	1.014	1.055	1.153	1.051
Final R indices [I>2sigma(I)]	*R*1 = 0.0276, w*R*2 = 0.0731	*R*1 = 0.0358, w*R*2 = 0.0916	R1 = 0.0631, wR2 = 0.1857	*R*1 = 0.0387, w*R*2 = 0.1054
Largest diff. peak and hole [$e \cdot Å^{-3}$]	0.426 and -0.259	0.496 and -0.355	0.966 and -0.463	0.501 and -0.477

Table S2 Data collection and structure refinement details for compounds **5**, **6**, and **7**.

Compound	5_{CN}	6	7	8
Formula	$C_{36}H_{38}N_3P_1S_2Si_1$	$C_{47}H_{69}N_2O_3P_1S_1Si_1$	$C_{94}H_{100}N_6O_2P_2Si_2$	$C_{36}H_{38}N_3O_3P_1Si_1$
CCDC	2366469	2350311	2350312	2429366
Formula weight	635.87	801.16	1463.91	619.75
Temperature [K]	100(2)	100(2)	100(2)	100(2)
Wave length [Å]	1.54184	1.54184	1.54184	1.54184
Crystal system	monoclinic	triclinic	monoclinic	monoclinic
Space group	*P*21/*c*	*P*-1	*I*2/*a*	$P2_1$/n
a [Å]	14.81430(10)	10.2796(2)	19.82520(10)	11.78680(10)
b [Å]	14.40780(10)	12.9114(2)	16.08220(10)	18.12460(10)
c [Å]	17.4032(2)	18.3044(2)	26.2606(2)	14.91140(10)
α[°]	90	74.2670(10)	90	90
β [°]	114.3500(10)	77.1070(10)	96.7770(10)	98.0340(10)
γ [°]	90	69.783(2)	90	90
Volumen [$Å^3$]	3384.13(6)	2171.70(7)	8314.24(9)	3154.27(4)
Z	4	2	4	4
Calc. density [$Mg\cdot m^{-3}$]	1.248	1.225	1.170	1.305
μ (MoKα) [mm^{-1}]	2.431	1.598	1.148	1.464
F(000)	3120	868	3120	1312
Crystal dimensions [mm]	0.207 x 0.143 x 0.078	0.464 x 0.266 x 0.182	0.446 x 0.326 x 0.313	0.265 x 0.108 x 0.086
Theta range θ [°]	3.275 to 76.881	2.534 to 67.080	3.229 to 67.068	3.861 to 76.622
Index ranges	-18 ≤ h ≤ 18	-12 ≤ h ≤ 12	-23 ≤ h ≤ 22	-14 ≤ h ≤ 14
	-18 ≤ k ≤ 15	-15 ≤ k ≤ 15	-19 ≤ k ≤ 19	-15 ≤ k≤ 21
	-21 ≤ l ≤ 21	-19 ≤ l ≤ 21	-30 ≤ l ≤ 31	18 ≤ l≤ 18
Reflections collected	45900	71149	53814	23088
Independent reflections	7005 [R(int) = 0.0409]	7735 [*R*(int) = 0.0665]	7425 [R(int) = 0.0608]	6321 [R(int) = 0.0315]
Data/Restraints/Parameter	7005/0/394	7735/0/506	7425/0/515	6321/0/403
Goodness-of-fit on F^2	1.051	1.051	1.038	1.054
Final R indices [I>2sigma(I)]	R1 = 0.0326, wR2 = 0.0893	*R*1 = 0.0322, w*R*2 = 0.0821	R1 = 0.0405, wR2 = 0.1098	R1 = 0.0341, wR2 = 0.0895
Largest diff. peak and hole [$e\cdot Å^{-3}$]	0.413 and -0.460	0.302 and -0.386	0.533 and -0.387	0.306 and -0.343

Table S3 Data collection and structure refinement details for compounds $\mathbf{8_{Ts}}$, $\mathbf{8_{CN}}$, and **9**.

**Compound**	$\mathbf{9_{Ts}}$	$\mathbf{9_{CN}}$	**10**
Formula	$C_{61}H_{81}N_4O_2P_1S_1Si_1$	$C_{48}H_{48}N_5P_1Si_1$	$C_{46}H_{72}N_3O_2P_1S_1Si$
CCDC	2366470	2366471	2366472
Formula weight	993.41	753.97	790.18
Temperature [K]	105(2)	109(2)	100(2)
Wave length [Å]	1.54184	1.54184	1.54184
Crystal system	triclinic	monoclinic	monoclinic
Space group	*P*-1	*P*21/*c*	*P*21/*n*
a [Å]	9.95910(10)	20.4704(2)	11.42850(10)
b [Å]	12.80600(10)	8.81830(10)	27.55120(10)
c [Å]	22.0685(2)	23.8065(2)	14.39760(10)
α[°]	76.2790(10)	90	90
β [°]	89.3570(10)	105.3050(10)	100.7750(10)
γ [°]	87.9160(10)	90	90
Volumen [Å^3]	2732.39(5)	4145.00(7)	4453.43(5)
Z	2	4	4
Calc. density [Mg·m^{-3}]	1.207	1.208	1.179
μ (MoKα) [mm^{-1}]	1.366	1.164	1.539
F(000)	1072	1600	1720
Crystal dimensions [mm]	0.060 x 0.050 x 0.040	0.310 x 0.160 x 0.080	0.170 x 0.130x 0.040
Theta range θ [°]	3.555 to 67.999	3.315 to 67.994	3.208 to 67.998
Index ranges	-11 ≤ h ≤ 11	-23 ≤ h ≤ 24	-13 ≤ h ≤ 13
	-14 ≤ k ≤ 15	-10 ≤ k ≤ 7	-30 ≤ k ≤ 33
	-26 ≤ l ≤ 26	-28 ≤ l ≤ 28	-17 ≤ l ≤ 17
Reflections collected	32168	31602	129827
Independent reflections	9892 [*R*(int) = 0.0319]	7555 [*R*(int) = 0.0402]	8107 [R(int) = 0.0687]
Data/Restraints/Parameter	9892/0/704	7555/0/502	8107/0/497
Goodness-of-fit on F^2	1.027	1.022	1.038
Final R indices [I>2sigma(I)]	*R*1 = 0.0345, w*R*2 = 0.0874	*R*1 = 0.0349, w*R*2 = 0.0879	R1 = 0.0330, wR2 = 0.0866
Largest diff. peak and hole [e·Å^{-3}]	0.403 and -0.364	0.348 and -0.306	0.297 and -0.396

6.2 Crystal structure determination of 2

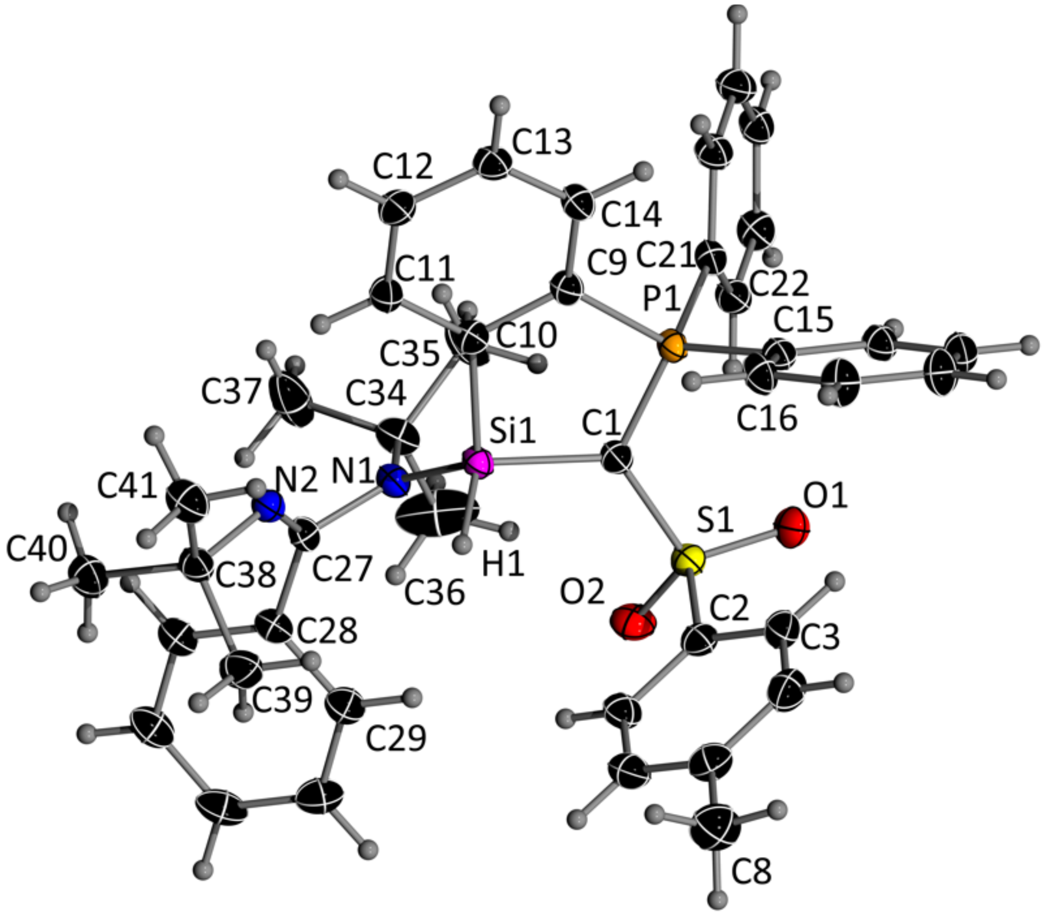

Figure S75. ORTEP of **2**. Thermal ellipsoids are drawn at 50% probability level.

Table S4. Atomic coordinates (x 10^4) and equivalentisotropic displacement parameters ($Å^2$x 10^3) for **2**. U(eq) is defined as one third of the trace of the orthogonalized U^{ij} tensor for all atoms.

Atom	X	Y	Z	U(eq)
S1	0.29976(5)	0.71674(5)	0.67519(3)	0.02127(13)
P1	0.59015(5)	0.64291(5)	0.67962(3)	0.01850(13)
Si1	0.40822(6)	0.47376(6)	0.75812(3)	0.01873(14)
O1	0.35305(17)	0.83292(16)	0.64679(10)	0.0279(4)
N1	0.35636(19)	0.4750(2)	0.85117(11)	0.0215(4)
C1	0.4299(2)	0.6247(2)	0.70945(13)	0.0206(5)
O2	0.19962(17)	0.72343(18)	0.73103(10)	0.0291(4)
N2	0.3250(2)	0.26407(19)	0.83833(11)	0.0218(4)
C2	0.2146(2)	0.6411(2)	0.59442(14)	0.0234(5)
C3	0.2493(3)	0.6668(2)	0.52099(15)	0.0274(5)
C4	0.1841(3)	0.6033(3)	0.45886(15)	0.0315(6)
C5	0.0829(3)	0.5144(3)	0.46905(15)	0.0305(6)
C6	0.0497(2)	0.4909(3)	0.54285(15)	0.0293(5)
C7	0.1134(2)	0.5524(2)	0.60550(15)	0.0257(5)
C8	0.0132(3)	0.4446(3)	0.40244(17)	0.0420(7)
C9	0.6622(2)	0.4903(2)	0.70088(13)	0.0200(5)
C10	0.5827(2)	0.4115(2)	0.74327(13)	0.0206(5)
C11	0.6348(2)	0.2926(2)	0.76334(14)	0.0236(5)
C12	0.7591(2)	0.2536(2)	0.73929(15)	0.0265(5)
C13	0.8332(2)	0.3319(2)	0.69464(14)	0.0243(5)
C14	0.7855(2)	0.4510(2)	0.67587(14)	0.0225(5)
C15	0.6076(2)	0.6744(2)	0.57970(14)	0.0210(5)
C16	0.5952(3)	0.5759(2)	0.52738(15)	0.0265(5)
C17	0.6054(3)	0.5981(3)	0.45041(15)	0.0303(6)
C18	0.6270(3)	0.7195(3)	0.42530(14)	0.0296(5)
C19	0.6399(3)	0.8179(2)	0.47675(15)	0.0268(5)
C20	0.6306(2)	0.7965(2)	0.55435(14)	0.0229(5)
C21	0.6949(2)	0.7582(2)	0.73357(13)	0.0215(5)

C22	0.8368(2)	0.7481(2)	0.74251(15)	0.0248(5)
C23	0.9134(2)	0.8317(3)	0.78876(15)	0.0271(5)
C24	0.8495(3)	0.9267(2)	0.82596(15)	0.0255(5)
C25	0.7093(3)	0.9393(2)	0.81595(14)	0.0261(5)
C26	0.6320(2)	0.8549(2)	0.77018(15)	0.0247(5)
C27	0.2794(2)	0.3662(2)	0.86451(13)	0.0208(5)
C28	0.1448(2)	0.3872(2)	0.89702(14)	0.0223(5)
C29	0.0489(2)	0.4619(3)	0.85558(14)	0.0267(5)
C30	-0.0806(3)	0.4770(3)	0.87903(15)	0.0314(5)
C31	-0.1155(3)	0.4182(3)	0.94560(16)	0.0327(6)
C32	-0.0191(3)	0.3483(3)	0.98909(15)	0.0307(6)
C33	0.1104(2)	0.3325(2)	0.96489(14)	0.0258(5)
C34	0.3989(2)	0.5622(2)	0.91563(14)	0.0260(5)
C35	0.5310(3)	0.6256(3)	0.89958(15)	0.0352(6)
C36	0.2915(4)	0.6606(4)	0.9266(2)	0.0619(11)
C37	0.4283(4)	0.4858(4)	0.98912(16)	0.0503(9)
C38	0.2487(2)	0.1447(2)	0.82389(14)	0.0235(5)
C39	0.1214(3)	0.1666(3)	0.76962(17)	0.0329(6)
C40	0.2115(3)	0.0774(3)	0.89625(16)	0.0317(6)
C41	0.3457(3)	0.0597(3)	0.78505(17)	0.0320(6)

Table S5. Anisotropic displacement parameters(Å^2) for **2**. The anisotropic displacement factor exponent takes the form: $-2\pi^2[\ h^2a^{*2}U^{11} + ... + 2\ h\ k\ a^*\ b^*\ U^{12}]$.

atom	U^{11}	U^{22}	U^{33}	U^{23}	U^{13}	U^{12}
S1	0.0193(3)	0.0169(3)	0.0281(3)	-0.0006(2)	0.0045(2)	0.0023(2)
P1	0.0178(3)	0.0147(3)	0.0236(3)	0.0001(2)	0.0051(2)	0.0002(2)
Si1	0.0174(3)	0.0166(3)	0.0228(3)	-0.0001(2)	0.0052(2)	-0.0001(2)
O1	0.0291(9)	0.0173(8)	0.0372(10)	0.0021(7)	0.0024(7)	0.0009(7)
N1	0.0200(9)	0.0195(9)	0.0257(10)	-0.0004(8)	0.0055(7)	-0.0024(8)
C1	0.0195(11)	0.0171(11)	0.0255(11)	0.0002(9)	0.0050(9)	0.0011(9)
O2	0.0231(8)	0.0305(9)	0.0344(9)	-0.0042(8)	0.0075(7)	0.0047(7)
N2	0.0192(9)	0.0201(10)	0.0263(10)	0.0013(8)	0.0035(8)	-0.0002(8)
C2	0.0195(10)	0.0208(11)	0.0300(12)	0.0006(10)	0.0028(9)	0.0044(9)
C3	0.0264(12)	0.0239(13)	0.0322(13)	0.0058(10)	0.0036(10)	0.0026(10)
C4	0.0341(14)	0.0337(14)	0.0270(13)	0.0050(11)	0.0032(10)	0.0053(11)
C5	0.0281(13)	0.0301(14)	0.0328(13)	-	-	0.0050(10)
C6	0.0212(11)	0.0307(13)	0.0361(13)	-	0.0041(10)	-
C7	0.0214(11)	0.0278(13)	0.0288(12)	0.0003(10)	0.0066(9)	0.0002(10)
C8	0.0425(16)	0.0477(18)	0.0349(15)	-	-	-
C9	0.0203(10)	0.0162(11)	0.0236(11)	-0.0020(9)	0.0026(8)	-0.0003(9)
C10	0.0205(11)	0.0186(12)	0.0229(12)	-0.0025(9)	0.0034(9)	0.0012(9)
C11	0.0232(12)	0.0203(12)	0.0277(12)	0.0039(10)	0.0041(9)	0.0005(9)
C12	0.0232(12)	0.0211(12)	0.0353(14)	0.0018(10)	0.0035(10)	0.0046(9)
C13	0.0204(11)	0.0225(12)	0.0305(12)	-	0.0050(9)	0.0028(10)
C14	0.0220(11)	0.0204(12)	0.0259(12)	-0.0004(9)	0.0069(9)	-0.0016(9)
C15	0.0180(10)	0.0186(11)	0.0269(12)	0.0005(9)	0.0047(9)	0.0016(8)
C16	0.0301(13)	0.0178(12)	0.0323(13)	0.0000(10)	0.0066(10)	-
C17	0.0406(15)	0.0232(13)	0.0274(13)	-	0.0050(11)	-
C18	0.0371(13)	0.0298(13)	0.0227(12)	0.0032(11)	0.0068(10)	-
C19	0.0280(13)	0.0219(12)	0.0310(13)	0.0043(10)	0.0048(10)	-
C20	0.0220(12)	0.0186(12)	0.0285(13)	-0.0005(9)	0.0039(9)	-0.0010(9)
C21	0.0230(12)	0.0191(12)	0.0227(12)	0.0022(9)	0.0042(9)	-0.0022(9)
C22	0.0222(12)	0.0229(13)	0.0298(13)	-	0.0064(9)	0.0011(10)
C23	0.0201(11)	0.0283(13)	0.0331(13)	0.0001(11)	0.0039(10)	-
C24	0.0266(12)	0.0212(12)	0.0283(12)	0.0016(10)	0.0009(10)	-
C25	0.0278(12)	0.0200(12)	0.0310(13)	-	0.0063(10)	0.0016(9)
C26	0.0210(11)	0.0211(12)	0.0324(13)	0.0007(10)	0.0046(9)	-0.0005(9)
C27	0.0210(11)	0.0219(12)	0.0200(11)	0.0023(9)	0.0043(9)	-0.0012(9)
C28	0.0197(11)	0.0224(12)	0.0250(12)	-0.0031(9)	0.0042(9)	-0.0030(9)
C29	0.0248(12)	0.0286(13)	0.0270(12)	-	0.0043(9)	-
C30	0.0236(12)	0.0377(14)	0.0326(13)	-	0.0017(10)	0.0055(11)

C31	0.0218(12)	0.0433(16)	0.0342(14)	-	0.0085(10)	-
C32	0.0288(13)	0.0370(14)	0.0278(13)	-	0.0113(10)	-
C33	0.0238(12)	0.0270(13)	0.0272(12)	-	0.0056(9)	-
C34	0.0240(12)	0.0289(13)	0.0255(12)	-	0.0045(10)	-
C35	0.0387(15)	0.0398(16)	0.0278(13)	-	0.0063(11)	-
C36	0.0426(18)	0.063(2)	0.078(3)	-0.050(2)	-	0.0167(17)
C37	0.060(2)	0.062(2)	0.0276(14)	0.0058(15)	-	-
C38	0.0217(11)	0.0198(11)	0.0295(12)	0.0005(10)	0.0043(9)	-
C39	0.0284(13)	0.0276(14)	0.0418(15)	-	-	-
C40	0.0330(14)	0.0256(13)	0.0371(15)	0.0015(11)	0.0065(11)	-
C41	0.0311(13)	0.0248(13)	0.0410(15)	-	0.0087(11)	-

6.3 Crystal structure determination of AYSi-2

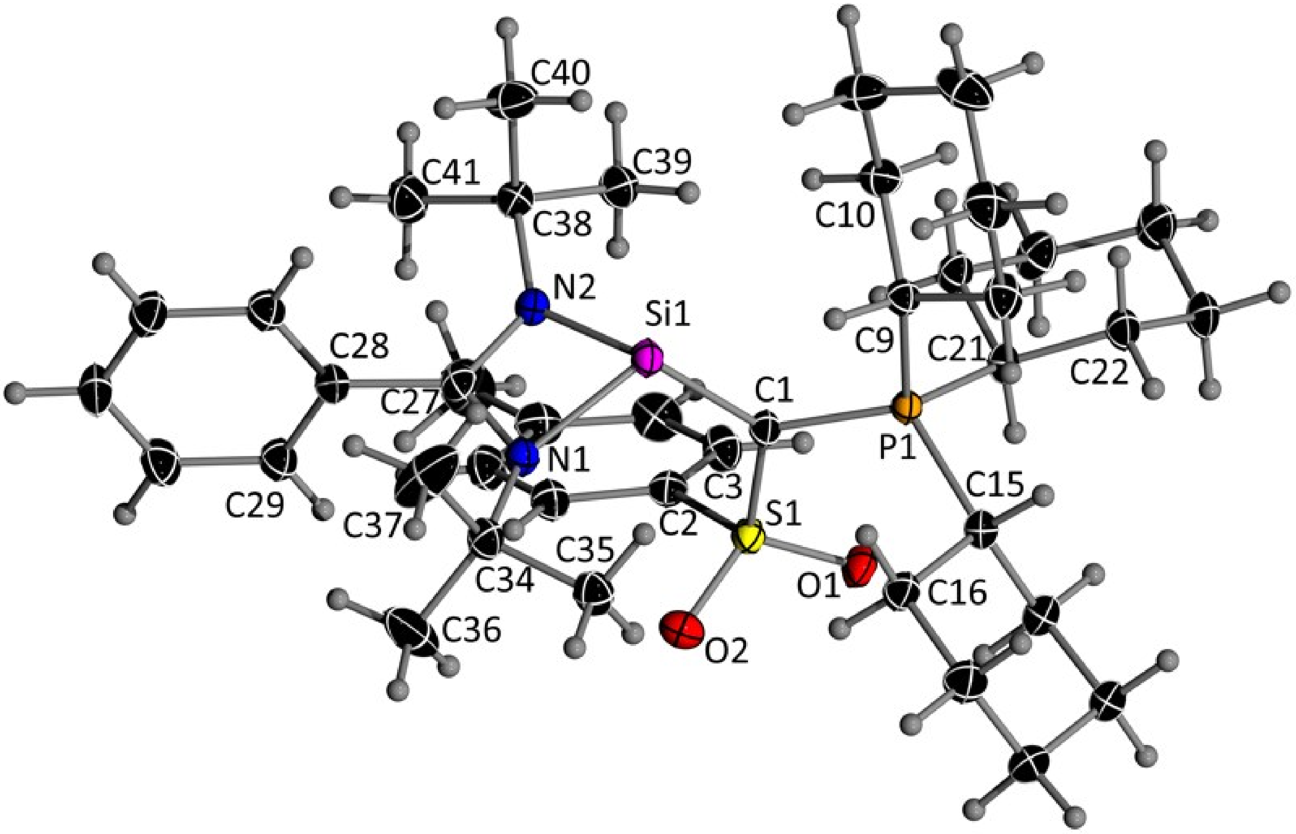

Figure S76. ORTEP of **AYSi-2**. Thermal ellipsoids are drawn at 50% probability level.

Table S6. Atomic coordinates (x 10^4) and equivalentisotropic displacement parameters (Å^{2}x 10^3) for **AYSi-2**. U(eq) is defined as one third of the trace of the orthogonalized U^{ij} tensor for all atoms.

Atom	X	Y	Z	U(eq)
S1	0.31498(3)	0.55339(4)	0.28710(2)	0.01786(10)
P1	0.30444(3)	0.65677(4)	0.17018(2)	0.01592(9)
Si1	0.59010(4)	0.55588(4)	0.22429(2)	0.01825(10)
O1	0.17759(10)	0.60635(12)	0.27829(5)	0.0243(2)
N1	0.63354(12)	0.40748(13)	0.26301(6)	0.0198(3)
O2	0.32215(11)	0.40747(11)	0.28643(5)	0.0236(2)
N2	0.70082(12)	0.61638(13)	0.30717(6)	0.0194(3)
C1	0.40612(14)	0.59790(15)	0.23212(7)	0.0171(3)
C2	0.38619(15)	0.63759(15)	0.36880(7)	0.0195(3)
C3	0.33637(16)	0.76196(17)	0.39999(8)	0.0253(3)
C4	0.38982(18)	0.82190(17)	0.46464(8)	0.0291(4)

C5	0.49293(17)	0.76019(17)	0.49905(8)	0.0279(3)
C6	0.53917(16)	0.63437(17)	0.46745(8)	0.0266(3)
C7	0.48644(15)	0.57283(16)	0.40311(7)	0.0225(3)
C8	0.5546(2)	0.8265(2)	0.56882(9)	0.0400(4)
C9	0.42101(15)	0.68185(15)	0.11225(7)	0.0187(3)
C10	0.51330(16)	0.81259(16)	0.13924(8)	0.0235(3)
C11	0.62119(17)	0.82026(17)	0.09513(9)	0.0307(4)
C12	0.5527(2)	0.81978(18)	0.02596(9)	0.0351(4)
C13	0.46268(18)	0.69032(19)	-0.00187(8)	0.0317(4)
C14	0.35494(16)	0.67435(18)	0.04138(8)	0.0269(3)
C15	0.16774(14)	0.53519(15)	0.11819(7)	0.0194(3)
C16	0.22103(15)	0.38862(15)	0.10269(8)	0.0225(3)
C17	0.11841(17)	0.29014(17)	0.05176(8)	0.0282(3)
C18	-0.02277(16)	0.29294(17)	0.07313(9)	0.0285(4)
C19	-0.07384(15)	0.43944(17)	0.08861(8)	0.0256(3)
C20	0.02754(15)	0.53414(16)	0.14161(8)	0.0235(3)
C21	0.21252(14)	0.81521(15)	0.19757(7)	0.0193(3)
C22	0.13978(15)	0.87405(16)	0.14102(8)	0.0229(3)
C23	0.04609(16)	0.98877(16)	0.16771(8)	0.0270(3)
C24	0.12787(16)	1.10278(17)	0.21959(9)	0.0295(4)
C25	0.20447(16)	1.04665(17)	0.27546(8)	0.0271(3)
C26	0.29673(15)	0.92915(15)	0.24978(7)	0.0216(3)
C27	0.72617(14)	0.48570(15)	0.30986(7)	0.0190(3)
C28	0.83824(15)	0.43403(15)	0.35225(7)	0.0208(3)
C29	0.81028(16)	0.38763(16)	0.40642(8)	0.0244(3)
C30	0.91372(17)	0.33628(18)	0.44488(8)	0.0297(4)
C31	1.04559(17)	0.33010(18)	0.42922(8)	0.0301(4)
C32	1.07449(16)	0.37728(17)	0.37600(8)	0.0271(3)
C33	0.97143(15)	0.42939(16)	0.33754(8)	0.0234(3)
C34	0.63456(16)	0.25856(16)	0.23224(8)	0.0245(3)
C35	0.50998(16)	0.22845(16)	0.17914(8)	0.0261(3)
C36	0.6213(3)	0.1705(2)	0.28050(11)	0.0543(6)
C37	0.76280(19)	0.2266(2)	0.19933(13)	0.0545(6)
C38	0.77461(15)	0.74846(15)	0.34102(8)	0.0220(3)
C39	0.66555(16)	0.85720(16)	0.34882(8)	0.0260(3)
C40	0.85038(19)	0.74694(18)	0.40897(9)	0.0336(4)
C41	0.87477(17)	0.78386(18)	0.29810(9)	0.0318(4)

Table S7. Anisotropic displacement parameters(Å^2) for **AYSi-2**. The anisotropic displacement factor exponent takes the form: -2π^2[$h^2a^{*2}U^{11}$ + ...+ 2 h k a* b* U^{12}].

atom	U^{11}	U^{22}	U^{33}	U^{23}	U^{13}	U^{12}
S1	0.01478(17)	0.02166(19)	0.01836(18)	0.00673(13)	0.00311(13)	0.00166(13)
P1	0.01366(17)	0.01720(18)	0.01745(18)	0.00553(14)	0.00168(13)	0.00285(13)
Si1	0.01534(19)	0.0212(2)	0.0199(2)	0.00786(15)	0.00324(15)	0.00416(15)
O1	0.0137(5)	0.0354(6)	0.0265(6)	0.0120(5)	0.0038(4)	0.0035(4)
N1	0.0173(6)	0.0203(6)	0.0226(6)	0.0070(5)	0.0029(5)	0.0036(5)
O2	0.0270(6)	0.0213(5)	0.0243(5)	0.0071(4)	0.0062(4)	-0.0006(4)
N2	0.0161(6)	0.0205(6)	0.0224(6)	0.0077(5)	0.0012(5)	0.0013(5)
C1	0.0146(6)	0.0197(7)	0.0181(7)	0.0065(6)	0.0024(5)	0.0033(5)
C2	0.0177(7)	0.0242(7)	0.0188(7)	0.0073(6)	0.0062(6)	0.0008(6)
C3	0.0249(8)	0.0277(8)	0.0256(8)	0.0083(6)	0.0079(6)	0.0074(6)
C4	0.0351(9)	0.0274(8)	0.0254(8)	0.0026(7)	0.0118(7)	0.0056(7)
C5	0.0328(9)	0.0306(9)	0.0210(8)	0.0061(6)	0.0059(7)	-0.0019(7)
C6	0.0270(8)	0.0321(9)	0.0223(8)	0.0109(7)	0.0014(6)	0.0023(7)
C7	0.0224(7)	0.0252(8)	0.0220(7)	0.0080(6)	0.0055(6)	0.0036(6)
C8	0.0538(12)	0.0402(10)	0.0232(9)	0.0032(8)	0.0036(8)	-0.0011(9)
C9	0.0179(7)	0.0197(7)	0.0196(7)	0.0061(6)	0.0038(6)	0.0024(6)
C10	0.0239(8)	0.0205(8)	0.0267(8)	0.0051(6)	0.0066(6)	0.0001(6)
C11	0.0292(8)	0.0245(8)	0.0392(9)	0.0031(7)	0.0146(7)	-0.0034(7)
C12	0.0442(10)	0.0302(9)	0.0394(10)	0.0150(8)	0.0235(8)	0.0056(8)
C13	0.0353(9)	0.0395(10)	0.0244(8)	0.0130(7)	0.0089(7)	0.0034(7)
C14	0.0262(8)	0.0356(9)	0.0210(8)	0.0114(7)	0.0030(6)	0.0047(7)
C15	0.0181(7)	0.0206(7)	0.0197(7)	0.0062(6)	0.0008(6)	0.0019(6)
C16	0.0183(7)	0.0213(8)	0.0267(8)	0.0046(6)	0.0015(6)	0.0017(6)
C17	0.0277(8)	0.0223(8)	0.0317(9)	0.0028(7)	0.0004(7)	-0.0005(6)

C18	0.0231(8)	0.0256(8)	0.0352(9)	0.0087(7)	-0.0036(7)	-0.0046(6)
C19	0.0187(7)	0.0285(8)	0.0295(8)	0.0102(7)	-0.0020(6)	-0.0015(6)
C20	0.0171(7)	0.0266(8)	0.0266(8)	0.0070(6)	0.0015(6)	0.0014(6)
C21	0.0155(7)	0.0200(7)	0.0229(7)	0.0059(6)	0.0027(6)	0.0044(5)
C22	0.0202(7)	0.0217(8)	0.0265(8)	0.0071(6)	0.0001(6)	0.0047(6)
C23	0.0202(7)	0.0237(8)	0.0371(9)	0.0097(7)	0.0002(7)	0.0074(6)
C24	0.0230(8)	0.0211(8)	0.0421(10)	0.0040(7)	0.0027(7)	0.0073(6)
C25	0.0210(7)	0.0256(8)	0.0310(8)	-0.0011(7)	0.0040(6)	0.0053(6)
C26	0.0178(7)	0.0211(7)	0.0246(8)	0.0033(6)	0.0017(6)	0.0043(6)
C27	0.0153(7)	0.0227(7)	0.0216(7)	0.0083(6)	0.0061(6)	0.0038(5)
C28	0.0187(7)	0.0203(7)	0.0239(8)	0.0071(6)	0.0015(6)	0.0027(6)
C29	0.0205(7)	0.0279(8)	0.0277(8)	0.0112(7)	0.0054(6)	0.0046(6)
C30	0.0309(9)	0.0365(9)	0.0255(8)	0.0157(7)	0.0031(7)	0.0050(7)
C31	0.0254(8)	0.0364(9)	0.0299(9)	0.0141(7)	-0.0012(7)	0.0097(7)
C32	0.0189(7)	0.0323(9)	0.0311(9)	0.0096(7)	0.0030(6)	0.0049(6)
C33	0.0209(7)	0.0262(8)	0.0250(8)	0.0093(6)	0.0046(6)	0.0030(6)
C34	0.0215(7)	0.0187(7)	0.0321(8)	0.0052(6)	0.0011(6)	0.0041(6)
C35	0.0286(8)	0.0220(8)	0.0261(8)	0.0040(6)	0.0019(6)	0.0036(6)
C36	0.0847(16)	0.0285(10)	0.0433(12)	0.0181(9)	-0.0275(11)	-0.0197(10)
C37	0.0246(9)	0.0383(11)	0.0844(17)	-0.0213(11)	0.0125(10)	0.0056(8)
C38	0.0193(7)	0.0207(7)	0.0257(8)	0.0066(6)	0.0002(6)	-0.0001(6)
C39	0.0239(8)	0.0227(8)	0.0316(8)	0.0067(6)	0.0040(6)	0.0032(6)
C40	0.0367(9)	0.0271(9)	0.0322(9)	0.0073(7)	-0.0110(7)	-0.0026(7)
C41	0.0254(8)	0.0279(8)	0.0429(10)	0.0062(7)	0.0113(7)	-0.0031(7)

6.4 Crystal structure determination of AYSi-3

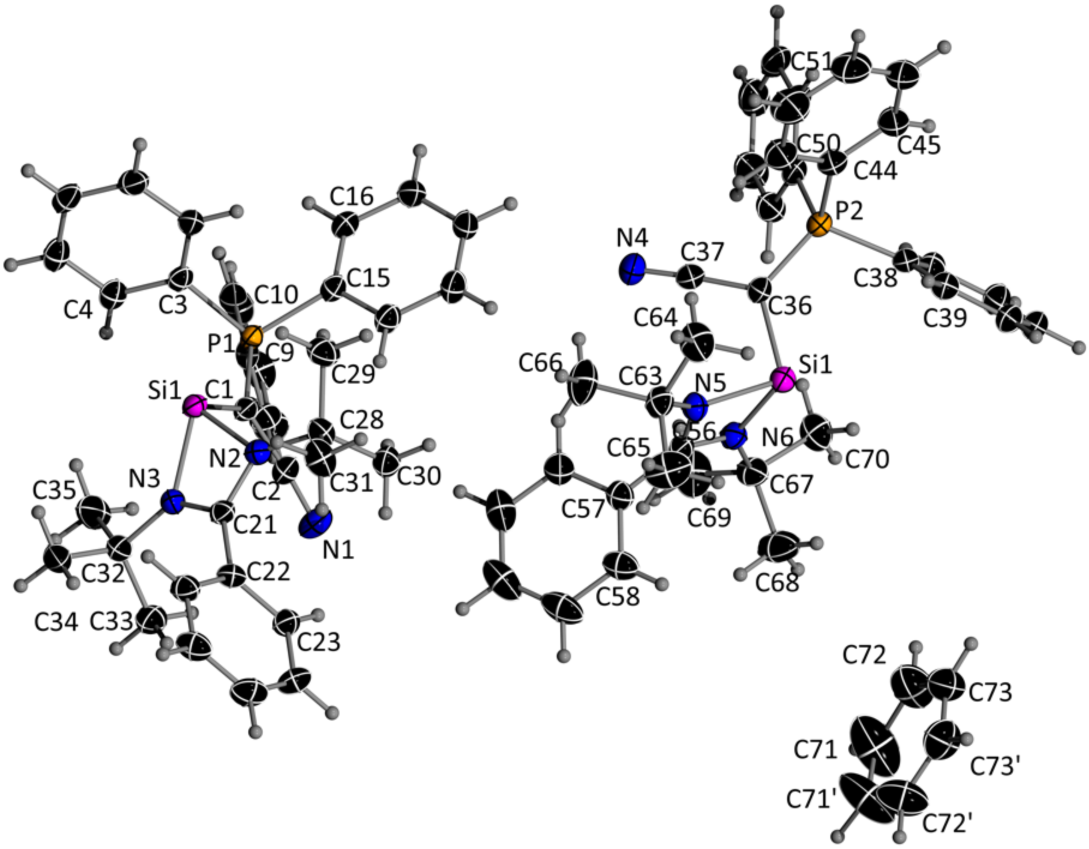

Figure S77. ORTEP of **AYSi-3**. Ellipsoids are drawn at the 50% probability level.

Table S8. Atomic coordinates (x 10^4) and equivalent isotropic displacement parameters ($Å^2$x 10^3) for **AYSi-3**. U(eq) is defined as one third of the trace of the orthogonalized U^{ij} tensor for all atoms.

atom	x	y	z	U(eq)
P1	0.67894(2)	0.20481(6)	0.71480(3)	0.02354(15)
P2	0.55885(2)	0.97076(7)	0.68663(3)	0.02762(16)
Si1	0.71035(2)	0.25260(7)	0.60514(3)	0.02553(16)
Si2	0.56513(2)	0.85289(7)	0.54184(4)	0.02792(17)
N1	0.65733(4)	0.1522(3)	0.53243(12)	0.0381(6)
N2	0.70189(3)	0.3533(2)	0.52812(11)	0.0259(4)
N3	0.71062(3)	0.1548(2)	0.52533(11)	0.0276(5)
N4	0.59624(4)	0.6992(3)	0.69411(14)	0.0404(6)
N5	0.56293(3)	0.6722(2)	0.53249(11)	0.0285(5)
N6	0.59029(3)	0.7802(2)	0.51768(11)	0.0283(5)
C5	0.73421(4)	0.0953(3)	0.80923(14)	0.0335(6)
C6	0.74267(4)	0.2111(3)	0.83364(14)	0.0357(6)
C7	0.73168(4)	0.3254(3)	0.82420(14)	0.0348(6)
C8	0.71184(4)	0.3247(3)	0.79061(13)	0.0305(6)
C9	0.66376(4)	0.0711(3)	0.74447(14)	0.0279(5)
C10	0.66345(4)	0.0493(3)	0.81443(14)	0.0326(6)
C11	0.64983(5)	-0.0396(3)	0.83864(16)	0.0373(6)
C1	0.68420(4)	0.2006(3)	0.63196(12)	0.0254(5)
C2	0.66866(4)	0.1741(3)	0.57955(13)	0.0291(5)
C12	0.63642(5)	-0.1077(3)	0.79338(18)	0.0415(7)
C13	0.63722(5)	-0.0906(3)	0.72450(18)	0.0417(7)
C14	0.65081(5)	-0.0008(3)	0.69934(15)	0.0351(6)
C15	0.66374(4)	0.3441(3)	0.73805(13)	0.0263(5)
C16	0.65928(4)	0.3700(3)	0.80457(14)	0.0332(6)
C17	0.64591(4)	0.4706(3)	0.81807(14)	0.0351(6)
C18	0.63710(4)	0.5462(3)	0.76548(15)	0.0339(6)
C19	0.64163(4)	0.5225(3)	0.69999(15)	0.0357(6)
C20	0.65496(4)	0.4218(3)	0.68578(13)	0.0303(5)
C21	0.70591(4)	0.2577(3)	0.48584(13)	0.0251(5)
C22	0.70679(4)	0.2660(3)	0.41092(13)	0.0262(5)
C23	0.68874(4)	0.2679(3)	0.36766(13)	0.0297(5)
C24	0.68988(5)	0.2785(3)	0.29827(14)	0.0362(6)
C25	0.70902(5)	0.2880(3)	0.27155(14)	0.0400(7)
C26	0.72703(5)	0.2854(3)	0.31425(15)	0.0370(6)
C27	0.72601(4)	0.2746(3)	0.38361(14)	0.0311(6)
C28	0.69460(4)	0.4871(3)	0.51554(14)	0.0300(5)
C29	0.69902(5)	0.5584(3)	0.58309(16)	0.0382(6)
C30	0.67109(4)	0.4858(3)	0.49618(15)	0.0349(6)
C31	0.70540(5)	0.5561(3)	0.45996(17)	0.0413(7)
C32	0.71553(5)	0.0199(3)	0.50945(14)	0.0334(6)
C33	0.70027(5)	-0.0316(3)	0.45255(16)	0.0379(6)
C34	0.73780(5)	0.0067(3)	0.48935(19)	0.0462(8)
C35	0.71333(7)	-0.0578(3)	0.57408(17)	0.0502(9)
C36	0.57090(4)	0.8654(3)	0.63652(14)	0.0281(5)
C37	0.58481(4)	0.7763(3)	0.66962(14)	0.0313(6)

C38	0.57105(4)	1.1264(3)	0.70400(15)	0.0351(6)
C39	0.59214(5)	1.1409(3)	0.69641(18)	0.0441(7)
C40	0.60178(6)	1.2597(4)	0.7105(2)	0.0546(9)
C41	0.59037(6)	1.3640(4)	0.7304(2)	0.0540(9)
C42	0.56940(6)	1.3493(3)	0.73867(19)	0.0499(8)
C43	0.55978(5)	1.2312(3)	0.72572(18)	0.0431(7)
C44	0.55589(4)	0.8976(3)	0.76861(14)	0.0349(6)
C45	0.56002(5)	0.9635(4)	0.82957(15)	0.0437(8)
C46	0.55774(5)	0.9007(5)	0.89152(16)	0.0546(10)
C47	0.55123(7)	0.7735(4)	0.89191(18)	0.0570(10)
C48	0.54709(7)	0.7065(4)	0.83170(17)	0.0559(10)
C49	0.54975(5)	0.7681(3)	0.77047(15)	0.0416(7)
C50	0.53324(4)	1.0113(3)	0.64826(13)	0.0278(5)
C51	0.51561(4)	0.9497(3)	0.66902(14)	0.0317(6)
C52	0.49616(4)	0.9757(3)	0.63610(16)	0.0362(6)
C53	0.49429(4)	1.0618(3)	0.58233(15)	0.0364(6)
C54	0.51178(5)	1.1234(3)	0.56169(14)	0.0348(6)
C55	0.53122(4)	1.0991(3)	0.59466(13)	0.0304(5)
C56	0.58205(4)	0.6629(3)	0.51086(12)	0.0261(5)
C57	0.59204(4)	0.5436(3)	0.48607(14)	0.0300(5)
C58	0.59057(5)	0.5084(3)	0.41821(15)	0.0372(6)
C59	0.60001(5)	0.3949(4)	0.39785(18)	0.0486(8)
C60	0.61124(6)	0.3184(3)	0.4454(2)	0.0524(9)
C61	0.61278(5)	0.3533(3)	0.5129(2)	0.0495(8)
C62	0.60312(5)	0.4640(3)	0.53345(17)	0.0397(7)
C63	0.54556(4)	0.5810(3)	0.52865(15)	0.0351(6)
C64	0.52815(5)	0.6476(3)	0.56340(18)	0.0428(7)
C65	0.53733(6)	0.5663(4)	0.45274(19)	0.0566(10)
C66	0.55120(6)	0.4535(4)	0.5600(2)	0.0583(10)
C67	0.61015(4)	0.8322(3)	0.49670(15)	0.0331(6)
C68	0.61027(6)	0.8304(4)	0.42028(18)	0.0543(9)
C69	0.62855(5)	0.7573(4)	0.5302(2)	0.0533(9)
C70	0.61113(5)	0.9712(3)	0.5229(2)	0.0500(8)
C71	0.49714(9)	0.4605(5)	0.2812(3)	0.0929(19)
C72	0.49521(8)	0.5764(5)	0.3158(2)	0.0685(12)
C73	0.49789(6)	0.6924(4)	0.28246(19)	0.0542(9)
C3	0.70329(4)	0.2084(3)	0.76623(12)	0.0249(5)
C4	0.71456(4)	0.0932(3)	0.77551(13)	0.0292(5)

Table S9. Anisotropic displacement parameters(Å^2) for **AYSi-3**. The anisotropic displacement factor exponent takes the form: $-2\pi^2[\ h^2a^{*2}U^{11} + ...+ 2\ h\ k\ a^*\ b^*\ U^{12}]$.

atom	U^{11}	U^{22}	U^{33}	U^{23}	U^{13}	U^{12}
P1	0.0208(3)	0.0289(3)	0.0204(3)	0.0021(2)	-0.0013(2)	0.0017(2)
P2	0.0242(3)	0.0310(3)	0.0266(3)	-0.0050(3)	-0.0044(2)	0.0039(2)
Si1	0.0221(3)	0.0344(4)	0.0198(3)	-0.0024(3)	-0.0007(2)	0.0019(3)
Si2	0.0255(3)	0.0319(4)	0.0260(3)	-0.0002(3)	-0.0001(3)	0.0030(3)
N1	0.0309(12)	0.0524(16)	0.0299(12)	0.0035(11)	-0.0052(10)	-0.0054(11)
N2	0.0267(10)	0.0280(11)	0.0228(10)	-0.0018(8)	0.0014(8)	-0.0016(8)
N3	0.0304(11)	0.0282(11)	0.0237(10)	-0.0022(8)	-0.0002(8)	0.0081(9)

N4	0.0338(13)	0.0411(14)	0.0445(14)	-0.0040(11)	-0.0088(11)	0.0101(11)
N5	0.0233(10)	0.0336(12)	0.0284(11)	-0.0031(9)	0.0016(8)	-0.0016(9)
N6	0.0234(10)	0.0315(11)	0.0303(11)	-0.0006(9)	0.0037(8)	0.0005(9)
C5	0.0266(13)	0.0467(16)	0.0278(13)	0.0115(12)	0.0050(10)	0.0065(12)
C6	0.0215(12)	0.0619(19)	0.0234(12)	0.0008(12)	0.0010(10)	0.0041(12)
C7	0.0262(13)	0.0499(17)	0.0280(13)	-0.0101(12)	0.0007(10)	-0.0023(12)
C8	0.0269(13)	0.0386(15)	0.0257(12)	-0.0061(11)	-0.0002(10)	0.0060(11)
C9	0.0225(12)	0.0282(13)	0.0329(13)	0.0049(10)	0.0019(10)	0.0058(10)
C10	0.0302(13)	0.0358(14)	0.0323(14)	0.0040(11)	0.0048(11)	0.0057(11)
C11	0.0395(15)	0.0372(15)	0.0370(15)	0.0085(12)	0.0141(12)	0.0082(12)
C1	0.0226(11)	0.0313(13)	0.0217(11)	0.0004(10)	-0.0025(9)	0.0002(10)
C2	0.0244(12)	0.0362(14)	0.0263(12)	0.0040(11)	-0.0007(10)	-0.0005(10)
C12	0.0385(16)	0.0334(15)	0.0546(19)	0.0053(14)	0.0165(14)	-0.0007(12)
C13	0.0374(15)	0.0371(16)	0.0508(18)	-0.0017(14)	0.0036(13)	-0.0073(13)
C14	0.0362(15)	0.0349(15)	0.0340(14)	0.0017(12)	0.0011(11)	-0.0012(12)
C15	0.0209(11)	0.0318(13)	0.0257(12)	0.0021(10)	-0.0006(9)	-0.0002(10)
C16	0.0329(14)	0.0396(15)	0.0259(13)	-0.0003(11)	-0.0042(10)	0.0071(12)
C17	0.0303(13)	0.0448(16)	0.0298(13)	-0.0070(12)	0.0001(11)	0.0055(12)
C18	0.0254(13)	0.0341(14)	0.0412(15)	-0.0066(12)	-0.0032(11)	0.0054(11)
C19	0.0329(14)	0.0362(15)	0.0370(15)	0.0059(12)	-0.0028(11)	0.0073(12)
C20	0.0287(13)	0.0364(14)	0.0256(12)	0.0048(11)	-0.0001(10)	0.0030(11)
C21	0.0206(11)	0.0317(13)	0.0226(12)	-0.0029(10)	-0.0002(9)	-0.0006(9)
C22	0.0302(13)	0.0270(12)	0.0215(12)	-0.0030(9)	0.0031(10)	-0.0012(10)
C23	0.0263(12)	0.0367(14)	0.0259(12)	-0.0014(11)	0.0018(10)	0.0007(11)
C24	0.0404(15)	0.0438(16)	0.0235(13)	0.0002(11)	-0.0037(11)	-0.0029(13)
C25	0.0528(18)	0.0455(17)	0.0226(13)	0.0013(12)	0.0079(12)	-0.0015(14)
C26	0.0381(15)	0.0414(16)	0.0334(14)	-0.0048(12)	0.0143(12)	-0.0057(12)
C27	0.0282(13)	0.0347(14)	0.0306(13)	-0.0052(11)	0.0030(10)	-0.0027(11)
C28	0.0315(13)	0.0287(13)	0.0304(13)	-0.0009(11)	0.0062(10)	0.0020(10)
C29	0.0430(16)	0.0313(14)	0.0402(16)	-0.0089(12)	0.0027(13)	-0.0028(12)
C30	0.0338(14)	0.0384(15)	0.0323(14)	0.0031(12)	0.0009(11)	0.0079(12)
C31	0.0436(17)	0.0337(15)	0.0487(18)	0.0061(13)	0.0155(14)	0.0028(13)
C32	0.0392(15)	0.0291(14)	0.0305(13)	-0.0056(11)	-0.0059(11)	0.0080(11)
C33	0.0435(16)	0.0291(14)	0.0395(15)	-0.0026(12)	-0.0065(13)	-0.0004(12)
C34	0.0356(16)	0.0453(18)	0.0557(19)	-0.0216(15)	-0.0078(14)	0.0112(13)
C35	0.077(2)	0.0347(16)	0.0378(16)	0.0028(13)	-0.0012(16)	0.0156(16)
C36	0.0210(11)	0.0313(13)	0.0311(13)	-0.0042(10)	-0.0028(10)	0.0026(10)
C37	0.0294(13)	0.0333(14)	0.0304(13)	-0.0067(11)	-0.0033(11)	0.0011(11)
C38	0.0315(14)	0.0358(15)	0.0365(15)	-0.0083(12)	-0.0067(11)	-0.0004(11)
C39	0.0331(15)	0.0439(18)	0.0544(19)	-0.0126(15)	-0.0011(13)	-0.0043(13)
C40	0.0400(18)	0.054(2)	0.069(2)	-0.0144(18)	0.0003(16)	-0.0146(16)
C41	0.058(2)	0.0429(19)	0.058(2)	-0.0134(16)	-0.0102(17)	-0.0102(16)
C42	0.054(2)	0.0405(18)	0.052(2)	-0.0148(15)	-0.0123(16)	0.0037(15)
C43	0.0360(16)	0.0408(17)	0.0508(18)	-0.0150(14)	-0.0065(13)	0.0028(13)
C44	0.0306(13)	0.0477(17)	0.0251(13)	-0.0032(12)	-0.0056(10)	0.0129(12)
C45	0.0324(15)	0.064(2)	0.0329(15)	-0.0107(14)	-0.0075(12)	0.0109(14)
C46	0.0445(18)	0.093(3)	0.0250(14)	-0.0070(17)	-0.0055(13)	0.0276(19)
C47	0.073(3)	0.065(2)	0.0333(17)	0.0112(16)	0.0038(16)	0.033(2)
C48	0.081(3)	0.053(2)	0.0346(17)	0.0099(15)	0.0078(17)	0.0255(19)

C49	0.058(2)	0.0394(16)	0.0268(14)	0.0034(12)	0.0016(13)	0.0180(14)
C50	0.0266(12)	0.0296(13)	0.0262(12)	-0.0065(10)	-0.0034(10)	0.0035(10)
C51	0.0299(13)	0.0334(14)	0.0316(13)	0.0019(11)	0.0008(11)	0.0036(11)
C52	0.0273(13)	0.0394(16)	0.0413(16)	-0.0038(12)	-0.0011(11)	0.0010(11)
C53	0.0298(14)	0.0422(16)	0.0356(14)	-0.0065(12)	-0.0068(11)	0.0082(12)
C54	0.0387(15)	0.0370(15)	0.0279(13)	0.0004(11)	-0.0029(11)	0.0099(12)
C55	0.0305(13)	0.0316(13)	0.0287(13)	-0.0019(11)	0.0013(10)	0.0025(11)
C56	0.0225(12)	0.0336(13)	0.0219(11)	-0.0009(10)	-0.0001(9)	0.0015(10)
C57	0.0254(12)	0.0327(14)	0.0323(13)	-0.0022(11)	0.0051(10)	-0.0012(10)
C58	0.0365(15)	0.0417(16)	0.0349(14)	-0.0064(12)	0.0120(12)	-0.0079(12)
C59	0.0500(19)	0.0508(19)	0.0485(19)	-0.0156(16)	0.0266(15)	-0.0094(15)
C60	0.053(2)	0.0354(17)	0.073(2)	-0.0088(16)	0.0304(18)	-0.0003(15)
C61	0.0397(17)	0.0398(17)	0.070(2)	0.0037(16)	0.0099(16)	0.0081(14)
C62	0.0361(15)	0.0386(16)	0.0438(17)	-0.0024(13)	-0.0003(13)	0.0042(12)
C63	0.0258(13)	0.0405(16)	0.0394(15)	-0.0091(12)	0.0051(11)	-0.0077(11)
C64	0.0325(15)	0.0484(18)	0.0492(18)	0.0005(14)	0.0134(13)	-0.0024(13)
C65	0.0424(18)	0.077(3)	0.050(2)	-0.0193(19)	0.0008(15)	-0.0142(18)
C66	0.047(2)	0.047(2)	0.082(3)	0.0129(19)	0.0095(19)	-0.0064(16)
C67	0.0234(12)	0.0374(15)	0.0386(15)	-0.0001(12)	0.0026(11)	-0.0022(11)
C68	0.054(2)	0.068(2)	0.0423(18)	0.0057(17)	0.0133(15)	-0.0243(18)
C69	0.0265(15)	0.055(2)	0.078(3)	0.0095(18)	-0.0009(15)	-0.0011(14)
C70	0.0349(16)	0.0418(18)	0.074(2)	-0.0046(17)	0.0099(16)	-0.0088(13)
C71	0.109(4)	0.060(3)	0.119(5)	0.025(3)	0.070(4)	0.008(3)
C72	0.089(3)	0.068(3)	0.052(2)	0.011(2)	0.026(2)	0.015(2)
C73	0.058(2)	0.054(2)	0.0505(19)	-0.0125(17)	0.0035(17)	0.0001(17)
C3	0.0230(11)	0.0343(13)	0.0174(11)	0.0029(10)	0.0013(9)	0.0027(10)
C4	0.0269(12)	0.0353(14)	0.0257(12)	0.0071(11)	0.0038(10)	0.0014(11)

6.5 Crystal structure determination of CS_2 adduct 4

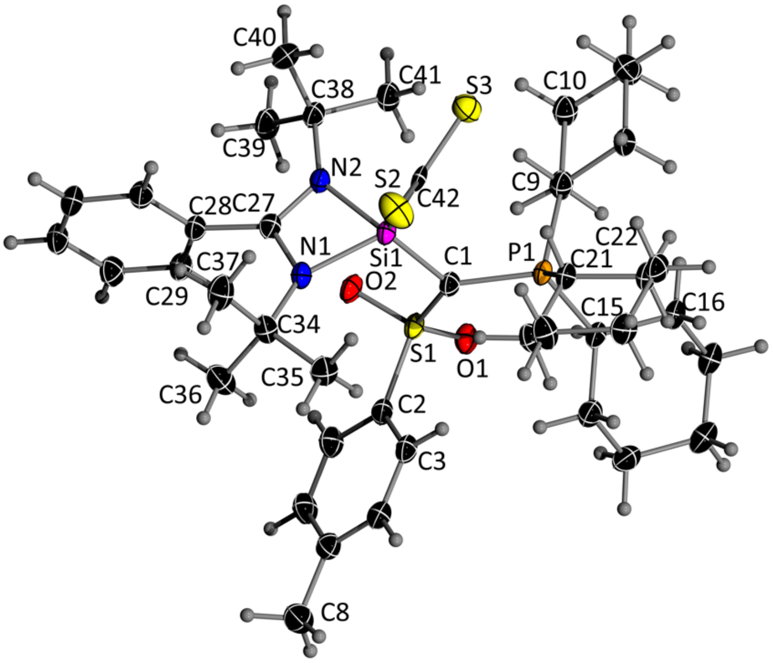

Figure S78. ORTEP of $\mathbf{5_{Ts}}$. Thermal ellipsoids are drawn at the 50% probability level.

Table S10. Atomic coordinates (x 10^4) and equivalentisotropic displacement parameters (Å^{2}x 10^3) for **5$_{Ts}$**. U(eq) is defined as one third of the trace of the orthogonalized U^{ij} tensor for all atoms.

atom	X	Y	Z	U(eq)
Si1	0.67462(3)	0.51472(3)	0.70011(2)	0.01928(12)
O1	0.92993(10)	0.45400(9)	0.84091(6)	0.0257(3)
O2	0.89580(9)	0.46524(9)	0.72176(6)	0.0250(3)
S1	0.86063(3)	0.43852(3)	0.78049(2)	0.02057(12)
S2	0.55229(4)	0.68093(3)	0.71635(2)	0.03213(13)
S3	0.44907(4)	0.51151(4)	0.66494(3)	0.03990(15)
P1	0.71793(3)	0.53335(3)	0.85414(2)	0.01901(12)
N1	0.67914(11)	0.42910(10)	0.63835(7)	0.0213(3)
N2	0.74208(11)	0.56280(10)	0.63991(7)	0.0210(3)
C1	0.74667(13)	0.48715(12)	0.78117(8)	0.0202(4)
C2	0.84780(14)	0.31849(12)	0.77359(8)	0.0229(4)
C12	0.82865(15)	0.83327(13)	0.89386(9)	0.0292(4)
C13	0.71676(15)	0.81867(13)	0.87200(10)	0.0284(4)
C14	0.68706(14)	0.72207(13)	0.88574(9)	0.0241(4)
C15	0.58327(14)	0.51771(12)	0.85294(8)	0.0218(4)
C16	0.54720(14)	0.54371(13)	0.91643(8)	0.0242(4)
C17	0.43317(14)	0.54094(13)	0.90489(9)	0.0265(4)
C18	0.39473(15)	0.44719(14)	0.88516(10)	0.0293(4)
C19	0.43245(15)	0.41730(15)	0.82407(10)	0.0316(4)
C20	0.54621(14)	0.42217(13)	0.83263(9)	0.0264(4)
C21	0.79406(14)	0.48906(12)	0.93014(8)	0.0221(4)
C22	0.78515(14)	0.54432(13)	0.99170(8)	0.0250(4)
C23	0.86779(15)	0.51592(14)	1.04803(9)	0.0280(4)
C24	0.86269(16)	0.41557(15)	1.06210(9)	0.0316(4)
C25	0.86449(16)	0.35958(14)	1.00103(9)	0.0305(4)
C26	0.78265(15)	0.38832(13)	0.94391(9)	0.0269(4)
C27	0.73217(13)	0.48545(12)	0.60717(8)	0.0208(4)
C28	0.77925(14)	0.46088(12)	0.55068(8)	0.0227(4)
C29	0.87431(14)	0.42231(13)	0.56433(9)	0.0251(4)
C30	0.92069(15)	0.39634(14)	0.51340(9)	0.0288(4)
C31	0.87229(16)	0.40766(14)	0.44950(9)	0.0306(4)
C32	0.77859(16)	0.44561(14)	0.43624(9)	0.0305(4)
C33	0.73173(15)	0.47351(13)	0.48675(9)	0.0265(4)
C34	0.63124(14)	0.34185(13)	0.61403(9)	0.0251(4)
C35	0.55357(16)	0.35697(16)	0.55186(10)	0.0345(5)
C36	0.57869(15)	0.30520(14)	0.66690(9)	0.0288(4)
C37	0.70966(16)	0.27339(14)	0.60155(11)	0.0343(5)
C38	0.78994(15)	0.64877(13)	0.62532(8)	0.0245(4)
C39	0.89028(16)	0.63412(14)	0.60405(10)	0.0308(4)
C40	0.71689(17)	0.69886(14)	0.57275(9)	0.0328(4)
C41	0.80826(15)	0.70336(14)	0.68838(9)	0.0284(4)
C42	0.54936(13)	0.57457(12)	0.69350(7)	0.0192(4)
C3	0.77012(14)	0.27045(13)	0.79147(8)	0.0245(4)
C4	0.76599(15)	0.17724(13)	0.78482(9)	0.0268(4)
C5	0.84054(15)	0.13038(13)	0.76138(9)	0.0276(4)
C6	0.91918(15)	0.17983(14)	0.74422(9)	0.0294(4)

C7	0.92321(14)	0.27250(14)	0.74987(9)	0.0272(4)
C8	0.83761(18)	0.02910(14)	0.75406(11)	0.0365(5)
C9	0.74722(14)	0.65481(12)	0.85253(8)	0.0220(4)
C10	0.86058(14)	0.67008(13)	0.87081(9)	0.0245(4)
C11	0.88732(15)	0.76798(13)	0.85905(9)	0.0280(4)

Table S11. Anisotropic displacement parameters($Å^2$) for **5**$_{\mathbf{Ts}}$. The anisotropic displacement factor exponent takes the form: $-2\pi^2[\,h^2a^{*2}U^{11} + ...+ 2\,h\,k\,a^*\,b^*\,U^{12}\,]$.

atom	U^{11}	U^{22}	U^{33}	U^{23}	U^{13}	U^{12}
Si1	0.0198(2)	0.0238(3)	0.0153(2)	-0.00060(17)	0.00619(18)	0.00082(18)
O1	0.0211(7)	0.0344(7)	0.0220(6)	-0.0012(5)	0.0047(5)	0.0028(5)
O2	0.0235(7)	0.0342(7)	0.0199(6)	0.0037(5)	0.0106(5)	0.0033(5)
S1	0.0192(2)	0.0272(2)	0.0166(2)	0.00052(16)	0.00675(16)	0.00174(17)
S2	0.0329(3)	0.0300(3)	0.0341(3)	-0.00232(19)	0.0080(2)	0.0038(2)
S3	0.0267(3)	0.0413(3)	0.0505(3)	-0.0120(2)	0.0042(2)	0.0016(2)
P1	0.0200(2)	0.0234(2)	0.0150(2)	-0.00017(16)	0.00694(17)	0.00018(17)
N1	0.0216(8)	0.0250(8)	0.0184(7)	-0.0016(6)	0.0069(6)	0.0003(6)
N2	0.0241(8)	0.0240(8)	0.0159(7)	0.0002(6)	0.0063(6)	0.0007(6)
C1	0.0209(9)	0.0242(9)	0.0167(8)	0.0005(7)	0.0067(7)	0.0027(7)
C2	0.0241(9)	0.0279(10)	0.0171(8)	-0.0002(7)	0.0052(7)	0.0034(7)
C12	0.0333(11)	0.0281(10)	0.0276(9)	-0.0029(8)	0.0089(8)	-0.0040(8)
C13	0.0316(11)	0.0260(10)	0.0285(9)	-0.0013(7)	0.0079(8)	0.0017(8)
C14	0.0242(9)	0.0280(10)	0.0210(8)	-0.0019(7)	0.0068(7)	0.0004(7)
C15	0.0218(9)	0.0269(9)	0.0179(8)	-0.0005(7)	0.0067(7)	-0.0002(7)
C16	0.0255(10)	0.0301(10)	0.0191(8)	-0.0012(7)	0.0095(7)	-0.0011(8)
C17	0.0264(10)	0.0328(10)	0.0235(9)	0.0008(8)	0.0129(8)	0.0021(8)
C18	0.0232(10)	0.0363(11)	0.0310(10)	0.0002(8)	0.0117(8)	-0.0028(8)
C19	0.0257(10)	0.0376(11)	0.0337(10)	-0.0096(9)	0.0110(8)	-0.0057(8)
C20	0.0245(10)	0.0285(10)	0.0281(9)	-0.0050(7)	0.0100(8)	-0.0010(8)
C21	0.0231(9)	0.0286(10)	0.0155(8)	0.0013(7)	0.0062(7)	0.0013(7)
C22	0.0270(10)	0.0321(10)	0.0174(8)	-0.0006(7)	0.0074(7)	-0.0006(8)
C23	0.0258(10)	0.0404(11)	0.0181(8)	0.0009(8)	0.0050(7)	-0.0030(8)
C24	0.0295(10)	0.0430(12)	0.0222(9)	0.0080(8)	0.0045(8)	-0.0012(9)
C25	0.0324(11)	0.0318(11)	0.0285(10)	0.0087(8)	0.0086(8)	0.0027(8)
C26	0.0302(10)	0.0298(10)	0.0217(9)	0.0019(7)	0.0074(8)	-0.0012(8)
C27	0.0189(9)	0.0265(9)	0.0172(8)	0.0010(7)	0.0035(7)	0.0034(7)
C28	0.0272(10)	0.0236(9)	0.0190(8)	-0.0010(7)	0.0092(7)	-0.0010(7)
C29	0.0245(9)	0.0306(10)	0.0216(9)	-0.0002(7)	0.0080(7)	0.0003(8)
C30	0.0272(10)	0.0332(10)	0.0284(10)	-0.0014(8)	0.0113(8)	0.0026(8)
C31	0.0365(11)	0.0345(11)	0.0246(9)	-0.0040(8)	0.0157(8)	0.0006(9)
C32	0.0385(11)	0.0361(11)	0.0181(9)	-0.0004(8)	0.0081(8)	0.0001(9)
C33	0.0285(10)	0.0303(10)	0.0214(9)	-0.0007(7)	0.0066(8)	0.0020(8)
C34	0.0244(9)	0.0274(9)	0.0247(9)	-0.0060(7)	0.0075(7)	-0.0035(8)
C35	0.0323(11)	0.0455(13)	0.0253(10)	-0.0048(9)	0.0041(8)	-0.0073(9)
C36	0.0261(10)	0.0314(10)	0.0299(10)	-0.0019(8)	0.0079(8)	-0.0050(8)
C37	0.0352(11)	0.0275(10)	0.0442(12)	-0.0084(9)	0.0175(9)	-0.0036(9)
C38	0.0302(10)	0.0256(9)	0.0195(8)	0.0002(7)	0.0097(7)	-0.0034(8)
C39	0.0337(11)	0.0342(11)	0.0285(10)	-0.0029(8)	0.0159(8)	-0.0069(9)
C40	0.0445(12)	0.0289(10)	0.0255(9)	0.0061(8)	0.0077(9)	0.0011(9)

C41	0.0334(11)	0.0299(10)	0.0238(9)	-0.0037(8)	0.0106(8)	-0.0076(8)
C42	0.0189(8)	0.0311(10)	0.0090(7)	0.0025(6)	0.0060(6)	0.0020(7)
C3	0.0239(9)	0.0325(10)	0.0185(8)	0.0006(7)	0.0073(7)	0.0045(8)
C4	0.0291(10)	0.0318(10)	0.0204(8)	0.0024(7)	0.0067(7)	0.0000(8)
C5	0.0317(10)	0.0295(10)	0.0207(8)	0.0003(7)	0.0024(8)	0.0050(8)
C6	0.0254(10)	0.0354(11)	0.0283(10)	-0.0045(8)	0.0073(8)	0.0078(8)
C7	0.0225(9)	0.0348(11)	0.0258(9)	-0.0021(8)	0.0083(7)	0.0028(8)
C8	0.0405(12)	0.0327(11)	0.0362(11)	-0.0032(9)	0.0068(9)	0.0034(9)
C9	0.0234(9)	0.0251(9)	0.0190(8)	-0.0003(7)	0.0076(7)	-0.0016(7)
C10	0.0228(9)	0.0289(10)	0.0231(9)	-0.0029(7)	0.0080(7)	-0.0005(7)
C11	0.0281(10)	0.0315(10)	0.0261(9)	-0.0034(8)	0.0096(8)	-0.0063(8)

6.6 Crystal structure determination of CS_2 adduct 5_{CN}

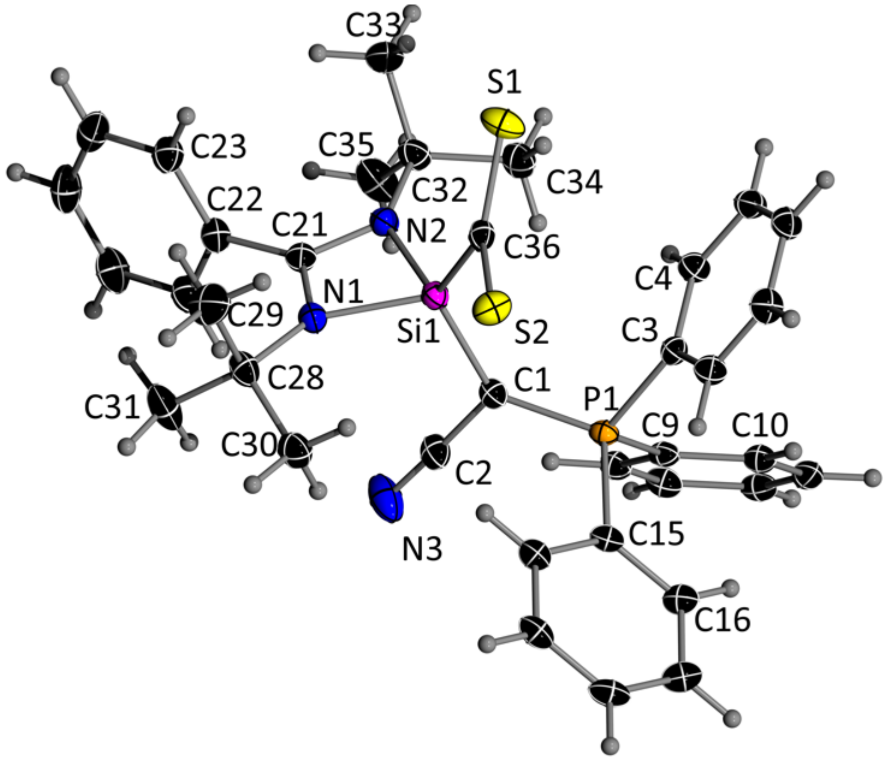

Figure S79. ORTEP of **5_{CN}**. Ellipsoids are drawn at the 50% probability level.

Table S12 Atomic coordinates (x 10^4) and equivalentisotropic displacement parameters (Å^{2}x 10^3) for **5_{CN}**. U(eq) is defined as one third of the trace of the orthogonalized U^{ij} tensor for all atoms.

atom	X	Y	Z	U(eq)
Si1	0.70009(3)	0.38525(2)	0.77590(2)	0.01735(9)
N1	0.60753(10)	0.60876(9)	0.67061(10)	0.0381(3)
C1	0.74601(10)	0.49135(9)	0.74974(8)	0.0188(3)
S2	0.80233(3)	0.21770(3)	0.88505(2)	0.03057(10)
S1	0.79374(3)	0.24593(2)	0.71029(2)	0.02613(9)
P1	0.86326(2)	0.52021(2)	0.75988(2)	0.01607(9)
N2	0.56839(8)	0.37701(8)	0.71081(7)	0.0199(2)
C2	0.67092(10)	0.55675(9)	0.70627(9)	0.0236(3)

N3	0.63608(8)	0.40536(8)	0.84407(7)	0.0199(2)
C3	0.95449(9)	0.43715(9)	0.82229(8)	0.0175(2)
C4	0.97458(10)	0.42777(9)	0.90751(8)	0.0207(3)
C5	1.04629(10)	0.36598(10)	0.95715(9)	0.0227(3)
C6	1.09863(10)	0.31341(9)	0.92248(9)	0.0232(3)
C7	1.07884(10)	0.32246(10)	0.83775(9)	0.0242(3)
C8	1.00713(10)	0.38460(9)	0.78750(9)	0.0211(3)
C9	0.90616(10)	0.63038(9)	0.81345(8)	0.0179(3)
C10	1.00791(10)	0.65001(10)	0.84899(8)	0.0206(3)
C11	1.04246(10)	0.73363(10)	0.89030(8)	0.0224(3)
C12	0.97603(11)	0.79831(10)	0.89609(8)	0.0227(3)
C13	0.87520(11)	0.77938(10)	0.86122(9)	0.0225(3)
C14	0.83978(10)	0.69525(9)	0.82022(8)	0.0203(3)
C15	0.87076(10)	0.52787(9)	0.65891(8)	0.0190(3)
C16	0.94367(11)	0.57952(10)	0.64709(9)	0.0228(3)
C17	0.94906(12)	0.57854(10)	0.56918(9)	0.0261(3)
C18	0.88160(12)	0.52599(10)	0.50328(9)	0.0248(3)
C19	0.80935(11)	0.47457(10)	0.51494(9)	0.0242(3)
C20	0.80373(10)	0.47499(10)	0.59272(9)	0.0220(3)
C21	0.54855(10)	0.39625(9)	0.77774(9)	0.0198(3)
C22	0.45044(10)	0.41052(10)	0.78010(9)	0.0218(3)
C23	0.40408(11)	0.49677(10)	0.75657(10)	0.0267(3)
C24	0.31637(11)	0.51366(11)	0.76516(10)	0.0317(3)
C25	0.27599(11)	0.44530(12)	0.79738(11)	0.0333(4)
C26	0.32137(11)	0.35907(12)	0.81941(10)	0.0309(3)
C27	0.40879(10)	0.34104(10)	0.81060(9)	0.0251(3)
C28	0.50239(10)	0.34809(10)	0.62315(9)	0.0244(3)
C29	0.48379(14)	0.24393(12)	0.62389(11)	0.0389(4)
C30	0.55781(11)	0.36863(11)	0.56802(9)	0.0282(3)
C31	0.40509(12)	0.40200(14)	0.58760(11)	0.0395(4)
C32	0.66035(10)	0.43014(10)	0.93368(9)	0.0235(3)
C33	0.60267(13)	0.51547(13)	0.94012(10)	0.0364(4)
C34	0.64089(12)	0.34733(13)	0.97984(10)	0.0346(4)
C35	0.77044(11)	0.45271(11)	0.97241(9)	0.0268(3)
C36	0.77260(9)	0.27496(9)	0.79477(9)	0.0192(3)

Table S13 Anisotropic displacement parameters(Å^2) for **5$_{CN}$**. The anisotropic displacement factor exponent takes the form: $-2\pi^2[h^2a^{*2}U^{11} + ... + 2hka^*b^*U^{12}]$.

atom	U^{11}	U^{22}	U^{33}	U^{23}	U^{13}	U^{12}
Si1	0.01681(17)	0.01659(17)	0.01728(17)	0.00076(12)	0.00565(14)	0.00077(12)
N1	0.0286(7)	0.0219(6)	0.0464(8)	-0.0010(6)	-0.0021(6)	0.0029(5)
C1	0.0187(6)	0.0179(6)	0.0181(6)	0.0002(5)	0.0058(5)	0.0009(5)
S2	0.0406(2)	0.02389(18)	0.02373(19)	0.00525(13)	0.00973(16)	0.00815(15)
S1	0.03141(19)	0.02181(18)	0.03011(19)	0.00003(13)	0.01765(16)	0.00120(13)
P1	0.01843(16)	0.01538(16)	0.01384(16)	-0.00019(11)	0.00609(13)	0.00013(11)
N2	0.0171(5)	0.0200(5)	0.0201(6)	-0.0004(4)	0.0052(4)	0.0000(4)
C2	0.0218(7)	0.0177(6)	0.0266(7)	-0.0019(5)	0.0050(6)	-0.0027(5)
N3	0.0195(5)	0.0216(5)	0.0184(5)	0.0014(4)	0.0073(4)	0.0012(4)
C3	0.0174(6)	0.0157(6)	0.0184(6)	-0.0011(5)	0.0065(5)	-0.0022(5)

C4	0.0220(6)	0.0218(6)	0.0189(6)	-0.0009(5)	0.0092(5)	0.0008(5)
C5	0.0249(7)	0.0231(7)	0.0187(6)	0.0025(5)	0.0074(5)	-0.0006(5)
C6	0.0209(6)	0.0187(6)	0.0259(7)	0.0027(5)	0.0056(5)	0.0010(5)
C7	0.0240(7)	0.0226(7)	0.0274(7)	-0.0018(5)	0.0120(6)	0.0032(5)
C8	0.0234(6)	0.0219(6)	0.0187(6)	-0.0009(5)	0.0094(5)	0.0004(5)
C9	0.0225(6)	0.0172(6)	0.0130(6)	-0.0001(5)	0.0063(5)	-0.0023(5)
C10	0.0225(6)	0.0221(6)	0.0174(6)	0.0004(5)	0.0082(5)	0.0001(5)
C11	0.0237(7)	0.0260(7)	0.0166(6)	-0.0004(5)	0.0074(5)	-0.0053(5)
C12	0.0325(7)	0.0192(6)	0.0160(6)	-0.0023(5)	0.0096(6)	-0.0060(5)
C13	0.0287(7)	0.0195(6)	0.0184(6)	-0.0009(5)	0.0089(6)	0.0019(5)
C14	0.0226(6)	0.0199(6)	0.0168(6)	-0.0002(5)	0.0065(5)	-0.0002(5)
C15	0.0236(6)	0.0174(6)	0.0158(6)	0.0019(5)	0.0080(5)	0.0036(5)
C16	0.0289(7)	0.0194(6)	0.0204(7)	-0.0004(5)	0.0104(6)	-0.0007(5)
C17	0.0348(8)	0.0221(7)	0.0263(7)	0.0027(6)	0.0175(6)	0.0011(6)
C18	0.0352(8)	0.0236(7)	0.0179(6)	0.0035(5)	0.0132(6)	0.0083(6)
C19	0.0260(7)	0.0257(7)	0.0168(6)	-0.0013(5)	0.0048(5)	0.0057(5)
C20	0.0223(6)	0.0229(7)	0.0186(6)	0.0002(5)	0.0064(5)	0.0018(5)
C21	0.0200(6)	0.0147(6)	0.0236(7)	0.0024(5)	0.0080(5)	0.0011(5)
C22	0.0186(6)	0.0214(6)	0.0236(7)	-0.0023(5)	0.0068(5)	-0.0003(5)
C23	0.0239(7)	0.0230(7)	0.0307(8)	0.0000(6)	0.0087(6)	0.0021(6)
C24	0.0246(7)	0.0284(8)	0.0370(9)	-0.0061(6)	0.0076(7)	0.0060(6)
C25	0.0206(7)	0.0425(9)	0.0372(9)	-0.0112(7)	0.0124(6)	-0.0008(6)
C26	0.0246(7)	0.0356(8)	0.0340(8)	-0.0060(6)	0.0134(6)	-0.0085(6)
C27	0.0229(7)	0.0233(7)	0.0275(7)	-0.0025(6)	0.0088(6)	-0.0029(5)
C28	0.0213(6)	0.0249(7)	0.0210(7)	-0.0035(5)	0.0028(5)	-0.0017(5)
C29	0.0469(10)	0.0296(8)	0.0353(9)	-0.0075(7)	0.0121(8)	-0.0154(7)
C30	0.0279(7)	0.0312(8)	0.0212(7)	-0.0031(6)	0.0059(6)	-0.0023(6)
C31	0.0246(8)	0.0576(11)	0.0266(8)	-0.0042(7)	0.0007(6)	0.0096(7)
C32	0.0237(7)	0.0286(7)	0.0180(6)	0.0004(5)	0.0085(5)	0.0020(6)
C33	0.0366(9)	0.0435(10)	0.0268(8)	-0.0067(7)	0.0106(7)	0.0128(7)
C34	0.0327(8)	0.0453(9)	0.0250(8)	0.0082(7)	0.0110(6)	-0.0050(7)
C35	0.0262(7)	0.0313(8)	0.0211(7)	-0.0036(6)	0.0079(6)	-0.0016(6)
C36	0.0158(6)	0.0164(6)	0.0230(7)	-0.0013(5)	0.0057(5)	-0.0024(5)

6.7 Crystal structure determination of Silanone 6

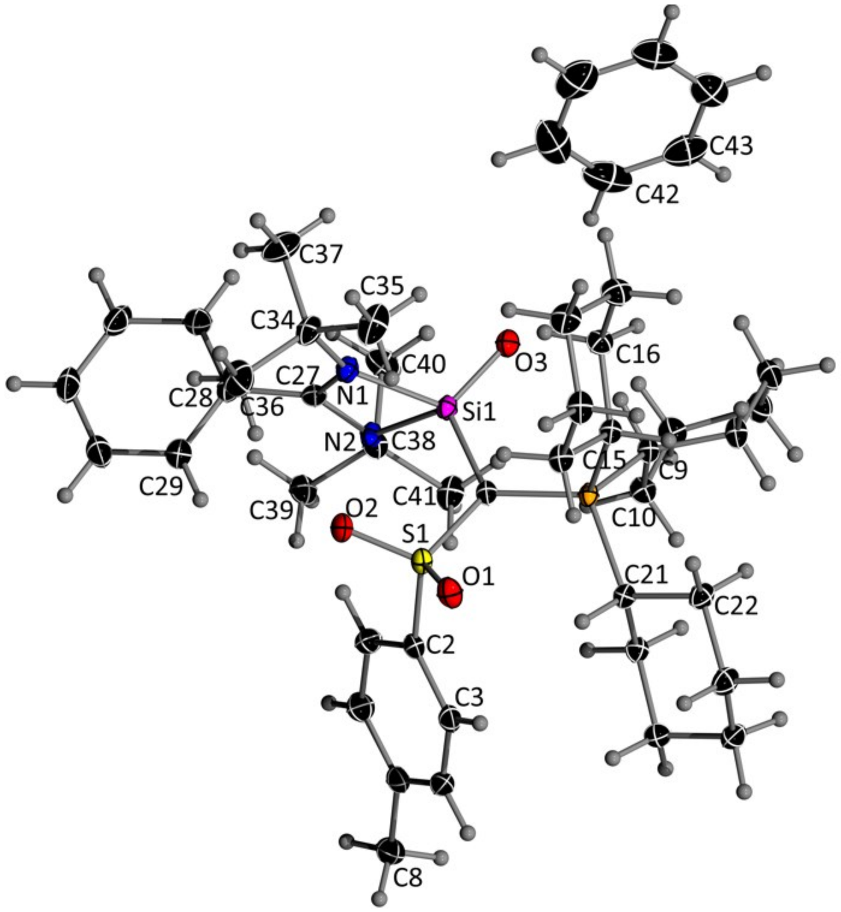

Figure S80. ORTEP of **6**. Ellipsoids are drawn at the 50% probability level.

Table S14. Atomic coordinates (x 10^4) and equivalentisotropic displacement parameters (Å^{2}x 10^3) for **6**. U(eq) is defined as one third of the trace of the orthogonalized U^{ij} tensor for all atoms.

atom	X	Y	Z	U(eq)
S1	0.43585(3)	0.87957(2)	0.67710(2)	0.01582(8)
P1	0.51059(3)	0.65855(3)	0.62962(2)	0.01312(8)
Si1	0.45516(3)	0.66546(3)	0.80421(2)	0.01463(9)
O1	0.37936(10)	0.92562(8)	0.60505(5)	0.0213(2)
O2	0.35330(9)	0.92565(8)	0.74264(5)	0.0211(2)
O3	0.47901(9)	0.53672(8)	0.82236(5)	0.0198(2)
N1	0.30731(11)	0.74716(9)	0.86621(6)	0.0173(2)
N2	0.52755(11)	0.71552(9)	0.86671(6)	0.0160(2)
C1	0.47623(13)	0.73586(10)	0.70141(7)	0.0151(2)
C10	0.76552(13)	0.50533(11)	0.68179(8)	0.0181(3)
C11	0.82304(14)	0.38875(11)	0.73174(8)	0.0212(3)
C12	0.83895(14)	0.29613(11)	0.68986(8)	0.0218(3)
C13	0.70284(14)	0.30966(11)	0.66217(8)	0.0229(3)
C14	0.64405(14)	0.42859(11)	0.61448(8)	0.0194(3)
C15	0.35322(12)	0.63619(10)	0.61211(7)	0.0157(3)
C16	0.29227(13)	0.55951(11)	0.68074(8)	0.0186(3)
C17	0.15930(14)	0.54603(12)	0.66485(8)	0.0241(3)

C2	0.59047(13)	0.92111(10)	0.66123(7)	0.0171(3)
C18	0.05013(14)	0.65964(13)	0.64184(9)	0.0256(3)
C19	0.11178(14)	0.73236(12)	0.57186(8)	0.0221(3)
C20	0.24230(13)	0.74975(11)	0.58751(8)	0.0187(3)
C21	0.58624(13)	0.72669(10)	0.53507(7)	0.0155(2)
C22	0.57114(14)	0.68448(12)	0.46738(7)	0.0194(3)
C23	0.62909(14)	0.74920(12)	0.39113(8)	0.0225(3)
C24	0.78029(14)	0.74507(12)	0.38870(8)	0.0218(3)
C25	0.79468(14)	0.78544(11)	0.45661(7)	0.0198(3)
C26	0.74113(13)	0.71673(11)	0.53219(7)	0.0167(3)
C27	0.39733(13)	0.76044(10)	0.90186(7)	0.0163(3)
C28	0.36373(13)	0.82113(11)	0.96541(7)	0.0179(3)
C29	0.36184(14)	0.93373(12)	0.94546(8)	0.0220(3)
C30	0.33229(15)	0.99512(12)	1.00165(9)	0.0264(3)
C31	0.30734(15)	0.94347(13)	1.07794(9)	0.0287(3)
C32	0.30978(16)	0.83162(13)	1.09798(8)	0.0282(3)
C33	0.33732(14)	0.76967(12)	1.04188(8)	0.0231(3)
C34	0.15142(13)	0.79270(12)	0.87650(8)	0.0210(3)
C35	0.10573(15)	0.76433(15)	0.81275(9)	0.0332(4)
C36	0.09728(15)	0.92032(13)	0.87034(10)	0.0335(4)
C37	0.09123(15)	0.73337(15)	0.95372(9)	0.0328(4)
C38	0.65914(13)	0.66651(11)	0.90206(8)	0.0182(3)
C39	0.69255(14)	0.75313(12)	0.93162(8)	0.0218(3)
C40	0.64885(15)	0.56517(12)	0.96729(8)	0.0260(3)
C41	0.77606(14)	0.62591(13)	0.83900(8)	0.0250(3)
C42	0.3376(2)	0.33036(17)	0.84273(10)	0.0464(5)
C43	0.37049(19)	0.23376(16)	0.81526(9)	0.0391(4)
C44	0.26690(18)	0.18966(14)	0.81374(9)	0.0351(4)
C45	0.12989(18)	0.24070(15)	0.84083(10)	0.0377(4)
C46	0.0949(2)	0.33709(16)	0.86832(10)	0.0453(4)
C47	0.1993(3)	0.38263(16)	0.86906(10)	0.0514(5)
C3	0.64969(14)	0.96108(11)	0.58757(8)	0.0203(3)
C4	0.76914(14)	0.99406(11)	0.57737(8)	0.0226(3)
C5	0.82983(14)	0.98981(11)	0.63937(8)	0.0222(3)
C6	0.76618(15)	0.95235(12)	0.71289(8)	0.0246(3)
C7	0.64813(14)	0.91806(12)	0.72392(8)	0.0223(3)
C8	0.96018(15)	1.02453(12)	0.62754(9)	0.0285(3)
C9	0.62293(13)	0.51540(10)	0.66280(7)	0.0153(2)

Table S15. Anisotropic displacement parameters(Å^2) for **6**. The anisotropic displacement factor exponent takes the form: $-2\pi^2[h^2a^{*2}U^{11} + ...+ 2 h k a^* b^* U^{12}]$.

atom	U^{11}	U^{22}	U^{33}	U^{23}	U^{13}	U^{12}
S1	0.01663(15)	0.01413(15)	0.01647(16)	-0.00567(11)	-0.00192(11)	-0.00281(11)
P1	0.01193(15)	0.01421(15)	0.01396(16)	-0.00495(12)	-0.00215(11)	-0.00319(12)
Si1	0.01384(17)	0.01711(18)	0.01446(17)	-0.00591(13)	-0.00170(13)	-0.00483(13)
O1	0.0227(5)	0.0174(4)	0.0224(5)	-0.0042(4)	-0.0075(4)	-0.0017(4)
O2	0.0216(5)	0.0180(5)	0.0224(5)	-0.0087(4)	0.0018(4)	-0.0040(4)
O3	0.0230(5)	0.0201(5)	0.0179(5)	-0.0061(4)	-0.0022(4)	-0.0075(4)
N1	0.0145(5)	0.0208(5)	0.0176(5)	-0.0070(4)	-0.0010(4)	-0.0051(4)

N2	0.0154(5)	0.0182(5)	0.0160(5)	-0.0063(4)	-0.0023(4)	-0.0050(4)
C1	0.0152(6)	0.0148(6)	0.0162(6)	-0.0058(5)	-0.0021(5)	-0.0037(5)
C10	0.0162(6)	0.0182(6)	0.0208(7)	-0.0046(5)	-0.0054(5)	-0.0044(5)
C11	0.0198(6)	0.0203(7)	0.0225(7)	-0.0032(5)	-0.0075(5)	-0.0031(5)
C12	0.0200(7)	0.0175(6)	0.0243(7)	-0.0048(5)	-0.0041(5)	-0.0002(5)
C13	0.0221(7)	0.0167(6)	0.0309(8)	-0.0094(6)	-0.0042(6)	-0.0035(5)
C14	0.0179(6)	0.0192(6)	0.0230(7)	-0.0101(5)	-0.0051(5)	-0.0023(5)
C15	0.0130(6)	0.0180(6)	0.0173(6)	-0.0063(5)	-0.0028(5)	-0.0039(5)
C16	0.0168(6)	0.0216(7)	0.0196(7)	-0.0049(5)	-0.0035(5)	-0.0076(5)
C17	0.0213(7)	0.0291(7)	0.0265(7)	-0.0054(6)	-0.0052(5)	-0.0125(6)
C2	0.0198(6)	0.0128(6)	0.0188(6)	-0.0056(5)	-0.0010(5)	-0.0044(5)
C18	0.0147(6)	0.0349(8)	0.0311(8)	-0.0113(6)	-0.0044(5)	-0.0084(6)
C19	0.0167(6)	0.0251(7)	0.0257(7)	-0.0097(6)	-0.0077(5)	-0.0018(5)
C20	0.0153(6)	0.0194(6)	0.0213(7)	-0.0059(5)	-0.0049(5)	-0.0025(5)
C21	0.0155(6)	0.0163(6)	0.0146(6)	-0.0042(5)	-0.0018(5)	-0.0042(5)
C22	0.0182(6)	0.0255(7)	0.0168(6)	-0.0069(5)	-0.0017(5)	-0.0081(5)
C23	0.0247(7)	0.0285(7)	0.0153(7)	-0.0054(5)	-0.0024(5)	-0.0088(6)
C24	0.0222(7)	0.0258(7)	0.0176(7)	-0.0063(5)	0.0019(5)	-0.0094(5)
C25	0.0192(6)	0.0212(6)	0.0196(7)	-0.0048(5)	0.0000(5)	-0.0081(5)
C26	0.0159(6)	0.0179(6)	0.0171(6)	-0.0050(5)	-0.0029(5)	-0.0048(5)
C27	0.0181(6)	0.0154(6)	0.0148(6)	-0.0021(5)	-0.0013(5)	-0.0058(5)
C28	0.0141(6)	0.0222(7)	0.0190(7)	-0.0084(5)	-0.0016(5)	-0.0050(5)
C29	0.0227(7)	0.0224(7)	0.0212(7)	-0.0071(5)	-0.0021(5)	-0.0058(5)
C30	0.0259(7)	0.0225(7)	0.0336(8)	-0.0131(6)	-0.0031(6)	-0.0060(6)
C31	0.0266(7)	0.0362(8)	0.0294(8)	-0.0208(7)	0.0008(6)	-0.0093(6)
C32	0.0306(8)	0.0398(9)	0.0173(7)	-0.0112(6)	0.0028(6)	-0.0147(7)
C33	0.0238(7)	0.0258(7)	0.0223(7)	-0.0073(6)	-0.0001(5)	-0.0110(6)
C34	0.0127(6)	0.0282(7)	0.0223(7)	-0.0096(6)	-0.0003(5)	-0.0048(5)
C35	0.0163(7)	0.0532(10)	0.0345(9)	-0.0223(7)	-0.0045(6)	-0.0054(6)
C36	0.0170(7)	0.0311(8)	0.0498(10)	-0.0161(7)	0.0005(6)	-0.0014(6)
C37	0.0197(7)	0.0484(10)	0.0287(8)	-0.0046(7)	0.0001(6)	-0.0132(7)
C38	0.0165(6)	0.0200(6)	0.0200(7)	-0.0056(5)	-0.0062(5)	-0.0047(5)
C39	0.0209(7)	0.0244(7)	0.0243(7)	-0.0075(6)	-0.0062(5)	-0.0084(5)
C40	0.0288(7)	0.0221(7)	0.0285(8)	-0.0009(6)	-0.0113(6)	-0.0083(6)
C41	0.0166(6)	0.0309(8)	0.0284(8)	-0.0121(6)	-0.0051(5)	-0.0028(6)
C42	0.0689(13)	0.0527(11)	0.0309(9)	0.0128(8)	-0.0257(9)	-0.0399(10)
C43	0.0395(9)	0.0523(11)	0.0234(8)	0.0055(7)	-0.0079(7)	-0.0196(8)
C44	0.0493(10)	0.0313(8)	0.0253(8)	0.0032(6)	-0.0161(7)	-0.0136(7)
C45	0.0406(9)	0.0395(9)	0.0328(9)	0.0103(7)	-0.0166(7)	-0.0187(8)
C46	0.0480(10)	0.0452(10)	0.0275(9)	0.0048(7)	-0.0071(7)	-0.0040(8)
C47	0.0998(17)	0.0322(9)	0.0251(9)	-0.0004(7)	-0.0261(10)	-0.0173(10)
C3	0.0259(7)	0.0152(6)	0.0185(7)	-0.0054(5)	-0.0021(5)	-0.0040(5)
C4	0.0264(7)	0.0161(6)	0.0216(7)	-0.0044(5)	0.0045(5)	-0.0066(5)
C5	0.0218(7)	0.0145(6)	0.0290(7)	-0.0080(5)	0.0024(5)	-0.0051(5)
C6	0.0272(7)	0.0271(7)	0.0235(7)	-0.0071(6)	-0.0042(6)	-0.0115(6)
C7	0.0261(7)	0.0242(7)	0.0181(7)	-0.0038(5)	-0.0004(5)	-0.0115(6)
C8	0.0251(7)	0.0236(7)	0.0374(8)	-0.0100(6)	0.0039(6)	-0.0106(6)
C9	0.0147(6)	0.0150(6)	0.0164(6)	-0.0057(5)	-0.0024(5)	-0.0028(5)

6.8 Crystal structure determination of Siloxane 7

Additional information concerning the structure refinement: The unit cell contained a disordered benzene molecule. The disorder (occupancy 0.68:0.32) was solved using the PART instructions.

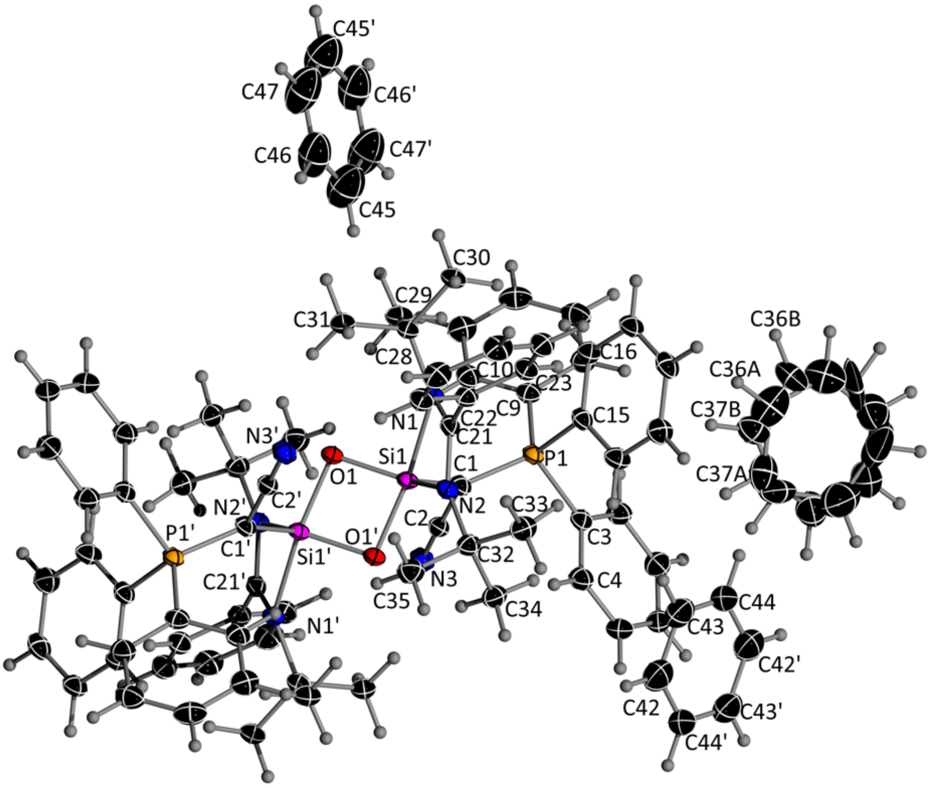

Figure S81. ORTEP of **7**. Ellipsoids are drawn at the 50% probability level.

Table S16. Atomic coordinates (x 10^4) and equivalent isotropic displacement parameters ($Å^2$x 10^3) for **7**. U(eq) is defined as one third of the trace of the orthogonalized U^{ij} tensor for all atoms.

Atom	X	Y	Z	U(eq)
P1	0.30797(2)	0.41919(2)	0.37431(2)	0.01924(11)
Si1	0.29340(2)	0.30185(2)	0.26924(2)	0.01769(11)
O1	0.28629(5)	0.19840(6)	0.26613(4)	0.0197(2)
N1	0.39461(6)	0.29445(7)	0.28416(5)	0.0202(3)
N2	0.33712(6)	0.36722(8)	0.22565(5)	0.0212(3)
N3	0.14752(6)	0.33155(8)	0.34768(5)	0.0257(3)
C1	0.27016(7)	0.35457(9)	0.32761(5)	0.0205(3)
C9	0.32985(7)	0.37145(10)	0.43705(6)	0.0226(3)
C10	0.31362(8)	0.28821(10)	0.44257(6)	0.0256(3)
C11	0.33029(8)	0.24768(11)	0.48897(7)	0.0319(4)
C12	0.36207(9)	0.29080(12)	0.53076(6)	0.0338(4)
C13	0.37774(9)	0.37420(11)	0.52593(6)	0.0335(4)
C2	0.20234(7)	0.34156(9)	0.33754(5)	0.0208(3)
C14	0.36311(8)	0.41444(10)	0.47920(6)	0.0282(3)
C15	0.38573(7)	0.46929(9)	0.36047(6)	0.0219(3)
C16	0.44863(7)	0.44371(10)	0.38492(6)	0.0245(3)
C17	0.50720(8)	0.48759(10)	0.37766(6)	0.0273(3)
C18	0.50387(8)	0.55685(10)	0.34626(6)	0.0281(3)

C19	0.44163(8)	0.58161(10)	0.32125(6)	0.0297(3)
C20	0.38282(8)	0.53828(10)	0.32817(6)	0.0268(3)
C21	0.40002(7)	0.33940(9)	0.24295(6)	0.0207(3)
C22	0.46239(7)	0.35494(9)	0.21718(6)	0.0228(3)
C23	0.51074(8)	0.41284(10)	0.23707(6)	0.0266(3)
C24	0.56668(8)	0.42895(10)	0.21137(7)	0.0310(4)
C25	0.57447(8)	0.38782(11)	0.16613(7)	0.0336(4)
C26	0.52681(8)	0.32914(11)	0.14654(7)	0.0319(4)
C27	0.47047(8)	0.31254(10)	0.17195(6)	0.0258(3)
C28	0.44235(7)	0.22953(9)	0.30688(6)	0.0225(3)
C29	0.41520(8)	0.20018(10)	0.35592(6)	0.0273(3)
C30	0.51515(8)	0.26188(10)	0.32085(7)	0.0281(3)
C31	0.44391(8)	0.15627(10)	0.26989(6)	0.0262(3)
C32	0.31445(8)	0.42771(9)	0.18451(6)	0.0239(3)
C33	0.36939(8)	0.49147(10)	0.17474(7)	0.0303(4)
C34	0.29117(9)	0.37987(11)	0.13518(6)	0.0309(4)
C35	0.25426(8)	0.47634(10)	0.20135(6)	0.0273(3)
C42	0.24494(13)	0.71247(13)	0.20287(9)	0.0564(6)
C43	0.30377(11)	0.70422(13)	0.23638(10)	0.0518(5)
C44	0.30801(11)	0.74163(12)	0.28344(10)	0.0543(6)
C45	0.44863(18)	0.04760(17)	0.47509(10)	0.0750(8)
C46	0.49073(17)	0.00323(16)	0.44739(9)	0.0698(7)
C47	0.54163(18)	-0.04430(17)	0.47197(10)	0.0747(8)
C3	0.24913(7)	0.50399(9)	0.38346(6)	0.0216(3)
C4	0.21342(8)	0.53959(10)	0.33970(6)	0.0263(3)
C5	0.17125(8)	0.60753(10)	0.34423(6)	0.0297(3)
C6	0.16312(8)	0.63940(10)	0.39215(7)	0.0291(3)
C7	0.19613(8)	0.60264(11)	0.43563(7)	0.0304(3)
C8	0.23951(8)	0.53536(10)	0.43130(6)	0.0268(3)
C36A	0.4231(4)	0.6724(2)	0.4570(3)	0.059(2)
C37A	0.3597(3)	0.6974(3)	0.43377(18)	0.0463(18)
C38A	0.32850(15)	0.7672(4)	0.45171(18)	0.0532(14)
C39A	0.3607(2)	0.8120(2)	0.4928(2)	0.0628(16)
C40A	0.4241(2)	0.7870(4)	0.51606(13)	0.0605(17)
C41A	0.45530(17)	0.7172(5)	0.4981(2)	0.056(2)
C36B	0.4491(3)	0.6796(6)	0.4836(4)	0.043(3)
C41B	0.4498(8)	0.7523(8)	0.5123(3)	0.074(6)
C40B	0.3978(11)	0.8101(4)	0.5022(5)	0.086(7)
C39B	0.3451(7)	0.7953(7)	0.4635(7)	0.076(5)
C38B	0.3444(4)	0.7226(9)	0.4348(5)	0.050(4)
C37B	0.3964(6)	0.6647(5)	0.4448(4)	0.042(3)

Table S17. Anisotropic displacement parameters(Å^2) for **7**. The anisotropic displacement factor exponent takes the form: $-2\pi^2[h^2a^{*2}U^{11} + ...+ 2 h k a^* b^* U^{12}]$.

atom	U^{11}	U^{22}	U^{33}	U^{23}	U^{13}	U^{12}
P1	0.01341(18)	0.0218(2)	0.0223(2)	-0.00203(13)	0.00133(13)	-0.00058(13)
Si1	0.01178(19)	0.0198(2)	0.0214(2)	-0.00119(14)	0.00145(14)	-0.00063(14)
O1	0.0136(5)	0.0214(5)	0.0236(5)	-0.0009(4)	0.0004(4)	-0.0003(4)

N1	0.0134(6)	0.0215(6)	0.0257(6)	-0.0017(5)	0.0016(5)	-0.0002(5)
N2	0.0152(6)	0.0229(6)	0.0256(6)	-0.0002(5)	0.0024(5)	-0.0009(5)
N3	0.0179(6)	0.0300(7)	0.0292(7)	-0.0043(5)	0.0029(5)	-0.0013(5)
C1	0.0142(7)	0.0234(7)	0.0236(7)	-0.0024(6)	0.0012(5)	-0.0019(5)
C9	0.0171(7)	0.0268(7)	0.0241(7)	-0.0005(6)	0.0032(5)	0.0024(6)
C10	0.0195(7)	0.0295(8)	0.0283(8)	-0.0013(6)	0.0045(6)	-0.0005(6)
C11	0.0279(9)	0.0339(9)	0.0346(9)	0.0066(7)	0.0073(7)	0.0011(7)
C12	0.0302(9)	0.0444(10)	0.0270(8)	0.0074(7)	0.0038(7)	0.0077(7)
C13	0.0302(9)	0.0429(10)	0.0260(8)	-0.0044(7)	-0.0019(7)	0.0055(7)
C2	0.0178(7)	0.0217(7)	0.0224(7)	-0.0031(6)	0.0002(5)	0.0007(5)
C14	0.0249(8)	0.0299(8)	0.0293(8)	-0.0037(6)	0.0009(6)	0.0028(6)
C15	0.0163(7)	0.0250(7)	0.0245(7)	-0.0049(6)	0.0027(5)	-0.0021(6)
C16	0.0192(7)	0.0243(7)	0.0297(8)	-0.0026(6)	0.0013(6)	0.0005(6)
C17	0.0153(7)	0.0317(8)	0.0341(8)	-0.0055(7)	-0.0001(6)	0.0002(6)
C18	0.0189(7)	0.0328(8)	0.0329(8)	-0.0060(7)	0.0050(6)	-0.0068(6)
C19	0.0246(8)	0.0310(8)	0.0338(9)	0.0040(7)	0.0052(6)	-0.0035(6)
C20	0.0174(7)	0.0323(8)	0.0301(8)	0.0013(6)	0.0008(6)	-0.0010(6)
C21	0.0159(7)	0.0203(7)	0.0261(7)	-0.0046(6)	0.0028(6)	-0.0017(5)
C22	0.0162(7)	0.0228(7)	0.0296(8)	0.0023(6)	0.0036(6)	0.0006(6)
C23	0.0195(7)	0.0259(8)	0.0346(8)	-0.0006(6)	0.0033(6)	-0.0009(6)
C24	0.0189(8)	0.0272(8)	0.0472(10)	0.0035(7)	0.0048(7)	-0.0033(6)
C25	0.0213(8)	0.0358(9)	0.0458(10)	0.0067(8)	0.0130(7)	-0.0007(7)
C26	0.0275(8)	0.0350(9)	0.0351(9)	0.0000(7)	0.0118(7)	0.0031(7)
C27	0.0203(7)	0.0260(8)	0.0315(8)	-0.0003(6)	0.0044(6)	-0.0007(6)
C28	0.0146(7)	0.0241(7)	0.0282(8)	-0.0001(6)	0.0008(6)	0.0006(6)
C29	0.0194(7)	0.0324(8)	0.0298(8)	0.0031(6)	0.0010(6)	0.0033(6)
C30	0.0161(7)	0.0298(8)	0.0373(9)	-0.0020(7)	-0.0017(6)	0.0005(6)
C31	0.0198(7)	0.0260(8)	0.0325(8)	-0.0018(6)	0.0012(6)	0.0037(6)
C32	0.0203(7)	0.0231(7)	0.0283(8)	0.0036(6)	0.0032(6)	0.0004(6)
C33	0.0252(8)	0.0283(8)	0.0381(9)	0.0073(7)	0.0069(7)	-0.0004(6)
C34	0.0320(9)	0.0322(9)	0.0279(8)	0.0019(7)	0.0007(7)	0.0025(7)
C35	0.0225(8)	0.0246(8)	0.0350(8)	0.0039(6)	0.0040(6)	0.0027(6)
C42	0.0700(15)	0.0368(11)	0.0578(13)	0.0014(10)	-0.0110(11)	0.0063(10)
C43	0.0453(12)	0.0324(10)	0.0761(15)	0.0069(10)	-0.0001(10)	0.0031(8)
C44	0.0463(12)	0.0324(10)	0.0766(16)	0.0036(10)	-0.0241(11)	-0.0003(9)
C45	0.109(2)	0.0569(15)	0.0586(15)	0.0038(12)	0.0099(15)	0.0102(15)
C46	0.116(2)	0.0500(13)	0.0420(12)	-0.0030(10)	0.0058(13)	-0.0041(14)
C47	0.118(2)	0.0527(14)	0.0560(15)	-0.0036(12)	0.0223(15)	0.0092(15)
C3	0.0145(7)	0.0228(7)	0.0277(7)	-0.0025(6)	0.0029(5)	-0.0022(5)
C4	0.0213(7)	0.0297(8)	0.0278(8)	-0.0026(6)	0.0015(6)	0.0017(6)
C5	0.0218(8)	0.0307(8)	0.0352(9)	0.0013(7)	-0.0026(6)	0.0041(6)
C6	0.0194(7)	0.0262(8)	0.0412(9)	-0.0046(7)	0.0016(6)	0.0032(6)
C7	0.0255(8)	0.0340(9)	0.0320(8)	-0.0080(7)	0.0046(6)	0.0038(7)
C8	0.0224(7)	0.0303(8)	0.0275(8)	-0.0017(6)	0.0018(6)	0.0032(6)
C36A	0.073(6)	0.052(2)	0.057(5)	0.007(2)	0.039(4)	0.007(3)
C37A	0.052(4)	0.039(3)	0.052(3)	-0.003(2)	0.021(3)	-0.010(3)
C38A	0.051(3)	0.050(3)	0.058(3)	-0.009(2)	0.006(2)	-0.005(2)
C39A	0.063(4)	0.054(3)	0.069(3)	-0.015(2)	-0.002(3)	-0.002(2)
C40A	0.054(3)	0.059(5)	0.069(3)	-0.010(3)	0.008(2)	-0.010(3)

C41A	0.048(3)	0.077(6)	0.047(3)	0.002(3)	0.014(2)	0.002(3)
C36B	0.031(4)	0.061(7)	0.036(6)	-0.007(5)	-0.003(4)	-0.009(4)
C41B	0.122(15)	0.052(9)	0.044(6)	-0.020(6)	-0.007(8)	-0.047(9)
C40B	0.13(2)	0.037(5)	0.093(11)	-0.017(6)	0.013(11)	0.017(9)
C39B	0.057(7)	0.062(9)	0.108(15)	0.013(9)	0.009(8)	0.008(7)
C38B	0.030(4)	0.048(8)	0.069(9)	0.027(6)	-0.003(4)	0.002(5)
C37B	0.039(7)	0.050(5)	0.034(4)	0.008(3)	-0.001(4)	-0.005(5)

6.8 Crystal structure determination of Carbonate Complex 8

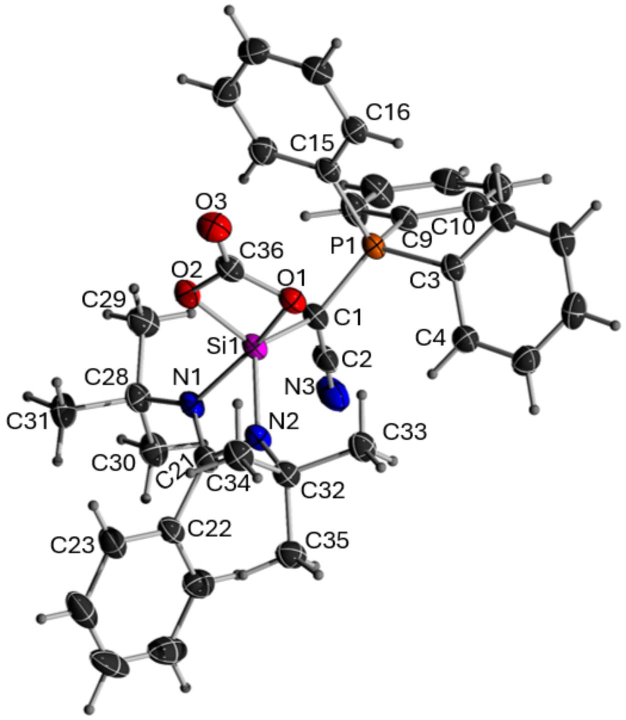

Figure S82. ORTEP of **8**. Ellipsoids are drawn at the 50% probability level.

Table S18. Atomic coordinates (x 104) and equivalentisotropic displacement parameters (Å2x 103) for **8** U(eq) is defined as one third of the trace of the orthogonalized Uij tensor for all atoms.

Atom	x	y	z	U(eq)
Si(1)	8582(1)	5765(1)	6885(1)	22(1)
O(1)	9583(1)	5691(1)	7911(1)	26(1)
N(1)	8022(1)	5804(1)	5632(1)	24(1)
C(1)	7263(1)	5821(1)	7381(1)	24(1)
C(3)	7666(1)	6322(1)	9290(1)	24(1)
C(4)	7883(1)	7006(1)	8929(1)	30(1)
C(5)	8250(1)	7592(1)	9494(1)	36(1)
C(6)	8411(1)	7501(1)	10422(1)	34(1)
C(7)	8190(1)	6827(1)	10790(1)	33(1)
C(8)	7809(1)	6239(1)	10234(1)	28(1)
C(9)	5652(1)	5472(1)	8602(1)	25(1)

C(10)	5160(1)	5799(1)	9299(1)	30(1)
C(11)	4000(1)	5709(1)	9344(1)	33(1)
C(12)	3322(1)	5303(1)	8693(1)	33(1)
C(13)	3811(1)	4955(1)	8018(1)	36(1)
C(14)	4971(1)	5038(1)	7968(1)	34(1)
P(1)	7153(1)	5603(1)	8493(1)	21(1)
C(15)	7875(1)	4733(1)	8801(1)	24(1)
C(16)	8628(1)	4620(1)	9599(1)	26(1)
C(2)	6301(1)	6168(1)	6891(1)	28(1)
O(3)	10706(1)	4660(1)	7976(1)	35(1)
N(3)	5520(1)	6452(1)	6476(1)	43(1)
C(17)	9089(1)	3926(1)	9804(1)	30(1)
C(18)	8818(1)	3341(1)	9218(1)	32(1)
C(19)	8087(1)	3452(1)	8416(1)	33(1)
C(20)	7618(1)	4143(1)	8207(1)	31(1)
C(21)	8663(1)	6385(1)	5540(1)	24(1)
C(22)	8830(1)	6758(1)	4680(1)	28(1)
C(23)	9728(1)	6543(1)	4225(1)	36(1)
C(24)	9902(2)	6912(1)	3437(1)	49(1)
C(25)	9200(2)	7485(1)	3118(1)	54(1)
C(26)	8293(2)	7689(1)	3560(1)	49(1)
C(27)	8098(2)	7325(1)	4343(1)	36(1)
C(28)	7311(1)	5357(1)	4929(1)	28(1)
C(29)	6806(2)	4728(1)	5428(1)	38(1)
O(2)	9308(1)	4946(1)	6795(1)	26(1)
N(2)	9169(1)	6573(1)	6384(1)	24(1)
C(30)	6341(1)	5818(1)	4420(1)	34(1)
C(31)	8055(1)	5033(1)	4266(1)	35(1)
C(32)	10105(1)	7115(1)	6661(1)	26(1)
C(33)	10006(1)	7370(1)	7625(1)	31(1)
C(34)	11255(1)	6733(1)	6629(1)	32(1)
C(35)	10018(1)	7804(1)	6061(1)	33(1)
C(36)	9961(1)	5052(1)	7618(1)	27(1)

Table S19. Anisotropic displacement parameters($Å^2$) for **8**The anisotropic displacement factor exponent takes the form: $-2\pi^2[h^2a^{*2}U^{11} + ...+ 2 h k a^* b^* U^{12}]$.

Atom	U^{11}	U^{22}	U^{33}	U^{23}	U^{13}	U^{12}

Si(1)	20(1)	28(1)	17(1)	0(1)	3(1)	-2(1)
O(1)	25(1)	32(1)	21(1)	0(1)	3(1)	-3(1)
N(1)	23(1)	31(1)	18(1)	-1(1)	2(1)	-2(1)
C(1)	22(1)	30(1)	19(1)	1(1)	4(1)	0(1)
C(3)	20(1)	29(1)	23(1)	-2(1)	4(1)	0(1)
C(4)	32(1)	30(1)	28(1)	0(1)	8(1)	0(1)
C(5)	40(1)	28(1)	42(1)	-4(1)	14(1)	-5(1)
C(6)	25(1)	36(1)	40(1)	-14(1)	6(1)	-2(1)
C(7)	30(1)	43(1)	26(1)	-8(1)	3(1)	2(1)
C(8)	28(1)	32(1)	25(1)	-1(1)	5(1)	0(1)
C(9)	21(1)	31(1)	23(1)	3(1)	4(1)	-2(1)
C(10)	26(1)	34(1)	30(1)	-2(1)	7(1)	-2(1)
C(11)	29(1)	38(1)	36(1)	1(1)	11(1)	0(1)
C(12)	23(1)	41(1)	36(1)	10(1)	6(1)	-3(1)
C(13)	26(1)	51(1)	29(1)	-2(1)	-1(1)	-10(1)
C(14)	28(1)	47(1)	26(1)	-6(1)	4(1)	-5(1)
P(1)	19(1)	27(1)	18(1)	0(1)	4(1)	-2(1)
C(15)	20(1)	29(1)	23(1)	0(1)	6(1)	-3(1)
C(16)	22(1)	33(1)	24(1)	-1(1)	5(1)	-2(1)
C(2)	29(1)	37(1)	21(1)	1(1)	8(1)	3(1)
O(3)	26(1)	42(1)	35(1)	6(1)	-1(1)	3(1)
N(3)	40(1)	60(1)	30(1)	8(1)	8(1)	20(1)
C(17)	22(1)	39(1)	29(1)	6(1)	3(1)	1(1)
C(18)	25(1)	31(1)	41(1)	6(1)	9(1)	2(1)
C(19)	34(1)	30(1)	36(1)	-4(1)	7(1)	-2(1)
C(20)	31(1)	33(1)	27(1)	-1(1)	1(1)	-2(1)
C(21)	21(1)	30(1)	21(1)	0(1)	4(1)	2(1)
C(22)	31(1)	33(1)	19(1)	0(1)	3(1)	-5(1)
C(23)	40(1)	44(1)	27(1)	-4(1)	11(1)	-7(1)
C(24)	60(1)	63(1)	28(1)	-9(1)	19(1)	-25(1)
C(25)	85(2)	53(1)	21(1)	6(1)	0(1)	-32(1)
C(26)	71(1)	38(1)	31(1)	7(1)	-15(1)	-12(1)
C(27)	42(1)	35(1)	28(1)	2(1)	-5(1)	-2(1)
C(28)	27(1)	34(1)	21(1)	-4(1)	0(1)	-3(1)
C(29)	43(1)	40(1)	29(1)	-2(1)	-1(1)	-14(1)
O(2)	23(1)	33(1)	22(1)	-1(1)	2(1)	1(1)
N(2)	24(1)	31(1)	19(1)	0(1)	4(1)	-4(1)

C(30)	28(1)	45(1)	27(1)	-5(1)	-2(1)	0(1)
C(31)	35(1)	41(1)	28(1)	-9(1)	2(1)	2(1)
C(32)	24(1)	31(1)	24(1)	-1(1)	3(1)	-6(1)
C(33)	33(1)	35(1)	26(1)	-4(1)	5(1)	-6(1)
C(34)	25(1)	40(1)	32(1)	-1(1)	5(1)	-4(1)
C(35)	36(1)	33(1)	29(1)	2(1)	4(1)	-6(1)
C(36)	21(1)	35(1)	24(1)	2(1)	4(1)	-4(1)

6.9 Crystal structure determination of Silazine 9_{Ts}

Additional information concerning the structure refinement: The unit cell contained a disordered toluene molecule. The disorder (occupancy 0.57:0.43) was solved using the PART instructions.

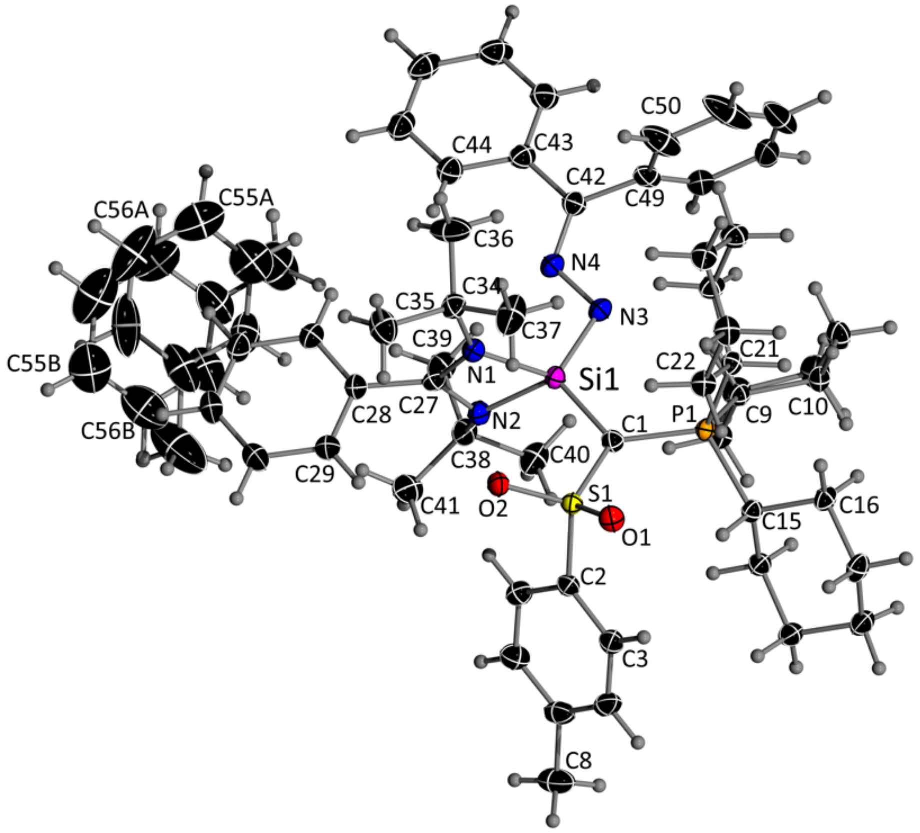

Figure S83. ORTEP of $\mathbf{9_{Ts}}$. Ellipsoids are drawn at the 50% probability level.

Table S20. Atomic coordinates (x 10^4) and equivalent isotropic displacement parameters (Å^{2}x 10^3) for $\mathbf{9_{Ts}}$. U(eq) is defined as one third of the trace of the orthogonalized U^{ij} tensor for all atoms.

Atom	X	Y	Z	U(eq)
Si1	0.35941(4)	0.68631(3)	0.24412(2)	0.01783(9)
O1	0.35264(10)	0.90943(8)	0.07104(5)	0.0228(2)
N2	0.40429(12)	0.75420(10)	0.30526(6)	0.0215(3)
S1	0.38810(3)	0.88173(3)	0.13662(2)	0.01788(9)

P1	0.46789(3)	0.66631(3)	0.11205(2)	0.01544(9)
O2	0.29698(10)	0.91998(8)	0.17905(5)	0.0225(2)
N1	0.20449(12)	0.74534(10)	0.27100(6)	0.0206(3)
N3	0.36445(13)	0.55149(10)	0.26123(6)	0.0218(3)
N4	0.32025(12)	0.51525(10)	0.32206(6)	0.0220(3)
C3	0.61255(16)	0.99462(11)	0.08791(7)	0.0246(3)
C4	0.72983(16)	1.04699(12)	0.09322(8)	0.0291(4)
C1	0.41602(14)	0.74562(11)	0.16391(7)	0.0181(3)
C2	0.53934(15)	0.95014(11)	0.14107(7)	0.0206(3)
C5	0.77429(16)	1.05799(12)	0.15088(8)	0.0294(4)
C6	0.69660(17)	1.01610(13)	0.20334(8)	0.0299(4)
C7	0.58057(16)	0.96197(12)	0.19879(7)	0.0259(3)
C8	0.90332(18)	1.11320(14)	0.15662(10)	0.0406(4)
C9	0.56449(14)	0.54724(11)	0.15407(6)	0.0177(3)
C10	0.68409(15)	0.56798(12)	0.19225(7)	0.0214(3)
C11	0.73162(17)	0.46219(13)	0.23573(7)	0.0273(3)
C12	0.77288(18)	0.37901(13)	0.19866(8)	0.0315(4)
C13	0.66011(17)	0.36225(12)	0.15603(7)	0.0272(3)
C14	0.60901(15)	0.46893(11)	0.11369(7)	0.0206(3)
C15	0.32646(14)	0.61132(11)	0.07765(6)	0.0180(3)
C16	0.25041(15)	0.52508(12)	0.12460(7)	0.0225(3)
C17	0.13388(15)	0.48510(13)	0.09274(8)	0.0263(3)
C18	0.03888(15)	0.57695(13)	0.06012(8)	0.0265(3)
C19	0.11597(15)	0.65868(13)	0.01222(7)	0.0258(3)
C20	0.23118(15)	0.70233(12)	0.04294(7)	0.0221(3)
C21	0.56352(14)	0.74199(11)	0.04438(6)	0.0185(3)
C22	0.70997(14)	0.75966(11)	0.06025(7)	0.0203(3)
C23	0.77840(15)	0.83427(12)	0.00498(7)	0.0246(3)
C24	0.77529(16)	0.78945(13)	-0.05319(7)	0.0268(3)
C25	0.63285(16)	0.76544(13)	-0.06855(7)	0.0247(3)
C26	0.56222(15)	0.69306(12)	-0.01319(7)	0.0213(3)
C27	0.27325(15)	0.77903(11)	0.31310(7)	0.0204(3)
C28	0.22110(15)	0.84396(12)	0.35643(7)	0.0236(3)
C29	0.22828(18)	0.95528(13)	0.33496(8)	0.0321(4)
C30	0.1811(2)	1.02180(14)	0.37188(9)	0.0394(4)
C31	0.12876(19)	0.97833(14)	0.43028(9)	0.0372(4)
C32	0.12201(17)	0.86803(14)	0.45194(8)	0.0316(4)
C33	0.16745(16)	0.80031(13)	0.41476(7)	0.0252(3)
C34	0.05843(15)	0.74771(12)	0.25890(7)	0.0237(3)
C35	0.00265(18)	0.64481(15)	0.29960(11)	0.0436(5)
C36	0.04166(18)	0.74965(19)	0.19033(9)	0.0452(5)
C37	-0.01639(18)	0.84594(14)	0.27201(9)	0.0368(4)
C38	0.51308(15)	0.74007(13)	0.35296(7)	0.0256(3)
C39	0.54219(19)	0.84530(15)	0.37106(8)	0.0349(4)
C40	0.47683(17)	0.65475(14)	0.41172(8)	0.0303(4)
C41	0.63697(16)	0.70004(16)	0.32303(8)	0.0338(4)
C42	0.29243(15)	0.41459(12)	0.34089(7)	0.0224(3)
C43	0.24544(15)	0.37667(12)	0.40583(7)	0.0240(3)
C44	0.20012(16)	0.44949(13)	0.44084(8)	0.0282(3)

C45	0.15430(17)	0.41344(15)	0.50133(8)	0.0322(4)
C46	0.15161(18)	0.30450(15)	0.52890(8)	0.0343(4)
C47	0.19704(19)	0.23166(14)	0.49529(8)	0.0343(4)
C48	0.24251(17)	0.26700(13)	0.43455(8)	0.0298(4)
C49	0.29999(18)	0.33672(12)	0.29960(7)	0.0284(4)
C50	0.4135(2)	0.27270(14)	0.29687(9)	0.0404(4)
C51	0.4144(3)	0.19651(16)	0.26077(11)	0.0601(7)
C52	0.3032(4)	0.18472(17)	0.22753(9)	0.0682(9)
C53	0.1917(3)	0.24898(17)	0.22908(10)	0.0667(8)
C54	0.1882(2)	0.32505(14)	0.26508(9)	0.0451(5)
C55A	0.3259(10)	0.8287(10)	0.5812(6)	0.059(2)
C56A	0.3563(5)	0.7300(5)	0.5655(3)	0.0484(12)
C57A	0.2662(5)	0.6480(5)	0.5788(2)	0.0557(14)
C58A	0.1478(5)	0.6552(5)	0.6064(2)	0.0624(16)
C59A	0.1142(8)	0.7520(11)	0.6245(3)	0.076(3)
C60A	0.2046(7)	0.8347(6)	0.6123(3)	0.071(2)
C61A	0.4233(8)	0.9179(5)	0.5624(4)	0.104(3)
C55B	0.2939(11)	0.7186(5)	0.5909(3)	0.057(2)
C56B	0.1686(10)	0.7291(10)	0.6223(5)	0.061(3)
C57B	0.1341(13)	0.8284(10)	0.6337(4)	0.079(3)
C58B	0.2124(8)	0.9166(7)	0.6140(3)	0.071(2)
C59B	0.3278(10)	0.9079(7)	0.5834(4)	0.073(2)
C60B	0.3641(15)	0.8042(14)	0.5750(8)	0.060(3)
C61B	0.3343(13)	0.6135(7)	0.5820(3)	0.076(3)

Table S21. Anisotropic displacement parameters(Å^2) for **9$_{Ts}$**. The anisotropic displacement factor exponent takes the form: $-2\pi^2[h^2a^{*2}U^{11} + ...+ 2 h k a^* b^* U^{12}]$.

atom	U^{11}	U^{22}	U^{33}	U^{23}	U^{13}	U^{12}
Si1	0.01913(19)	0.01897(19)	0.01566(19)	-0.00452(15)	0.00133(14)	-0.00180(14)
O1	0.0285(5)	0.0201(5)	0.0182(5)	-0.0020(4)	-0.0023(4)	0.0031(4)
N2	0.0223(6)	0.0250(6)	0.0187(6)	-0.0076(5)	0.0015(5)	-0.0039(5)
S1	0.02124(17)	0.01539(16)	0.01652(17)	-0.00302(13)	0.00162(13)	0.00023(12)
P1	0.01628(17)	0.01675(17)	0.01343(17)	-0.00392(13)	-0.00019(13)	-0.00012(13)
O2	0.0256(5)	0.0188(5)	0.0233(5)	-0.0059(4)	0.0052(4)	0.0017(4)
N1	0.0215(6)	0.0211(6)	0.0200(6)	-0.0062(5)	0.0032(5)	-0.0026(5)
N3	0.0254(6)	0.0224(6)	0.0173(6)	-0.0037(5)	0.0033(5)	-0.0024(5)
N4	0.0244(6)	0.0223(6)	0.0189(6)	-0.0039(5)	0.0022(5)	-0.0021(5)
C3	0.0306(8)	0.0170(7)	0.0246(8)	-0.0023(6)	0.0046(6)	0.0011(6)
C4	0.0296(8)	0.0198(7)	0.0345(9)	0.0001(6)	0.0097(7)	-0.0016(6)
C1	0.0208(7)	0.0164(7)	0.0168(7)	-0.0034(5)	-0.0004(5)	0.0002(5)
C2	0.0236(7)	0.0143(6)	0.0230(8)	-0.0030(6)	0.0028(6)	0.0003(5)
C5	0.0251(8)	0.0183(7)	0.0426(10)	-0.0025(7)	0.0022(7)	-0.0016(6)
C6	0.0306(9)	0.0284(8)	0.0312(9)	-0.0071(7)	-0.0020(7)	-0.0053(7)
C7	0.0281(8)	0.0250(8)	0.0234(8)	-0.0030(6)	0.0030(6)	-0.0041(6)
C8	0.0306(9)	0.0307(9)	0.0581(13)	-0.0046(8)	0.0014(8)	-0.0091(7)
C9	0.0196(7)	0.0175(7)	0.0156(7)	-0.0033(5)	0.0008(5)	0.0000(5)
C10	0.0218(7)	0.0236(7)	0.0185(7)	-0.0042(6)	-0.0031(6)	0.0004(6)
C11	0.0297(8)	0.0289(8)	0.0214(8)	-0.0019(6)	-0.0059(6)	0.0020(6)

C12	0.0361(9)	0.0274(8)	0.0269(9)	0.0001(7)	-0.0046(7)	0.0109(7)
C13	0.0354(9)	0.0204(7)	0.0248(8)	-0.0043(6)	0.0018(7)	0.0045(6)
C14	0.0229(7)	0.0203(7)	0.0191(7)	-0.0064(6)	0.0009(6)	0.0025(6)
C15	0.0178(7)	0.0214(7)	0.0158(7)	-0.0065(6)	-0.0009(5)	-0.0005(5)
C16	0.0198(7)	0.0266(7)	0.0212(8)	-0.0053(6)	-0.0006(6)	-0.0038(6)
C17	0.0226(8)	0.0300(8)	0.0270(8)	-0.0074(7)	-0.0013(6)	-0.0065(6)
C18	0.0184(7)	0.0373(9)	0.0272(8)	-0.0142(7)	-0.0032(6)	-0.0020(6)
C19	0.0222(8)	0.0313(8)	0.0251(8)	-0.0094(6)	-0.0064(6)	0.0025(6)
C20	0.0204(7)	0.0248(7)	0.0212(7)	-0.0056(6)	-0.0036(6)	0.0012(6)
C21	0.0198(7)	0.0194(7)	0.0158(7)	-0.0032(5)	0.0015(5)	-0.0001(5)
C22	0.0203(7)	0.0203(7)	0.0200(7)	-0.0043(6)	0.0012(6)	-0.0006(5)
C23	0.0224(7)	0.0240(7)	0.0266(8)	-0.0045(6)	0.0053(6)	-0.0026(6)
C24	0.0265(8)	0.0286(8)	0.0243(8)	-0.0045(6)	0.0082(6)	-0.0016(6)
C25	0.0278(8)	0.0288(8)	0.0159(7)	-0.0025(6)	0.0036(6)	0.0020(6)
C26	0.0222(7)	0.0264(7)	0.0154(7)	-0.0055(6)	0.0015(6)	-0.0013(6)
C27	0.0260(8)	0.0182(7)	0.0162(7)	-0.0018(5)	0.0036(6)	-0.0048(6)
C28	0.0262(8)	0.0245(7)	0.0218(8)	-0.0087(6)	0.0032(6)	-0.0037(6)
C29	0.0436(10)	0.0248(8)	0.0294(9)	-0.0088(7)	0.0117(7)	-0.0087(7)
C30	0.0554(12)	0.0247(8)	0.0412(11)	-0.0140(8)	0.0127(9)	-0.0070(8)
C31	0.0472(11)	0.0340(9)	0.0363(10)	-0.0202(8)	0.0093(8)	-0.0025(8)
C32	0.0349(9)	0.0385(9)	0.0227(8)	-0.0102(7)	0.0060(7)	-0.0021(7)
C33	0.0275(8)	0.0259(8)	0.0225(8)	-0.0060(6)	0.0027(6)	-0.0016(6)
C34	0.0188(7)	0.0268(8)	0.0268(8)	-0.0091(6)	0.0017(6)	-0.0010(6)
C35	0.0232(9)	0.0337(9)	0.0688(14)	-0.0014(9)	0.0019(8)	-0.0072(7)
C36	0.0250(9)	0.0815(15)	0.0373(11)	-0.0305(10)	-0.0033(8)	0.0003(9)
C37	0.0308(9)	0.0358(9)	0.0466(11)	-0.0166(8)	-0.0052(8)	0.0089(7)
C38	0.0245(8)	0.0351(8)	0.0191(8)	-0.0096(6)	-0.0019(6)	-0.0054(6)
C39	0.0380(10)	0.0421(10)	0.0277(9)	-0.0130(7)	-0.0030(7)	-0.0121(8)
C40	0.0289(8)	0.0389(9)	0.0226(8)	-0.0060(7)	-0.0026(6)	-0.0021(7)
C41	0.0237(8)	0.0539(11)	0.0260(9)	-0.0137(8)	-0.0019(7)	-0.0029(7)
C42	0.0253(8)	0.0218(7)	0.0200(7)	-0.0049(6)	-0.0003(6)	-0.0023(6)
C43	0.0240(8)	0.0270(8)	0.0213(8)	-0.0059(6)	-0.0001(6)	-0.0054(6)
C44	0.0306(8)	0.0305(8)	0.0238(8)	-0.0069(7)	0.0021(6)	-0.0039(7)
C45	0.0303(9)	0.0435(10)	0.0253(9)	-0.0125(7)	0.0040(7)	-0.0055(7)
C46	0.0336(9)	0.0495(10)	0.0192(8)	-0.0052(7)	0.0023(7)	-0.0131(8)
C47	0.0436(10)	0.0337(9)	0.0229(8)	0.0004(7)	-0.0004(7)	-0.0133(7)
C48	0.0372(9)	0.0282(8)	0.0246(8)	-0.0064(7)	0.0008(7)	-0.0075(7)
C49	0.0485(10)	0.0197(7)	0.0162(7)	-0.0016(6)	0.0048(7)	-0.0118(7)
C50	0.0497(11)	0.0305(9)	0.0444(11)	-0.0147(8)	0.0219(9)	-0.0135(8)
C51	0.0991(19)	0.0309(10)	0.0549(14)	-0.0191(10)	0.0516(14)	-0.0213(11)
C52	0.160(3)	0.0276(10)	0.0209(10)	-0.0093(8)	0.0245(13)	-0.0373(15)
C53	0.139(3)	0.0334(11)	0.0268(10)	0.0007(9)	-0.0271(13)	-0.0352(14)
C54	0.0747(14)	0.0268(9)	0.0319(10)	-0.0009(7)	-0.0173(9)	-0.0119(9)
C55A	0.060(6)	0.052(5)	0.074(4)	-0.035(3)	-0.030(4)	0.020(3)
C56A	0.045(3)	0.066(4)	0.041(3)	-0.028(2)	-0.0022(19)	0.003(2)
C57A	0.055(3)	0.060(4)	0.049(3)	-0.007(2)	-0.021(2)	0.008(2)
C58A	0.054(3)	0.080(4)	0.039(2)	0.015(2)	-0.0145(19)	-0.005(2)
C59A	0.046(4)	0.145(10)	0.026(2)	-0.002(4)	-0.006(3)	0.022(5)
C60A	0.081(4)	0.100(6)	0.044(3)	-0.044(3)	-0.026(3)	0.044(4)

C61A	0.087(5)	0.066(3)	0.172(7)	-0.052(4)	-0.046(5)	0.003(3)
C55B	0.093(7)	0.043(4)	0.034(4)	-0.009(3)	-0.019(4)	0.003(4)
C56B	0.057(6)	0.073(7)	0.038(4)	0.016(4)	-0.007(5)	0.007(5)
C57B	0.076(6)	0.106(8)	0.039(4)	0.012(5)	-0.001(4)	0.035(6)
C58B	0.090(6)	0.067(5)	0.054(4)	-0.009(4)	-0.011(4)	0.017(4)
C59B	0.077(6)	0.057(5)	0.086(5)	-0.019(4)	-0.025(4)	0.000(4)
C60B	0.048(7)	0.064(9)	0.075(6)	-0.032(6)	-0.019(5)	0.010(5)
C61B	0.128(9)	0.063(5)	0.039(4)	-0.013(3)	-0.013(5)	-0.004(5)

6.10 Crystal structure determination of Silazine 9_{CN}

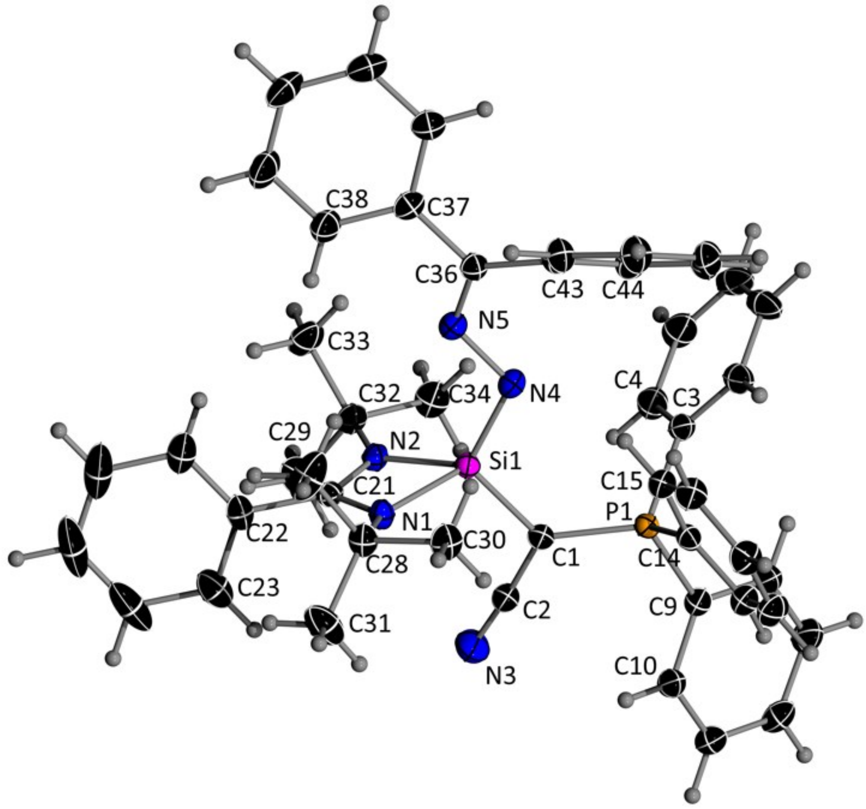

Figure S84. ORTEP of **9_{CN}**. Ellipsoids are drawn at the 50% probability level.

Table S22. Atomic coordinates (x 10^4) and equivalent isotropic displacement parameters (Å^{2}x 10^3) for **9_{CN}**. U(eq) is defined as one third of the trace of the orthogonalized U^{ij} tensor for all atoms.

Atom	X	Y	Z	U(eq)
P1	0.56381(2)	0.36639(4)	0.38945(2)	0.01781(9)
Si1	0.53994(2)	0.31006(4)	0.25525(2)	0.01808(9)
N1	0.52823(6)	0.13503(12)	0.21167(5)	0.0201(2)
N2	0.59694(6)	0.31585(12)	0.20828(5)	0.0196(2)
N3	0.70028(7)	0.15201(17)	0.34300(6)	0.0376(3)
N4	0.47453(6)	0.42701(13)	0.24638(5)	0.0221(2)
N5	0.44783(6)	0.45954(13)	0.18800(5)	0.0209(2)
C4	0.60628(8)	0.65225(17)	0.37042(7)	0.0298(3)
C5	0.60357(9)	0.80928(17)	0.36767(7)	0.0362(4)
C1	0.58633(7)	0.28537(14)	0.33109(6)	0.0194(3)

C2	0.64917(7)	0.21311(15)	0.33951(6)	0.0229(3)
C3	0.55355(7)	0.57010(15)	0.38295(6)	0.0223(3)
C6	0.54783(9)	0.88387(17)	0.37651(7)	0.0372(4)
C7	0.49535(9)	0.80404(17)	0.38915(7)	0.0349(4)
C8	0.49826(8)	0.64596(16)	0.39301(6)	0.0271(3)
C9	0.62904(7)	0.33773(15)	0.45679(6)	0.0211(3)
C10	0.64464(8)	0.45467(16)	0.49787(6)	0.0273(3)
C11	0.69431(8)	0.43383(18)	0.54951(7)	0.0331(3)
C12	0.72920(8)	0.29784(18)	0.56053(6)	0.0311(3)
C13	0.71270(8)	0.18001(17)	0.52085(6)	0.0280(3)
C14	0.66254(7)	0.19910(16)	0.46928(6)	0.0245(3)
C15	0.48617(7)	0.29050(14)	0.40119(6)	0.0205(3)
C16	0.42629(7)	0.30267(16)	0.35665(6)	0.0236(3)
C17	0.36692(7)	0.23964(17)	0.36382(7)	0.0282(3)
C18	0.36706(8)	0.16292(17)	0.41477(7)	0.0294(3)
C19	0.42598(8)	0.15197(18)	0.45918(7)	0.0303(3)
C20	0.48567(7)	0.21610(16)	0.45281(6)	0.0255(3)
C21	0.57365(7)	0.18337(15)	0.18401(6)	0.0192(3)
C22	0.59593(7)	0.10217(16)	0.13751(6)	0.0247(3)
C23	0.64620(8)	-0.00738(18)	0.15356(8)	0.0352(4)
C24	0.66919(10)	-0.0812(2)	0.11058(11)	0.0533(6)
C25	0.64178(12)	-0.0460(2)	0.05251(11)	0.0605(6)
C26	0.59183(12)	0.0621(2)	0.03674(8)	0.0526(5)
C27	0.56844(9)	0.13706(19)	0.07897(7)	0.0343(4)
C28	0.47664(7)	0.01322(16)	0.19508(6)	0.0241(3)
C29	0.43172(9)	0.0400(2)	0.13356(7)	0.0433(4)
C30	0.43267(8)	0.02668(17)	0.23755(7)	0.0291(3)
C31	0.51010(9)	-0.14210(18)	0.20080(9)	0.0410(4)
C32	0.64549(7)	0.42528(16)	0.19444(6)	0.0237(3)
C33	0.61225(9)	0.50319(18)	0.13685(7)	0.0355(4)
C34	0.65896(8)	0.54283(17)	0.24298(7)	0.0323(3)
C35	0.71148(8)	0.34851(19)	0.19323(9)	0.0388(4)
C36	0.38819(7)	0.52121(15)	0.17196(6)	0.0204(3)
C37	0.36261(7)	0.55676(16)	0.10908(6)	0.0227(3)
C38	0.38740(8)	0.47806(19)	0.06778(7)	0.0309(3)
C39	0.36427(9)	0.5104(2)	0.00894(7)	0.0398(4)
C40	0.31618(9)	0.6233(2)	-0.01049(7)	0.0388(4)
C41	0.29191(8)	0.70361(18)	0.02981(7)	0.0332(3)
C42	0.31433(7)	0.67026(16)	0.08890(6)	0.0264(3)
C43	0.34429(7)	0.54889(15)	0.21182(6)	0.0209(3)
C44	0.27917(7)	0.48833(17)	0.19834(6)	0.0260(3)
C45	0.23816(7)	0.50427(18)	0.23604(7)	0.0303(3)
C46	0.26159(8)	0.58198(18)	0.28779(7)	0.0296(3)
C47	0.32628(8)	0.64346(17)	0.30188(6)	0.0283(3)
C48	0.36762(7)	0.62673(15)	0.26437(6)	0.0238(3)

Table S23. Anisotropic displacement parameters(Å^2) for **9$_{CN}$**. The anisotropic displacement factor exponent takes the form: $-2\pi^2[h^2a^{*2}U^{11} + ...+ 2 h k a^* b^* U^{12}]$.

atom	U^{11}	U^{22}	U^{33}	U^{23}	U^{13}	U^{12}
P1	0.02107(17)	0.01520(16)	0.01680(16)	-0.00034(12)	0.00437(13)	0.00016(12)
Si1	0.01945(18)	0.01830(18)	0.01680(18)	-0.00055(13)	0.00533(14)	-0.00032(13)
N1	0.0212(5)	0.0206(6)	0.0197(6)	-0.0017(4)	0.0074(4)	-0.0025(4)
N2	0.0213(6)	0.0193(5)	0.0193(5)	0.0003(4)	0.0074(4)	-0.0022(4)
N3	0.0334(7)	0.0453(8)	0.0346(7)	0.0019(6)	0.0099(6)	0.0152(6)
N4	0.0242(6)	0.0236(6)	0.0180(5)	0.0006(4)	0.0044(4)	0.0009(5)
N5	0.0220(6)	0.0216(5)	0.0186(5)	0.0009(4)	0.0046(4)	-0.0016(4)
C4	0.0406(9)	0.0226(7)	0.0276(7)	-0.0017(6)	0.0112(6)	-0.0046(6)
C5	0.0552(10)	0.0236(8)	0.0297(8)	-0.0007(6)	0.0109(7)	-0.0098(7)
C1	0.0211(6)	0.0182(6)	0.0191(6)	0.0001(5)	0.0054(5)	0.0004(5)
C2	0.0262(7)	0.0231(7)	0.0195(7)	0.0007(5)	0.0063(5)	0.0010(6)
C3	0.0304(7)	0.0173(6)	0.0169(6)	-0.0014(5)	0.0022(5)	-0.0002(5)
C6	0.0591(11)	0.0169(7)	0.0266(8)	-0.0012(6)	-0.0045(7)	-0.0011(7)
C7	0.0411(9)	0.0238(7)	0.0314(8)	-0.0082(6)	-0.0053(7)	0.0084(7)
C8	0.0302(8)	0.0234(7)	0.0237(7)	-0.0040(6)	0.0001(6)	0.0029(6)
C9	0.0231(7)	0.0220(6)	0.0183(6)	-0.0002(5)	0.0056(5)	-0.0023(5)
C10	0.0359(8)	0.0215(7)	0.0227(7)	-0.0018(6)	0.0046(6)	0.0015(6)
C11	0.0450(9)	0.0290(8)	0.0211(7)	-0.0044(6)	0.0013(6)	-0.0032(7)
C12	0.0325(8)	0.0368(8)	0.0203(7)	0.0035(6)	0.0005(6)	-0.0016(6)
C13	0.0304(8)	0.0293(7)	0.0236(7)	0.0047(6)	0.0055(6)	0.0039(6)
C14	0.0289(7)	0.0218(7)	0.0218(7)	-0.0002(5)	0.0053(6)	0.0001(6)
C15	0.0247(7)	0.0168(6)	0.0214(7)	-0.0023(5)	0.0085(5)	0.0010(5)
C16	0.0257(7)	0.0241(7)	0.0217(7)	0.0004(5)	0.0076(6)	0.0006(6)
C17	0.0238(7)	0.0324(8)	0.0283(8)	-0.0004(6)	0.0065(6)	-0.0001(6)
C18	0.0262(7)	0.0308(8)	0.0349(8)	0.0012(6)	0.0144(6)	-0.0031(6)
C19	0.0331(8)	0.0331(8)	0.0278(8)	0.0064(6)	0.0136(6)	0.0001(6)
C20	0.0275(7)	0.0268(7)	0.0229(7)	0.0018(6)	0.0078(6)	0.0003(6)
C21	0.0186(6)	0.0202(6)	0.0180(6)	0.0019(5)	0.0034(5)	0.0015(5)
C22	0.0263(7)	0.0229(7)	0.0288(7)	-0.0057(6)	0.0143(6)	-0.0067(6)
C23	0.0319(8)	0.0268(8)	0.0520(10)	-0.0045(7)	0.0202(7)	-0.0024(6)
C24	0.0499(11)	0.0298(9)	0.0965(17)	-0.0188(10)	0.0484(11)	-0.0057(8)
C25	0.0787(15)	0.0519(12)	0.0729(15)	-0.0324(11)	0.0589(13)	-0.0234(11)
C26	0.0730(13)	0.0577(12)	0.0377(10)	-0.0208(9)	0.0331(10)	-0.0257(11)
C27	0.0430(9)	0.0369(9)	0.0265(8)	-0.0071(6)	0.0153(7)	-0.0115(7)
C28	0.0257(7)	0.0228(7)	0.0254(7)	-0.0041(5)	0.0095(6)	-0.0078(6)
C29	0.0391(9)	0.0607(11)	0.0276(8)	-0.0037(8)	0.0045(7)	-0.0244(8)
C30	0.0281(7)	0.0299(8)	0.0325(8)	-0.0017(6)	0.0137(6)	-0.0071(6)
C31	0.0447(10)	0.0228(8)	0.0648(12)	-0.0052(7)	0.0309(9)	-0.0059(7)
C32	0.0257(7)	0.0220(7)	0.0257(7)	0.0025(6)	0.0105(6)	-0.0055(6)
C33	0.0508(10)	0.0283(8)	0.0274(8)	0.0058(6)	0.0103(7)	-0.0084(7)
C34	0.0376(8)	0.0282(8)	0.0319(8)	-0.0032(6)	0.0106(7)	-0.0132(6)
C35	0.0282(8)	0.0335(8)	0.0598(11)	0.0000(8)	0.0205(8)	-0.0049(7)
C36	0.0207(6)	0.0203(6)	0.0208(7)	0.0003(5)	0.0065(5)	-0.0011(5)
C37	0.0205(6)	0.0251(7)	0.0223(7)	0.0033(5)	0.0053(5)	-0.0040(5)

C38	0.0299(8)	0.0393(8)	0.0255(8)	0.0053(6)	0.0109(6)	0.0059(7)
C39	0.0420(9)	0.0562(11)	0.0255(8)	0.0078(7)	0.0167(7)	0.0072(8)
C40	0.0383(9)	0.0546(11)	0.0243(8)	0.0146(7)	0.0095(7)	0.0004(8)
C41	0.0298(8)	0.0343(8)	0.0336(8)	0.0137(7)	0.0050(6)	0.0012(6)
C42	0.0244(7)	0.0254(7)	0.0288(8)	0.0029(6)	0.0058(6)	-0.0026(6)
C43	0.0220(6)	0.0191(6)	0.0219(7)	0.0022(5)	0.0063(5)	0.0028(5)
C44	0.0247(7)	0.0286(7)	0.0251(7)	-0.0051(6)	0.0071(6)	-0.0013(6)
C45	0.0248(7)	0.0350(8)	0.0330(8)	-0.0030(6)	0.0110(6)	-0.0024(6)
C46	0.0295(7)	0.0351(8)	0.0277(8)	-0.0016(6)	0.0135(6)	0.0053(6)
C47	0.0312(8)	0.0296(8)	0.0241(7)	-0.0045(6)	0.0071(6)	0.0044(6)
C48	0.0234(7)	0.0226(7)	0.0247(7)	-0.0009(5)	0.0050(6)	0.0011(5)

6.11 Crystal structure determination of 10

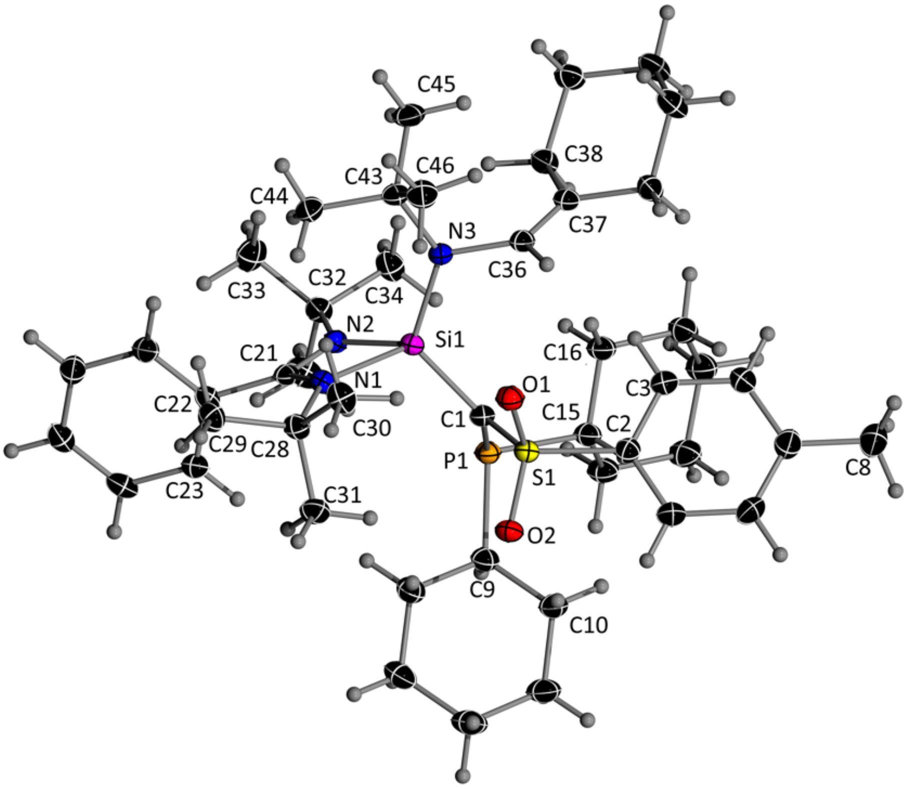

Figure S85. ORTEP of **10**. Ellipsoids are drawn at the 50% probability level.

Table S24. Atomic coordinates (x 10^4) and equivalent isotropic displacement parameters (Å^{2}x 10^3) for **10**. U(eq) is defined as one third of the trace of the orthogonalized U^{ij} tensor for all atoms.

Atom	X	Y	Z	U(eq)
S1	0.23568(3)	0.66812(2)	0.19612(2)	0.01822(9)
P1	0.42169(3)	0.58382(2)	0.21623(2)	0.01960(9)
Si1	0.42574(3)	0.66932(2)	0.36008(2)	0.01628(9)
O1	0.20710(9)	0.70555(3)	0.26089(7)	0.0227(2)
N1	0.51412(10)	0.72414(4)	0.33722(8)	0.0180(2)
C1	0.35322(12)	0.63697(5)	0.25617(9)	0.0184(3)
O2	0.25105(8)	0.68600(4)	0.10397(6)	0.0222(2)
N2	0.58335(10)	0.65437(4)	0.38925(8)	0.0182(2)

N3	0.36137(10)	0.67170(4)	0.45902(8)	0.0183(2)
C2	0.10674(12)	0.63033(5)	0.16968(9)	0.0202(3)
C3	0.06984(12)	0.60450(5)	0.24192(10)	0.0226(3)
C4	-0.03292(13)	0.57697(5)	0.22345(10)	0.0228(3)
C5	-0.10197(13)	0.57466(5)	0.13318(11)	0.0270(3)
C6	-0.06516(14)	0.60155(6)	0.06199(11)	0.0331(4)
C7	0.03860(13)	0.62908(6)	0.07931(10)	0.0268(3)
C8	-0.21240(15)	0.54355(7)	0.11417(12)	0.0381(4)
C9	0.31060(13)	0.53386(5)	0.21195(9)	0.0218(3)
C10	0.29129(13)	0.52287(5)	0.31251(10)	0.0241(3)
C11	0.19820(14)	0.48328(5)	0.31279(10)	0.0272(3)
C12	0.23394(15)	0.43675(5)	0.26744(11)	0.0300(3)
C13	0.25883(15)	0.44685(5)	0.16857(11)	0.0299(3)
C14	0.35157(14)	0.48697(5)	0.16960(10)	0.0261(3)
C15	0.43314(13)	0.58798(5)	0.08870(10)	0.0233(3)
C16	0.32058(13)	0.58506(5)	0.01150(10)	0.0247(3)
C17	0.35422(15)	0.58031(6)	-0.08632(10)	0.0311(3)
C18	0.43138(16)	0.62287(6)	-0.10601(11)	0.0339(4)
C19	0.54150(14)	0.62842(6)	-0.02807(11)	0.0308(3)
C20	0.50846(13)	0.63170(5)	0.07036(10)	0.0262(3)
C21	0.61375(12)	0.69803(5)	0.36007(9)	0.0183(3)
C22	0.73798(12)	0.71241(5)	0.35536(9)	0.0194(3)
C23	0.78793(13)	0.69716(5)	0.27916(10)	0.0220(3)
C24	0.90656(13)	0.70730(5)	0.27777(10)	0.0245(3)
C25	0.97505(13)	0.73261(5)	0.35153(11)	0.0254(3)
C26	0.92497(13)	0.74793(5)	0.42709(10)	0.0249(3)
C27	0.80637(12)	0.73821(5)	0.42952(10)	0.0220(3)
C28	0.49868(12)	0.77171(5)	0.28568(10)	0.0211(3)
C29	0.38375(13)	0.79489(5)	0.30440(11)	0.0268(3)
C30	0.48982(14)	0.76232(5)	0.17979(10)	0.0270(3)
C31	0.60124(13)	0.80710(5)	0.32006(11)	0.0255(3)
C32	0.66283(12)	0.61499(5)	0.43692(10)	0.0212(3)
C33	0.75082(14)	0.63437(6)	0.52203(11)	0.0320(3)
C34	0.73056(14)	0.59005(6)	0.36827(12)	0.0317(3)
C35	0.58119(13)	0.57829(5)	0.47133(11)	0.0280(3)
C36	0.24439(12)	0.64993(5)	0.45161(9)	0.0195(3)
C37	0.21706(12)	0.60874(5)	0.49180(9)	0.0208(3)
C38	0.30165(13)	0.57313(5)	0.54868(10)	0.0234(3)
C39	0.26582(13)	0.55946(6)	0.64319(10)	0.0273(3)
C40	0.13594(14)	0.54376(6)	0.62983(11)	0.0302(3)
C41	0.05430(14)	0.58242(6)	0.57695(11)	0.0308(3)
C42	0.08783(12)	0.59370(6)	0.48095(10)	0.0253(3)
C43	0.38507(12)	0.70777(5)	0.54063(9)	0.0214(3)
C44	0.28485(14)	0.74562(6)	0.52815(11)	0.0279(3)
C45	0.50453(13)	0.73351(5)	0.54675(10)	0.0253(3)
C46	0.38922(15)	0.68078(6)	0.63430(10)	0.0297(3)

Table S25. Anisotropic displacement parameters(Å^2) for **9**. The anisotropic displacement factor exponent takes the form: $-2\pi^2[h^2a^{*2}U^{11} + ...+ 2 h k a^* b^* U^{12}]$.

atom	U11	U22	U33	U23	U13	U12
S1	0.01967(16)	0.01988(16)	0.01530(16)	0.00062(12)	0.00377(12)	-0.00120(12)
P1	0.02307(18)	0.02075(18)	0.01570(17)	-0.00115(12)	0.00545(13)	-0.00038(13)
Si1	0.01720(18)	0.01772(18)	0.01446(17)	0.00012(13)	0.00434(13)	-0.00009(13)
O1	0.0270(5)	0.0222(5)	0.0198(5)	-0.0016(4)	0.0065(4)	-0.0003(4)
N1	0.0190(5)	0.0182(5)	0.0174(5)	0.0010(4)	0.0052(4)	-0.0001(4)
C1	0.0201(6)	0.0205(6)	0.0148(6)	-0.0006(5)	0.0040(5)	-0.0007(5)
O2	0.0250(5)	0.0253(5)	0.0170(5)	0.0039(4)	0.0055(4)	-0.0010(4)
N2	0.0176(5)	0.0197(6)	0.0177(5)	0.0012(4)	0.0042(4)	0.0006(4)
N3	0.0190(5)	0.0215(6)	0.0150(5)	-0.0021(4)	0.0045(4)	-0.0025(4)
C2	0.0195(6)	0.0212(7)	0.0199(7)	-0.0012(5)	0.0037(5)	0.0003(5)
C3	0.0240(7)	0.0255(7)	0.0179(7)	0.0001(5)	0.0032(5)	-0.0006(6)
C4	0.0239(7)	0.0226(7)	0.0230(7)	0.0012(5)	0.0074(6)	-0.0008(6)
C5	0.0251(7)	0.0271(7)	0.0277(7)	-0.0018(6)	0.0021(6)	-0.0032(6)
C6	0.0331(8)	0.0418(9)	0.0210(7)	0.0007(6)	-0.0038(6)	-0.0087(7)
C7	0.0292(8)	0.0319(8)	0.0189(7)	0.0028(6)	0.0032(6)	-0.0040(6)
C8	0.0344(9)	0.0430(10)	0.0341(9)	0.0005(7)	-0.0005(7)	-0.0132(7)
C9	0.0277(7)	0.0212(7)	0.0174(6)	-0.0006(5)	0.0069(5)	-0.0016(6)
C10	0.0323(8)	0.0229(7)	0.0182(7)	-0.0011(5)	0.0075(6)	-0.0023(6)
C11	0.0344(8)	0.0251(7)	0.0238(7)	0.0003(6)	0.0098(6)	-0.0037(6)
C12	0.0409(9)	0.0231(7)	0.0272(8)	-0.0005(6)	0.0092(7)	-0.0056(6)
C13	0.0424(9)	0.0236(7)	0.0248(7)	-0.0052(6)	0.0093(7)	-0.0038(6)
C14	0.0350(8)	0.0235(7)	0.0213(7)	-0.0028(6)	0.0090(6)	-0.0001(6)
C15	0.0265(7)	0.0256(7)	0.0195(7)	-0.0021(5)	0.0086(6)	-0.0010(6)
C16	0.0304(8)	0.0269(7)	0.0175(7)	-0.0018(5)	0.0063(6)	-0.0027(6)
C17	0.0442(9)	0.0323(8)	0.0180(7)	-0.0024(6)	0.0086(6)	-0.0031(7)
C18	0.0485(10)	0.0345(9)	0.0218(7)	0.0011(6)	0.0140(7)	-0.0032(7)
C19	0.0362(8)	0.0313(8)	0.0293(8)	0.0015(6)	0.0178(7)	-0.0020(7)
C20	0.0284(8)	0.0275(8)	0.0247(7)	-0.0016(6)	0.0102(6)	-0.0022(6)
C21	0.0214(7)	0.0206(7)	0.0135(6)	-0.0019(5)	0.0049(5)	0.0000(5)
C22	0.0198(7)	0.0193(6)	0.0196(6)	0.0030(5)	0.0055(5)	0.0009(5)
C23	0.0260(7)	0.0218(7)	0.0189(7)	0.0009(5)	0.0062(5)	0.0002(5)
C24	0.0264(7)	0.0260(7)	0.0240(7)	0.0064(6)	0.0123(6)	0.0047(6)
C25	0.0196(7)	0.0268(7)	0.0306(8)	0.0090(6)	0.0065(6)	0.0015(6)
C26	0.0230(7)	0.0245(7)	0.0257(7)	0.0028(6)	0.0007(6)	-0.0031(6)
C27	0.0242(7)	0.0225(7)	0.0199(7)	0.0002(5)	0.0056(5)	-0.0003(5)
C28	0.0244(7)	0.0179(6)	0.0212(7)	0.0027(5)	0.0045(5)	-0.0001(5)
C29	0.0264(7)	0.0204(7)	0.0341(8)	0.0034(6)	0.0069(6)	0.0024(6)
C30	0.0336(8)	0.0258(7)	0.0213(7)	0.0047(6)	0.0042(6)	-0.0032(6)
C31	0.0279(7)	0.0207(7)	0.0281(7)	0.0018(6)	0.0055(6)	-0.0027(6)
C32	0.0194(7)	0.0215(7)	0.0228(7)	0.0046(5)	0.0040(5)	0.0031(5)
C33	0.0306(8)	0.0291(8)	0.0318(8)	0.0056(6)	-0.0054(6)	0.0013(6)
C34	0.0322(8)	0.0293(8)	0.0366(9)	0.0064(7)	0.0146(7)	0.0112(6)
C35	0.0234(7)	0.0273(8)	0.0328(8)	0.0106(6)	0.0039(6)	0.0011(6)
C36	0.0189(6)	0.0250(7)	0.0149(6)	-0.0012(5)	0.0042(5)	0.0005(5)

C37	0.0219(7)	0.0258(7)	0.0155(6)	-0.0017(5)	0.0060(5)	-0.0001(5)
C38	0.0235(7)	0.0258(7)	0.0221(7)	0.0024(6)	0.0075(6)	0.0008(6)
C39	0.0299(8)	0.0304(8)	0.0222(7)	0.0066(6)	0.0063(6)	0.0001(6)
C40	0.0317(8)	0.0316(8)	0.0297(8)	0.0100(6)	0.0121(6)	0.0002(6)
C41	0.0261(8)	0.0360(9)	0.0337(8)	0.0097(7)	0.0139(6)	0.0018(6)
C42	0.0216(7)	0.0305(8)	0.0242(7)	0.0048(6)	0.0055(6)	-0.0025(6)
C43	0.0253(7)	0.0240(7)	0.0160(6)	-0.0042(5)	0.0062(5)	-0.0013(6)
C44	0.0296(8)	0.0278(8)	0.0276(8)	-0.0061(6)	0.0090(6)	0.0011(6)
C45	0.0265(7)	0.0288(8)	0.0209(7)	-0.0072(6)	0.0052(6)	-0.0038(6)
C46	0.0423(9)	0.0306(8)	0.0165(7)	-0.0040(6)	0.0063(6)	-0.0060(7)

7 Computational Details

7.1 General remarks

All calculations were performed without symmetry restrictions. Starting coordinates were obtained with GaussView 6.0[40] or directly from the crystal structure analyses if accessible. The geometry optimizations were carried out with the Gaussian16 (Revision C.01)[41] program package. The geometry optimizations were performed using Density Functional Theory (DFT)[31] with the PBE0[32] functional using the def2svp[42] in conjunction with Grimme's D3 dispersion correction[43] with Becke-Johnson damping[44]. Harmonic vibrational frequency analyses were performed at the same level of theory as the optimizations to determine the nature of the structure. The vibrational frequency analysis showed no imaginary frequencies. Single point energies were calculated using the def2tzvpp[42] basis set. The NBO analysis was performed with NBO Version 7.0[45] using the def2tzvpp basis set. The optimized structures were used for the quantum theory of atoms in molecules (QTAIM)[46] and noncovalent interaction (NCI)[47] analyses using Multiwfn[48] to depict the topological properties of the molecules. The results were visualized using the VMD 1.9.4a51 software.[49]

7.2 Hydrogen Bonding in Silanone 6

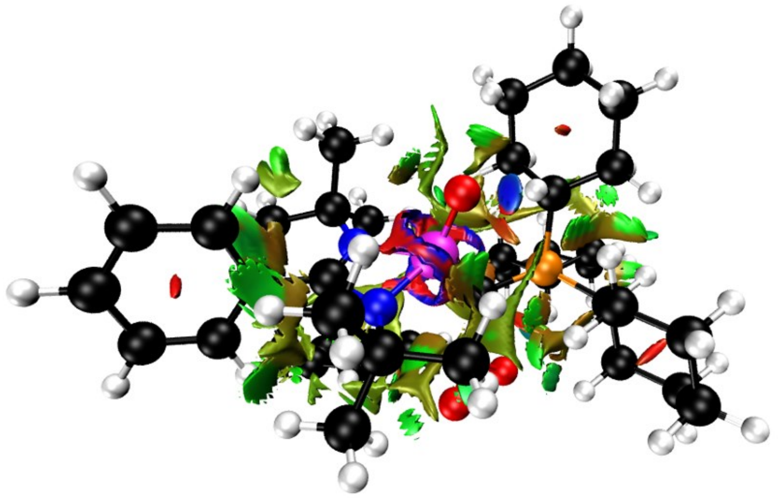

Figure S86. NCI plot for the silanone **6** coloured in a blue-green-red scheme over the range of -0.035 < sign(λ2)ρ < 0.02 and isosurface of RDG = 0.3. Blue indicates strong attraction, green indicates weak interaction, and red indicates repulsion.

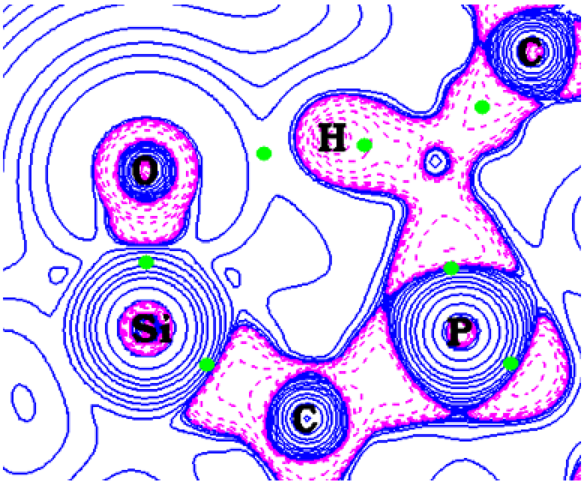

Figure S87. Contour plot of the Laplacian of the electron density around the Si-O bond in silanone 6 to showcase the bond critical point (BCP) between the oxygen atom and the hydrogen atom. Electron density ρ(r) = 0.035 au, Laplacian of the electron density $\nabla^2\rho(r)$ = 0.111 au, Kinetic energy density G(r) = 0.027 au, Potential energy density V(r) = -0.026, Total electronic energy density H(r) = 0.0006 au, and bond ellipticity $\varepsilon(r_c)$ = 0.047.

7.3 Results of the NBO analysis

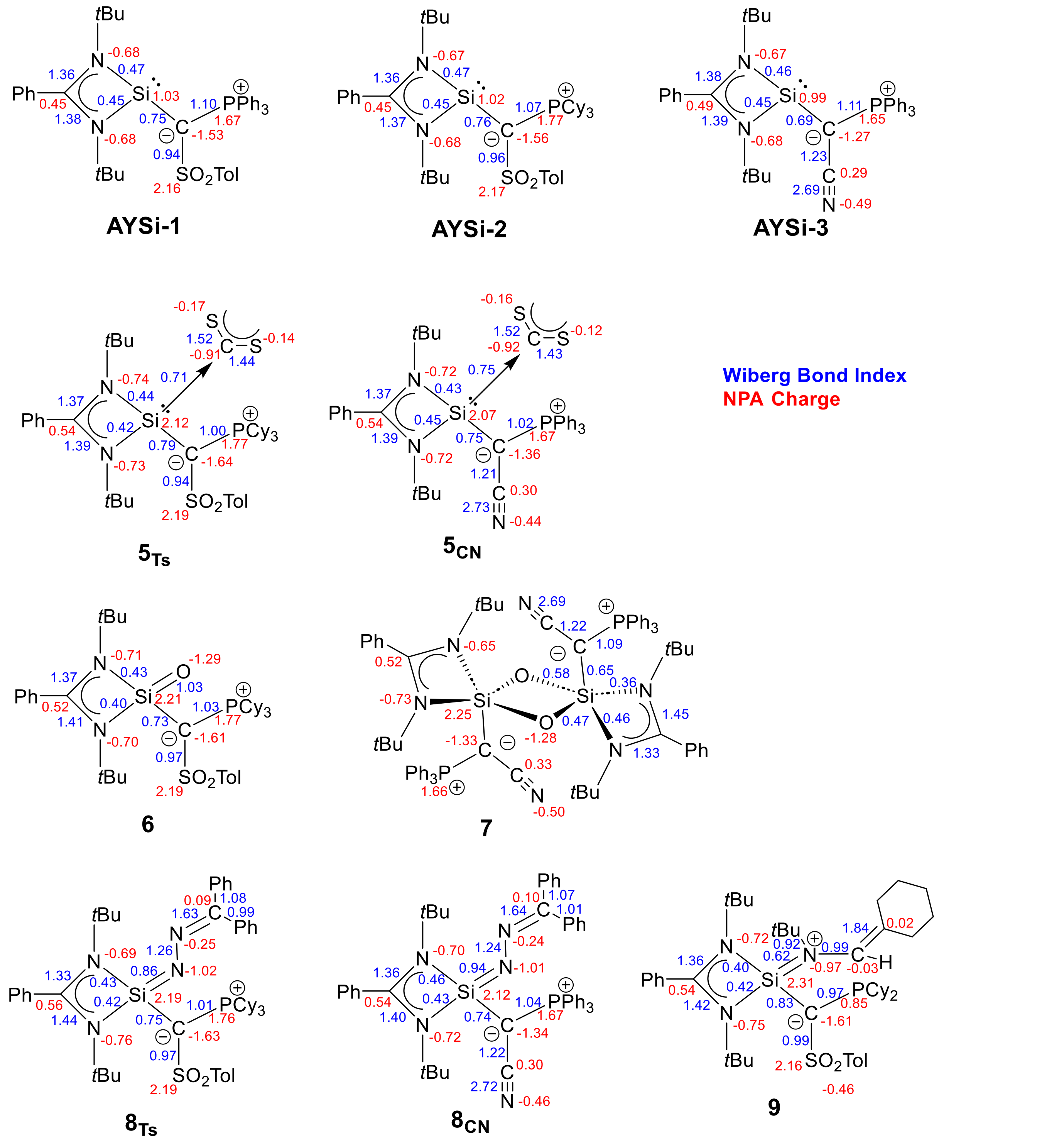

Figure S88. Wiberg Bond Indeces (blue) and NPA charges (red) for **AYSi-1**, **AYSI-2**, **AYSi-3**, **4-9** calculated at the PBE0/def2tzvpp level of theory.

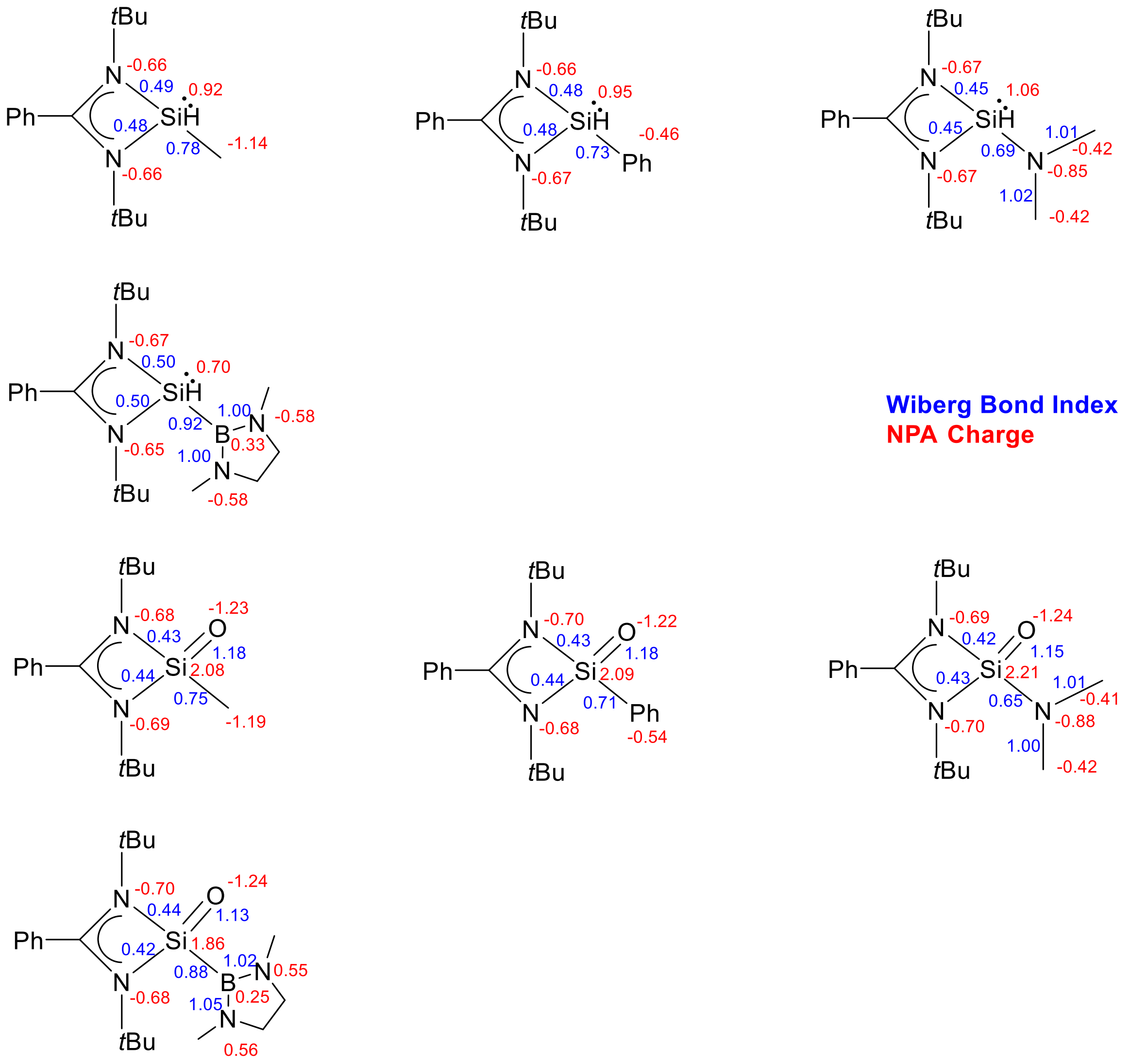

Figure S89. Wiberg Bond Indeces (blue) and NPA charges (red) for amidine-substituted silylenes and their respective silanones calculated at the PBE0/def2tzvpp level of theory.

Table S26. Second order perturbation theory analysis of Fock Matrix in NBO Basis of Silanone 6

Donor (L) NBO	Acceptor (NL) NBO	*E*(2) (kcal/mol)	E(NL)-E(L) (a.u.)	F(L,NL) (a.u.)
from unit 3 to unit 1				
LP (1) O112	BD*(1) C 9- H 19	2.99	0.90	0.046
LP (2) O112	BD*(1) C 9- H 19	6.76	0.64	0.059
LP (3) O112	BD*(1) C 9- H 19	2.47	0.64	0.035

Table S27. Second order perturbation theory analysis of Fock Matrix in NBO Basis of AYSi-2

Donor (L) NBO	Acceptor (NL) NBO	*E*(2) (kcal/mol)	E(NL)-E(L) (a.u.)	F(L,NL) (a.u.)
within unit 1				
BD (1) C 3-Si 7	BD*(1) S 1- O 2	2.97	0.74	0.042

Table S28. NBO analysis of different amidine-substituted silylenes with substituents R.

**R**	**WBI Si-R**	**HOMO (kcal/mol)**	**LUMO (kcal/mol)**	**DE_{H-L} (kcal/mol)**
Me	0.78	-115.59	-20.77	94.82
Ph	0.73	-120.62	-22.98	97.64
NMe_2	0.69	-121.53	-20.19	101.34
BR_2	0.92	-108.07	-20.44	87.63
PhYCN (AYSi-3)	0.69	-115.17	-28.24	86.93
CyYTos (AYSi-2)	0.76	-111.33	-17.16	93.73
Bis(amidinato)	0.95 (avg.)	-100.41	-21.08	79.33

Table S29. NBO analysis of different amidine-substituted silylanones with substituents R.

**R**	**WBI Si-R**	**WBI Si-O**	**DE_{H-L} (kcal/mol)**
Me	0.75	1.18	119.45
Ph	0.71	1.18	121.53
NMe_2	0.65	1.15	109.07
BR_2	0.88	1.13	100.77
PhYCN (AYSi-3)	0.68	1.09	101.46
CyYTos (AYSi-2)	0.73	1.03	113.22

7.4 Coordinates of the optimized structures

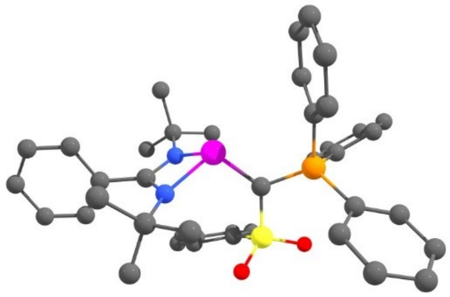

AYSi-1

E = -2876.71922576

S	2.181465000	3.900648000	5.849055000
O	0.842128000	4.511608000	5.673936000
C	3.252165000	4.710083000	4.765808000
O	2.294353000	2.436468000	5.745766000
C	2.642168000	4.265856000	7.540428000
Si	5.099748000	4.500473000	4.505359000
C	1.959448000	5.262434000	8.231882000
C	3.606225000	3.493870000	8.182182000
N	5.551244000	2.827561000	5.273516000
N	5.935469000	4.707522000	6.213249000
C	6.273790000	3.419115000	6.229056000
H	1.148866000	5.795281000	7.731681000
C	2.296456000	5.531211000	9.556779000
C	3.922076000	3.762301000	9.508615000
H	4.081307000	2.679318000	7.633875000
C	5.564987000	1.458248000	4.759326000
C	6.462779000	5.878005000	6.907364000
C	7.379955000	2.810893000	7.014049000
H	1.762276000	6.316123000	10.099201000
C	3.289526000	4.796377000	10.213202000
H	4.675539000	3.155534000	10.019172000
C	4.569753000	1.435054000	3.598463000
C	5.080008000	0.484819000	5.836383000
C	6.953562000	1.061738000	4.249854000
C	5.317394000	6.893070000	6.935082000
C	6.888781000	5.574429000	8.344090000
C	7.643594000	6.463391000	6.123876000
C	7.136121000	2.105637000	8.195679000
C	8.698614000	2.966793000	6.570293000
C	3.678458000	5.100798000	11.629645000
H	4.872083000	2.150211000	2.815928000
H	3.572714000	1.704784000	3.974763000
H	4.528331000	0.430469000	3.152425000
H	5.011972000	-0.532593000	5.421431000
H	4.080929000	0.786901000	6.182874000
H	5.774077000	0.449776000	6.688034000
H	6.907629000	0.080082000	3.753641000
H	7.685007000	0.990094000	5.066909000
H	7.315669000	1.801659000	3.519347000
H	4.959610000	7.098296000	5.914388000
H	5.648187000	7.841299000	7.384627000
H	4.471168000	6.498614000	7.516008000
H	7.783143000	4.939384000	8.391273000
H	6.074939000	5.078641000	8.891045000

H	7.121697000	6.519960000	8.857003000
H	7.347248000	6.665603000	5.083756000
H	8.490437000	5.761768000	6.109709000
H	7.988341000	7.402049000	6.585398000
H	6.111095000	1.980901000	8.547650000
C	8.194632000	1.572752000	8.928377000
C	9.755440000	2.432619000	7.302321000
H	8.889568000	3.503261000	5.638163000
H	4.663182000	5.595972000	11.666089000
H	3.756311000	4.182649000	12.231460000
H	2.952485000	5.767892000	12.114888000
H	7.992602000	1.026138000	9.852403000
C	9.505800000	1.737184000	8.485427000
H	10.780538000	2.558032000	6.945848000
H	10.335168000	1.319798000	9.061135000
P	2.354498000	5.623498000	3.604803000
C	3.476208000	6.129908000	2.262380000
C	3.568963000	5.403831000	1.072956000
C	4.301651000	7.244105000	2.461438000
C	4.489385000	5.781673000	0.096871000
H	2.922298000	4.540787000	0.905077000
C	5.216243000	7.619115000	1.483642000
H	4.226896000	7.819979000	3.386866000
C	5.314654000	6.884896000	0.301738000
H	4.558432000	5.208916000	-0.830739000
H	5.860665000	8.485653000	1.647811000
H	6.035914000	7.177795000	-0.465014000
C	1.692625000	7.224201000	4.170139000
C	1.146378000	8.142311000	3.264083000
C	1.836884000	7.577918000	5.511360000
C	0.713083000	9.387695000	3.711771000
H	1.079846000	7.894314000	2.201586000
C	1.408341000	8.826816000	5.955954000
H	2.309499000	6.873265000	6.195585000
C	0.838017000	9.728530000	5.059263000
H	0.285706000	10.100799000	3.002964000
H	1.526581000	9.096324000	7.008127000
H	0.501437000	10.708125000	5.407246000
C	1.011023000	4.684359000	2.811514000
C	-0.248396000	5.221011000	2.535372000
C	1.281196000	3.347671000	2.495739000
C	-1.218982000	4.433699000	1.920453000
H	-0.488301000	6.244313000	2.826750000
C	0.309098000	2.567189000	1.876640000
H	2.246026000	2.913345000	2.766080000
C	-0.940801000	3.110151000	1.583231000
H	-2.205666000	4.855686000	1.715655000
H	0.525973000	1.523233000	1.639372000
H	-1.707110000	2.494493000	1.106222000

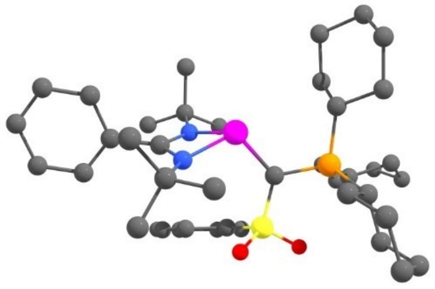

AYSi-2

E = -2887.60483548

S	2.163801000	4.091619000	5.787871000
O	0.826602000	4.718032000	5.630017000
C	3.249970000	4.813451000	4.674153000
O	2.211975000	2.619388000	5.737678000
C	2.649513000	4.503722000	7.460681000
P	2.451801000	5.740004000	3.420843000
Si	5.073723000	4.411116000	4.502157000
C	2.089067000	5.618124000	8.078989000
C	3.511174000	3.666713000	8.162585000
C	3.790996000	6.342023000	2.292083000
C	1.443921000	7.147177000	4.051065000
C	1.321098000	4.784505000	2.304837000
N	5.422839000	2.746045000	5.329058000
N	5.923179000	4.633756000	6.203604000
C	6.178082000	3.327943000	6.264960000
H	1.356823000	6.217949000	7.537101000
C	2.447311000	5.929198000	9.388080000
C	3.850658000	3.980929000	9.473787000
H	3.888236000	2.768451000	7.671964000
H	4.480475000	5.473647000	2.285990000
C	4.590757000	7.500726000	2.892286000
C	3.403207000	6.640816000	0.841482000
H	0.595711000	6.608906000	4.505197000
C	2.111359000	7.930020000	5.181644000
C	0.905264000	8.085039000	2.970784000
H	1.246219000	5.415916000	1.401818000
C	-0.107684000	4.537605000	2.794003000
C	2.002403000	3.467303000	1.928386000
C	5.365211000	1.354560000	4.875060000
C	6.532598000	5.783816000	6.867947000
C	7.240868000	2.678383000	7.075495000
H	2.010627000	6.807785000	9.870638000
C	3.342515000	5.125412000	10.103113000
H	4.523068000	3.322436000	10.031353000
H	3.964796000	8.408172000	2.931546000
H	4.877038000	7.260747000	3.926890000
C	5.836794000	7.781826000	2.062882000
C	4.648302000	6.953746000	0.012581000
H	2.885704000	5.783547000	0.387601000
H	2.704777000	7.494142000	0.795663000
H	2.512050000	7.233822000	5.930836000
H	2.971711000	8.500602000	4.798051000
C	1.110896000	8.897509000	5.802823000
C	-0.084503000	9.072824000	3.585753000
H	1.737305000	8.644359000	2.508800000
H	0.416747000	7.520721000	2.160378000
H	-0.083314000	3.969385000	3.734224000

H	-0.605980000	5.490589000	3.031039000
C	-0.906205000	3.783892000	1.734590000
C	1.196892000	2.696753000	0.889319000
H	3.029992000	3.641120000	1.567588000
H	2.104006000	2.868481000	2.846888000
C	4.407524000	1.336571000	3.685488000
C	4.787541000	0.465286000	5.979000000
C	6.738829000	0.845689000	4.428426000
C	5.442631000	6.854020000	6.951434000
C	7.012948000	5.473263000	8.286698000
C	7.701294000	6.307442000	6.024858000
C	6.948227000	2.030284000	8.278105000
C	8.568281000	2.737672000	6.634552000
C	3.760583000	5.476539000	11.500318000
H	6.399553000	8.619747000	2.505294000
H	6.495560000	6.896478000	2.112356000
C	5.477327000	8.081466000	0.613590000
H	5.269367000	6.042372000	-0.048165000
H	4.351925000	7.198131000	-1.020365000
H	0.293155000	8.319695000	6.270485000
H	1.591540000	9.472090000	6.611265000
C	0.529791000	9.839779000	4.752689000
H	-0.448179000	9.771875000	2.815180000
H	-0.967817000	8.513375000	3.942159000
H	-1.927988000	3.601372000	2.104572000
H	-1.010311000	4.412492000	0.830367000
C	-0.235147000	2.468461000	1.357306000
H	1.186749000	3.261722000	-0.061228000
H	1.692239000	1.736047000	0.674649000
H	4.780159000	1.986330000	2.877586000
H	3.421593000	1.694109000	4.010802000
H	4.310119000	0.314566000	3.290810000
H	4.666237000	-0.563936000	5.607666000
H	3.801051000	0.846690000	6.279657000
H	5.453010000	0.425776000	6.852941000
H	6.636978000	-0.147139000	3.963992000
H	7.438321000	0.750623000	5.270209000
H	7.175606000	1.530424000	3.685002000
H	5.023591000	7.059494000	5.956431000
H	5.850743000	7.791455000	7.358101000
H	4.618780000	6.516182000	7.594575000
H	7.894710000	4.820118000	8.303378000
H	6.211725000	4.999751000	8.870866000
H	7.285809000	6.417037000	8.782720000
H	7.364445000	6.536518000	5.002703000
H	8.503072000	5.557312000	5.958884000
H	8.124361000	7.220266000	6.472643000
H	5.916050000	1.983917000	8.628258000
C	7.968614000	1.457375000	9.034456000
C	9.586616000	2.163514000	7.390022000
H	8.796221000	3.231111000	5.687044000
H	4.781673000	5.893459000	11.508107000
H	3.767992000	4.590887000	12.153370000
H	3.092911000	6.226538000	11.946819000
H	4.898999000	9.022599000	0.569158000
H	6.386823000	8.247898000	0.014008000
H	-0.220221000	10.508426000	5.205220000
H	1.336282000	10.492298000	4.370808000

H	-0.816327000	1.946328000	0.579878000
H	-0.223174000	1.805742000	2.240652000
H	7.729428000	0.956277000	9.975240000
C	9.289081000	1.524692000	8.593996000
H	10.619045000	2.213157000	7.036167000
H	10.088476000	1.075987000	9.188234000

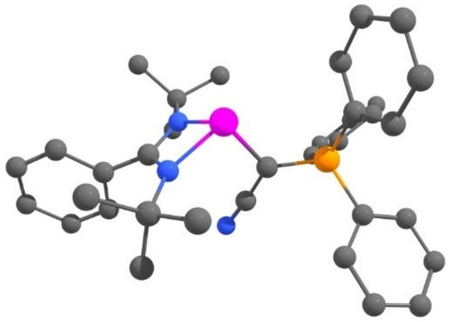

AYSi-3

E = -2150.32166346

Si	28.008270000	8.495859000	9.110436000
N	27.641690000	6.628834000	9.274481000
N	26.232116000	8.161236000	9.749853000
C	27.551416000	8.634578000	7.263324000
C	26.421647000	6.846860000	9.758973000
C	28.408199000	5.404512000	9.077613000
C	25.091138000	8.989479000	10.116615000
C	26.535548000	7.796388000	6.791147000
P	28.118288000	9.862550000	6.225935000
C	25.453570000	5.816719000	10.216071000
C	27.670424000	4.429918000	8.154251000
C	29.715685000	5.831475000	8.408166000
C	28.722778000	4.736004000	10.419476000
C	23.819840000	8.546756000	9.385772000
C	24.871839000	8.966024000	11.632532000
C	25.444354000	10.412874000	9.678367000
N	25.677114000	7.054683000	6.492245000
C	29.505885000	10.732816000	6.990554000
C	26.899200000	11.154152000	5.793173000
C	28.697282000	9.228941000	4.619285000
C	25.395400000	5.419607000	11.555096000
C	24.583375000	5.245574000	9.279258000
H	27.336509000	4.943138000	7.240571000
H	26.786403000	4.000896000	8.645046000
H	28.336623000	3.598173000	7.877348000
H	29.510291000	6.320995000	7.444633000
H	30.360973000	4.958690000	8.231406000
H	30.262078000	6.548816000	9.040191000
H	29.375602000	3.861724000	10.270756000
H	27.804517000	4.389885000	10.915210000
H	29.236496000	5.443462000	11.088155000
H	24.015145000	8.436386000	8.309012000
H	23.024420000	9.293935000	9.531450000
H	23.449094000	7.584926000	9.765120000
H	24.062790000	9.656783000	11.917289000
H	25.790573000	9.270555000	12.156620000
H	24.592044000	7.959349000	11.974793000
H	25.583229000	10.459229000	8.588073000
H	26.378425000	10.743489000	10.160503000

H	24.643135000	11.111792000	9.959517000
C	29.262380000	11.563806000	8.092315000
C	30.814288000	10.526159000	6.545854000
C	25.555627000	10.776121000	5.669152000
C	27.267521000	12.489211000	5.589251000
C	28.832974000	10.063644000	3.503658000
C	29.037863000	7.876191000	4.525093000
H	26.077144000	5.867369000	12.282021000
C	24.473166000	4.455032000	11.957382000
C	23.663479000	4.283288000	9.688516000
H	24.641222000	5.572701000	8.236507000
H	28.240030000	11.724529000	8.441732000
C	30.325695000	12.190951000	8.734520000
C	31.875036000	11.154596000	7.195792000
H	31.003358000	9.872393000	5.691391000
H	25.261333000	9.735941000	5.833754000
C	24.594751000	11.728656000	5.339962000
C	26.299509000	13.438125000	5.263480000
H	28.311996000	12.792077000	5.698345000
H	28.546281000	11.116029000	3.567121000
C	29.323005000	9.547905000	2.307127000
C	29.522167000	7.363513000	3.323191000
H	28.908043000	7.234042000	5.399703000
H	24.431850000	4.146445000	13.004632000
C	23.606719000	3.886588000	11.024829000
H	22.984847000	3.840097000	8.955985000
H	30.134490000	12.835143000	9.595380000
C	31.630736000	11.985905000	8.287403000
H	32.897183000	10.992596000	6.846423000
H	23.548586000	11.428877000	5.245233000
C	24.964180000	13.058745000	5.138156000
H	26.590753000	14.480231000	5.111871000
H	29.428703000	10.200222000	1.437196000
C	29.668398000	8.198654000	2.216906000
H	29.780145000	6.304604000	3.250443000
H	22.883639000	3.130906000	11.341213000
H	32.463983000	12.477182000	8.795618000
H	24.206195000	13.804199000	4.885867000
H	30.046218000	7.795064000	1.274360000

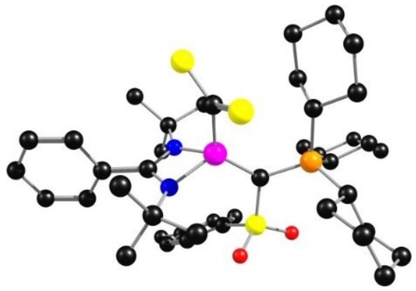

4

E = -3138.14290991

S	6.907402000	8.873046000	10.927597000
O	7.168416000	8.627974000	9.416168000
C	5.445952000	9.663699000	10.714428000
O	6.989385000	7.677409000	11.781160000
C	8.351262000	9.824918000	11.397302000
P	4.153722000	10.127925000	11.857974000
Si	5.444389000	9.458442000	8.881536000

C	9.531707000	9.646984000	10.676345000
C	8.330937000	10.649995000	12.516115000
C	4.868241000	11.413318000	13.004322000
C	3.841940000	8.692455000	13.021959000
N	4.864574000	7.824449000	8.144753000
N	3.570250000	9.528829000	8.422472000
N	6.190142000	10.695135000	7.859345000
C	3.596092000	8.252515000	8.085892000
H	9.539872000	8.982329000	9.811137000
C	10.679572000	10.330269000	11.064955000
C	9.484747000	11.331905000	12.889679000
H	7.417237000	10.768246000	13.097888000
H	5.680387000	10.964211000	13.604594000
C	5.435092000	12.575162000	12.195251000
C	3.805688000	11.913527000	13.984831000
H	2.896200000	8.982468000	13.521331000
C	4.877529000	8.408557000	14.109779000
C	3.552032000	7.446827000	12.185097000
C	5.413001000	6.460345000	7.962869000
C	2.437675000	10.473674000	8.444732000
C	7.250039000	11.359420000	8.547619000
C	6.399029000	10.637452000	6.383905000
C	2.416740000	7.426740000	7.722291000
H	11.601442000	10.192991000	10.493132000
C	10.675576000	11.193390000	12.167810000
H	9.456646000	11.987585000	13.764214000
H	4.622816000	12.991370000	11.571858000
H	6.195601000	12.204721000	11.490914000
C	5.992530000	13.675532000	13.088879000
C	4.357122000	13.013863000	14.888201000
H	3.419057000	11.082912000	14.597621000
H	2.945207000	12.299743000	13.408283000
H	5.000712000	9.289309000	14.760427000
H	5.851744000	8.210337000	13.637394000
C	4.481633000	7.202026000	14.958415000
C	3.174063000	6.244145000	13.041199000
H	2.752119000	7.678301000	11.461194000
H	4.453276000	7.209922000	11.599301000
C	6.817882000	6.571675000	7.362706000
C	5.471058000	5.746909000	9.315240000
C	4.595578000	5.610275000	6.983388000
C	1.786509000	10.618337000	7.064551000
C	1.376150000	10.054204000	9.464747000
C	3.019119000	11.827589000	8.834736000
H	8.154311000	10.767476000	8.735774000
C	7.255395000	12.625029000	8.999599000
C	7.781442000	10.056721000	6.055329000
C	5.333434000	9.785783000	5.702360000
C	6.304122000	12.050458000	5.803846000
C	1.760485000	6.685172000	8.708304000
C	1.976021000	7.364002000	6.397751000
C	11.901493000	11.971420000	12.543593000
H	6.868470000	13.287975000	13.641293000
H	6.364705000	14.512183000	12.473601000
C	4.944126000	14.167387000	14.081272000
H	5.144406000	12.586337000	15.535748000
H	3.567694000	13.381180000	15.564429000
H	3.560601000	7.436372000	15.524347000
H	5.263804000	6.998648000	15.708330000
C	4.238707000	5.968317000	14.096643000
H	3.028061000	5.357243000	12.401032000
H	2.205326000	6.433000000	13.539779000
H	7.247504000	5.563670000	7.268431000
H	6.777116000	7.012671000	6.355527000
H	7.478784000	7.173615000	7.993953000
H	6.100592000	6.289048000	10.032231000
H	4.461925000	5.649673000	9.742926000

H	5.884110000	4.734121000	9.190863000
H	3.620532000	5.302150000	7.377798000
H	4.440048000	6.130546000	6.027041000
H	5.169191000	4.695336000	6.775328000
H	1.272699000	9.702051000	6.747949000
H	1.033972000	11.419726000	7.106894000
H	2.525420000	10.896630000	6.299139000
H	1.835524000	9.949642000	10.458188000
H	0.597729000	10.830306000	9.522777000
H	0.888129000	9.112044000	9.179798000
H	3.500481000	11.769883000	9.820707000
H	3.768652000	12.138680000	8.097104000
H	2.221445000	12.583087000	8.878393000
C	6.151906000	13.623766000	8.815464000
C	8.451520000	13.151694000	9.745012000
H	7.909760000	9.070236000	6.522134000
H	7.906045000	9.947331000	4.967023000
H	8.585467000	10.712586000	6.419553000
H	4.328234000	10.150630000	5.945734000
H	5.464364000	9.842827000	4.612106000
H	5.402221000	8.731721000	5.996577000
H	7.040033000	12.719064000	6.270264000
H	6.501414000	12.031635000	4.720926000
H	5.300085000	12.471448000	5.963675000
H	2.108985000	6.734293000	9.741332000
C	0.672501000	5.884512000	8.369178000
C	0.888816000	6.561970000	6.062528000
H	2.498425000	7.933735000	5.626354000
H	11.931079000	12.183459000	13.621991000
H	12.820876000	11.432990000	12.272160000
H	11.922322000	12.941144000	12.018197000
H	5.372243000	14.928790000	14.753749000
H	4.130826000	14.666894000	13.523960000
H	5.181294000	5.690907000	13.592880000
H	3.947297000	5.109760000	14.723971000
H	5.363876000	13.202503000	8.182971000
H	5.694147000	13.827906000	9.799278000
C	6.671398000	14.944299000	8.241853000
C	8.961564000	14.465662000	9.154314000
H	8.153600000	13.334201000	10.794983000
H	9.247516000	12.393641000	9.778702000
H	0.164841000	5.306456000	9.144452000
C	0.235969000	5.820401000	7.046749000
H	0.551655000	6.512716000	5.024760000
H	5.851036000	15.679180000	8.199344000
H	6.999603000	14.785380000	7.199463000
C	7.840151000	15.492467000	9.051770000
H	9.372605000	14.273915000	8.146939000
H	9.792646000	14.856994000	9.764027000
H	-0.615896000	5.190078000	6.781495000
H	7.489969000	15.748737000	10.068988000
H	8.211633000	16.429573000	8.605716000

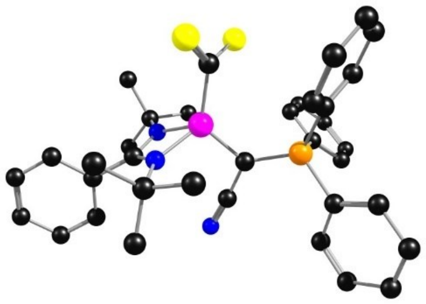

5

E = -2984.57056842

Si	2.821351000	8.874098000	3.344628000

N	3.732174000	5.524164000	4.215686000
C	1.962990000	7.300637000	3.642230000
S	2.286505000	11.321321000	1.577026000
S	1.003418000	10.874334000	4.306368000
P	0.273289000	6.946554000	3.708021000
N	4.290820000	9.009229000	4.446841000
C	2.886791000	6.293000000	3.962555000
N	4.326481000	8.482959000	2.362156000
C	-0.397573000	6.831137000	5.392103000
C	-1.178622000	5.751108000	5.815529000
H	-1.405192000	4.932889000	5.128736000
C	-1.664231000	5.720495000	7.122256000
H	-2.268469000	4.873723000	7.455649000
C	-1.378664000	6.765772000	7.998635000
H	-1.760187000	6.737758000	9.022218000
C	-0.608721000	7.848899000	7.571008000
H	-0.390920000	8.673275000	8.253856000
C	-0.113563000	7.884462000	6.271528000
H	0.472556000	8.738890000	5.916275000
C	5.091760000	8.664470000	3.434953000
C	6.554652000	8.463036000	3.508372000
C	7.042960000	7.188149000	3.814432000
H	6.337533000	6.368967000	3.979938000
C	8.416553000	6.989344000	3.914685000
H	8.801941000	5.996528000	4.157068000
C	9.297484000	8.052166000	3.710149000
H	10.374956000	7.890953000	3.791439000
C	8.805720000	9.319649000	3.401982000
H	9.494426000	10.151795000	3.240322000
C	7.431716000	9.529519000	3.300422000
H	7.037451000	10.519999000	3.062239000
C	4.559445000	9.405968000	5.835157000
C	4.786309000	10.919403000	5.865743000
H	4.937459000	11.265270000	6.899697000
H	5.677899000	11.191241000	5.280109000
H	3.914938000	11.443601000	5.445323000
C	3.307261000	9.049616000	6.634787000
H	3.438720000	9.342531000	7.686661000
H	2.436859000	9.586791000	6.229078000
H	3.112547000	7.967624000	6.591570000
C	5.751086000	8.663994000	6.438881000
H	5.793977000	8.893231000	7.513831000
H	5.632665000	7.576530000	6.325101000
H	6.710015000	8.963508000	5.997128000
C	4.642395000	8.098716000	0.981282000
C	5.412489000	6.777900000	0.933214000
H	4.902682000	6.009818000	1.534112000
H	5.470377000	6.427734000	-0.108290000
H	6.440104000	6.887567000	1.304206000
C	1.976020000	10.482319000	2.982831000
C	3.296047000	7.924021000	0.279744000
H	2.720832000	7.107918000	0.743383000
H	2.723849000	8.864771000	0.338589000
H	3.454115000	7.678438000	-0.780092000
C	5.423608000	9.226577000	0.305104000
H	6.409763000	9.361932000	0.772682000
H	5.584363000	8.991001000	-0.757899000
H	4.854213000	10.165239000	0.381536000
C	-0.102737000	5.347168000	2.928232000
C	-1.101427000	5.256984000	1.951855000
C	0.597528000	4.195494000	3.317481000
C	-1.391692000	4.027432000	1.363321000
H	-1.651744000	6.150479000	1.649380000
C	0.301994000	2.972281000	2.722573000
H	1.380763000	4.249009000	4.076187000
C	-0.690649000	2.885963000	1.746253000
H	-2.170751000	3.964462000	0.600226000

H	0.855116000	2.080248000	3.024790000
H	-0.917966000	1.923365000	1.282030000
C	-0.678731000	8.206108000	2.840850000
C	-1.840415000	8.742900000	3.406033000
C	-0.245430000	8.632845000	1.580979000
C	-2.544991000	9.727281000	2.721360000
H	-2.174771000	8.414316000	4.391693000
C	-0.953155000	9.621702000	0.905756000
H	0.658394000	8.211142000	1.138783000
C	-2.097643000	10.172272000	1.477392000
H	-3.439269000	10.163707000	3.171172000
H	-0.587668000	9.982706000	-0.057112000
H	-2.640720000	10.962496000	0.954140000

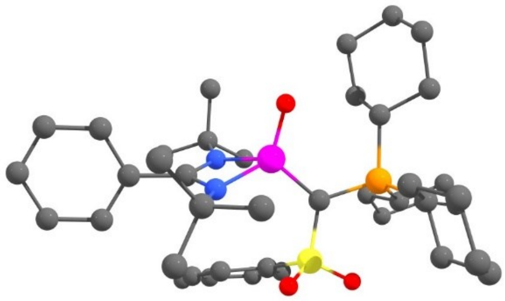

6

E = -2961.96737

S	1.952011000	4.059594000	5.982820000
O	0.616319000	4.677317000	5.837531000
C	3.022195000	4.745520000	4.828768000
O	2.019596000	2.587691000	5.977038000
C	2.486625000	4.522914000	7.627619000
P	2.274009000	5.692507000	3.549641000
Si	4.758796000	4.167284000	4.692798000
C	1.956176000	5.661134000	8.229507000
C	3.378541000	3.710697000	8.321879000
C	3.611430000	6.351313000	2.475975000
C	1.203089000	7.040107000	4.199011000
C	1.220103000	4.703058000	2.399971000
N	5.148800000	2.541688000	5.499235000
N	5.797479000	4.455052000	6.237142000
C	5.973058000	3.145195000	6.358466000
H	1.197113000	6.240476000	7.702909000
C	2.379987000	6.022926000	9.505170000
C	3.790010000	4.079734000	9.598112000
H	3.716301000	2.785461000	7.852723000
H	4.305915000	5.481778000	2.445434000
C	4.429752000	7.466699000	3.129973000
C	3.235389000	6.734175000	1.042545000
H	0.377865000	6.474160000	4.659959000
C	1.857734000	7.851308000	5.317792000
C	0.626759000	7.961151000	3.123267000
H	1.022337000	5.392853000	1.559464000
C	-0.132542000	4.241721000	2.944167000
C	2.026674000	3.511280000	1.878293000
C	5.050470000	1.145375000	5.058313000

C	6.441899000	5.623212000	6.834896000
C	6.974814000	2.471670000	7.220167000
H	1.965716000	6.919387000	9.974304000
C	3.315789000	5.250834000	10.204029000
H	4.490013000	3.443271000	10.146954000
H	3.846443000	8.404211000	3.165663000
H	4.675886000	7.201110000	4.167414000
C	5.710275000	7.691239000	2.335201000
C	4.506463000	7.003603000	0.236764000
H	2.669692000	5.931700000	0.547633000
H	2.592079000	7.631165000	1.029095000
H	2.297718000	7.176111000	6.064157000
H	2.688379000	8.451704000	4.915449000
C	0.831745000	8.782948000	5.951777000
C	-0.391538000	8.913526000	3.747427000
H	1.437741000	8.547758000	2.658520000
H	0.151706000	7.382818000	2.315021000
H	0.022869000	3.597915000	3.821833000
H	-0.721926000	5.098616000	3.304816000
C	-0.907081000	3.484352000	1.869934000
C	1.251338000	2.737449000	0.818391000
H	3.005852000	3.829168000	1.487514000
H	2.243836000	2.853072000	2.733239000
C	4.093110000	1.163952000	3.867476000
C	4.457044000	0.275043000	6.166930000
C	6.411806000	0.614439000	4.603306000
C	5.344086000	6.671441000	7.031846000
C	7.078799000	5.343901000	8.195670000
C	7.491351000	6.139922000	5.844855000
C	6.617046000	1.854796000	8.420259000
C	8.312332000	2.466804000	6.808531000
C	3.809110000	5.664361000	11.558814000
H	6.318156000	8.479040000	2.809726000
H	6.292334000	6.754597000	2.373296000
C	5.394654000	8.056088000	0.889916000
H	5.071282000	6.059215000	0.150409000
H	4.238093000	7.307232000	-0.788451000
H	0.040271000	8.177059000	6.429114000
H	1.302031000	9.374974000	6.753807000
C	0.205773000	9.703091000	4.907978000
H	-0.785930000	9.598769000	2.979660000
H	-1.251635000	8.324038000	4.111964000
H	-1.872670000	3.147564000	2.280189000
H	-1.145226000	4.168959000	1.034674000
C	-0.112347000	2.297935000	1.337204000
H	1.116964000	3.374165000	-0.075505000
H	1.841062000	1.865720000	0.491837000
H	4.490410000	1.811597000	3.070496000
H	3.115024000	1.544524000	4.194244000
H	3.966135000	0.148058000	3.466029000
H	4.289906000	-0.746245000	5.791911000
H	3.491540000	0.692550000	6.487414000
H	5.134680000	0.203545000	7.029648000
H	6.286837000	-0.373856000	4.135589000
H	7.112168000	0.499414000	5.442071000
H	6.854043000	1.296462000	3.861287000
H	4.795700000	6.850603000	6.097289000
H	5.781575000	7.623341000	7.367402000

H	4.616019000	6.334422000	7.781955000
H	7.962543000	4.697257000	8.130764000
H	6.353532000	4.880963000	8.879885000
H	7.396663000	6.301458000	8.634498000
H	7.044349000	6.282938000	4.850255000
H	8.310354000	5.413276000	5.735927000
H	7.918877000	7.092581000	6.193145000
H	5.574899000	1.852809000	8.741605000
C	7.592110000	1.248434000	9.209786000
C	9.281729000	1.852654000	7.595556000
H	8.584107000	2.940974000	5.862811000
H	4.800013000	6.142736000	11.482428000
H	3.917719000	4.799590000	12.230122000
H	3.130200000	6.385875000	12.034467000
H	4.879015000	9.034135000	0.864387000
H	6.323709000	8.180459000	0.310176000
H	-0.563554000	10.343787000	5.368441000
H	0.984020000	10.384299000	4.518081000
H	-0.677173000	1.777384000	0.546781000
H	0.031785000	1.567974000	2.153595000
H	7.306813000	0.772297000	10.150503000
C	8.923937000	1.246353000	8.799976000
H	10.323285000	1.847720000	7.266654000
H	9.686041000	0.767781000	9.419211000
O	5.552052000	4.432204000	3.372088000

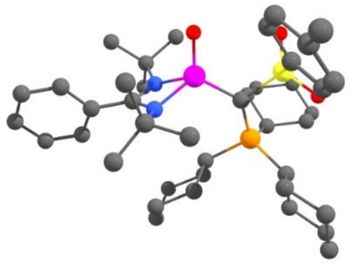

6_conformer

E = -2962.84913401

S	2.805945000	6.222786000	3.290858000
O	1.357273000	6.530837000	3.165895000
C	2.956353000	5.067324000	4.583816000
O	3.714284000	7.358555000	3.456625000
C	3.226094000	5.408499000	1.753317000
P	1.529204000	4.943245000	5.574127000
Si	4.664244000	4.458482000	4.826564000
C	2.188145000	4.941807000	0.949062000
C	4.557295000	5.276391000	1.356353000
C	1.924995000	3.707567000	6.902859000
C	-0.003312000	4.454613000	4.672875000
C	1.061595000	6.500658000	6.474919000
N	4.828646000	2.614854000	5.122638000
N	5.278686000	4.141611000	6.576724000
C	5.441638000	2.845411000	6.279799000
H	1.155668000	5.110699000	1.258051000
C	2.487860000	4.291082000	-0.244950000
C	4.834230000	4.620010000	0.161102000
H	5.349485000	5.620664000	2.026712000
H	3.013089000	3.829589000	7.042033000
C	1.658821000	2.274327000	6.429695000
C	1.266629000	3.958520000	8.263287000

H	-0.187745000	5.379300000	4.103314000
C	0.190340000	3.358625000	3.625602000
C	-1.224284000	4.190678000	5.556047000
H	0.478358000	6.144575000	7.342256000
C	0.181936000	7.507345000	5.727931000
C	2.337616000	7.171250000	6.983091000
C	4.868211000	1.511682000	4.156125000
C	6.003047000	5.015233000	7.514194000
C	6.208917000	1.876510000	7.101573000
H	1.673912000	3.921412000	-0.874986000
C	3.813906000	4.113699000	-0.655422000
H	5.877065000	4.497569000	-0.145861000
H	0.582676000	2.150557000	6.233322000
H	2.183339000	2.096256000	5.482547000
C	2.081732000	1.237062000	7.460331000
C	1.724806000	2.922362000	9.286216000
H	1.513397000	4.963130000	8.636184000
H	0.167670000	3.916290000	8.167832000
H	1.080082000	3.578484000	3.019091000
H	0.368554000	2.384795000	4.107753000
C	-1.056118000	3.256893000	2.754170000
C	-2.472051000	4.062890000	4.683326000
H	-1.091230000	3.260640000	6.134634000
H	-1.364532000	4.998779000	6.291664000
H	0.694487000	7.832960000	4.812172000
H	-0.756834000	7.035220000	5.399844000
C	-0.129701000	8.704561000	6.621291000
C	2.034799000	8.385517000	7.851972000
H	2.952824000	6.448974000	7.542571000
H	2.937248000	7.468604000	6.105970000
C	3.745734000	1.780740000	3.150081000
C	4.650495000	0.155287000	4.822419000
C	6.199062000	1.558159000	3.392271000
C	5.954848000	6.413431000	6.892482000
C	5.308274000	5.013743000	8.877225000
C	7.472866000	4.618294000	7.672161000
C	5.716116000	1.462519000	8.342779000
C	7.456549000	1.412557000	6.670089000
C	4.139931000	3.428642000	-1.950436000
H	1.816376000	0.230519000	7.098460000
H	3.179022000	1.244851000	7.550340000
C	1.449835000	1.498801000	8.820410000
H	2.808498000	3.052734000	9.459262000
H	1.234627000	3.115475000	10.253755000
H	-1.178704000	4.199193000	2.190681000
H	-0.929369000	2.458477000	2.005308000
C	-2.301125000	3.003573000	3.598836000
H	-3.348833000	3.834129000	5.310733000
H	-2.673370000	5.039896000	4.208910000
H	-0.753096000	9.423151000	6.065399000
H	-0.732226000	8.375712000	7.488855000
C	1.142559000	9.379239000	7.119502000
H	1.532768000	8.057004000	8.781019000
H	2.978901000	8.863384000	8.159452000
H	3.889297000	2.748643000	2.645918000
H	2.760009000	1.795144000	3.633653000
H	3.739327000	0.995846000	2.380333000
H	4.640149000	-0.632021000	4.054022000

H	3.687289000	0.126577000	5.352276000
H	5.446164000	-0.087538000	5.539729000
H	6.145209000	0.919562000	2.497719000
H	7.031094000	1.192937000	4.008964000
H	6.408074000	2.595739000	3.088875000
H	6.436125000	6.406920000	5.903011000
H	6.462047000	7.137831000	7.546137000
H	4.919096000	6.746119000	6.747177000
H	5.356878000	4.021071000	9.348374000
H	4.250801000	5.300509000	8.778852000
H	5.793012000	5.732463000	9.555167000
H	7.946891000	4.516216000	6.684705000
H	7.604889000	3.682897000	8.231282000
H	8.000457000	5.411676000	8.222197000
H	4.756915000	1.839936000	8.698114000
C	6.444322000	0.570373000	9.126611000
C	8.187277000	0.530852000	7.460395000
H	7.867911000	1.772053000	5.726559000
H	4.975772000	2.722532000	-1.831289000
H	4.443551000	4.160779000	-2.717056000
H	3.276112000	2.874905000	-2.344937000
H	0.357599000	1.346791000	8.750432000
H	1.815762000	0.771662000	9.563410000
H	-3.199433000	2.964753000	2.961824000
H	-2.216480000	2.009532000	4.075383000
H	0.898724000	10.232892000	7.772453000
H	1.694406000	9.788876000	6.255442000
H	6.044426000	0.246299000	10.089983000
C	7.680009000	0.100747000	8.686337000
H	9.164062000	0.182036000	7.118038000
H	8.253489000	-0.594909000	9.303122000
O	5.809197000	4.964235000	3.899660000

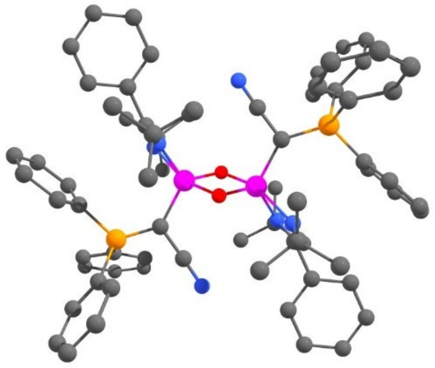

7

E = -4451.26309083

P	11.778769000	9.353438000	16.314094000
C	12.419279000	10.406938000	17.498143000
C	10.226202000	8.502460000	16.736433000
C	11.490749000	10.110580000	14.673731000
C	12.966912000	7.992503000	16.016508000
Si	11.735780000	11.228627000	19.023043000
C	13.773920000	10.672026000	17.238127000
C	10.280778000	7.344235000	17.520890000
C	8.998689000	8.919398000	16.212653000
C	10.766837000	9.482749000	13.650157000
C	12.053744000	11.370624000	14.449273000
C	13.074727000	7.332039000	14.788837000
C	13.769536000	7.590551000	17.091818000

Si	13.350880000	12.895398000	20.093476000
O	11.877110000	12.910484000	19.136753000
O	13.209509000	11.213532000	19.979820000
N	10.721138000	10.154361000	20.146425000
N	9.786526000	11.356065000	18.630584000
C	9.547239000	10.617758000	19.696636000
N	14.892485000	10.892173000	16.976181000
H	11.236664000	7.002011000	17.921390000
C	9.126658000	6.603165000	17.760538000
C	7.848103000	8.166823000	16.439283000
H	8.942180000	9.826930000	15.610269000
H	10.306921000	8.505561000	13.817095000
C	10.611008000	10.112812000	12.417952000
C	11.899894000	11.994329000	13.213131000
H	12.603690000	11.862734000	15.254129000
H	12.482193000	7.657145000	13.932397000
C	13.959702000	6.265920000	14.645179000
C	14.650699000	6.522857000	16.943358000
H	13.708876000	8.133174000	18.038157000
C	12.667406000	13.717375000	21.618259000
N	14.365417000	13.969835000	18.970068000
N	15.300104000	12.767959000	20.485719000
C	15.539327000	13.506400000	19.419711000
C	11.059485000	9.196921000	21.205747000
C	8.950654000	12.398599000	18.022795000
C	8.229123000	10.381348000	20.338506000
H	9.181534000	5.699471000	18.371331000
C	7.911577000	7.004368000	17.206102000
H	6.895482000	8.497210000	16.019227000
H	10.039003000	9.622988000	11.626355000
C	11.178937000	11.368240000	12.198257000
H	12.342604000	12.979202000	13.048421000
H	14.041990000	5.757619000	13.681644000
C	14.744487000	5.856929000	15.722441000
H	15.277465000	6.220783000	17.785413000
P	13.308016000	14.770603000	22.802501000
C	11.312717000	13.452423000	21.878183000
C	14.026933000	14.927511000	17.911013000
C	16.135993000	11.725297000	21.093274000
C	16.857379000	13.742881000	18.777748000
C	12.281236000	8.402711000	20.731577000
C	11.416606000	9.971964000	22.476683000
C	9.934284000	8.202700000	21.497762000
C	8.853832000	13.613765000	18.951990000
C	7.537703000	11.914355000	17.682708000
C	9.660079000	12.806257000	16.732011000
C	7.875322000	11.112462000	21.477241000
C	7.351415000	9.428630000	19.815759000
H	7.009232000	6.413296000	17.379644000
H	11.053181000	11.861640000	11.231435000
H	15.441494000	5.023552000	15.605415000
C	14.860404000	15.621917000	22.380150000
C	13.596445000	14.013103000	24.442640000
C	12.119759000	16.131275000	23.100658000
N	10.194087000	13.232438000	22.139977000
C	12.805309000	15.721652000	18.385623000
C	13.669536000	14.152742000	16.639974000
C	15.152111000	15.921752000	17.619009000

C	16.232819000	10.510303000	20.163851000
C	17.548963000	12.209462000	21.433393000
C	15.426622000	11.317390000	22.384008000
C	17.210969000	13.011910000	17.638858000
C	17.735209000	14.695487000	19.300479000
H	12.026382000	7.801214000	19.847001000
H	12.614445000	7.719193000	21.527136000
H	13.102838000	9.084097000	20.475025000
H	12.241578000	10.662643000	22.255821000
H	11.728724000	9.281569000	23.276389000
H	10.560159000	10.564818000	22.827615000
H	9.586038000	7.714514000	20.575107000
H	9.070932000	8.667606000	21.989745000
H	10.323879000	7.423279000	22.170079000
H	9.861323000	13.964706000	19.206738000
H	8.340814000	13.356145000	19.889086000
H	8.288420000	14.420705000	18.458955000
H	7.037828000	12.675210000	17.063758000
H	6.924914000	11.761140000	18.580083000
H	7.558952000	10.972387000	17.117728000
H	9.729869000	11.961332000	16.031412000
H	10.678090000	13.151734000	16.955687000
H	9.111937000	13.620209000	16.234567000
H	8.565942000	11.864875000	21.874217000
C	6.640481000	10.880493000	22.082535000
C	6.126099000	9.196875000	20.432282000
H	7.636912000	8.866371000	18.927766000
C	14.805569000	16.780406000	21.596104000
C	16.088058000	15.204943000	22.903583000
C	14.320675000	14.640639000	25.466169000
C	13.033380000	12.753070000	24.666964000
C	12.012079000	16.791278000	24.328592000
C	11.317000000	16.533601000	22.025589000
H	13.060361000	16.322914000	19.270303000
H	12.471978000	16.405364000	17.590284000
H	11.983728000	15.040233000	18.642170000
H	12.844613000	13.462009000	16.860860000
H	13.357258000	14.843316000	15.840485000
H	14.525920000	13.559977000	16.288734000
H	15.500549000	16.409723000	18.541707000
H	16.015350000	15.456936000	17.126745000
H	14.762401000	16.701337000	16.946949000
H	15.225338000	10.159407000	19.909015000
H	16.745852000	10.768105000	19.226812000
H	16.798232000	9.703273000	20.656737000
H	18.048846000	11.448499000	22.052203000
H	18.161721000	12.362804000	20.536020000
H	17.527764000	13.151337000	21.998529000
H	15.356795000	12.162207000	23.084739000
H	14.408628000	10.971878000	22.160302000
H	15.974822000	10.503393000	22.881316000
H	16.520283000	12.259551000	17.241886000
C	18.445723000	13.243907000	17.033394000
C	18.960430000	14.927282000	18.683786000
H	17.449879000	15.257633000	20.188597000
H	6.362784000	11.456598000	22.968460000
C	5.767130000	9.923365000	21.568080000
H	5.446469000	8.445422000	20.022659000

H	13.849574000	17.122659000	21.195874000
C	15.959569000	17.521687000	21.356502000
C	17.238512000	15.957730000	22.677029000
H	16.144768000	14.297215000	23.505647000
H	14.780602000	15.617843000	25.299345000
C	14.476765000	14.010279000	26.698188000
C	13.187496000	12.129059000	25.902918000
H	12.483172000	12.261212000	23.862134000
H	12.604729000	16.465862000	25.184834000
C	11.127087000	17.857317000	24.472754000
C	10.435814000	17.601209000	22.174564000
H	11.377574000	15.991356000	21.079034000
H	18.723256000	12.667894000	16.147357000
C	19.319194000	14.200933000	17.547830000
H	19.640153000	15.678649000	19.093413000
H	4.803499000	9.743475000	22.051269000
H	15.904489000	18.425574000	20.746012000
C	17.174782000	17.120437000	21.910606000
H	18.191241000	15.627323000	23.096829000
H	15.049009000	14.499876000	27.489753000
C	13.908785000	12.754847000	26.917741000
H	12.744736000	11.144189000	26.067518000
H	11.044903000	18.365262000	25.436485000
C	10.342157000	18.266679000	23.395739000
H	9.808929000	17.903577000	21.332703000
H	20.282754000	14.380843000	17.064506000
H	18.077035000	17.711666000	21.737115000
H	14.034758000	12.261210000	27.884414000
H	9.645140000	19.099993000	23.513157000

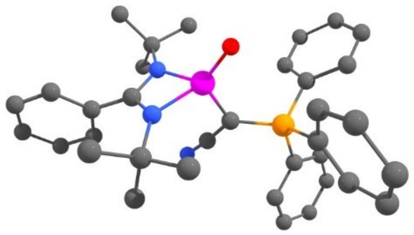

7_monomer

E = -2225.58477529

Si	12.372111000	12.340904000	20.178416000
O	12.425979000	10.911932000	20.782600000
C	12.217670000	13.789474000	21.304826000
N	11.390506000	12.763425000	18.667995000
N	13.525670000	12.916391000	18.828212000
C	12.487297000	13.195894000	18.045244000
P	12.305999000	13.580770000	23.009694000
C	11.987581000	15.033306000	20.713212000
C	9.990376000	12.679759000	18.256063000
C	14.937603000	13.294799000	18.762363000
C	12.527468000	13.920596000	16.753133000
C	13.798102000	12.738525000	23.629609000
C	10.906524000	12.642456000	23.692767000
C	12.269252000	15.198826000	23.828128000
N	11.810213000	16.016880000	20.101429000
C	9.249362000	12.092741000	19.457603000
C	9.854515000	11.734666000	17.060071000
C	9.415584000	14.059327000	17.925599000

C	15.607967000	12.623989000	17.561706000
C	15.110229000	14.814397000	18.695668000
C	15.561744000	12.766100000	20.054825000
C	12.778452000	13.241941000	15.557584000
C	12.311334000	15.304215000	16.748504000
C	14.639401000	13.373263000	24.552615000
C	14.097821000	11.444068000	23.174919000
C	11.055660000	11.714965000	24.727240000
C	9.636800000	12.912549000	23.171631000
C	11.477373000	15.416468000	24.960337000
C	13.083729000	16.228120000	23.336984000
H	9.296925000	12.786399000	20.309110000
H	8.191970000	11.921181000	19.209541000
H	9.704438000	11.141473000	19.772928000
H	10.274664000	10.746941000	17.302016000
H	8.793913000	11.606576000	16.795088000
H	10.375095000	12.133202000	16.177279000
H	9.637095000	14.774070000	18.732115000
H	9.830443000	14.458986000	16.990191000
H	8.323993000	13.987113000	17.803704000
H	15.460553000	11.534260000	17.599269000
H	15.202038000	13.001936000	16.612791000
H	16.689349000	12.828795000	17.570314000
H	16.173024000	15.075042000	18.814267000
H	14.771416000	15.218192000	17.731614000
H	14.533319000	15.307099000	19.491857000
H	15.116571000	13.260988000	20.931228000
H	15.390221000	11.684089000	20.155446000
H	16.644689000	12.956150000	20.063605000
H	12.946676000	12.162735000	15.570859000
C	12.810876000	13.945342000	14.355101000
C	12.351729000	15.998579000	15.541959000
H	12.116526000	15.821613000	17.693279000
H	14.404129000	14.375469000	24.916342000
C	15.783312000	12.723500000	25.012544000
C	15.244850000	10.808380000	23.645036000
H	13.447580000	10.961382000	22.426630000
H	12.047760000	11.499438000	25.129534000
C	9.935356000	11.056141000	25.231747000
C	8.520926000	12.257847000	23.682753000
H	9.536277000	13.636458000	22.358635000
H	10.840179000	14.616398000	25.343297000
C	11.498565000	16.656683000	25.595333000
C	13.099182000	17.464133000	23.976127000
H	13.699903000	16.065172000	22.450732000
H	13.004014000	13.413244000	13.420885000
C	12.599074000	15.323124000	14.346769000
H	12.185979000	17.078241000	15.537998000
H	16.437254000	13.223869000	25.730604000
C	16.087496000	11.441436000	24.558834000
H	15.481256000	9.803798000	23.285855000
H	10.053402000	10.322034000	26.032025000
C	8.670991000	11.326310000	24.711253000
H	7.530842000	12.468071000	23.271774000
H	10.875674000	16.823885000	26.476932000
C	12.307698000	17.679556000	25.104278000
H	13.728854000	18.266057000	23.584773000
H	12.626806000	15.873462000	13.403316000

H	16.985290000	10.933448000	24.920057000
H	7.796096000	10.803949000	25.105720000
H	12.319503000	18.652579000	25.601210000

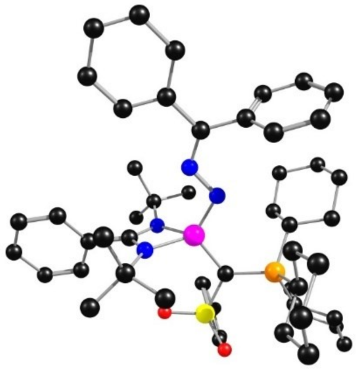

8$_{TS}$

E = -3498.07299151

Si	3.861394000	10.016455000	5.246757000
N	3.847401000	8.368503000	5.606337000
C	4.437150000	10.395753000	3.553564000
N	4.416138000	11.159013000	6.604953000
N	2.401768000	10.989369000	5.845426000
C	3.155480000	11.605384000	6.736056000
N	3.308992000	8.193001000	6.825101000
S	4.253678000	12.011310000	3.008834000
P	4.887585000	9.139348000	2.404841000
C	5.469227000	11.135164000	7.636224000
C	0.990128000	11.140509000	5.481345000
C	2.717990000	12.686715000	7.645003000
C	3.185886000	7.001541000	7.324723000
O	3.758450000	12.078838000	1.619833000
O	3.512887000	12.777618000	4.028506000
C	5.864789000	12.788320000	2.961865000
C	5.882593000	9.844213000	1.011927000
C	3.450698000	8.290650000	1.614536000
C	5.783090000	7.810019000	3.285876000
C	6.590493000	10.246719000	7.104626000
C	4.941065000	10.503414000	8.927424000
C	6.012717000	12.539356000	7.909313000
C	0.851387000	10.573403000	4.071636000
C	0.534442000	12.599722000	5.465194000
C	0.155195000	10.305716000	6.456388000
C	1.989773000	12.436981000	8.810038000
C	3.026431000	13.999411000	7.270879000
C	2.564372000	6.876717000	8.655182000
C	3.609265000	5.775418000	6.592518000
C	6.709502000	12.678463000	4.064457000
C	6.228660000	13.557097000	1.863983000
H	5.380859000	10.806113000	0.811821000
C	7.336192000	10.122724000	1.408977000
C	5.833220000	9.025546000	-0.284366000
H	3.902455000	7.659325000	0.828345000
C	2.708645000	7.363441000	2.579922000
C	2.506835000	9.293560000	0.948699000
H	5.000884000	7.498179000	4.012410000

C	6.188023000	6.618634000	2.417127000
C	6.956642000	8.264518000	4.151469000
H	6.207871000	9.240036000	6.882124000
H	7.385305000	10.157697000	7.858755000
H	7.041191000	10.660890000	6.191201000
H	4.506752000	9.516167000	8.713725000
H	4.173462000	11.131846000	9.398920000
H	5.760543000	10.383591000	9.652006000
H	6.337552000	13.027464000	6.978628000
H	6.877327000	12.475708000	8.587368000
H	5.260468000	13.181450000	8.386213000
H	1.449482000	11.163177000	3.363950000
H	-0.200770000	10.598356000	3.753570000
H	1.193808000	9.529120000	4.039343000
H	1.221331000	13.199805000	4.852207000
H	0.475986000	13.033756000	6.471653000
H	-0.471739000	12.651475000	5.022799000
H	-0.900330000	10.294033000	6.144599000
H	0.203133000	10.723166000	7.472363000
H	0.529690000	9.272102000	6.495994000
H	1.753145000	11.410049000	9.095657000
C	1.575696000	13.503112000	9.606095000
C	2.600822000	15.058656000	8.066448000
H	3.573495000	14.168681000	6.340318000
C	2.399093000	5.631067000	9.284479000
C	2.091045000	8.017694000	9.334338000
C	4.716084000	5.025288000	7.009055000
C	2.881764000	5.330401000	5.481685000
H	6.389722000	12.079595000	4.917860000
C	7.941380000	13.316918000	4.050664000
C	7.468574000	14.197221000	1.862887000
H	5.540470000	13.637735000	1.020094000
H	7.383448000	10.703695000	2.340598000
H	7.846076000	9.164597000	1.602006000
C	8.076232000	10.848406000	0.291041000
C	6.591616000	9.741371000	-1.399742000
H	6.276229000	8.028369000	-0.119771000
H	4.795134000	8.864390000	-0.606642000
H	3.386804000	6.598322000	2.981848000
H	2.374345000	7.933494000	3.460867000
C	1.524859000	6.687249000	1.897209000
C	1.333694000	8.590928000	0.275332000
H	2.138008000	9.992468000	1.712521000
H	3.045618000	9.924625000	0.226732000
H	5.357950000	6.280462000	1.777629000
H	7.010739000	6.911122000	1.740084000
C	6.641065000	5.468440000	3.313255000
C	7.355709000	7.123666000	5.079194000
H	7.819581000	8.546678000	3.528235000
H	6.674997000	9.154974000	4.724884000
H	1.011097000	13.308304000	10.520533000
C	1.878217000	14.812311000	9.234562000
H	2.831302000	16.084517000	7.770387000
H	2.735235000	4.724258000	8.777814000
C	1.807623000	5.533182000	10.543122000
C	1.503030000	7.917079000	10.587176000
H	2.194522000	8.982011000	8.835179000
H	5.292565000	5.364540000	7.873725000

C	5.075597000	3.851932000	6.346227000
C	3.238641000	4.160320000	4.815537000
H	2.016473000	5.909888000	5.153623000
H	8.606724000	13.221814000	4.913709000
C	8.345903000	14.083143000	2.944951000
H	7.761939000	14.796552000	0.996653000
H	7.615758000	11.838863000	0.138249000
H	9.117654000	11.036176000	0.598165000
C	8.029507000	10.055165000	-1.007760000
H	6.562831000	9.129888000	-2.316003000
H	6.065413000	10.683318000	-1.636677000
H	1.901213000	5.995222000	1.121182000
H	0.986228000	6.064102000	2.629888000
C	0.582129000	7.694164000	1.250944000
H	0.657141000	9.342683000	-0.161658000
H	1.705365000	7.979622000	-0.567863000
H	5.772945000	5.114470000	3.891744000
H	6.973282000	4.619240000	2.693705000
C	7.744703000	5.884466000	4.279657000
H	6.498115000	6.889804000	5.733277000
H	8.186629000	7.437699000	5.732017000
H	1.547077000	15.646149000	9.857978000
H	1.694534000	4.549867000	11.007292000
C	1.359224000	6.672256000	11.206940000
H	1.145807000	8.820161000	11.090465000
H	5.938491000	3.275559000	6.689708000
C	4.334553000	3.413256000	5.249723000
H	2.653341000	3.824405000	3.955233000
C	9.689580000	14.750313000	2.933377000
H	8.535885000	10.605349000	-1.817172000
H	8.586550000	9.109258000	-0.878487000
H	-0.245317000	7.175003000	0.740810000
H	0.122448000	8.319114000	2.036775000
H	8.670054000	6.095541000	3.711926000
H	7.979400000	5.048400000	4.958350000
H	0.895871000	6.593643000	12.193281000
H	4.612323000	2.492153000	4.731610000
H	10.500747000	14.004474000	2.915728000
H	9.837391000	15.365435000	3.834545000
H	9.811222000	15.398745000	2.054822000

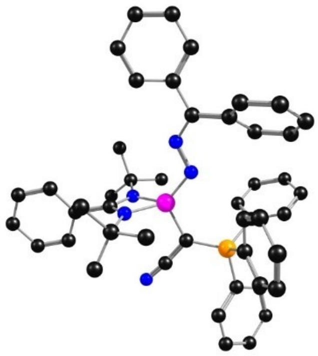

8CN

E = -2760.78307901

P	9.041049000	3.156686000	8.767207000
C	9.962131000	2.613578000	7.413209000
C	7.346563000	2.503494000	8.900100000
C	9.895442000	2.696526000	10.298469000

C	8.834149000	4.958142000	8.785938000
Si	9.462954000	2.891505000	5.687148000
C	11.078105000	1.777595000	7.542982000
C	6.950906000	1.678201000	9.959607000
C	6.430356000	2.852028000	7.896369000
C	10.288311000	1.364194000	10.489412000
C	10.158419000	3.648610000	11.288799000
C	9.770766000	5.753357000	8.122619000
C	7.783327000	5.550959000	9.493947000
N	8.186314000	3.939205000	5.490767000
C	10.689305000	1.639179000	4.206828000
N	10.942332000	2.893457000	4.590663000
N	9.553725000	1.265973000	4.786050000
N	12.004354000	1.062153000	7.522468000
H	7.654797000	1.418642000	10.752937000
C	5.644901000	1.192878000	10.006340000
C	5.127606000	2.365125000	7.956996000
H	6.754928000	3.485030000	7.058310000
H	10.101551000	0.615512000	9.717137000
C	10.934760000	0.993955000	11.664810000
C	10.806524000	3.269784000	12.462729000
H	9.860850000	4.688647000	11.140381000
H	10.578754000	5.271509000	7.570166000
C	9.653012000	7.139944000	8.158306000
C	7.681195000	6.939330000	9.542572000
H	7.039320000	4.927483000	9.995339000
N	7.906817000	4.243643000	4.211377000
C	11.576691000	0.798858000	3.373591000
C	11.827531000	3.920671000	4.039323000
C	8.863974000	-0.023595000	4.865547000
H	5.337599000	0.548492000	10.833393000
C	4.734421000	1.535335000	9.007210000
H	4.420213000	2.650704000	7.175024000
H	11.245246000	-0.043617000	11.805192000
C	11.193552000	1.944704000	12.651850000
H	11.012794000	4.017760000	13.231540000
H	10.377112000	7.759983000	7.625062000
C	8.610034000	7.733193000	8.869755000
H	6.863432000	7.403943000	10.098378000
C	6.798193000	4.853273000	3.921114000
C	11.378169000	0.666674000	1.997576000
C	12.643032000	0.145903000	4.004065000
C	13.271937000	3.433410000	3.928801000
C	11.283365000	4.364386000	2.678707000
C	11.763534000	5.089657000	5.021561000
C	8.334924000	-0.408851000	3.482455000
C	9.778611000	-1.115840000	5.423794000
C	7.686466000	0.196962000	5.815505000
H	3.710163000	1.156559000	9.051371000
H	11.704529000	1.650846000	13.571791000
H	8.517158000	8.821504000	8.896302000
C	5.776709000	5.212191000	4.939586000
C	6.545949000	5.144045000	2.496150000
H	10.543082000	1.179851000	1.515657000
C	12.252724000	-0.117666000	1.247456000
C	13.506611000	-0.639933000	3.246723000
H	12.780691000	0.261378000	5.083426000
H	13.392347000	2.665772000	3.153026000

H	13.613712000	3.016864000	4.888127000
H	13.922609000	4.280538000	3.664587000
H	11.344284000	3.542893000	1.948799000
H	11.868147000	5.209191000	2.283946000
H	10.229702000	4.668598000	2.775732000
H	12.371206000	5.928839000	4.653872000
H	12.150506000	4.783447000	6.005591000
H	10.725123000	5.437496000	5.137271000
H	9.157117000	-0.605177000	2.779801000
H	7.706982000	0.397705000	3.074905000
H	7.726354000	-1.323090000	3.552196000
H	9.190786000	-2.023648000	5.627491000
H	10.255678000	-0.785041000	6.358254000
H	10.571025000	-1.383397000	4.711208000
H	7.044465000	1.016166000	5.458490000
H	8.039359000	0.458346000	6.824106000
H	7.079111000	-0.716444000	5.888498000
C	6.107713000	5.959499000	6.079849000
C	4.452346000	4.774662000	4.790394000
C	5.607536000	6.109301000	2.095428000
C	7.262318000	4.466947000	1.491368000
H	12.101594000	-0.218739000	0.170445000
C	13.315068000	-0.770428000	1.870570000
H	14.337931000	-1.152291000	3.736035000
H	7.137986000	6.289899000	6.220492000
C	5.138041000	6.264558000	7.031618000
C	3.483864000	5.074304000	5.747145000
H	4.183982000	4.191218000	3.905785000
H	5.044367000	6.656024000	2.855008000
C	5.396442000	6.388969000	0.746495000
C	7.051966000	4.749033000	0.147749000
H	7.985251000	3.710906000	1.802794000
H	13.998359000	-1.385382000	1.280205000
H	5.414495000	6.852583000	7.909232000
C	3.824588000	5.822331000	6.873193000
H	2.457540000	4.723261000	5.610499000
H	4.663770000	7.149075000	0.462630000
C	6.115815000	5.712678000	-0.236395000
H	7.618251000	4.205851000	-0.613933000
H	3.068452000	6.061642000	7.625103000
H	5.947847000	5.930639000	-1.293899000

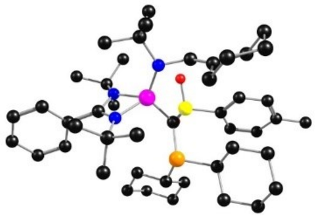

9

E = -3138.12199218

S	6.595994000	9.245056000	11.313968000
O	7.103165000	8.181791000	10.400057000
C	5.461148000	10.137970000	10.407344000
O	6.106022000	8.780017000	12.630867000
C	8.009890000	10.261230000	11.723144000
P	4.646668000	11.664609000	10.880635000
Si	4.859641000	9.173182000	9.011120000

C	8.865957000	10.669036000	10.704454000
C	8.268677000	10.607365000	13.044982000
C	5.997199000	12.922666000	11.156711000
C	3.888295000	11.479550000	12.585490000
N	3.790738000	7.679652000	9.471370000
N	3.084228000	9.540533000	8.626173000
N	5.826443000	8.995702000	7.580088000
C	2.701847000	8.371960000	9.152911000
H	8.656696000	10.356627000	9.683392000
C	9.962847000	11.464234000	11.007051000
C	9.365580000	11.416973000	13.335370000
H	7.610844000	10.236632000	13.831483000
H	6.726387000	12.545874000	11.894084000
C	6.725083000	13.164895000	9.837131000
C	5.416731000	14.238420000	11.678824000
H	3.245393000	12.379223000	12.663033000
C	4.806460000	11.447570000	13.804950000
C	2.969837000	10.258806000	12.569350000
C	3.909190000	6.377733000	10.170711000
C	2.277789000	10.632100000	8.043437000
C	7.165523000	9.495354000	7.670239000
C	5.640468000	8.061745000	6.424693000
C	1.298867000	7.923244000	9.335497000
H	10.632582000	11.784055000	10.203635000
C	10.222170000	11.867379000	12.325594000
H	9.563819000	11.699268000	14.372906000
H	5.982257000	13.497797000	9.087803000
H	7.135351000	12.215704000	9.462784000
C	7.813349000	14.222114000	9.969191000
C	6.500894000	15.303670000	11.823465000
H	4.911052000	14.089756000	12.645708000
H	4.640593000	14.591246000	10.973889000
H	5.445637000	12.343889000	13.838672000
H	5.467104000	10.574647000	13.711254000
C	4.005926000	11.323948000	15.098849000
C	2.184857000	10.106283000	13.867366000
H	2.284753000	10.335407000	11.709601000
H	3.595258000	9.366987000	12.404064000
C	5.266291000	5.776897000	9.808162000
C	3.821727000	6.594888000	11.682033000
C	2.831531000	5.379648000	9.733289000
C	1.373144000	10.127205000	6.914806000
C	1.432650000	11.326156000	9.112618000
C	3.268142000	11.627340000	7.449525000
H	7.880683000	8.830649000	8.169578000
C	7.612795000	10.663721000	7.178513000
C	6.725130000	6.978007000	6.451783000
C	4.272605000	7.387912000	6.447389000
C	5.746684000	8.839051000	5.111077000
C	0.639493000	8.136779000	10.548413000
C	0.646851000	7.247875000	8.298826000
C	11.380290000	12.770523000	12.631105000
H	8.596583000	13.844242000	10.649728000
H	8.301937000	14.389457000	8.993659000
C	7.250863000	15.529812000	10.515190000
H	7.217250000	14.979294000	12.600095000
H	6.059866000	16.248590000	12.181981000
H	3.379955000	12.225494000	15.236891000

H	4.689309000	11.285424000	15.963556000
C	3.112832000	10.088169000	15.076766000
H	1.577379000	9.184424000	13.839263000
H	1.472161000	10.945813000	13.966669000
H	5.416682000	4.856178000	10.390089000
H	5.302528000	5.506097000	8.742608000
H	6.086740000	6.469661000	10.036444000
H	4.614413000	7.266522000	12.039683000
H	2.845524000	7.020206000	11.958538000
H	3.926134000	5.629649000	12.200468000
H	1.834192000	5.618388000	10.121417000
H	2.773425000	5.298055000	8.637438000
H	3.109083000	4.390811000	10.126370000
H	0.546882000	9.508116000	7.285468000
H	0.931201000	10.992529000	6.399224000
H	1.944741000	9.551944000	6.171087000
H	2.089686000	11.744497000	9.888437000
H	0.861575000	12.147871000	8.654632000
H	0.713981000	10.632539000	9.571389000
H	3.973201000	11.972923000	8.220455000
H	3.826153000	11.164592000	6.624462000
H	2.729807000	12.501632000	7.056746000
C	6.768510000	11.703479000	6.502935000
C	9.070279000	11.027020000	7.281353000
H	6.745801000	6.472489000	7.426997000
H	6.532064000	6.228494000	5.669410000
H	7.721952000	7.403346000	6.268891000
H	3.466540000	8.133060000	6.471176000
H	4.153192000	6.792856000	5.530922000
H	4.158915000	6.715719000	7.304406000
H	6.714215000	9.350339000	5.030179000
H	5.657394000	8.148682000	4.258537000
H	4.945466000	9.589012000	5.029791000
H	1.149759000	8.659587000	11.358023000
C	-0.664401000	7.676669000	10.720957000
C	-0.654517000	6.789309000	8.476475000
H	1.169043000	7.068736000	7.356943000
H	11.583024000	12.818827000	13.710008000
H	12.297163000	12.434113000	12.123676000
H	11.176640000	13.797143000	12.283491000
H	8.055297000	16.270713000	10.655713000
H	6.556000000	15.962542000	9.772251000
H	3.750331000	9.187928000	15.026175000
H	2.529191000	10.013178000	16.009253000
H	5.748633000	11.329955000	6.370430000
H	6.696950000	12.573127000	7.179578000
C	7.363342000	12.173452000	5.173937000
C	9.644840000	11.454936000	5.930486000
H	9.173183000	11.878049000	7.981885000
H	9.648736000	10.190835000	7.703926000
H	-1.173493000	7.846102000	11.672220000
C	-1.312394000	7.002417000	9.687977000
H	-1.156746000	6.257841000	7.665186000
H	6.764086000	13.008138000	4.775626000
H	7.292765000	11.360144000	4.430826000
C	8.823015000	12.583604000	5.320898000
H	9.643292000	10.588824000	5.245035000
H	10.697065000	11.759843000	6.050037000

H	-2.333434000	6.639796000	9.826750000
H	8.889585000	13.473276000	5.974052000
H	9.239104000	12.882139000	4.344981000

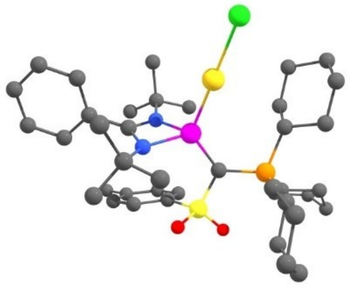

AYSi-2-AuCl

E = -3481.02731011

S	2.210043000	4.361961000	5.882109000
O	0.929348000	5.096416000	5.867949000
C	3.277472000	5.042219000	4.700218000
O	2.129680000	2.894606000	5.757622000
C	2.869437000	4.636553000	7.527112000
P	2.479910000	5.917055000	3.387297000
Si	4.996473000	4.411573000	4.613276000
C	2.745583000	5.889997000	8.124237000
C	3.323464000	3.549831000	8.266308000
C	3.753282000	6.651397000	2.280214000
C	1.326016000	7.219834000	3.984675000
C	1.484122000	4.821409000	2.288014000
N	5.292351000	2.758692000	5.425275000
N	5.976564000	4.646434000	6.184760000
C	6.121542000	3.326889000	6.302621000
H	2.320438000	6.722035000	7.562556000
C	3.125661000	6.061266000	9.450064000
C	3.684444000	3.730808000	9.599655000
H	3.362229000	2.568284000	7.793126000
H	4.509346000	5.841975000	2.228672000
C	4.459109000	7.867561000	2.882120000
C	3.328862000	6.939679000	0.836280000
H	0.547848000	6.616272000	4.476263000
C	1.912204000	8.123804000	5.068550000
C	0.669279000	8.051966000	2.882147000
H	1.161556000	5.478178000	1.459835000
C	0.231201000	4.228167000	2.937225000
C	2.375372000	3.712759000	1.723446000
C	5.159008000	1.355807000	4.999845000
C	6.704352000	5.761764000	6.797685000
C	7.126788000	2.630478000	7.143037000
H	3.029061000	7.047179000	9.912484000
C	3.605659000	4.986475000	10.211292000
H	4.019927000	2.872199000	10.188379000
H	3.763491000	8.723956000	2.897791000
H	4.739452000	7.668617000	3.925792000
C	5.686474000	8.236271000	2.056419000
C	4.559684000	7.311468000	0.009427000
H	2.841901000	6.066682000	0.379970000

H	2.596528000	7.764224000	0.808574000
H	2.426875000	7.513660000	5.821515000
H	2.673170000	8.793244000	4.639287000
C	0.803119000	8.962635000	5.691601000
C	-0.434647000	8.917064000	3.489275000
H	1.418889000	8.701684000	2.399048000
H	0.247873000	7.411359000	2.091670000
H	0.520692000	3.618021000	3.806264000
H	-0.422685000	5.022708000	3.326822000
C	-0.529335000	3.360528000	1.939112000
C	1.614373000	2.830189000	0.740699000
H	3.279094000	4.120984000	1.244402000
H	2.727066000	3.102824000	2.567280000
C	4.206154000	1.359385000	3.808112000
C	4.542962000	0.526043000	6.127182000
C	6.502272000	0.770510000	4.557261000
C	5.907357000	7.019347000	6.460106000
C	6.777023000	5.601820000	8.315860000
C	8.108752000	5.891186000	6.199639000
C	6.794276000	2.115649000	8.397990000
C	8.432819000	2.492494000	6.659328000
C	4.043280000	5.184595000	11.631455000
H	6.178188000	9.119285000	2.495588000
H	6.415736000	7.408261000	2.103391000
C	5.313273000	8.495577000	0.602550000
H	5.238288000	6.441585000	-0.035559000
H	4.253129000	7.528808000	-1.026316000
H	0.085517000	8.289689000	6.193483000
H	1.217900000	9.621805000	6.471630000
C	0.083677000	9.788472000	4.629108000
H	-0.893806000	9.542466000	2.706778000
H	-1.233009000	8.255457000	3.869697000
H	-1.418269000	2.931985000	2.428774000
H	-0.902603000	3.991513000	1.111407000
C	0.353045000	2.255040000	1.372358000
H	1.342377000	3.424272000	-0.150944000
H	2.274887000	2.023250000	0.385226000
H	4.639502000	1.925663000	2.968809000
H	3.247357000	1.806464000	4.103975000
H	4.028611000	0.329138000	3.467572000
H	4.421816000	-0.516683000	5.797589000
H	3.551417000	0.925579000	6.383278000
H	5.183806000	0.520571000	7.020407000
H	6.335090000	-0.208388000	4.083739000
H	7.189803000	0.618455000	5.399453000
H	6.983156000	1.429542000	3.817478000
H	5.899309000	7.183138000	5.373049000
H	6.368733000	7.899450000	6.930122000
H	4.868867000	6.931062000	6.806889000
H	7.409765000	4.753944000	8.609762000
H	5.772904000	5.454864000	8.736894000
H	7.210584000	6.511350000	8.757730000
H	8.058710000	5.925055000	5.100270000
H	8.750445000	5.050466000	6.495463000
H	8.585589000	6.816892000	6.556054000
H	5.779743000	2.237556000	8.776918000
C	7.762168000	1.473015000	9.166640000
C	9.396251000	1.847939000	7.430255000

H	8.685681000	2.877240000	5.668836000
H	5.092703000	5.522645000	11.668935000
H	3.978114000	4.251699000	12.209060000
H	3.436785000	5.948531000	12.138943000
H	4.681746000	9.401664000	0.541797000
H	6.217625000	8.701823000	0.009026000
H	-0.745647000	10.357984000	5.078446000
H	0.786934000	10.536414000	4.219580000
H	-0.205718000	1.652250000	0.638395000
H	0.639349000	1.569827000	2.190057000
H	7.496970000	1.075826000	10.149000000
C	9.063320000	1.339018000	8.685326000
H	10.412461000	1.739879000	7.044878000
H	9.820907000	0.834139000	9.289042000
Au	6.369333000	4.586262000	2.788949000
Cl	7.781794000	4.728623000	0.918918000

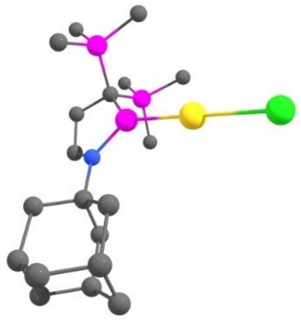

CAASi-AuCl

E = -2228.57331337

Si	5.829021000	3.273082000	9.530359000
N	7.291759000	1.519929000	10.035003000
C	7.107019000	4.278859000	10.428136000
C	8.821844000	2.328927000	10.823974000
C	7.388690000	0.882804000	10.008415000
Si	7.242238000	5.946073000	9.475097000
Si	6.409832000	4.606979000	12.178408000
C	8.581554000	3.796369000	10.587847000
H	9.871531000	2.078123000	11.026772000
H	8.347973000	2.212628000	11.826870000
C	6.881295000	2.315923000	8.187775000
C	5.313668000	1.711117000	10.593402000
C	7.583779000	-0.156271000	10.021284000
C	7.409798000	5.608191000	7.626179000
C	5.826942000	7.158576000	9.720492000
C	8.833835000	6.830069000	9.983188000
C	6.728717000	3.198001000	13.405928000
C	7.231958000	6.088969000	13.005987000
C	4.547080000	4.874779000	12.200125000
H	9.138371000	4.092892000	9.676828000
H	9.042674000	4.382862000	11.402529000
H	7.966299000	2.074620000	8.312003000
H	6.913596000	3.076031000	7.403461000
C	6.120851000	1.101417000	7.596148000
H	4.463643000	2.137079000	11.134662000
H	5.864076000	1.275800000	11.459548000
C	4.718588000	0.549692000	9.721224000
H	7.466791000	-0.600905000	10.962395000

H	8.581154000	-0.365010000	9.700503000
C	6.705464000	-0.896120000	9.073488000
H	6.479213000	5.213064000	7.189564000
H	8.239154000	4.917059000	7.408764000
H	7.636008000	6.557736000	7.114715000
H	5.715959000	7.493397000	10.761917000
H	4.863254000	6.747990000	9.381044000
H	6.040172000	8.049318000	9.107142000
H	8.852751000	7.806872000	9.472824000
H	9.736515000	6.280862000	9.678761000
H	8.896983000	7.020849000	11.063005000
H	6.284631000	3.515574000	14.364279000
H	7.799784000	3.023100000	13.589690000
H	6.259896000	2.238396000	13.147186000
H	7.037419000	7.037950000	12.487311000
H	8.322134000	5.959037000	13.088581000
H	6.832782000	6.180315000	14.029006000
H	4.242579000	5.087277000	13.238629000
H	3.972367000	4.000934000	11.859994000
H	4.229860000	5.720346000	11.574050000
H	5.955982000	1.288032000	6.524990000
C	4.729002000	0.931107000	8.227766000
C	6.806202000	-0.263315000	7.691524000
H	3.661332000	0.432645000	9.999452000
C	5.337281000	-0.862300000	9.746657000
H	7.095045000	-1.922935000	9.053650000
H	4.138088000	1.843712000	8.059959000
H	4.203983000	0.135807000	7.672021000
H	6.316952000	-0.934136000	6.969278000
H	7.871439000	-0.215059000	7.401956000
H	4.629424000	-1.524971000	9.226463000
H	5.438532000	-1.261177000	10.770940000
Au	3.990266000	4.330861000	8.703577000
Cl	2.088653000	5.399166000	7.841082000

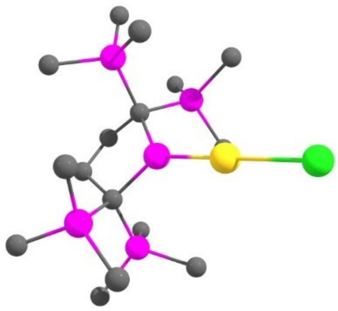

CA_2Si-AuCl

E = -2674.95208504

Si	2.739191000	8.852878000	9.828333000
C	1.987837000	7.158222000	9.721586000
C	2.766870000	9.046486000	11.674926000
Si	0.200434000	7.149532000	9.010786000
Si	3.172001000	6.169373000	8.562161000
C	2.041786000	6.663575000	11.195726000
Si	1.929375000	10.754698000	12.000288000
Si	4.536434000	9.017622000	12.427765000

C	1.897089000	7.850955000	12.159877000
C	-1.063576000	7.427089000	10.384933000
C	-0.258501000	5.460229000	8.307556000
C	-0.029518000	8.475996000	7.700686000
C	3.010429000	4.331636000	8.932100000
C	2.874388000	6.549211000	6.746622000
C	4.971577000	6.640556000	8.892889000
H	1.270875000	5.902545000	11.403488000
H	3.005803000	6.165658000	11.392476000
C	0.484519000	11.021670000	10.812718000
C	3.102940000	12.191628000	11.705240000
C	1.217034000	10.776290000	13.741749000
C	4.584721000	9.855150000	14.117248000
C	5.078553000	7.245014000	12.785255000
C	5.790775000	9.813558000	11.278128000
H	2.153235000	7.545283000	13.188272000
H	0.835392000	8.146570000	12.197652000
H	-1.004763000	8.420770000	10.848935000
H	-0.980220000	6.671452000	11.181105000
H	-2.069767000	7.329619000	9.945783000
H	-0.189168000	4.678440000	9.079957000
H	0.349531000	5.148146000	7.447356000
H	-1.308424000	5.502050000	7.974798000
H	-1.028319000	8.375178000	7.246160000
H	0.717336000	8.413865000	6.896642000
H	0.037934000	9.486347000	8.132980000
H	2.011697000	3.935662000	8.704265000
H	3.229663000	4.119492000	9.990072000
H	3.743251000	3.776613000	8.324477000
H	3.603106000	5.979677000	6.147504000
H	3.047906000	7.618769000	6.545305000
H	1.869744000	6.288808000	6.386610000
H	5.620574000	5.975416000	8.299828000
H	5.268622000	6.529274000	9.945144000
H	5.191956000	7.672447000	8.574035000
H	-0.031208000	11.954012000	11.095685000
H	-0.262373000	10.216070000	10.843203000
H	0.823768000	11.145930000	9.771301000
H	2.552672000	13.131355000	11.873879000
H	3.449089000	12.196591000	10.658954000
H	3.984719000	12.198756000	12.360369000
H	1.988450000	10.706268000	14.520397000
H	0.505215000	9.949320000	13.889493000
H	0.665772000	11.718418000	13.892830000
H	3.896949000	9.362426000	14.822063000
H	4.345732000	10.927243000	14.096800000
H	5.603988000	9.749347000	14.523045000
H	6.058237000	7.284657000	13.288692000
H	5.198508000	6.625572000	11.886279000
H	4.382680000	6.730451000	13.465591000
H	6.768831000	9.869758000	11.782722000
H	5.505479000	10.831149000	10.976075000
H	5.925477000	9.224402000	10.357804000
Au	3.415919000	10.251906000	8.205832000
Cl	4.116790000	11.696197000	6.529433000

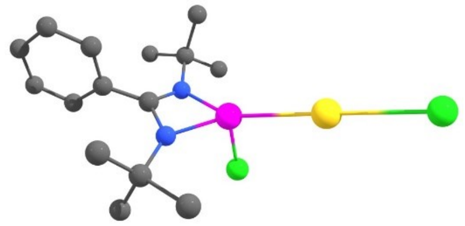

Roesky-AuCl

E = -2038.71458752

N	-2.158328000	10.651523000	2.130919000
Si	-0.989607000	9.198083000	2.221679000
C	-1.261080000	11.452701000	2.709986000
C	-3.213417000	10.923869000	1.129872000
N	-0.364107000	10.653956000	3.286249000
Cl	-2.032780000	8.157364000	3.792686000
C	-1.323095000	12.919469000	2.778776000
C	-4.089866000	12.090957000	1.524728000
C	-2.594099000	11.057661000	-0.219412000
C	-4.120286000	9.650279000	1.185366000
C	0.701217000	10.927334000	4.274065000
C	-0.617860000	13.516705000	1.735501000
C	-1.948888000	13.709104000	3.739204000
H	-3.546129000	12.942574000	1.529859000
H	-4.469289000	11.931164000	2.447994000
H	-4.847003000	12.180958000	0.861933000
H	-2.059852000	10.226447000	-0.430972000
H	-1.982624000	11.862184000	-0.234426000
H	-3.319118000	11.176368000	-0.913296000
H	-4.799374000	9.689703000	0.438184000
H	-4.599931000	9.618485000	2.074581000
H	-3.550517000	8.823379000	1.080174000
C	1.584876000	12.084060000	3.862858000
C	0.090101000	11.077428000	5.624238000
C	1.598013000	9.646644000	4.223249000
H	-0.173933000	12.955996000	1.053518000
C	-0.540612000	14.903066000	1.653164000
C	-1.870924000	15.095256000	3.654399000
H	-2.448593000	13.284932000	4.477872000
H	1.047825000	12.940018000	3.854342000
H	1.957033000	11.913100000	2.939170000
H	2.347157000	12.174171000	4.520364000
H	-0.448940000	10.251924000	5.845214000
H	-0.515342000	11.886589000	5.636224000
H	0.820383000	11.196790000	6.313066000
H	2.282689000	9.687079000	4.965840000
H	2.072019000	9.603303000	3.331819000
H	1.021655000	8.825994000	4.340719000
H	-0.040358000	15.327617000	0.913770000
C	-1.167416000	15.692196000	2.613475000
H	-2.315343000	15.655777000	4.337227000
H	-1.111163000	16.677858000	2.555020000
Au	-0.038499000	7.159171000	1.148699000
Cl	0.844866000	5.244518000	0.165589000

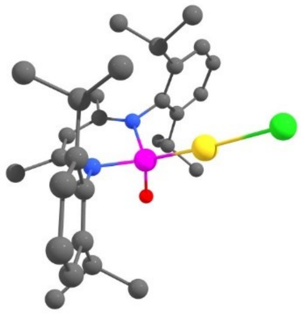

NacNac-SiOH-AuCl

E = -2197.88168406

Si	3.558419000	4.999660000	2.366204000
N	3.802686000	3.654808000	1.156868000
N	3.734646000	6.377238000	1.176677000
O	5.066462000	5.044532000	3.112408000
C	4.543167000	3.797172000	0.051592000
C	3.229549000	2.373598000	1.478279000
C	4.458823000	6.281794000	0.057456000
C	3.190979000	7.650233000	1.578387000
H	5.014956000	5.014322000	4.072699000
C	4.954891000	2.575594000	-0.707206000
C	4.924141000	5.053161000	-0.429161000
C	3.939788000	1.468661000	2.289290000
C	1.947096000	2.072198000	0.977514000
C	4.776569000	7.522410000	-0.716395000
C	4.004396000	8.570064000	2.267789000
C	1.839141000	7.927683000	1.282742000
H	5.594478000	1.942026000	-0.074557000
H	5.501736000	2.843670000	-1.618330000
H	4.081628000	1.963687000	-0.972392000
H	5.538138000	5.076330000	-1.327293000
C	3.349197000	0.231245000	2.562767000
C	5.282293000	1.808627000	2.905459000
C	1.404640000	0.820061000	1.275995000
C	1.156499000	3.052007000	0.132610000
H	5.503423000	8.133028000	-0.160462000
H	5.200202000	7.274213000	-1.696318000
H	3.882049000	8.146502000	-0.848188000
C	3.452279000	9.811682000	2.599241000
C	5.413911000	8.243186000	2.723302000
C	1.338988000	9.181890000	1.639189000
C	0.946419000	6.904126000	0.606159000
H	3.880588000	-0.485957000	3.192676000
C	2.098047000	-0.097091000	2.056701000
H	5.612059000	2.772208000	2.491822000
C	6.354862000	0.767510000	2.590297000
C	5.130426000	2.003627000	4.415721000
H	0.412891000	0.563769000	0.897141000
H	1.681494000	4.017787000	0.173762000
C	-0.248672000	3.273269000	0.687753000
C	1.108090000	2.618525000	-1.333454000
H	4.064613000	10.542158000	3.133010000
C	2.138574000	10.123011000	2.278365000
H	5.697529000	7.270967000	2.296124000
C	6.440956000	9.283063000	2.277667000
C	5.438648000	8.077662000	4.244862000
H	0.297290000	9.422456000	1.424423000

H	1.296658000	5.915395000	0.945199000
C	-0.511781000	7.015489000	1.036861000
C	1.078347000	6.943369000	-0.916838000
H	1.653725000	-1.069034000	2.283021000
H	6.117102000	-0.212757000	3.032021000
H	7.327456000	1.083937000	2.997625000
H	6.475877000	0.618830000	1.506098000
H	4.334494000	2.727802000	4.646540000
H	6.072403000	2.363085000	4.859163000
H	4.862086000	1.058793000	4.914447000
H	-0.848778000	2.350767000	0.658513000
H	-0.779917000	4.024389000	0.084180000
H	-0.220619000	3.627961000	1.729444000
H	2.113518000	2.520299000	-1.769293000
H	0.554561000	3.356213000	-1.935179000
H	0.600032000	1.647152000	-1.443263000
H	1.725029000	11.097685000	2.547402000
H	6.255000000	10.264720000	2.740480000
H	7.454509000	8.969562000	2.571368000
H	6.437519000	9.430891000	1.186839000
H	4.676984000	7.358262000	4.578071000
H	6.425066000	7.722166000	4.581358000
H	5.231379000	9.033275000	4.752045000
H	-0.981222000	7.945328000	0.679348000
H	-1.090529000	6.181625000	0.615294000
H	-0.610094000	6.964765000	2.131584000
H	2.102403000	6.724552000	-1.252332000
H	0.415441000	6.194011000	-1.377314000
H	0.795132000	7.932145000	-1.312210000
Au	1.653140000	4.945117000	3.581051000
Cl	-0.380759000	4.931976000	4.734005000

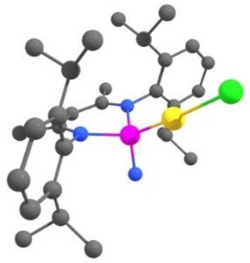

NacNac-SiNH$_2$-AuCl

E = -2178.02526035

Si	1.647495000	5.002247000	6.959501000
N	1.897237000	6.372592000	5.744168000
N	1.898128000	3.632522000	5.743734000
N	3.100197000	5.002891000	7.886750000
C	2.638516000	6.248659000	4.640587000
C	1.343288000	7.654588000	6.102934000
C	2.639741000	3.757103000	4.640446000
C	1.343599000	2.350470000	6.101299000
H	4.046002000	5.003369000	7.525155000
H	3.063708000	5.001856000	8.897226000
C	3.025367000	7.473875000	3.871213000
C	3.069491000	5.003095000	4.165623000
C	0.011898000	7.934097000	5.725003000
C	2.113847000	8.588092000	6.823450000
C	3.028164000	2.532164000	3.871429000

C	0.012950000	2.071079000	5.720728000
C	2.112702000	1.416885000	6.823205000
H	2.176607000	8.161711000	3.760834000
H	3.415164000	7.207871000	2.881802000
H	3.805048000	8.024415000	4.419120000
H	3.687297000	5.003456000	3.269583000
C	-0.518744000	9.181904000	6.057542000
C	-0.824927000	6.932540000	4.954512000
C	1.532719000	9.822798000	7.131218000
C	3.520993000	8.304847000	7.312431000
H	2.179920000	1.843867000	3.759958000
H	3.419053000	2.798395000	2.882511000
H	3.807425000	1.982012000	4.420348000
C	-0.518466000	0.823321000	6.052186000
C	-0.822231000	3.072628000	4.948451000
C	1.530870000	0.182228000	7.129844000
C	3.518950000	1.700131000	7.314696000
H	-1.546421000	9.416285000	5.776329000
C	0.233123000	10.123932000	6.749646000
H	-0.414854000	5.938792000	5.185649000
C	-0.697824000	7.140776000	3.444352000
C	-2.286899000	6.921212000	5.388061000
H	2.113278000	10.559475000	7.691413000
H	3.822767000	7.326480000	6.912426000
C	3.538989000	8.192445000	8.837787000
C	4.530144000	9.350186000	6.837009000
H	-1.545606000	0.589014000	5.768904000
C	0.231968000	-0.118766000	6.745773000
H	-0.410684000	4.066168000	5.177788000
C	-0.695218000	2.861476000	3.438683000
C	-2.284257000	3.086737000	5.381784000
H	2.110288000	-0.554527000	7.691117000
H	3.821311000	2.678589000	6.915361000
C	3.534240000	1.812364000	8.840092000
C	4.528984000	0.654981000	6.840768000
H	-0.202534000	11.093069000	7.003180000
H	-1.049226000	8.144188000	3.154585000
H	-1.306887000	6.400323000	2.902501000
H	0.339607000	7.033610000	3.095644000
H	-2.800840000	7.862337000	5.137806000
H	-2.386229000	6.740332000	6.469078000
H	-2.824343000	6.116406000	4.865752000
H	3.292688000	9.157409000	9.308060000
H	4.536244000	7.890084000	9.195241000
H	2.806375000	7.451974000	9.185956000
H	4.526164000	9.466055000	5.742571000
H	5.549305000	9.069074000	7.144806000
H	4.320098000	10.340749000	7.269172000
H	-0.204271000	-1.087861000	6.998465000
H	-1.048156000	1.858098000	3.150667000
H	-1.302991000	3.601914000	2.895370000
H	0.342461000	2.966312000	3.090002000
H	-2.799298000	2.145685000	5.133542000
H	-2.383525000	3.269969000	6.462419000
H	-2.820659000	3.891020000	4.857574000
H	3.287407000	0.847281000	9.309843000
H	4.530760000	2.115012000	9.199344000
H	2.800725000	2.552506000	9.187112000

H	4.527001000	0.539616000	5.746268000
H	5.547575000	0.935974000	7.150556000
H	4.318140000	-0.335767000	7.272122000
Au	-0.339457000	5.000985000	8.045829000
Cl	-2.452759000	4.999899000	9.057095000

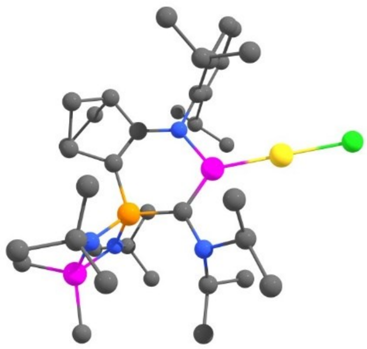

D-AuCl

E = -3141.37195577

P	4.989676000	17.328753000	4.277846000
N	4.277063000	18.800697000	3.755730000
N	6.325402000	18.264252000	4.731943000
C	4.189990000	16.344742000	5.476375000
C	5.375628000	16.274744000	2.939402000
Si	5.675964000	19.801645000	4.153768000
C	2.997080000	19.080941000	3.090988000
C	7.528879000	17.887177000	5.481061000
N	4.128721000	16.815808000	6.829554000
Si	3.339203000	14.856051000	4.963589000
C	4.838828000	15.048076000	2.646249000
C	6.348279000	16.596985000	1.813155000
C	6.593106000	20.515697000	2.687839000
C	5.402921000	21.131627000	5.431662000
C	2.023678000	19.747632000	4.069660000
C	3.250809000	20.039978000	1.925673000
C	2.361654000	17.796295000	2.560427000
C	7.853941000	16.411950000	5.250115000
C	7.304777000	18.152168000	6.971348000
C	8.689645000	18.740943000	4.971405000
C	3.153896000	17.914268000	6.988570000
C	4.161809000	15.759342000	7.863336000
N	3.889375000	14.370837000	3.345450000
C	5.449751000	14.598645000	1.334355000
C	5.524669000	16.896687000	0.537904000
H	7.101615000	17.355625000	2.064074000
C	6.860549000	15.184779000	1.490271000
H	6.701350000	19.767132000	1.889608000
H	7.598877000	20.848868000	2.987345000
H	6.067138000	21.389173000	2.273201000
H	4.825719000	21.965702000	5.001109000
H	6.364068000	21.544720000	5.777300000
H	4.857854000	20.741400000	6.301445000
H	1.710110000	19.047683000	4.855456000
H	1.118514000	20.088398000	3.544137000
H	2.488841000	20.621247000	4.551299000
H	3.639313000	21.006339000	2.285392000
H	2.316403000	20.244546000	1.382026000

H	3.980082000	19.620459000	1.217880000
H	2.997275000	17.311454000	1.808208000
H	1.390191000	18.023097000	2.097371000
H	2.184676000	17.078650000	3.375351000
H	7.025724000	15.767067000	5.574129000
H	8.750187000	16.130741000	5.822560000
H	8.046495000	16.213053000	4.185956000
H	7.155214000	19.228371000	7.151893000
H	8.167037000	17.822692000	7.571492000
H	6.399424000	17.624828000	7.303187000
H	8.860195000	18.575074000	3.896893000
H	9.615040000	18.493357000	5.512244000
H	8.494554000	19.814061000	5.131821000
H	3.194429000	18.415426000	6.013611000
C	1.718914000	17.432205000	7.176641000
C	3.543871000	18.996089000	7.994189000
H	3.250826000	15.128876000	7.789769000
C	5.365599000	14.839235000	7.690754000
C	4.195287000	16.312851000	9.281896000
C	3.488212000	13.064445000	2.894372000
H	5.354317000	13.527747000	1.119987000
C	4.864092000	15.530063000	0.235335000
H	6.185026000	17.213627000	-0.284250000
H	4.798151000	17.702839000	0.703712000
H	7.452456000	15.132592000	0.562475000
H	7.424145000	14.731058000	2.318674000
H	1.442975000	16.698111000	6.402581000
H	1.014481000	18.275865000	7.109356000
H	1.565127000	16.952690000	8.154780000
H	3.289676000	18.751745000	9.032792000
H	3.007512000	19.925484000	7.744602000
H	4.622917000	19.197702000	7.945997000
H	5.412353000	14.376115000	6.696441000
H	5.307263000	14.024347000	8.426436000
H	6.304557000	15.389022000	7.855268000
H	5.037168000	17.008034000	9.423152000
H	4.325594000	15.474852000	9.981690000
H	3.267637000	16.823247000	9.565740000
C	2.348419000	12.939324000	2.079454000
C	4.230200000	11.943608000	3.313559000
H	5.143736000	15.154243000	-0.760038000
H	3.766440000	15.561235000	0.271994000
C	1.961282000	11.655612000	1.686181000
C	1.523597000	14.146636000	1.682871000
C	3.806238000	10.681496000	2.889432000
C	5.448052000	12.068704000	4.209044000
H	1.072812000	11.529895000	1.063571000
C	2.682516000	10.535633000	2.084482000
H	2.141884000	15.035480000	1.875568000
C	0.278146000	14.260016000	2.563776000
C	1.158223000	14.153447000	0.200726000
H	4.360806000	9.795165000	3.206831000
H	5.581138000	13.136100000	4.446129000
C	6.718679000	11.606627000	3.495262000
C	5.247508000	11.330082000	5.532130000
H	2.360488000	9.539657000	1.771946000
H	-0.389212000	13.396650000	2.414071000
H	-0.287497000	15.175086000	2.326428000

H	0.541898000	14.281118000	3.632859000
H	0.459543000	13.340997000	-0.052598000
H	2.047525000	14.039583000	-0.437797000
H	0.663402000	15.100274000	-0.066208000
H	6.669295000	10.535367000	3.243675000
H	7.599805000	11.756828000	4.138577000
H	6.882126000	12.161110000	2.558915000
H	4.333325000	11.670916000	6.042543000
H	6.103367000	11.501786000	6.203497000
H	5.156176000	10.243618000	5.377397000
Au	1.983564000	13.407563000	6.046360000
Cl	0.611532000	11.916718000	7.191836000

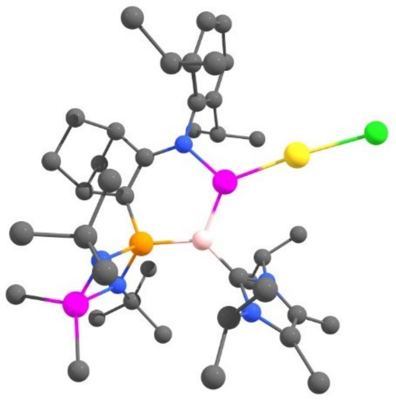

BoraylideSi-AuCl

E = -3376.64599352

Si	-3.126387000	12.969364000	8.048657000
B	-2.535792000	11.568089000	6.930543000
N	-3.412784000	14.483824000	7.127987000
P	-2.791530000	11.862328000	5.123573000
C	-1.967122000	10.322105000	7.692627000
C	-3.106424000	14.599790000	5.806418000
C	-3.705209000	15.677095000	7.873483000
N	-4.060567000	11.118124000	4.170320000
N	-1.737279000	11.165610000	3.954800000
C	-2.812468000	13.606649000	4.898181000
N	-2.500558000	9.078732000	7.774058000
N	-0.884728000	10.310338000	8.521712000
C	-3.078403000	15.913317000	5.045092000
C	-5.045430000	16.083189000	8.020556000
C	-2.649171000	16.399747000	8.461583000
Si	-2.989214000	10.455938000	2.947264000
C	-5.521538000	11.226868000	4.250339000
C	-0.285460000	11.016013000	4.002981000
C	-2.582613000	14.309473000	3.564724000
C	-1.785011000	8.293791000	8.662189000
C	-3.799098000	8.774095000	7.170788000
C	-0.761737000	9.072244000	9.133041000
C	0.041560000	11.445100000	8.673760000
H	-2.995693000	16.809375000	5.671020000
C	-4.306719000	15.914104000	4.094644000
C	-1.944162000	15.617069000	4.053883000
C	-5.309807000	17.236095000	8.763768000
C	-6.178759000	15.262741000	7.441470000
C	-2.962772000	17.550418000	9.190700000

C	-1.201526000	15.965666000	8.334056000
C	-3.107933000	11.191104000	1.227604000
C	-2.979394000	8.589618000	2.796369000
C	-6.126380000	9.916524000	4.765352000
C	-6.089136000	11.485807000	2.851584000
C	-5.936902000	12.360665000	5.185783000
C	0.355308000	12.238790000	4.661479000
C	0.082153000	9.737977000	4.766637000
C	0.227145000	10.905367000	2.566271000
C	-3.940998000	14.837374000	3.045569000
H	-2.012354000	13.717920000	2.836063000
C	-2.107502000	6.885507000	9.011738000
H	-3.916357000	9.571724000	6.422562000
C	-4.901220000	8.932886000	8.208394000
C	-3.825787000	7.442661000	6.437416000
C	0.261532000	8.713558000	10.150105000
H	-0.442817000	12.225972000	8.066659000
C	0.125242000	11.951272000	10.104206000
C	1.406483000	11.151939000	8.065389000
H	-5.238293000	15.695606000	4.634823000
H	-4.425094000	16.906859000	3.634972000
H	-1.847895000	16.383967000	3.267789000
H	-0.973156000	15.462524000	4.547076000
H	-6.343197000	17.563187000	8.897391000
C	-4.279144000	17.968503000	9.342367000
H	-5.740873000	14.624205000	6.662148000
C	-7.262822000	16.112606000	6.785175000
C	-6.766310000	14.335982000	8.507205000
H	-2.158515000	18.124343000	9.657562000
H	-1.189316000	15.010071000	7.785371000
C	-0.381280000	16.970372000	7.524326000
C	-0.575196000	15.714201000	9.704923000
H	-3.211049000	12.284774000	1.274864000
H	-3.964858000	10.785910000	0.668222000
H	-2.198319000	10.956443000	0.652603000
H	-2.147897000	8.243931000	2.161543000
H	-3.913251000	8.236884000	2.329166000
H	-2.887864000	8.113909000	3.782178000
H	-5.768630000	9.061267000	4.171612000
H	-7.225309000	9.941040000	4.702370000
H	-5.857680000	9.751896000	5.815367000
H	-5.659545000	12.392768000	2.404607000
H	-7.182096000	11.606964000	2.895694000
H	-5.879772000	10.640453000	2.176301000
H	-5.508038000	12.220215000	6.189494000
H	-7.032566000	12.388293000	5.282707000
H	-5.599067000	13.333505000	4.807724000
H	0.124626000	13.149944000	4.090732000
H	1.448853000	12.121849000	4.704270000
H	-0.024395000	12.383581000	5.682932000
H	-0.319675000	9.770277000	5.788649000
H	1.173497000	9.600244000	4.824963000
H	-0.345906000	8.856432000	4.265223000
H	-0.204561000	10.029333000	2.055017000
H	1.321087000	10.787368000	2.552881000
H	-0.032187000	11.806026000	1.990180000
H	-4.685454000	14.035043000	2.973329000
H	-3.831237000	15.276122000	2.041077000

H	-1.467991000	6.556613000	9.839842000
H	-1.941397000	6.198165000	8.168601000
H	-3.150099000	6.768523000	9.340613000
H	-4.789211000	8.216914000	9.036023000
H	-5.885511000	8.759625000	7.749621000
H	-4.884599000	9.950964000	8.626500000
H	-2.911991000	7.300569000	5.842235000
H	-4.682512000	7.434324000	5.749251000
H	-3.940716000	6.583475000	7.111375000
H	0.235554000	7.632594000	10.334596000
H	0.071720000	9.219788000	11.108579000
H	1.281248000	8.967093000	9.832049000
H	-0.876887000	12.107307000	10.532211000
H	0.637023000	12.923892000	10.104922000
H	0.695360000	11.280657000	10.761506000
H	1.993497000	10.436949000	8.659998000
H	1.983024000	12.087467000	8.020210000
H	1.315414000	10.761924000	7.044118000
H	-4.504704000	18.866203000	9.922768000
H	-7.992564000	15.467516000	6.271123000
H	-6.840815000	16.809257000	6.044577000
H	-7.822938000	16.708139000	7.523101000
H	-5.987813000	13.699262000	8.955930000
H	-7.542414000	13.684284000	8.074598000
H	-7.222685000	14.917394000	9.324143000
H	-0.807035000	17.129672000	6.522788000
H	0.654055000	16.615026000	7.400228000
H	-0.338685000	17.949160000	8.028302000
H	0.453197000	15.334174000	9.594608000
H	-1.160865000	14.979260000	10.278325000
H	-0.521666000	16.638714000	10.300859000
Au	-3.387126000	13.060721000	10.306475000
Cl	-3.502679000	13.010180000	12.651939000

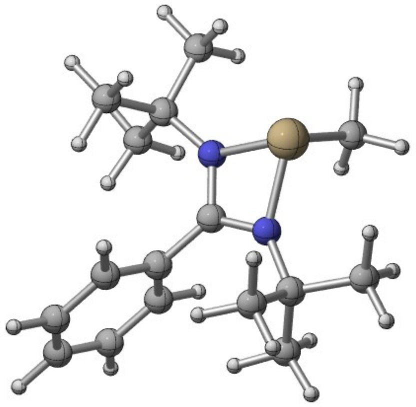

AMeSi

E = -1023.69100089

Si	28.02418500	8.31290600	9.23683800
N	27.50886700	6.47597900	9.11067500
N	26.17781500	8.05606900	9.66214500
C	26.29585600	6.73035700	9.59953000
C	25.30046700	5.72828600	10.06577000
C	25.32003300	5.27339800	11.38812300
C	24.33601000	5.23127100	9.18233400
H	26.07186400	5.65984500	12.07994200

C	24.38648400	4.33299800	11.82053500
C	23.40465500	4.29206700	9.61604900
H	24.32171400	5.58712800	8.14980700
H	24.40873100	3.98296100	12.85515000
C	23.42830700	3.84121300	10.93595300
H	22.65526500	3.90912900	8.91954000
H	22.69709500	3.10405200	11.27567700
C	27.82871200	8.79487100	7.38471800
H	27.66308800	9.88179300	7.29516900
H	28.76273400	8.57664200	6.83988900
H	26.99856900	8.26998500	6.88435000
C	28.24637100	5.22989700	8.93638100
C	28.71478600	4.69383200	10.29375600
C	27.41676900	4.17118100	8.20660800
C	29.46564300	5.58134600	8.08231800
H	29.28972700	5.46531600	10.82762900
H	27.85805100	4.40172300	10.91813600
H	29.35498600	3.80761500	10.16254600
H	27.00931800	4.57886700	7.26912200
H	28.05322700	3.30828000	7.95832700
H	26.58205000	3.80559800	8.81928700
H	30.11440100	4.70290200	7.95222000
H	29.15466300	5.93716300	7.08894200
H	30.05779700	6.37605600	8.56473500
C	25.14310700	8.91486300	10.22693300
C	23.74835600	8.53564600	9.72431200
C	25.18666200	8.86567000	11.75818300
C	25.47264900	10.33219200	9.75561800
H	23.73418900	8.48836200	8.62492000
H	23.41663400	7.56621600	10.11922800
H	23.01945600	9.29525000	10.04529600
H	26.19675900	9.11495500	12.11642500
H	24.47387100	9.58449000	12.19140200
H	24.92214200	7.86409300	12.12709300
H	24.76934800	11.05835300	10.18867200
H	26.49165500	10.61451200	10.06680900
H	25.41515900	10.40027200	8.65914300

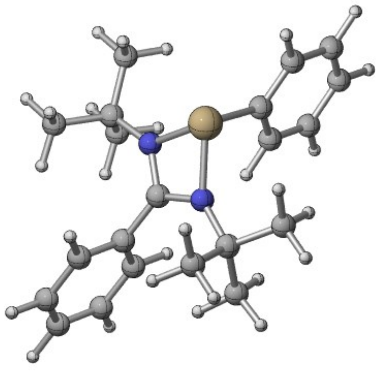

APhSi

E = -1215.27958758

Si	27.65783200	8.52431400	9.19392100
N	27.49056400	6.62868300	9.27806800
N	25.86606200	7.98474500	9.56227400
C	26.21606500	6.70513600	9.66437500
C	25.38224000	5.59447800	10.19595400
C	25.36731200	5.32381200	11.56841700
C	24.61244600	4.80852700	9.33208400
H	25.96190700	5.94173800	12.24503700
C	24.59467800	4.27798900	12.06859300

C	23.83900500	3.76501200	9.83449600
H	24.62808700	5.01560900	8.25971900
H	24.58915500	4.07295200	13.14158000
C	23.82970700	3.49749500	11.20294400
H	23.24142400	3.15585700	9.15256100
H	23.22368400	2.67801200	11.59615800
C	27.48452200	8.74437900	7.28474700
C	28.07709200	9.86139500	6.68026500
C	26.77755700	7.85628100	6.45823400
C	27.96569400	10.09260800	5.30703000
H	28.63899800	10.56860600	7.30034800
C	26.66270300	8.07419500	5.08794300
H	26.30977300	6.97044000	6.90131100
C	27.25766400	9.19792400	4.50861800
H	28.43515800	10.97271400	4.85940700
H	26.10828300	7.36790300	4.46353200
H	27.16905900	9.37212900	3.43310600
C	28.46326900	5.54197500	9.32269800
C	28.89570600	5.26785700	10.76667900
C	27.91374300	4.26620000	8.68098300
C	29.66487600	6.02934100	8.51132700
H	29.26731000	6.19246300	11.23351200
H	28.05475600	4.88750800	11.36470300
H	29.69870100	4.51510000	10.79555900
H	27.53315600	4.47668000	7.67002400
H	28.71718000	3.51901300	8.59578200
H	27.10409200	3.82074800	9.27429900
H	30.47089900	5.28129800	8.52536900
H	29.37684500	6.22020800	7.46704900
H	30.06064300	6.96746100	8.93322300
C	24.59457700	8.67110400	9.76407600
C	23.52932900	8.11109800	8.81691900
C	24.12760300	8.56493800	11.21871000
C	24.84675600	10.13918100	9.41811500
H	23.88722700	8.15129200	7.77724600
H	23.27842400	7.07010100	9.06438900
H	22.60558600	8.70522500	8.88966400
H	24.91369300	8.92646900	11.89876200
H	23.22643000	9.17752200	11.37528000
H	23.88100000	7.52861900	11.48895400
H	23.92812700	10.72855300	9.55236900
H	25.62818600	10.56248300	10.06825500
H	25.17619100	10.24077800	8.37297500

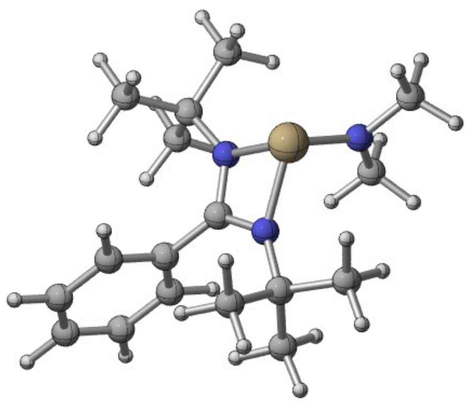

A(NMe$_2$)Si

E = -1118.30077822

Si	28.31282800	7.97150200	9.19350400
N	27.41592800	6.28766300	8.90376400

N	26.46501600	8.00512400	9.74907100
C	26.34097900	6.69105500	9.57699900
C	25.26309900	5.82443100	10.12477300
C	25.39022100	5.26383100	11.40003000
C	24.11435900	5.56219700	9.37065500
H	26.28639000	5.46728700	11.99053800
C	24.38075400	4.45183600	11.91306400
C	23.10694400	4.75027500	9.88535600
H	24.01462500	5.99966100	8.37480800
H	24.48836600	4.01824500	12.90998600
C	23.23822700	4.19372700	11.15726800
H	22.21355000	4.55092000	9.28918900
H	22.44749900	3.55686000	11.56046600
N	28.22151600	8.70611800	7.59887000
C	27.13402700	8.56149400	6.67396300
H	27.45916400	8.10411200	5.71670300
H	26.34777900	7.92045500	7.09885000
H	26.67052900	9.53723400	6.42030900
C	29.29279900	9.53471000	7.11786200
H	29.74761400	9.13557100	6.18808500
H	28.95644800	10.56769300	6.89319700
H	30.08876300	9.60087900	7.87655400
C	27.87292700	4.96346000	8.49833800
C	28.29609800	4.13066000	9.71290200
C	26.79678700	4.22713300	7.69695200
C	29.08791800	5.19922300	7.59857000
H	29.04133800	4.68061400	10.30714700
H	27.43537900	3.90001100	10.35690400
H	28.74213300	3.17657700	9.39166100
H	26.44952400	4.85023200	6.85874800
H	27.20960100	3.29411700	7.28411300
H	25.93070200	3.96363700	8.31896300
H	29.50203100	4.24111200	7.25170200
H	28.81011600	5.80373200	6.72276900
H	29.87624000	5.73888300	8.14629200
C	25.65296600	8.97438300	10.47570400
C	24.19401700	8.93134200	10.01505800
C	25.74093000	8.74365900	11.98790400
C	26.24011900	10.34644600	10.13815800
H	24.13439900	9.03482500	8.92091500
H	23.69991600	7.99428900	10.30526800
H	23.63259000	9.76138300	10.47026200
H	26.79356500	8.73634100	12.30862100
H	25.21630900	9.54382000	12.53282400
H	25.28149800	7.78594400	12.27156000
H	25.68476100	11.14139800	10.65718800
H	27.29535400	10.40246300	10.44804000
H	26.19534800	10.53145200	9.05492300

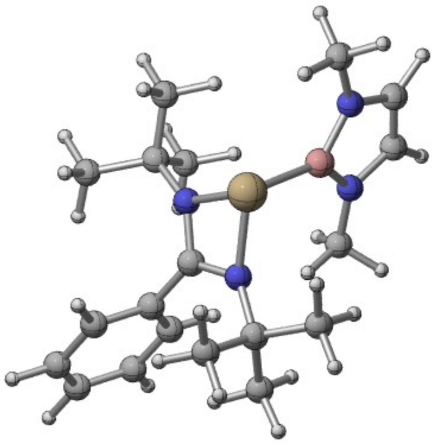

A(NHB)Si

E = -1275.23684263

Si	27.72301400	8.98657500	9.28183400
N	27.80377700	7.08067000	9.38422000
N	25.98675800	8.20046000	9.36050900
C	26.47607200	6.97508600	9.53006700
C	25.71098900	5.74901000	9.87053000
C	25.36053500	5.48009400	11.19835500
C	25.33247200	4.85138300	8.86625700
H	25.64507000	6.18670500	11.98120400
C	24.64777000	4.32663700	11.51670200
C	24.61465600	3.70116400	9.18552800
H	25.61112700	5.05740500	7.83042700
H	24.38044200	4.12325100	12.55627800
C	24.27314700	3.43595400	10.51110600
H	24.32236400	3.00650000	8.39469200
H	23.71233300	2.53242500	10.76148000
C	27.78249300	9.24091600	4.95915800
C	27.96572500	10.53943000	5.31725200
H	27.71874500	8.81518600	3.95843600
H	28.08042200	11.41143600	4.67477900
C	28.18171900	11.88213400	7.37655100
H	29.09748900	12.39515600	7.03843000
H	27.32891800	12.56321400	7.21191800
H	28.27006500	11.69884700	8.45694800
C	27.47434400	7.04362700	6.03239000
H	26.46153300	6.79936500	5.66495200
H	28.20277900	6.55620800	5.36270500
H	27.59197300	6.62121500	7.03759800
B	27.82172700	9.31007400	7.26105700
N	28.00196900	10.62683800	6.69687500
N	27.69230700	8.46207400	6.09951400
C	24.63268800	8.67537800	9.09947500
C	24.71359200	10.20208200	9.07617700
C	24.16747000	8.17257100	7.72872600
C	23.64942500	8.23994300	10.18896800
H	25.05977800	10.58844100	10.04625300
H	25.41824400	10.53671900	8.29825500
H	23.72774600	10.63656500	8.85532800
H	24.09015600	7.07532800	7.71931500
H	23.17680500	8.58335500	7.47982200
H	24.88151200	8.48266100	6.95105800
H	22.67460300	8.72487900	10.02716600
H	23.48826200	7.15350100	10.18667600
H	24.02039500	8.53500000	11.18206800
C	28.88498600	6.19308500	9.80716100
C	28.99707700	6.19131700	11.33531200

C	28.69732100	4.76414000	9.29281400
C	30.15891400	6.78139900	9.19925200
H	29.10909000	7.22123400	11.70621700
H	28.09781500	5.75194400	11.79207500
H	29.86650000	5.60073500	11.66376800
H	28.54373100	4.75505200	8.20308300
H	29.59966300	4.17380500	9.51289800
H	27.84446100	4.26113200	9.76737100
H	31.04228000	6.20302800	9.50715900
H	30.10259700	6.78080800	8.10041000
H	30.29598400	7.82272900	9.53432000

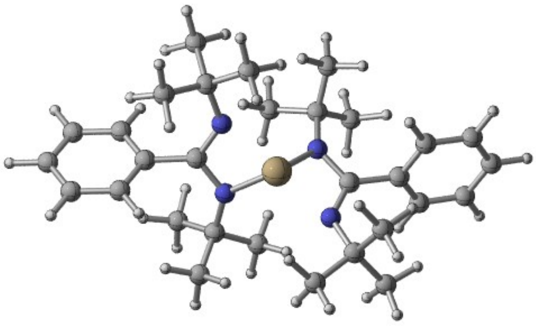

Tacke's bis(amidinato)silylene

E = -1678.23926798

C	-0.45575900	1.88833200	1.80675900
C	-1.29671000	-2.23855800	-0.85837100
C	-3.47572200	0.90168700	0.13909800
C	-3.36873300	-0.87750700	1.76772700
C	-4.84738900	1.01169200	0.34989000
C	-4.73909300	-0.75960800	1.98643400
C	-1.26781000	-0.18501300	0.59416200
C	-2.72691600	-0.04567400	0.84517100
C	-5.48144600	0.18350000	1.27629100
C	-1.57580700	-3.46580200	0.01710600
C	-0.26959900	-2.61461200	-1.93213200
C	-0.92332400	3.15096600	1.07568300
C	-1.40784900	1.58951500	2.97114300
H	-2.97709700	1.53597000	-0.59676900
H	-2.78110900	-1.60649600	2.32996000
H	-5.42689000	1.74615100	-0.21450700
H	-5.23086200	-1.40711900	2.71614700
H	-2.33041700	-3.23945600	0.78387700
H	-0.08752700	-1.76711200	-2.60754400
H	-0.27751700	3.35530800	0.21149600
H	-1.16842300	0.61992200	3.43385200
H	-6.55728200	0.27291000	1.44434400
H	-0.65952500	-3.80146500	0.52428100
H	0.69602900	-2.88430600	-1.48264100
H	-1.95615300	3.03993000	0.71724300
H	-2.46366000	1.58165100	2.67521700
H	-1.95519500	-4.29847200	-0.59546700
H	-0.63175100	-3.47276600	-2.51849900
H	-0.89149900	4.02276300	1.74762300
H	-1.28497600	2.36975400	3.73728300
N	-0.34984700	0.76178200	0.86291500
N	-0.68696900	-1.18791300	-0.03728400
C	3.21008400	2.28476300	-0.96442600
C	2.34092500	-1.82511500	1.69076300

C	5.48266800	0.65639800	1.46630000
C	5.23503200	-0.99646600	-0.27554600
C	6.84300300	0.36947100	1.54360500
C	6.59831300	-1.27312600	-0.20820300
C	3.20442900	0.23592100	0.48058400
C	4.66706200	-0.02884500	0.56090300
C	7.40385500	-0.59447000	0.70594700
C	2.44498500	-3.12831600	0.89606200
C	1.06512700	-1.84627100	2.53876700
C	2.06110400	3.21344200	-1.36330700
C	4.30068100	3.13021700	-0.29935400
H	5.03812800	1.40471100	2.12587300
H	4.59606400	-1.52756400	-0.98502200
H	7.47018000	0.90094500	2.26318800
H	7.03415500	-2.02496700	-0.87045700
H	3.36325100	-3.14601400	0.29122300
H	0.99497300	-0.93782400	3.15424800
H	1.22326500	2.62706100	-1.77098700
H	5.22052700	2.55780600	-0.12480400
H	8.47186300	-0.81696500	0.76555800
H	1.58450200	-3.23423200	0.22299800
H	0.17823800	-1.90360400	1.89699200
H	1.70123100	3.77908600	-0.49130300
H	3.94751000	3.53017700	0.66360200
H	2.46199200	-3.99728000	1.57189000
H	1.07616300	-2.71781400	3.21091800
H	2.39258900	3.93439800	-2.12539800
H	4.55542600	3.98204900	-0.94894700
N	2.62759300	1.29792500	-0.04854000
N	2.26039700	-0.68223200	0.76437200
Si	0.93741900	0.08629900	-0.36879300
C	-2.58288200	-1.82325400	-1.58395800
H	-3.43789000	-1.70384800	-0.90664000
H	-2.43312300	-0.87767300	-2.12654900
H	-2.84845300	-2.59746500	-2.32035900
C	0.92736400	2.13882100	2.41215800
H	1.26670400	1.25859800	2.97795300
H	1.66999300	2.33431900	1.62925700
H	0.88381800	2.99654800	3.09994500
C	3.75973800	1.60939500	-2.22609500
H	4.61512400	0.95970900	-1.99191400
H	2.97280200	0.99913700	-2.69434600
H	4.10026300	2.36527400	-2.95137100
C	3.52124000	-1.73448400	2.66370800
H	4.49394100	-1.89714200	2.18436700
H	3.54249900	-0.75919800	3.17277500
H	3.39610200	-2.51152600	3.43264500

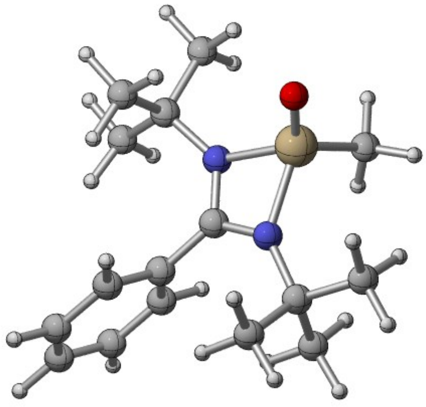

AMeSiO
E = -1098.95023378

N	27.56482100	6.49924200	9.29211900
N	26.07927600	8.03814400	9.54276300
C	26.30584200	6.72990900	9.65571700
C	25.31300600	5.72094800	10.09937400
C	25.32152800	5.25551000	11.41762200
C	24.34214900	5.26017200	9.20477600
H	26.07997100	5.61903900	12.11418200
C	24.36123600	4.33744800	11.83687600
C	23.39092600	4.33490700	9.62568800
H	24.33239000	5.63558300	8.17917600
H	24.36741900	3.98115100	12.86937000
C	23.39726800	3.87484500	10.94223900
H	22.63613400	3.97521000	8.92285100
H	22.64659400	3.15331200	11.27263500
C	27.81150000	8.60157000	7.16452000
H	27.50541200	9.63785500	6.95243600
H	28.81749700	8.46846600	6.73787800
H	27.11416700	7.92025700	6.65390100
C	28.28618100	5.26155200	8.99834800
C	28.40927700	4.39024000	10.25072100
C	27.60126400	4.48515200	7.87091500
C	29.68331700	5.69618800	8.55276200
H	28.84552100	4.97028200	11.07738000
H	27.43436000	3.99818100	10.57064500
H	29.06570900	3.53094800	10.04656600
H	27.48612600	5.12061400	6.97960500
H	28.20383900	3.60780800	7.59080400
H	26.60757000	4.12685500	8.17632800
H	30.31832300	4.81789100	8.36760900
H	29.63330000	6.28097000	7.62186400
H	30.15780400	6.32146400	9.32399300
C	25.13504300	8.92283100	10.22928200
C	23.68826700	8.44936500	10.10262800
C	25.55293200	9.03341700	11.69955100
C	25.29154100	10.28630700	9.55386100
H	23.41820600	8.29186200	9.04736300
H	23.50502400	7.51548700	10.65080000
H	23.01721200	9.21680300	10.51669700
H	26.61346700	9.32239800	11.76019400
H	24.94199400	9.78501900	12.22276800
H	25.41941900	8.07014200	12.21559000
H	24.63321900	11.02817100	10.02868000
H	26.33153800	10.63725800	9.64734300
H	25.03186800	10.22371000	8.48597400
Si	27.84543600	8.33775600	9.01562000
O	28.75454200	9.14421900	9.97133800

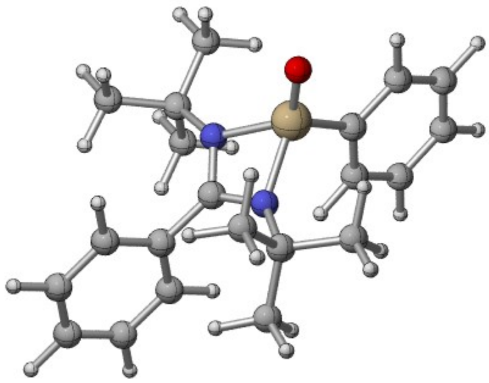

APhSIO

E = -1290.53764102

N	27.48440800	6.54263200	9.22864000
N	25.93563300	7.96947600	9.66242100
C	26.24393000	6.67627100	9.69940500
C	25.37391500	5.57097500	10.16807200
C	25.27178000	5.29221800	11.53418500
C	24.67701000	4.78757500	9.24311100
H	25.81622100	5.90765900	12.25355000
C	24.48011000	4.23189700	11.96953900
C	23.87982000	3.73488400	9.68395400
H	24.76636200	5.00271200	8.17608900
H	24.40702900	4.01381900	13.03727800
C	23.78269000	3.45394600	11.04651500
H	23.33485700	3.12701100	8.95839300
H	23.16070100	2.62439400	11.39032400
C	27.39745700	8.71479700	7.16221600
C	28.00681800	9.85720300	6.62466700
C	26.64441700	7.89485700	6.30883800
C	27.85586200	10.17952000	5.27601500
H	28.60722200	10.49039000	7.28449200
C	26.49288000	8.21062300	4.96048400
H	26.17707400	6.98656200	6.70244200
C	27.09726000	9.35785100	4.44368000
H	28.33511800	11.07454800	4.87104800
H	25.90520300	7.56068600	4.30688800
H	26.97931200	9.60816400	3.38625200
C	28.50268300	5.50752100	9.41336800
C	29.00614900	5.57018400	10.85893800
C	27.98381700	4.11148800	9.07426800
C	29.63721100	5.88250500	8.45833400
H	29.33190800	6.59461800	11.09497700
H	28.20947900	5.28279500	11.56232600
H	29.85265500	4.88214900	11.00630700
H	27.54656700	4.09118200	8.06449500
H	28.82030400	3.39718200	9.09843900
H	27.22624300	3.76271900	9.78898200
H	30.46905600	5.16947500	8.55204300
H	29.28631100	5.87768000	7.41536000
H	30.01574300	6.88968900	8.69413100
C	24.66720100	8.68399800	9.80985900
C	23.65291700	8.21092400	8.76571400
C	24.10672300	8.50972300	11.22273700
C	25.00014200	10.15802200	9.57346700
H	24.06916700	8.31351800	7.75237000
H	23.37008800	7.16062100	8.92813000

H	22.73687800	8.81799100	8.82457000
H	24.86012200	8.79666800	11.97132500
H	23.22404300	9.15295400	11.35669700
H	23.79773900	7.47264900	11.41293000
H	24.10366800	10.77973700	9.71036700
H	25.77821500	10.49631400	10.27406500
H	25.37243400	10.31414600	8.54920700
Si	27.63442400	8.37976100	8.98748400
O	28.66076200	9.19503700	9.80567800

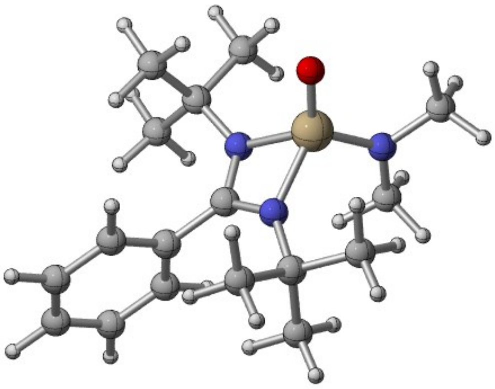

A(NMe$_2$)SiO

E = -1193.55745804

N	27.48555600	6.31574500	9.08322600
N	26.35794700	8.04590600	9.67957500
C	26.33960800	6.71258500	9.62667300
C	25.23252500	5.84342200	10.09011500
C	25.30962600	5.20370500	11.33072800
C	24.09489900	5.68225800	9.29272100
H	26.19781500	5.33473100	11.95234100
C	24.25149100	4.41119300	11.77049700
C	23.04522800	4.88058600	9.73203400
H	24.03565600	6.19074800	8.32796700
H	24.31250700	3.91871800	12.74349700
C	23.12078700	4.24653300	10.97203700
H	22.16031300	4.75415500	9.10439200
H	22.29381000	3.62219600	11.31781600
N	28.13025500	8.61893900	7.43167800
C	27.11266300	8.31572200	6.46852400
H	27.49038800	7.69036200	5.63331200
H	26.27845200	7.77428600	6.93978400
H	26.68892600	9.23454900	6.01734200
C	29.22435900	9.42691100	6.95625900
H	29.77772100	8.93275500	6.13295900
H	28.87468300	10.40704000	6.57659900
H	29.92475600	9.60701500	7.78468100
C	27.95546400	5.02056300	8.59452800
C	28.16223300	4.04748400	9.75774900
C	26.98210600	4.43411200	7.57005200
C	29.30184400	5.30485300	7.92618000
H	28.81063200	4.50105700	10.52190600
H	27.20741200	3.76838300	10.22468600
H	28.64255300	3.12509700	9.39765300
H	26.81442100	5.14420300	6.74626400
H	27.39492000	3.50701400	7.14454300
H	26.01209900	4.19082900	8.02685800
H	29.74842900	4.37365400	7.54893300
H	29.17762400	6.00254600	7.08473200
H	29.99650500	5.76432800	8.64619000

C	25.66717800	8.99117000	10.56095600
C	24.14890200	8.83970400	10.49726500
C	26.18299900	8.80118300	11.99107700
C	26.07202600	10.37716000	10.05569300
H	23.79366600	8.89760600	9.45706800
H	23.80690500	7.88967300	10.92967700
H	23.67654100	9.65471900	11.06586400
H	27.28265500	8.85494000	11.99417400
H	25.78098500	9.58062200	12.65636100
H	25.87416000	7.82323600	12.39129800
H	25.60505700	11.16110000	10.66945700
H	27.16567000	10.49350200	10.11561600
H	25.76117200	10.51684700	9.00941200
Si	28.11983100	8.07907700	9.06407100
O	29.22939900	8.53029400	10.04107600

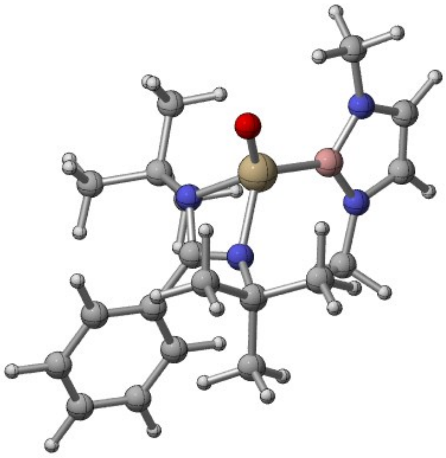

A(NHB)SiO

E = -1350.49768020

N	27.78217500	7.04779200	9.38311100
N	25.95733900	8.18629700	9.41713100
C	26.46237300	6.96709900	9.56351900
C	25.70629900	5.72316800	9.84988400
C	25.32230500	5.42378100	11.16053700
C	25.39610600	4.83655700	8.81442000
H	25.56151700	6.12221400	11.96532900
C	24.63829600	4.24138700	11.43190000
C	24.70304300	3.66040800	9.08899400
H	25.70436900	5.06972400	7.79300800
H	24.34482000	4.00867100	12.45795600
C	24.32650000	3.35972400	10.39750000
H	24.45986600	2.97202200	8.27654000
H	23.78766400	2.43404000	10.61211900
C	27.75042200	9.33288000	4.84434600
C	28.05369700	10.59279100	5.26041400
H	27.64980600	8.95860600	3.82656700
H	28.25272900	11.47623900	4.65533300
C	28.41875900	11.80287500	7.40354300
H	29.37464700	12.23699500	7.06862300
H	27.63675500	12.57395400	7.29991200
H	28.51093600	11.52205400	8.46441200
C	27.28355900	7.11534500	5.83773100
H	26.24498500	6.94151100	5.50459200
H	27.95680600	6.61671100	5.12136500
H	27.41977400	6.63771800	6.81755600
B	27.79711900	9.30714400	7.13244300
N	28.09633400	10.61987000	6.64061000
N	27.58912000	8.51508900	5.95093900

C	24.59716400	8.65810300	9.16290000
C	24.69295700	10.18483000	9.14264300
C	24.10946700	8.15493200	7.80108200
C	23.63570300	8.22493700	10.27058700
H	25.10869700	10.55928000	10.08936900
H	25.35264900	10.52190800	8.32747400
H	23.70003900	10.63031800	8.98474700
H	24.02541000	7.05790200	7.79120100
H	23.11742700	8.57162500	7.56919800
H	24.80829100	8.46437000	7.00927500
H	22.65902200	8.70979400	10.12300700
H	23.47365800	7.13860200	10.27352300
H	24.02337600	8.52519200	11.25547600
C	28.88806700	6.23271000	9.88704300
C	28.99579600	6.44454400	11.40086200
C	28.72535500	4.75181600	9.54921200
C	30.14127400	6.78471800	9.20559700
H	29.05597100	7.52203300	11.61794000
H	28.11514900	6.02991000	11.91547700
H	29.89013800	5.94388900	11.80268300
H	28.57714100	4.60973500	8.46764700
H	29.63607200	4.20830900	9.84247800
H	27.87809600	4.29421800	10.07766300
H	31.03549800	6.24453100	9.54900200
H	30.06917800	6.68152100	8.11187600
H	30.26452000	7.85200600	9.44819700
Si	27.68056500	8.89245700	9.07694300
O	28.33515100	9.83831300	10.12361400

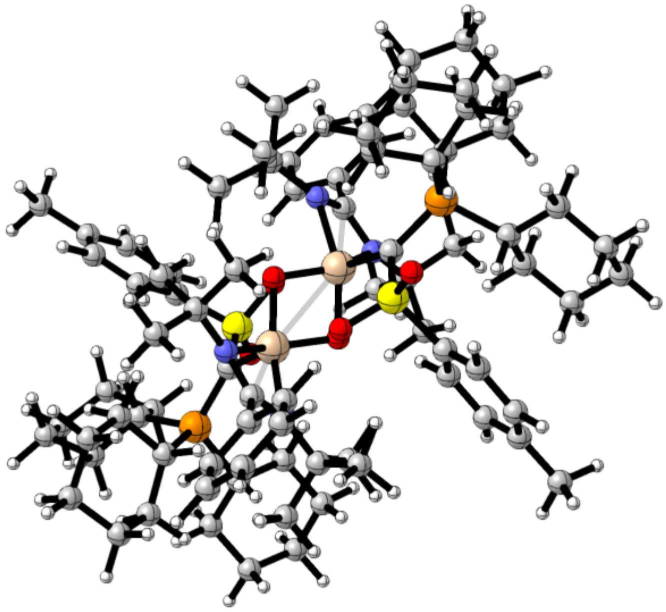

6-Dimer

E = -5923.916346

P	11.961884000	8.632106000	17.047976000
C	12.603327000	10.127814000	17.739006000
Si	11.779374000	11.584247000	18.740196000
Si	13.397695000	13.014310000	20.170772000
O	12.711105000	13.024748000	18.592804000
O	12.435473000	11.563654000	20.347843000
N	9.974768000	10.943914000	19.130742000
N	10.383659000	12.413621000	17.604189000
C	9.429530000	11.866738000	18.332396000
C	12.589701000	14.300584000	21.361728000
N	15.026075000	13.909703000	19.488291000
N	15.014947000	12.419020000	21.055728000
C	15.786673000	13.100494000	20.206631000

C	9.285443000	10.113589000	20.133367000
C	10.347467000	13.632452000	16.771998000
C	7.991960000	12.260948000	18.312851000
P	13.180010000	15.572654000	22.438731000
C	15.330326000	14.632887000	18.234809000
C	15.436958000	11.365195000	21.998722000
C	17.255470000	12.916336000	20.031082000
C	10.370340000	9.402437000	20.932602000
C	8.406173000	10.935781000	21.084539000
C	8.397050000	9.051605000	19.473840000
C	10.428943000	14.894178000	17.639403000
C	9.100525000	13.732958000	15.887223000
C	11.567687000	13.582557000	15.850094000
C	7.568159000	13.355754000	19.076591000
C	7.065556000	11.578933000	17.521639000
C	14.225322000	15.673633000	18.042446000
C	15.298198000	13.693307000	17.027094000
C	16.687047000	15.347046000	18.238663000
C	16.276975000	10.267464000	21.332805000
C	16.219150000	11.976357000	23.168437000
C	14.188987000	10.697779000	22.567618000
C	17.717070000	12.108249000	18.984623000
C	18.181906000	13.534603000	20.872105000
H	11.019664000	8.823616000	20.269465000
H	9.916072000	8.723829000	21.669338000
H	11.012505000	10.124780000	21.439254000
H	8.911963000	11.862508000	21.367942000
H	8.203082000	10.354163000	21.996268000
H	7.440639000	11.192470000	20.629572000
H	8.978618000	8.366618000	18.844146000
H	7.607745000	9.505346000	18.860080000
H	7.905156000	8.449270000	20.252996000
H	11.272627000	14.815872000	18.331946000
H	9.516377000	15.028291000	18.233544000
H	10.554488000	15.777805000	16.994229000
H	9.233378000	14.583574000	15.201699000
H	8.182245000	13.913142000	16.458848000
H	8.962060000	12.832536000	15.275443000
H	11.483813000	12.755704000	15.128510000
H	12.480077000	13.460706000	16.443166000
H	11.640134000	14.517849000	15.275825000
H	8.297222000	13.865531000	19.708139000
C	6.236220000	13.757562000	19.033810000
C	5.733214000	11.983994000	17.481604000
H	7.389940000	10.730529000	16.920126000
H	14.237251000	16.436929000	18.829925000
H	14.359636000	16.184063000	17.077419000
H	13.245774000	15.183077000	18.049483000
H	14.368082000	13.121439000	17.041847000
H	15.357166000	14.282832000	16.099127000
H	16.138165000	12.988343000	17.033202000
H	16.807963000	16.022102000	19.092305000
H	17.532341000	14.648089000	18.231601000
H	16.755715000	15.954090000	17.323323000
H	15.774710000	9.879319000	20.437620000
H	17.276852000	10.618086000	21.049032000
H	16.411206000	9.440360000	22.046813000
H	16.436369000	11.202355000	23.920813000
H	17.179648000	12.396361000	22.843257000
H	15.632019000	12.767410000	23.656825000
H	13.589597000	11.418589000	23.136614000
H	13.564190000	10.294278000	21.761835000
H	14.488576000	9.893363000	23.254851000
H	16.981327000	11.595790000	18.366163000
C	19.082305000	11.948461000	18.773642000
C	19.550189000	13.375224000	20.658158000
H	17.833451000	14.148067000	21.702128000

H	5.915897000	14.612662000	19.633703000
C	5.315249000	13.077680000	18.236624000
H	5.021491000	11.442919000	16.853572000
H	19.427490000	11.315529000	17.952519000
C	20.004586000	12.586462000	19.603921000
H	20.262533000	13.871751000	21.321281000
H	4.271470000	13.399316000	18.205464000
H	21.076738000	12.462144000	19.434112000
S	10.884575000	14.194734000	21.471554000
O	10.250934000	15.500661000	21.785009000
O	10.336883000	13.499607000	20.292928000
C	10.354778000	13.178622000	22.865090000
C	9.266253000	13.591992000	23.630652000
C	10.948312000	11.943708000	23.090864000
H	8.810119000	14.561646000	23.422957000
C	8.789270000	12.760309000	24.641641000
C	10.465818000	11.123996000	24.104436000
H	11.758576000	11.632719000	22.429938000
H	7.934454000	13.082319000	25.243122000
C	9.380552000	11.516976000	24.898683000
H	10.935218000	10.150677000	24.273306000
C	8.887962000	10.639906000	26.011732000
H	9.516914000	10.756585000	26.910353000
H	8.920717000	9.576896000	25.730269000
H	7.856015000	10.888866000	26.297229000
S	14.300400000	10.112904000	17.848503000
O	14.783258000	11.254976000	18.641268000
O	14.883874000	8.816842000	18.271006000
C	15.025560000	10.400653000	16.226062000
C	14.344848000	11.110079000	15.243782000
C	16.343368000	9.996173000	16.019078000
H	13.318292000	11.427778000	15.435162000
C	14.982147000	11.405192000	14.041777000
C	16.973602000	10.304856000	14.817672000
H	16.857693000	9.431001000	16.799508000
H	14.443306000	11.965606000	13.272806000
C	16.304535000	11.009246000	13.807356000
H	18.007211000	9.986050000	14.655385000
C	16.980907000	11.304188000	12.500799000
H	16.919577000	10.437389000	11.821507000
H	18.047951000	11.530533000	12.642226000
H	16.513886000	12.157203000	11.988414000
C	11.686828000	7.258904000	18.278304000
C	11.458349000	5.853865000	17.707748000
C	12.739861000	7.200903000	19.388125000
H	10.741072000	7.582030000	18.749031000
C	11.014387000	4.896084000	18.811181000
H	12.399603000	5.481516000	17.274508000
H	10.718141000	5.850635000	16.898701000
C	12.308457000	6.241024000	20.491172000
H	13.702332000	6.880147000	18.964404000
H	12.928174000	8.206092000	19.789943000
C	12.025812000	4.845704000	19.948981000
H	10.855577000	3.890829000	18.387627000
H	10.035125000	5.226832000	19.203396000
H	13.088340000	6.203125000	21.268540000
H	11.399890000	6.631877000	20.982501000
H	11.668766000	4.180295000	20.751879000
H	12.966747000	4.404590000	19.573259000
C	12.962003000	7.959043000	15.596093000
C	14.226403000	7.145635000	15.920568000
C	12.164517000	7.173541000	14.540227000
H	13.289359000	8.895412000	15.114080000
C	15.075151000	6.946914000	14.668873000
H	13.927029000	6.157484000	16.307460000
H	14.815195000	7.619525000	16.710018000
C	13.000574000	6.972203000	13.277366000

H	11.878645000	6.189405000	14.946079000
H	11.237422000	7.681320000	14.258903000
C	14.304368000	6.242720000	13.562902000
H	15.983427000	6.380573000	14.931520000
H	15.419854000	7.933317000	14.311985000
H	12.403449000	6.427022000	12.527969000
H	13.223229000	7.962247000	12.839293000
H	14.912312000	6.164831000	12.646713000
H	14.078047000	5.207388000	13.876610000
C	10.290485000	9.043938000	16.363605000
C	10.428086000	9.984949000	15.156672000
C	9.355570000	7.847717000	16.124751000
H	9.860069000	9.636617000	17.187943000
C	9.088044000	10.298926000	14.501278000
H	11.098147000	9.559936000	14.394315000
H	10.901175000	10.910297000	15.508362000
C	8.040447000	8.255423000	15.470489000
H	9.829801000	7.095440000	15.481534000
H	9.144026000	7.344640000	17.078944000
C	8.275828000	9.049855000	14.194077000
H	9.259534000	10.893634000	13.589840000
H	8.498014000	10.939001000	15.174576000
H	7.442777000	7.352240000	15.268453000
H	7.445884000	8.860491000	16.176461000
H	7.316540000	9.322644000	13.725561000
H	8.811667000	8.419386000	13.460754000
C	15.027864000	15.733335000	22.485596000
C	15.525822000	16.629050000	21.346205000
C	15.528565000	16.263127000	23.837009000
H	15.402076000	14.705771000	22.340466000
C	17.020461000	16.932644000	21.398218000
H	14.989045000	17.585186000	21.380797000
H	15.270308000	16.155702000	20.394380000
C	17.040617000	16.452663000	23.846452000
H	15.058498000	17.236532000	24.049099000
H	15.240014000	15.591661000	24.653725000
C	17.475396000	17.426803000	22.762732000
H	17.257163000	17.675674000	20.619585000
H	17.593743000	16.032481000	21.132549000
H	17.358488000	16.804698000	24.841046000
H	17.534300000	15.475603000	23.692659000
H	18.568284000	17.567121000	22.775584000
H	17.030555000	18.416945000	22.970514000
C	12.441627000	17.246539000	22.068253000
C	13.007178000	18.500971000	22.746310000
C	12.237283000	17.501818000	20.573650000
H	11.431607000	17.081161000	22.475805000
C	12.021334000	19.655094000	22.562231000
H	13.971073000	18.792357000	22.299525000
H	13.199085000	18.345373000	23.815995000
C	11.256063000	18.650189000	20.376514000
H	13.199622000	17.756413000	20.099461000
H	11.867829000	16.591429000	20.084489000
C	11.723408000	19.914876000	21.089070000
H	12.418547000	20.565518000	23.040685000
H	11.081477000	19.409262000	23.088703000
H	11.116652000	18.844704000	19.300721000
H	10.273279000	18.335852000	20.768986000
H	10.974834000	20.717686000	20.987939000
H	12.642842000	20.285796000	20.599762000
C	12.557542000	15.178246000	24.148081000
C	13.185970000	13.897219000	24.695459000
C	12.451202000	16.276772000	25.209337000
H	11.512106000	14.928902000	23.903534000
C	12.461201000	13.410090000	25.944111000
H	14.246807000	14.061085000	24.944867000
H	13.165764000	13.127489000	23.912224000

C	11.722384000	15.748569000	26.444473000
H	13.445764000	16.642717000	25.509235000
H	11.896295000	17.137058000	24.809838000
C	12.386910000	14.498702000	27.006134000
H	12.962650000	12.510260000	26.335692000
H	11.439918000	13.102795000	25.668097000
H	11.670654000	16.541134000	27.208664000
H	10.679862000	15.507973000	26.169371000
H	11.837383000	14.137331000	27.890506000
H	13.405915000	14.750705000	27.353972000

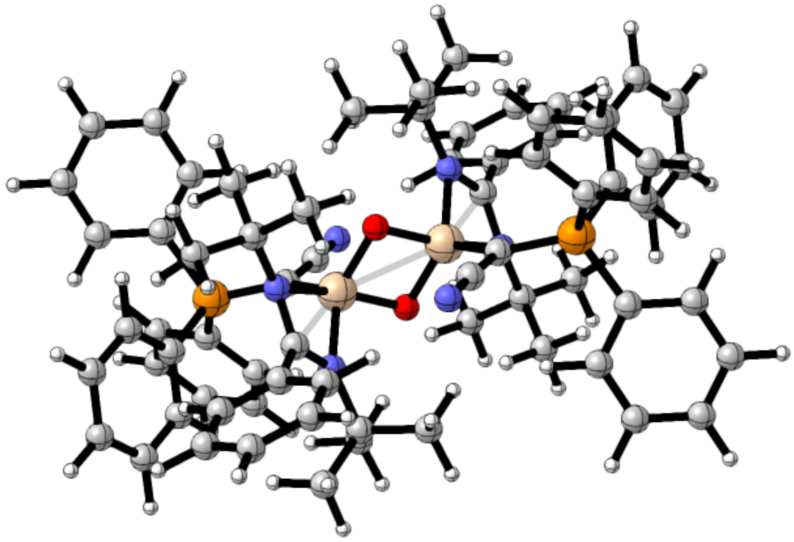

7
E = -4450.066907

P	12.150922000	9.233044000	16.359553000
C	12.660199000	10.329014000	17.568615000
C	10.594329000	8.342470000	16.671734000
C	11.978250000	9.947864000	14.684310000
C	13.398681000	7.903917000	16.190611000
Si	11.828930000	11.164209000	19.010395000
C	10.617643000	7.203869000	17.486004000
C	9.402621000	8.709457000	16.039152000
C	11.356976000	9.279824000	13.619592000
C	12.525911000	11.217716000	14.477799000
C	13.624214000	7.222014000	14.990941000
C	14.122954000	7.548798000	17.335727000
Si	13.299488000	12.904475000	20.169220000
O	11.910456000	12.851859000	19.094490000
O	13.217976000	11.216807000	20.085127000
N	10.756899000	10.082806000	20.072198000
N	9.916604000	11.222300000	18.456358000
C	9.611401000	10.500119000	19.516696000
H	11.546568000	6.900301000	17.972011000
C	9.469820000	6.432597000	17.647961000
C	8.259664000	7.926616000	16.188914000
H	9.369517000	9.601543000	15.412459000
H	10.910381000	8.293850000	13.770333000
C	11.286574000	9.880212000	12.364898000
C	12.458092000	11.811653000	13.219511000
H	12.995908000	11.740807000	15.313142000
H	13.093677000	7.511372000	14.082599000
C	14.548249000	6.180742000	14.943041000
C	15.043775000	6.505810000	17.282697000
H	13.970365000	8.108446000	18.261548000
C	12.468157000	13.739645000	21.610979000
N	14.371622000	13.985652000	19.107481000
N	15.212112000	12.846156000	20.723251000
C	15.517185000	13.568265000	19.662841000

C	11.033776000	9.161891000	21.180448000
C	9.104001000	12.224120000	17.755805000
C	8.252246000	10.237045000	20.053915000
H	9.500353000	5.544567000	18.282946000
C	8.293478000	6.783672000	16.986285000
H	7.335788000	8.218038000	15.684340000
H	10.793972000	9.358925000	11.540684000
C	11.838528000	11.145804000	12.163780000
H	12.887924000	12.804494000	13.069193000
H	14.722664000	5.655514000	14.001111000
C	15.255025000	5.818205000	16.088780000
H	15.609021000	6.240393000	18.178896000
P	12.977655000	14.835014000	22.820493000
C	14.094577000	14.906890000	17.999493000
C	16.024786000	11.844144000	21.423475000
C	16.876265000	13.831258000	19.125392000
C	12.315641000	8.398659000	20.830726000
C	11.257422000	9.977840000	22.456264000
C	9.919683000	8.137373000	21.401615000
C	8.895798000	13.457601000	18.641655000
C	7.738164000	11.688833000	17.314972000
C	9.905467000	12.622967000	16.517124000
C	7.784543000	10.982548000	21.141064000
C	7.449095000	9.245587000	19.485373000
H	7.397540000	6.168750000	17.099673000
H	11.779821000	11.615783000	11.179072000
H	15.982933000	5.004315000	16.047081000
C	14.533962000	15.725971000	22.508063000
C	13.151253000	14.119446000	24.495303000
C	11.729466000	16.163543000	22.990690000
C	12.812861000	15.670183000	18.349693000
C	13.870633000	14.091345000	16.723467000
C	15.208667000	15.931413000	17.778357000
C	16.232713000	10.610747000	20.537405000
C	17.390719000	12.379245000	21.864159000
C	15.223559000	11.445158000	22.662276000
C	17.343726000	13.085796000	18.038127000
C	17.679608000	14.822613000	19.693858000
H	12.156742000	7.773712000	19.940091000
H	12.599077000	7.740915000	21.666247000
H	13.135180000	9.101127000	20.631157000
H	12.072379000	10.694016000	22.285015000
H	11.527578000	9.317769000	23.295838000
H	10.354600000	10.545782000	22.721800000
H	9.667587000	7.616778000	20.465487000
H	9.003062000	8.583994000	21.806387000
H	10.274593000	7.387039000	22.124301000
H	9.867979000	13.847280000	18.967363000
H	8.316177000	13.206243000	19.540806000
H	8.348150000	14.233549000	18.083152000
H	7.269657000	12.418085000	16.636384000
H	7.058357000	11.539969000	18.163458000
H	7.832619000	10.733882000	16.780340000
H	10.057750000	11.764419000	15.846801000
H	10.891001000	13.005149000	16.814453000
H	9.376923000	13.407519000	15.955440000
H	8.417727000	11.765111000	21.574098000
C	6.511266000	10.725987000	21.648947000
C	6.184348000	8.989865000	20.005263000
H	7.823513000	8.672459000	18.638242000
C	14.510209000	16.865163000	21.694655000
C	15.726034000	15.358422000	23.139631000
C	13.772840000	14.787260000	25.559985000
C	12.603953000	12.849413000	24.701554000
C	11.502691000	16.843356000	24.191299000
C	11.005766000	16.520099000	21.845633000
H	12.972048000	16.294733000	19.240572000

H	12.529412000	16.328276000	17.514468000
H	11.993267000	14.967771000	18.549247000
H	13.055716000	13.375111000	16.894616000
H	13.600306000	14.751714000	15.884186000
H	14.773427000	13.523540000	16.457526000
H	15.460866000	16.451901000	18.714519000
H	16.125237000	15.484827000	17.373439000
H	14.853705000	16.681838000	17.055795000
H	15.260376000	10.221467000	20.211657000
H	16.812393000	10.862097000	19.638279000
H	16.780142000	9.834536000	21.095741000
H	17.859423000	11.649599000	22.542190000
H	18.070277000	12.528594000	21.015575000
H	17.296446000	13.333883000	22.399389000
H	15.071498000	12.303594000	23.332778000
H	14.237924000	11.063089000	22.365125000
H	15.752127000	10.660442000	23.223709000
H	16.710443000	12.303333000	17.605077000
C	18.616943000	13.342289000	17.530038000
C	18.944279000	15.078282000	19.173763000
H	17.305382000	15.395700000	20.541110000
H	6.144629000	11.313144000	22.494335000
C	5.711760000	9.730583000	21.089111000
H	5.563088000	8.208411000	19.560609000
H	13.581013000	17.169088000	21.209387000
C	15.657978000	17.636510000	21.532592000
C	16.868938000	16.141299000	22.989750000
H	15.759466000	14.465863000	23.765622000
H	14.219001000	15.773453000	25.409409000
C	13.843973000	14.186387000	26.814406000
C	12.672509000	12.254986000	25.959571000
H	12.133704000	12.326589000	23.866178000
H	12.032705000	16.552763000	25.099552000
C	10.578049000	17.884062000	24.240216000
C	10.084393000	17.562522000	21.899674000
H	11.159156000	15.961878000	20.919078000
H	18.983384000	12.755136000	16.684561000
C	19.416627000	14.337598000	18.089780000
H	19.565645000	15.859696000	19.618337000
H	4.717189000	9.531786000	21.496179000
H	15.627112000	18.525035000	20.898319000
C	16.834700000	17.284863000	22.193279000
H	17.793104000	15.849431000	23.493540000
H	14.336795000	14.707472000	27.638616000
C	13.292435000	12.920569000	27.015260000
H	12.242993000	11.261984000	26.109728000
H	10.402651000	18.407643000	25.182880000
C	9.871936000	18.248092000	23.094564000
H	9.519605000	17.829092000	21.003530000
H	20.411135000	14.536339000	17.682534000
H	17.730598000	17.899828000	22.079811000
H	13.351757000	12.450181000	27.999736000
H	9.143549000	19.061515000	23.137043000
C	14.023917000	10.629159000	17.416029000
N	15.153812000	10.877198000	17.242588000
C	11.104368000	13.439676000	21.763251000
N	9.974465000	13.191562000	21.936499000

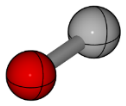

CO
E = -113.231244798

C	-4.51534200	2.57361600	0.00000000
O	-3.38727300	2.57361600	0.00000000

8. References

[22] H. Darmandeh, T. Scherpf, K.-S. Feichtner, C. Schwarz, V. H. Gessner, *Z. Anorg. Allg. Chem.* **2020**, *646*, 835 – 841.

[26b] S. S. Sen, H. W. Roesky, D. Stern, J. Henn, D. Stalke, *J. Am. Chem. Soc.* **2010**, *132*, 1123 – 1126.

[27a] T. Scherpf, R. Wirth, S. Molitor, K.-S. Feichtner, V. H. Gessner, *Angew. Chem. Int. Ed.* **2015**, *54*, 8542 – 8546.

[27b] C. Schwarz, L. T. Scharf, T. Scherpf, J. Weismann, V. H. Gessner, *Chem. Eur. J.* **2019**, *25*, 2793 – 2802.

[30] a) P. Hohenberg, W. Kohn, *Phys. Rev.* **1964**, *136*, B864 – B871. b) W. Kohn, L. J. Sham, *Phys Rev.* **1965**, *140*, A1133 – A1138.

[31] a) C. Adamo, V. Barone, *J. Chem. Phys.* **1999**, *110*, 6158 – 6169. b) A. Schaefer, H. Horn, R. Ahlrichs, *J. Chem. Phys.* **1992**, *97*, 2571 – 2577. c) A. Schaefer, C. Huber, R. Ahlrichs, *J. Chem. Phys.* **1994**, *100*, 5829 – 5835.

[36] G. M. Sheldrick, *Acta Cryst.* **2008**, *A64*, 112.

[37] G. M. Sheldrick, *Acta Cryst.* **2015**, *C71*, 3.

[38] A. Thorn, B. Dittrich, G. M. Sheldrick, *Acta Cryst.* **2012**, *A68*, 448.

[39] G. M. Sheldrick, *Acta Cryst.* **2015**, *A71*, 3.

[40] GaussView, Version 6.1, Roy Dennington, Todd A. Keith, and John M. Millam, Semichem Inc., Shawnee Mission, KS, 2016.

[41] Gaussian 16, Revision C.01, M. J. Frisch, G. W. Trucks, H. B. Schlegel, G. E. Scuseria, M. A. Robb, J. R. Cheeseman, G. Scalmani, V. Barone, G. A. Petersson, H. Nakatsuji, X. Li, M. Caricato, A. V. Marenich, J. Bloino, B. G. Janesko, R. Gomperts, B. Mennucci, H. P. Hratchian, J. V. Ortiz, A. F. Izmaylov, J. L. Sonnenberg, D. Williams-Young, F. Ding, F. Lipparini, F. Egidi, J. Goings, B. Peng, A. Petrone, T. Henderson, D. Ranasinghe, V. G. Zakrzewski, J. Gao, N. Rega, G. Zheng, W. Liang, M. Hada, M. Ehara, K. Toyota, R. Fukuda, J. Hasegawa, M. Ishida, T. Nakajima, Y. Honda, O. Kitao, H. Nakai, T. Vreven, K. Throssell, J. A. Montgomery, Jr., J. E. Peralta, F. Ogliaro, M. J. Bearpark, J. J. Heyd, E. N. Brothers, K. N. Kudin, V. N. Staroverov, T. A. Keith, R. Kobayashi, J. Normand, K. Raghavachari, A. P. Rendell, J. C. Burant, S. S. Iyengar, J. Tomasi, M. Cossi, J. M. Millam, M. Klene, C. Adamo, R. Cammi, J. W. Ochterski, R. L. Martin, K. Morokuma, O. Farkas, J. B. Foresman, and D. J. Fox, Gaussian, Inc., Wallingford CT, 2016.

[42] F. Weigend, R. Ahlrichs, *Phys. Chem. Chem. Phys.* **2005**, *7*, 3297 – 3305.

[43] S. Grimme, J. Antony, S. Ehrlich, H. Krieg, *J. Chem. Phys.* **2010**, *132*, 154104.

[44] S. Grimme, S. Ehrlich, L. Goerigk, *J. Comp. Chem.* **2011**, *32*, 1456 – 1465.

[45] NBO 7.0. E. D. Glendening, J. K. Badenhoop, A. E. Reed, J. E. Carpenter, J. A. Bohmann, C. M. Morales, P. Karafiloglou, C. R. Landis, and F. Weinhold, Theoretical Chemistry Institute, University of Wisconsin, Madison, WI (2018).

[46] R. F. W. Bader, *Chem. Rev.* **1991**, *91*, 893 – 928.

[47] E. R. Johnson, S. Keinan, P. Mori-Sánchez, J. Contreras-García, A. J. Cohen, W. Yang, *J. Am. Chem. Soc.* **2010**, *132*, 6498 – 6506.

[48] T. Lu, F. Chen, *J. Comput. Chem.* **2012**, *33*, 580 – 592.

[49] W. Humphrey, A. Dalke, K. Schulten, *J. Molec. Graphics* **1996**, *14*, 33 – 38.
